# An efficient and chemoselective method to generate arynes†‡

**DOI:** 10.1039/d3sc05429b

**Published:** 2023-11-17

**Authors:** Bryan E. Metze, Riley A. Roberts, Aleksandra Nilova, David R. Stuart

**Affiliations:** a Department of Chemistry, Portland State University Portland OR 97201 USA dstuart@pdx.edu

## Abstract

Arynes hold immense potential as reactive intermediates in organic synthesis as they engage in a diverse range of mechanistically distinct chemical reactions. However, the poor functional group compatibility of generating arynes or their precursors has stymied their widespread use. Here, we show that generating arynes by deprotonation of an arene and elimination of an “onium” leaving group is mild, efficient and broad in scope. This is achieved by using aryl(TMP)iodonium salts (TMP = 2,4,6-trimethoxyphenyl) as the aryne precursor and potassium phosphate as the base, and a range of arynophiles are compatible. Additionally, we have performed the first quantitative analysis of functional group compatibility for several methods to generate arynes, including the method developed here and the current state of the art. Finally, we show that a range of “sensitive” functional groups such as Lewis and Brønsted acids and electrophiles are compatible under our conditions.

## Abstract 2

A chemoselective method to generate arynes by deprotonation of aryl(Mes)iodonium salts with potassium phosphate has been developed and was shown to be one of the most functional group compatible methods to form these reactive intermediates.

## Graphical abstract

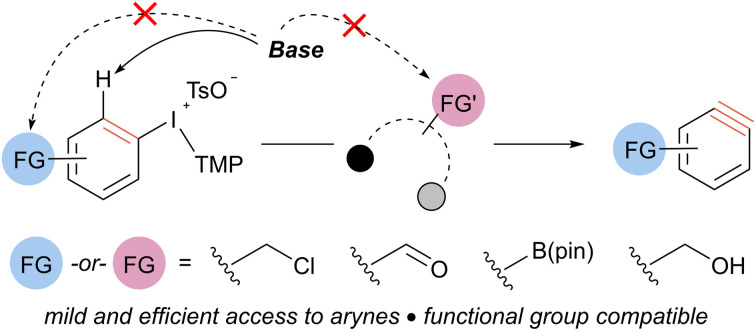

## Introduction

Functional group compatibility is an aspirational goal in the development of chemical reactions for organic synthesis. Indeed, chemoselectivity^1^ underpins the efficient synthesis of complex molecules,^2^ and the application of bio-orthogonal reactions.^3^ Arynes are highly reactive intermediates that continue to attract attention from synthetic chemists because of their diverse reactivity profile.^4^ Although arynes are well-established electrophiles, dienophiles, and dipolarophiles, and the electrophilicity parameter of arynes has been determined,^5^ the functional group compatibility of methods to generate these intermediates remains anecdotal. Here, we describe the formal analysis of functional group compatibility of four methods to generate arynes, including novel conditions for β-elimination by arene deprotonation with a weak base and extrusion of a super leaving group.

**Scheme 1:**
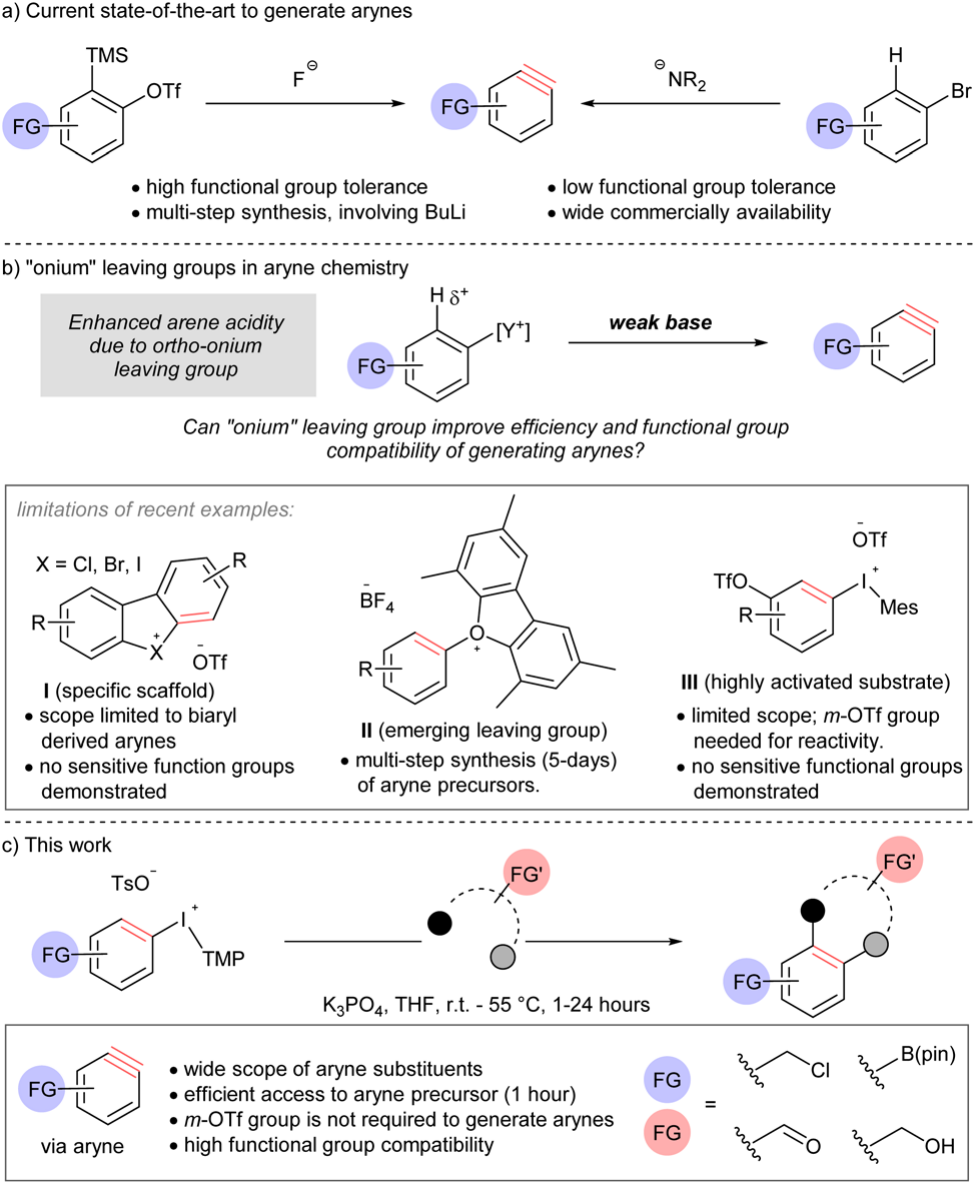
Comparison of methods to generate arynes.

The use of [*o*-trimethylsilyl]phenyl triflate reagents is generally regarded as the current state-of-the-art and the most mild, and therefore functional group compatible, approach to arynes due to the highly chemoselective reaction between the fluoride activator and electrofugal trimethylsilyl leaving group (Scheme 1a).^5*h*,6^ However, the relatively limited commercial availability and multi-step synthesis of these reagents is a drawback to their use; harsh reagents, such as butyllithium, are often a component in these synthetic sequences. On the other hand, accessing arynes by deprotonating an arene and ejection of an *ortho*-leaving group, which is typically a (pseudo)halide, is highly efficient because of the extensive commercial availability of such reagents (Scheme 1a).^7^ However, this approach requires strong bases, such as lithium amides, butyllithium, and metal alkoxides.^7^ As a consequence of multi-step synthesis and use of harsh reagents (*i.e.*, butyllithium), the inclusion of sensitive functional groups on arynes or their precursors is relatively rare Novel methods that address this deficiency, and are compatible with sensitive functional groups, have the potential to open new applications of these versatile intermediates.

Onium substituents have extreme inductive electron-withdrawing effects and have been termed super leaving groups.^8^ As such, onium groups have the potential to activate a proton in the *ortho*-position for deprotonation with a relatively weak and mild base ultimately leading to arynes (Scheme 1b). The use of onium leaving groups to generate arynes has been known for some time,^9^ and although recent advances include more robust synthetic protocols and expanded scope,^10^ they still require relatively strong bases (*e.g.* NaO*t*-Bu or LiHMDS). Reaction conditions to generate arynes using weaker, more mild, bases have begun to emerge but are currently limited to specific scaffolds, emerging leaving groups, or highly activated substrates (Scheme 1b, limitations).^11^ Moreover, there is a distinct lack of sensitive functional groups in the substrate scope of methods using weak base where this strategy would prove most appropriate. Wencel-Delord and others have shown that cyclic diarylbromonium and chloronium salts lead to arynes when treated with metal carbonates at room temperature (I, Scheme 1b).^11*a*–*f*^ Consistent with an updated bonding model for diarylhalonium compounds,^11*d*,12^ the corresponding cyclic diaryliodoniums require substantially elevated temperature (120 °C) to turn on the aryne pathway.^11*f*^ Collectively, these methods are limited to biaryl-derived arynes,^11*a*–*f*^ and arynes that do not have an aryl group appended at the 3-psoition are not possible from this substrates class (I, Scheme 1b). Reactions of mono-substituted aryne precursors with mild bases have also been developed recently. Smith and co-workers described the use of triaryloxonium salts to generate arynes with weak base (II, Scheme 1b).^11*g*^ This report demonstrates that an oxonium leaving group enables the use of a weak base for arene deprotonation and the inclusion of some sensitive functional groups. However, a major limitation of this approach is that the installation of the oxonium leaving group requires substantial material and time investment; a typical synthetic sequence involves (i) S_N_Ar (16 hours), (ii) Suzuki cross-coupling (24 hours), (iii) diazotization and intramolecular *O*-arylation (36–48 hours). Two examples of aryl(Mes)iodonium salts as aryne precursors have been indepedently described by Li and Han (III, Scheme 1b).^11*h*,*i*^ The reaction conditions developed for this class of compound are very similar to those previously developed by Wencel-Delord for cyclic diarylbromonium: metal carbonate, in dichloromethane at room temperature.^11*a*^ The key limitation of this method is that it requires a 3-triflyloxy group to activate the arene for deprotonation. Therefore, although substrates III are more synthetically accessible than II, the scope of arynes is more limited.

Here we describe the discovery and development of a unique set of mild reaction conditions that efficiently generate arynes by ejection of an iodonium leaving group. Specifically, we use aryl(TMP)iodonium salts as the aryne precursor and deprotonate the aryl ring with potassium phosphate (Scheme 1c). Importantly, the aryne precursors described here are synthesized in 1 hour without metals or chromatographic purification and do not require an activating 3-triflyloxy on the aryne precursor for deprotonation of the aryl ring (Scheme 1b and c). A wide range of sensitive functional groups on both aryne and arynophile, including electrophilic benzyl halides and carbonyls, Lewis acidic boronate esters, and Brønsted acidic O–H groups are compatible, and the first quantitative comparison of functional group tolerance of methods to generate arynes is reported.

## Results and discussion

### Method development and scope

**Scheme 2:**
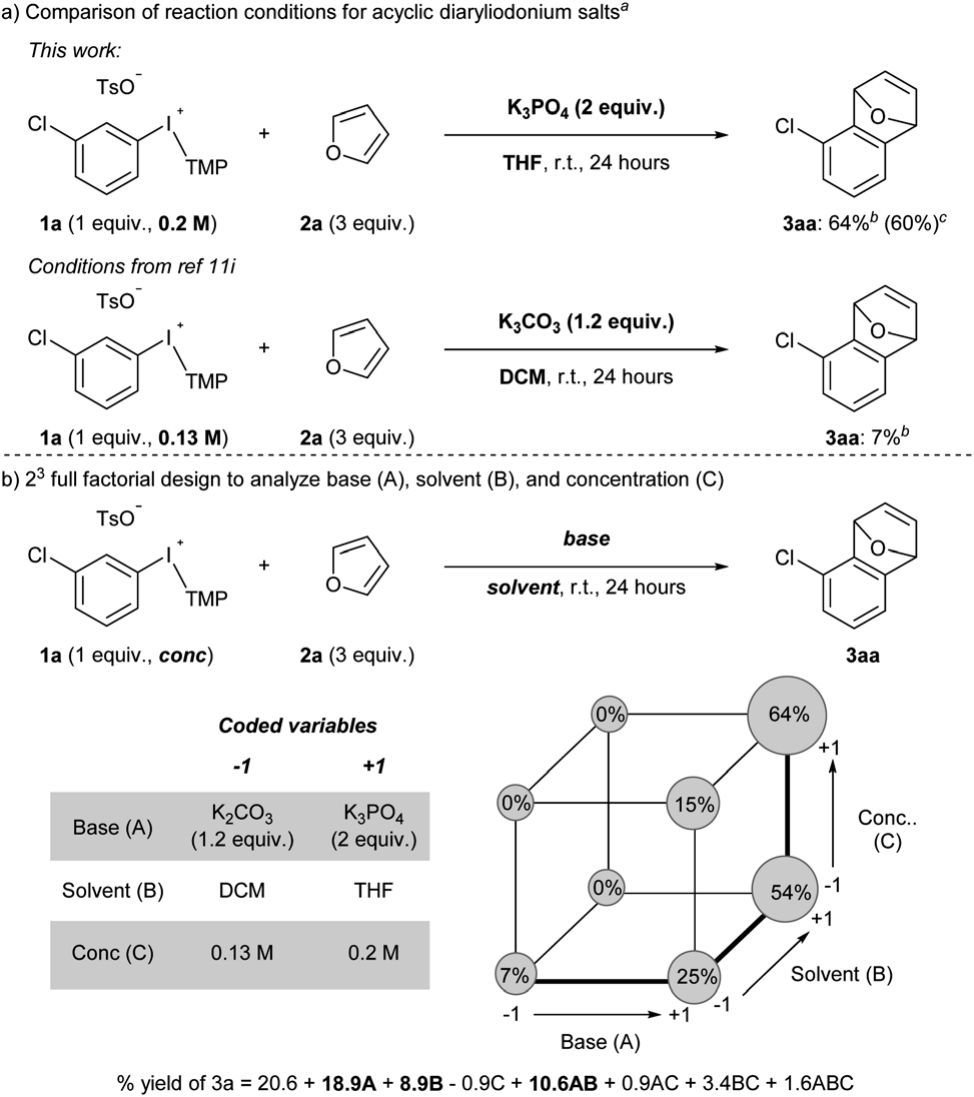
Analysis of reaction conditions. ^*a*^ Reaction conducted on 0.1 mmol of 1a. ^*b*^ Yield determined from crude ^1^H NMR yield. ^*c*^ Reaction conducted on 0.5 mmol of 1a and yield obtained from isolated material after column chromatography.

Acyclic diaryliodonium salts are readily synthesized from simple building blocks and therefore have the potential to provide efficient access to a wide range of arynes.^13^ However, the inherent inertness of acyclic diaryliodonium salts to deprotonation with weak base is a significant hurdle,^11*a*,*d*,*g*^ and recent work has used a neighbouring sulfonyloxy group as an activator to address this challenge.^11*h*,*i*^ Our efforts focused on substrate 1a bearing a chloro-substituent, which is less inductively withdrawing that a triflyl group,^14^ and we identified a set of reaction conditions that generated and trapped an aryne in moderate yield with furan 2a (Scheme 2a). Specifically, we found that K_3_PO_4_ in THF was sufficient to generate an aryne from 0.2 M solution of 1a and we observed 64% yield of 3a in the crude ^1^H NMR spectrum, and obtained 60% yield of 3a upon isolation (Scheme 2a). During the scoping phase of our study, the reports by Li and Han appeared.^11*h*,*i*^ Given the perceived similarity of our substrates (1a and III) and reaction conditions (Scheme 2a), we tested the conditions developed by Han on our substrate 1a.^11*i*,15^ We observed a marked, almost 10-fold, decrease in the yield of 3a when the reaction was conducted with K_2_CO_3_ in DCM at a concentration of 0.13 M (7% NMR yield, Scheme 2a).^11*i*^ The main difference in these conditions is the identity of the base (K_2_CO_3_*vs.* K_3_PO_4_) and the solvent (DCM *vs.* THF) as well as the concentration of 1a (0.13 M *vs.* 0.2 M), and we used Design of Experiment (DoE) to determine which variables had the largest impact on these drastically different yields.^16^ A 2-level full factorial design with base (A), solvent (B), and concentration (C) coded as “−1” or “+1” revealed that base and solvent, and more importantly the combination of base and solvent, have the largest impact on yield (Scheme 2b). The concentration of 1a alone had a marginally negative impact on yield, though interaction effects of concentration with base and solvent ultimately resulted in higher yields when the concentration was high (0.2 M). This analysis illustrates the unique set of conditions that lead to generation of arynes from aryl(TMP)iodonium salts and we have used these and variations of them to establish the scope of this method.

**Scheme 3:**
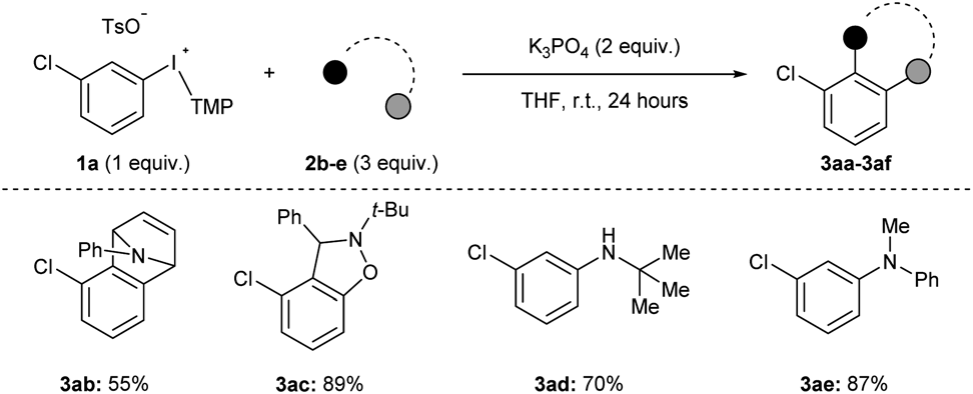
Scope of arynophiles.^*a*^ Conditions: 1a (0.5 mmol, 1 equiv.), 2b–e (1.5 mmol, 3 equiv.), K_3_PO_4_ (1.0 mmol, 2 equiv.), THF (2.5 mL), r.t., 24 hours.

We assessed the scope of this reaction in several different ways. First, using the conditions described in Scheme 2a (top) we surveyed other arynophiles that engage arynes in different types of reactions, *i.e.*, [4 + 2] and [3 + 2] cycloaddition and nucleophilic addition. When other arynophiles that react *via* [4 + 2] cycloaddition were used, such as *N*-phenylpyrrole 2b, a moderate yield of 3ab is observed (55%, Scheme 3). On the other hand, the reaction of nitrone 3c with 3-chlorobenzyne resulted in high isolated yield (89%) of 3ac under our conditions (Scheme 3). Alkyl and aryl amines also function as potent arynophiles under these conditions and we observed high yield of 3ad and 3ae (70% and 87%, respectively; Scheme 3).

**Scheme 4:**
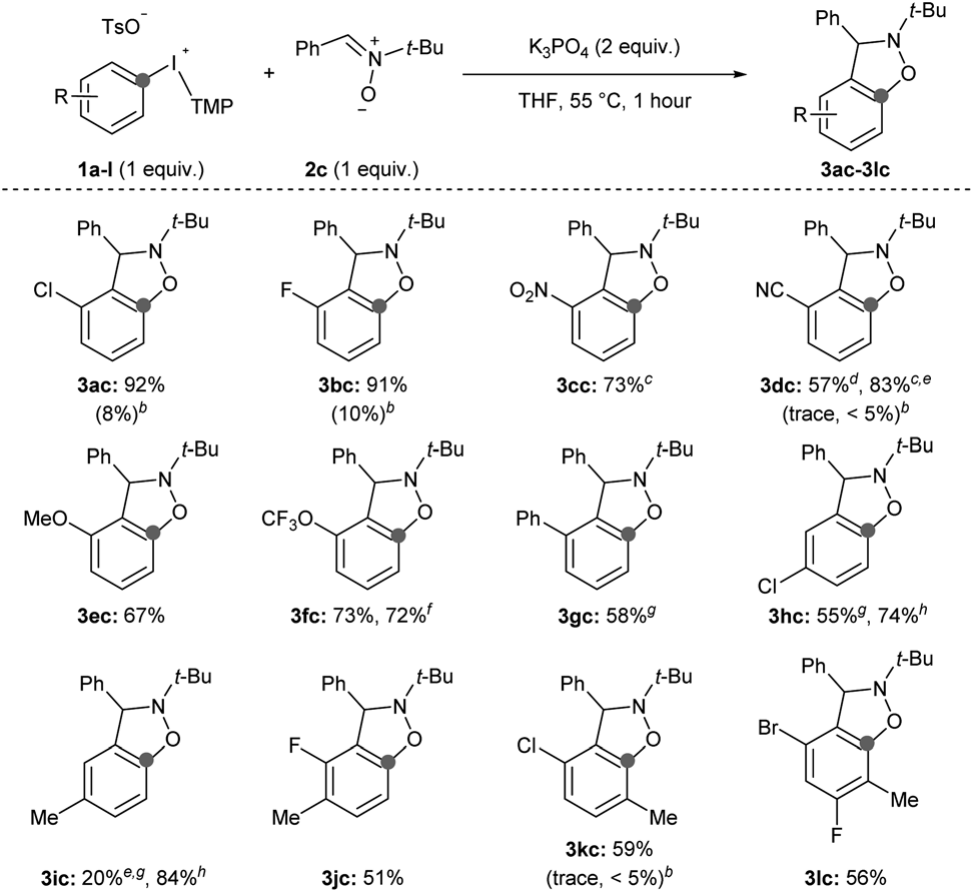
Scope of aryne precursors.^*a*^ Conditions: 1a–l (0.5 mmol, 1 equiv.), 2c (0.5 mmol, 1 equiv.), K_3_PO_4_ (1.0 mmol, 2 equiv.), THF (2.5 mL), 55 °C, 1 hour. ^*b*1^H NMR yield with conditions from ref. 11i. ^*c*^ Reaction conducted at room temperature for 24 hours. ^*d*^ 3-Cyanophenyl(Mes)iodonium tosylate used as aryne precursor. ^*e*^ Yield obtained from the crude ^1^H NMR spectrum. ^*f*^ Reaction conducted on 1.5 mmol scale of 1f for 1.5 hours. ^*g*^ 24 hours reaction time. ^*h*^ Conditions: 1h,i (0.5 mmol, 1 equiv.), 2c (0.5 mmol, 1 equiv.), NaO*t*-Bu (0.75 mmol, 1.5 equiv.), TBME (2.5 mL), r.t., 1 hour.

Second, we surveyed the electronic and steric effects of aryl substituents on the aryne precursors 1a–l (Scheme 4). During the course of surveying arynophiles (Scheme 3) we found that high yields of 3ac were still obtained with 1 equivalent of arynophile 2c in much shorter reaction time (1 hour) at slightly elevated temperature (55 °C).^15^ Under these slightly modified, but more efficient, conditions a range of differently substituted arynes engage with nitrone 2c in a [3 + 2] cycloaddition (Scheme 4). In the products 3ac–lc, the position formerly occupied by the iodonium leaving group is indicated by a grey dot, and both the relative reactivity and regioselectivity of deprotonation and addition to the aryne are influenced by the ring substituents (Scheme 4). We have previously shown that halogens *meta* to the iodonium leaving group activate aryne formation^10*h*^ and here we observed high yield in both cases in which a chloro and fluoro-substituent are placed at this position (3ac and 3bc, Scheme 4). We also observed that nitro (3cc), nitrile (3dc), methoxy (3ec) and trifluoromethoxy (3fc) were compatible inductively withdrawing substituents in the *meta*-position (Scheme 4). In the case of the nitro (3cc) and nitrile (3dc) substituents higher yields were observed when the reactions were conducted at room temperature for 24 hours. Consistent with our previous observations,^10*a*,*g*,*h*^ in all of these cases (1a–f) deprotonation occurs selectively between the substituent and the iodonium leaving group and the aryne forms next to the substituent (Scheme 4). Additionally, in all of these cases (3ac–fc) trapping of the aryne occurs selectively consistent with the aryne distortion model and the negatively polarized end of the nitrone dipole attacks the carbon distal to the σ-withdrawing substituent (Scheme 4).^17,18^ The phenyl substituent in 3gc is substantially less inductively withdrawing than the substituents in 3ac–fc,^14^ yet we still observed selective deprotonation at the more sterically hindered position albeit in moderate yield; this substrate also required extended reaction time of 24 hours (3gc, Scheme 4). Substrates with substituents located *para* to the iodonium leaving group were less reactive. Substrate 1h, with a chloro-substituent, produced aryne adduct 3hc in 55% yield after 24 hours of reaction time; compare this to substrate 1a that yields 92% of 3ac after only 1 hour (Scheme 4). Additionally, substrate 1i, bearing an electron donating methyl substituent in the *para*-position, results in low yield of 3ic (20%; Scheme 4). It is important to note that neither 1h nor 1i have “sensitive” functional groups and therefore using a stronger base, such as NaO*t*-Bu,^10*g*,*h*^ results in substantially higher yields of the aryne adducts 3hc and 3ic (74 and 84%, respectively; Scheme 4). We attempted to improve the low yield of 3ic with weak base by using the acyclic *p*-tolyl(Mes)bromonium and chloronium salts.^15^ However, in both cases complete consumption of the halonium substrates occurred with only trace product of 3ic observed suggesting that these acyclic diarylhalonium salts lack the stability to be efficient aryne precursors.^15^ Aryne intermediates may also facilitate the synthesis of highly substituted benzenoid rings, which are challenging to synthesize by other methods.^19^ Substrates 1j–l with various substitution patterns on the aryne precursor result in tetra and penta-substituted benzenoid products (3jc–lc, Scheme 4). Finally, we also tested several substrates under previously reported conditions (1b, 1d, 1k; Scheme 4).^11*i*^ Using the conditions developed by Han for 3-sulfonyloxyphenyl(Mes)iodonium salts as aryne precursors we observed little to no aryne adducts 3bc, 3dc, or 3kc using diaryliodonium salts that lack a 3-sulfonyloxy group (Scheme 4). Under these conditions, we also observed that aryl(Mes)iodonium salts are useful aryne precursors, albeit forming aryne adducts in slightly lower yield than the corresponding aryl(TMP)iodonium salts;^15^ we consistently observed yields that were approximately 10% lower for the aryl(Mes)iodonium salts. However, in one case we observed that isolation, and therefore yield, were improved by using the aryl(Mes)iodonium salt (3dc, Scheme 4).

### Functional group compatibility

**Scheme 5:**
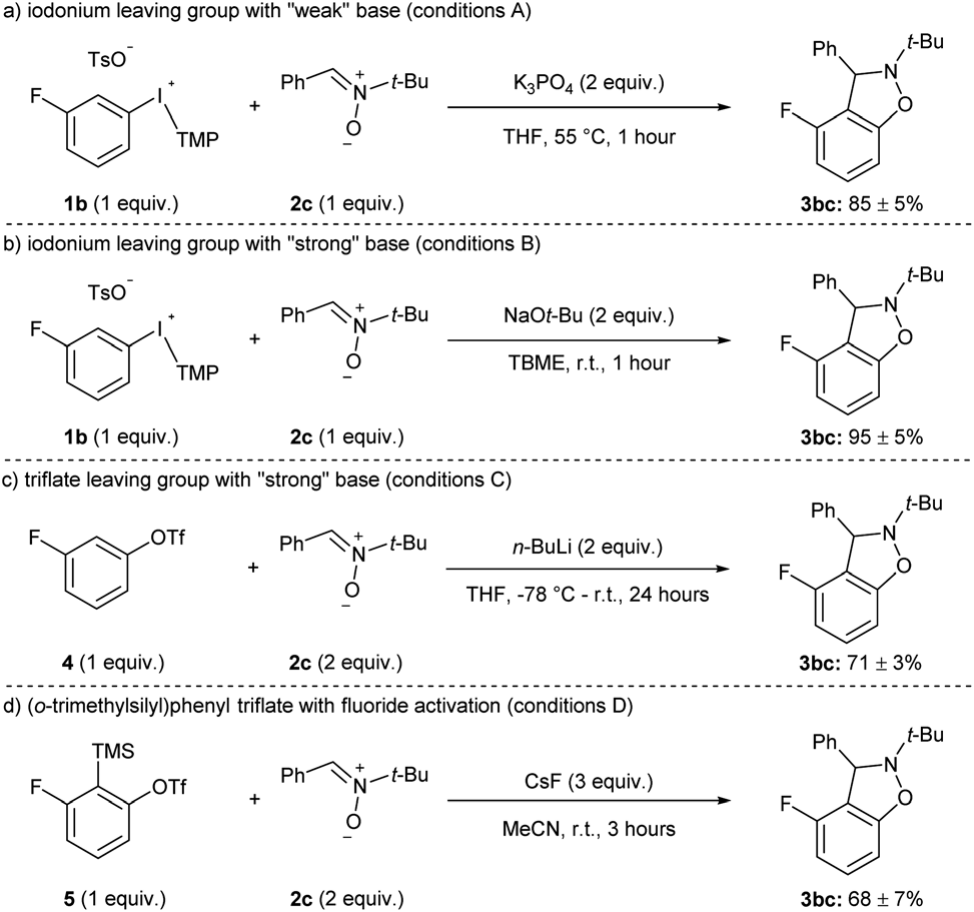
Conditions used in functional group compatibility study.^*a*^ See the ESI‡ for reaction conditions A–D.

The yields of 3hc and 3ic using weak (K_3_PO_4_) and strong (NaO*t*-Bu) base are representative of the current state of the field for methods that have been developed with other weak bases, such as carbonates.^11^ That is, the aryne precursors and arynophiles previously reported rarely have any base-sensitive functional groups that require using a weak base,^11^ and we posit that a strong base could provide higher yields in shorter reaction times in many cases. Indeed, substrates 1a–j and 1l have used as aryne precursors using strong base (LiHMDS or NaO*t*-Bu).^10*g*–*k*,15^ Moreover, although the use of (*o*-trimethylsilyl)phenyl triflates is generally regarded as the most mild approach to arynes,^4*h*,20^ to the best of our knowledge there are no systematic studies of the functional group compatibility of this and other methods to generate arynes. Here, we compared our conditions using weak base with our previous method using strong base and the more common methods of generating arynes. In this analysis, we used aryl(TMP)iodonium salt 1b bearing a fluoride group and the conditions presented in Scheme 4 are considered “conditions A” (Scheme 5a). Our previously reported conditions using NaO*t*-Bu as base are “conditions B” (Scheme 5b).^10*g*,*h*^ In order to assess the functional group compatibility of generating arynes *via* deprotonation of aryl (pseudo)halide with strong base, we used aryl triflate 4 and *n*-BuLi as base and these are considered “conditions C” (Scheme 5c).^21^ We tested other strong bases that are known to generate arynes from aryl triflates, such as LDA and LiTMP, but these were competitive nucleophiles for the aryne with the nitrone arynophile.^15,22^ Finally, we assessed the functional group compatibility of (*o*-trimethylsilyl)aryl triflate 5 with CsF as the activator and these are considered “conditions D” (Scheme 5d).^23^ In each case, using 1b, 4, or 5 results in the same aryne intermediate and product 3bc (Scheme 4 and 5). The yield of 3bc using conditions A–D ranges from 68–95% and are reproducible over three trials (Scheme 5).

**Scheme 6:**
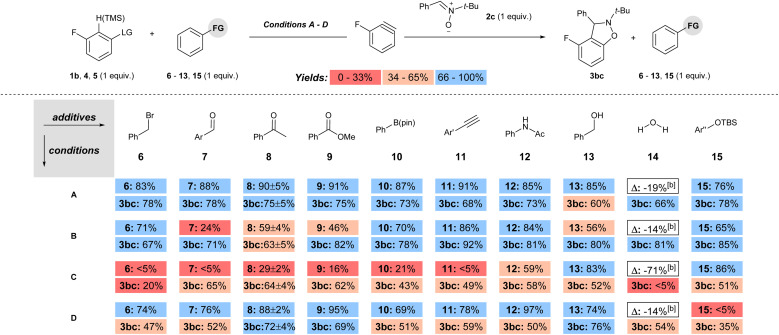
Functional group analysis of methods to generate arynes.

The functional group compatibility was tested for each set of conditions by the method developed by Glorius in which molecules with functional groups of interest are introduced as additives to a model reaction (conditions A–D, Scheme 5).^24^ Additives with electrophilic, Lewis and Brønsted acidic, as well as a protecting group were tested under each set of conditions (6–15, Scheme 6). The yield of the remaining additive (6–15) as well as the product 3bc was quantified for each reaction, and the reproducibility was checked with additive 8 by triplicate runs for each set of conditions.^15^ The yields of both additive (6–15) and product 3bc were colour coded as low (red; 0–33%), moderate (beige; 34–66%), or high (blue; 67–100%) in Scheme 6. Inspection of Scheme 6 reveals several key trends. First, under conditions A, the percent remaining additive 6–13, 15 is high (76–91%), indicating high functional group compatibility (Scheme 6). Moreover, the observed yield of 3bc is high (66–78%) in all but one case in which it is moderate (13, 60%; Scheme 6). Second, the use of 1b with a stronger base (NaO*t*-Bu, conditions B) results in a less functional group tolerant reaction based on the percent remaining additive, which ranges from low to high (24–86%, Scheme 6). However, despite the lower functional group compatibility of this system, moderate to high yields were still obtained for 3bc under conditions B (63–92%, Scheme 6). Third, the use of aryl triflate 4 with strong base (BuLi, conditions C) results in a wide range of percent recovery of the additives (0–85%, Scheme 6).^25^ The recovery of additives is especially low for electrophilic and acidic functional groups, in which a value of <5% refers to trace quantities observed in the crude ^1^H NMR spectra. In conditions C, low to moderate yield of 3bc was observed (0–64%, Scheme 6). Fourth, under conditions D, which are the most common method for generating arynes, the percent recovery of additive was generally high (69–97%) except for the additive 15 bearing a silyl ether protecting group, which was quantitatively consumed (Scheme 6). The yield of 3bc under conditions D ranged from moderate to high (35–76%, Scheme 6). Notably, the addition of H_2_O (14) as an additive had only a modest impact on the yield of 3bc for conditions A, B and D, but completely inhibited the formation of 3bc when BuLi was used as the base (conditions C, Scheme 6).

The functional group compatibility of each set of conditions is summarized in Table 1. Based on the average percent remaining additive the order of functional group compatibility is conditions A > D > B ≫ C (column 3, Table 1). The combination of a super leaving group and a weak base allows for inclusion of a wide variety of base sensitive functional groups. The average percent yield of 3bc for each set of conditions (A–D) over all the additives (6–15) is not a fair comparison because each of the model reactions has a different yield as a starting point (Scheme 5). Therefore, the difference in yield (column 5, Table 1; *Δ*% yield) of 3bc between the model reactions (Scheme 5) and the reactions with additives (as an average, Scheme 6) is a more accurate assessment of functional group compatibility. Incidentally, “*Δ*% yield” generally aligns with functional group compatibility (Table 1). The most chemoselective conditions (A and D) had the smallest difference in yield (13% and 11%, respectively; Table 1, entries 1 and 4), and the least chemoselective conditions (B and C) involving strong base had a substantially larger difference in yield (17% and 25%, respectively; Table 1, entries 2 and 3).

**Table 1:** Summary of functional group compatibility

Entry	Conditions	Avg. % additive	Avg. % 3bc	*Δ*% yield^a^
1	A	86 ± 5%	72 ± 6%	−13%
2	B	62 ± 18%	78 ± 8%	−17%
3	C	33 ± 33%	46 ± 20%	−25%
4	D	72 ± 27%	57 ± 12%	−11%

a Calculated as the difference in average yield of 3bc in the absence or presence of additives (*cf*. average yields from Scheme 5 and column 4 above).

**Scheme 7:**
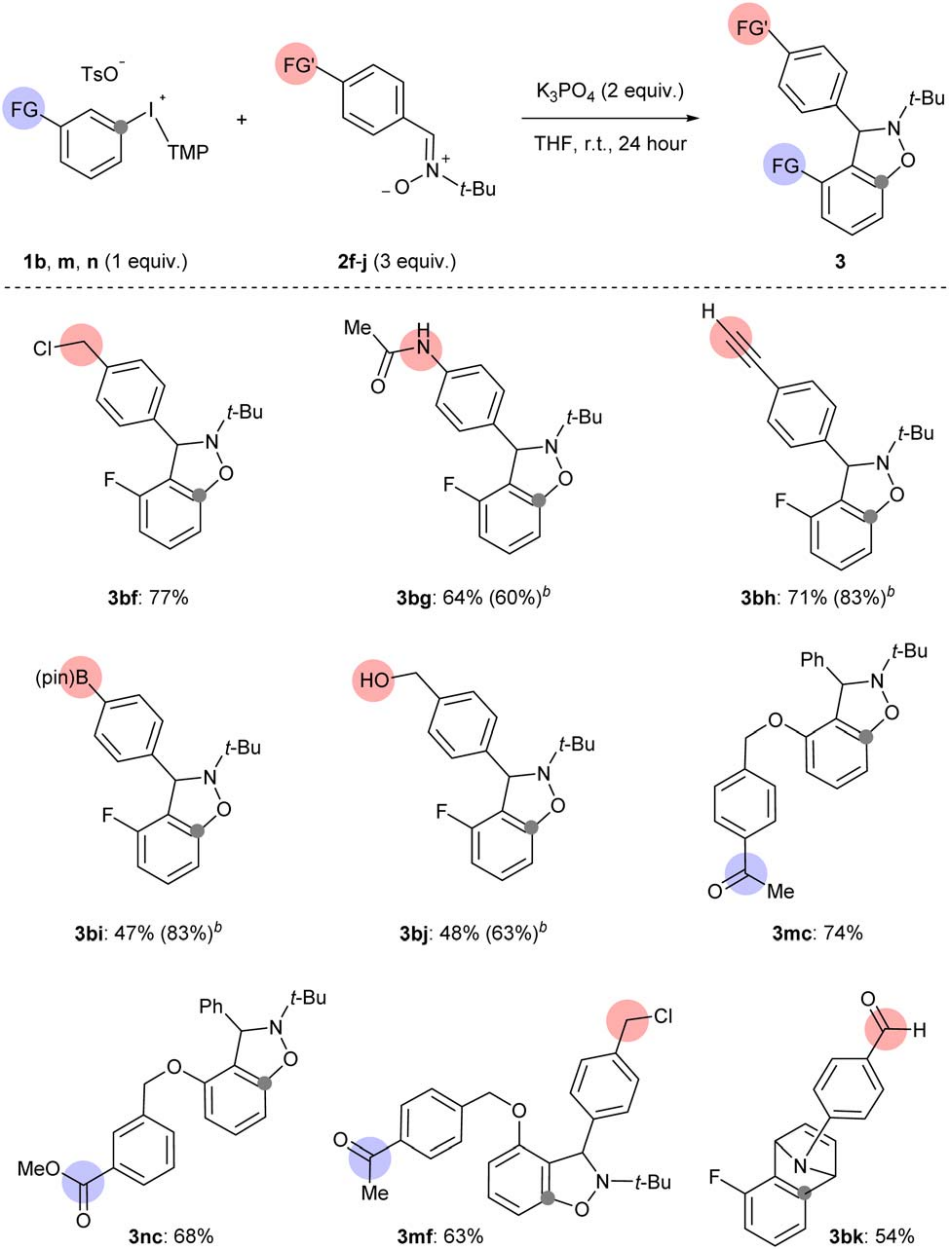
Demonstration of functional group compatibility in generation and trapping of arynes.^*a*^ Conditions: 1 (0.5 mmol, 1 equiv.), 2 (1.5 mmol, 3 equiv.), K_3_PO_4_ (1.0 mmol, 2 equiv.), THF (2.5 mL), r.t., 24 hours. ^*b*1^H NMR yield.

The third way in which we assessed the scope of generating arynes using mild base was to test the functional group compatibility of the reaction with aryne precursors and arynophiles bearing sensitive functional groups that were part of the additives in the analysis above (Scheme 6 and 7). Arynes were successfully generated from 1b and trapped with functionalized nitrones (2f–j) and *N*-arylpyrrole (2k) under our mild base conditions (3bf–bk, Scheme 7). Specifically, aryne-nitrone cycloadducts bearing benzyl chloride (3bf), acetanilide (3bg), terminal alkyne (3bh), pinacol boronate ester (3bi), and benzylic alcohol (3bj), were obtained in moderate to high yield consistent with our functional group compatibility study (Scheme 6 and 7). In the cases of boronate ester (3p) and benzylic alcohol (3q) the isolated yield was reduced by challenging purification, however the ^1^H NMR yields were 83% and 63% for 3bi and 3bj, respectively (Scheme 7). Aryne precursors 1m and 1n bearing ketone and ester groups are compatible in the reaction and lead to aryne-nitrone adducts 3mc and 3nc in 74% and 68% yield, respectively (Scheme 7). We also tested the coupling of acetophenone functionalized aryne precursor 1m and benzyl chloride functionalized aryne trap 2f under our mild base conditions (Scheme 7). The alkylation of acetophenone with benzyl halides under basic conditions has been previously described.^26^ Here we show that our conditions result in chemoselective aryne formation and trapping in the formation of 3mf (63% yield) and both acetophenone and benzyl chloride functional groups remain intact (Scheme 7). An aryne-pyrrole cycloadduct 3bk derived from 1b and *N*-arylpyrrole 2k, which contains an aldehyde, was obtained in 54% yield (Scheme 7). Although this yield is moderate, it compares well with that obtained from unsubstituted *N*-phenylpyrrole 2b (Scheme 3, 3ab), and is consistent with the compatibility of aldehyde functional groups that is suggested in Scheme 6 (88% recovery of additive 7).

Finally, we compared the compatibility of functionalized nitrones with 1b and other basic conditions (NaO*t*-Bu or K_2_CO_3_),^10*H*,11*i*^ or other aryne precursors (4 and 5). The functionalized nitrones included those with an acetanilide 2g, terminal alkyne 2h, and boronate ester 2i (Scheme 7 and Table 2). Synthesis of 3bg using 1b, 4 or 5 (conditions B, C or D, respectively) resulted in lower yields than using 1b and K_3_PO_4_ (Table 2, entries 1–4). Although the yield of 3bg is only slightly lower when 5 is used relative to 1b, it is important to point out that 5 requires multiple synthetic steps (days) to synthesize and 1b requires 1 hour. The results are slightly different for the synthesis of 3bh using 1b, 4, and 5 (Table 2, entries 5–8). In this case, the yield of 3bh is almost the same when 1b is used as the aryne precursor and either K_3_PO_4_ or NaO*t*-Bu is used as the base (Table 2, entries 5 and 6), which is consistent with the results in Scheme 6. However, relative to the synthesis of 3bg, for 3bh there is a larger difference in yield between the reactions that use 1b (and K_3_PO_4_) and 5 as aryne precursors (Table 2, entries 5 and 8). We included another set of conditions that use weak base in a comparison of ways to synthesize 3bi (Table 2, entries 9–13). When 1b was used as the aryne precursor and 2i as the arynophile, the conditions using K_2_CO_3_ (in DCM) resulted in a much lower yield of 3bi than when K_3_PO_4_ (in THF) was used (Table 2, entries 9 and 10).^11*i*^ This result aligns with the low yield that we observed when K_2_CO_3_ (in DCM) was used in the synthesis of 3bc, and reinforces that these conditions are not applicable to generating arynes that lack an activating 3-sulfonyloxy group (Scheme 4). Again, substrates 1b (with NaO*t*-Bu), 4 and 5 (conditions B, C and D, respectively) resulted in much lower yields of 3bi (Table 2, entries 11–13). Collectively, these results are clear evidence that the conditions developed here are uniquely capable of delivering the reactivity and chemoselectivity to generate and trap arynes derived from aryl(TMP)iodonium salts.

**Table 2:** Comparison of compatibility with functionalized nitrones^a^

Entry	Aryne precursor	Conditions	Product	Yield^b^
1	1b	Scheme 7	3bg	60%
2	1b	B	3bg	47%
3	4	C	3bg	21%
4	5	D	3bg	53%
5	1b	Scheme 7	3bh	83%
6	1b	B	3bh	87%
7	4	C	3bh	20%
8	5	D	3bh	66%
9	1b	Scheme 7	3bi	83%
10	1b	Ref. 11i	3bi	25%
11	1b	B	3bi	33%
12	4	C	3bi	7%
13	5	D	3bi	51%

a See ESI for conditions.

b Yield obtained from ^1^H NMR spectrum.

## Conclusions

We have discovered reaction conditions that generate arynes from aryl(TMP)iodonium salts by deprotonation/elimination with K_3_PO_4_ as a weak base. DoE revealed that the solvent (THF *vs.* DCM) and base (K_3_PO_4_*vs.* K_2_CO_3_), and particularly the combination thereof, are uniquely responsible for high yield of aryne adducts with these substrates. This method is a more functional group compatible way to generate arynes than fluoride activation of *o*-trimethylsilylaryl triflates, the current state-of-the-art, based on a systematic analysis of functional group additives to model reactions. The scope of aryl(TMP)iodonium salts as aryne precursors includes groups *meta* to the iodonium leaving group, even marginally withdrawing phenyl groups. Substrates with substituents *para* to the iodonium leaving group are less reactive. The use of a weak and non-nucleophilic base renders sensitive functional groups compatible in this reaction, including benzyl halide, boronate esters and ketones. This work provides new opportunities to generate arynes under conditions that are highly functional group compatible.

## Data availability

The datasets supporting this article have been uploaded as part of the ESI.‡

## Author contributions

DRS, BEM, and AN conceptualized the project. AN established proof-of-concept for this investigation and BEM and RAR completed the investigation. BEM curated the data. DRS wrote the manuscript with input from BEM. All authors have approved the final version of the manuscript.

## Conflicts of interest

There are no conflicts to declare.

## Supplementary Material

SC-014-D3SC05429B-s001
