# Supplementary material for: An efficient and chemoselective method to generate arynes
Source: Chem Sci. 2023 Nov 17;14(47):13885–92. doi: 10.1039/d3sc05429b (PMC10699571; doi:10.1039/d3sc05429b)
Supplement: SC-014-D3SC05429B-s001 [file SC-014-D3SC05429B-s001.pdf]

Electronic Supplementary Material (ESI) for Chemical Science.
This journal is © The Royal Society of Chemistry 2023

An efficient and chemoselective method to generate arynes

Bryan E. Metze, Riley A. Roberts, Aleksandra Nilova*, David R. Stuart

Department of Chemistry, Portland State University, Portland, OR 97201, United States

* Current address: National Center for Advancing Translational Sciences, National Institutes of Health, Rockville, MD 20850, United States

Correspondence should be addressed to

dstuart@pdx.edu

Contents

General Information 2
Synthesis of Known Compounds 2
Comparison of Aryne Yields With "Strong Base" 3
Experimental Design 4
Table of Control Experiments for Coupling with Nitrone 2c 5
Synthesis of 3-chlorophenyl(3,5-dimethylisoxazole)iodonium tosylate (SI-3) 6
Synthesis of 3-Chlorophenyl(thianthrenium) triflate (SI-4) 8
Synthesis of reference standards for LDA and LiTMP mediated reactions (SI-5/6) 9
Synthesis of tert-butyl(4-chlorophenoxy)dimethylsilane (15) 10
Synthesis of Aryl(TMP)iodonium tosylates GP(A) 12
Synthesis of Nitrones GP(B) 14
Synthesis of N-Aryl Pyrrole 2k 17
Synthesis of Aryne-Nitrone Cycloadducts GP(C) 17
Limitations of the K_3PO_4 Aryl(TMP)iodonium tosylate system 32
Deuterium Incorporation Experiment 33
Experiments with halonium salts and literature conditions 34
Robustness Analysis 35
Overlays of ^{1}H-NMR Spectra for Robustness Screen 38
^{1}H, ^{13}C{^{1}H}, and ^{19}F{^{1}H} NMR Spectra 48
References 153

General Information

Materials: Commercially available reagents and solvents were used without further purification unless otherwise stated. A table of manufacturers, including lot #'s, and purity for all chemicals used in the robustness screen is provided on page S-34 of this document. 3-fluorophenyl triflate (**4**) was purchased from oakwood chemicals. The percentage of active oxidant for *m*-CPBA was determined by iodometric titration.[1] n-Butyllithium was titrated using diphenylacetic acid prior to use.[2] The preparation of all other materials is described in detail below.

Methods: Reactions performed above ambient room temperature were done in an oil bath or aluminum block heated externally. Reactions performed below ambient room temperature were done in an ice-water or dry ice-acetone bath. Crude reaction mixtures were analyzed by ^{1}H NMR spectroscopy or thin-layer chromatography (TLC) on Selecto Scientific Flexible TLC plates (silica gel 60 Å F-254) and visualized by UV irradiation, iodine, permanganate, or *p*-anisaldehyde stain. Crude material was purified by flash column chromatography on SiliaFlash P60 silica gel or using a COMBIFLASH RF200 UV/Vis unless otherwise stated. Assay yields were determined using tetrachloronitrobenzene, trimethoxybenzene, or 2,4-dichlorobenzaldehyde as an internal standard. ^{1}H, $^{13}C\{^{1}H\}$, and $^{19}F\{^{1}H\}$/^{19}F spectra were recorded in $CDCl_3$ or DMSO-d_6 with tetramethylsilane as an internal standard. NMR spectra were recorded on a Bruker Avance II 400 MHz spectrometer; the following notation is used: br – broad, s – singlet, d – doublet, t – triplet, q – quartet, p – pentet, m – multiplet, dd – doublet of doublets, dt – doublet of triplets, and ddd – doublet of doublet of doublets. FT-IR spectra were recorded on a Thermo Scientific Nicolet iS5 Infra-red spectrometer. High resolution mass spectrometry (HRMS) data were recorded on Thermo Scientific LTQ Orbitrap Mass Spectrometer by electrospray ionization (ESI). Melting points were recorded on Mel-Temp (Thermo scientific) and are reported as uncorrected.

Synthesis of Known Compounds

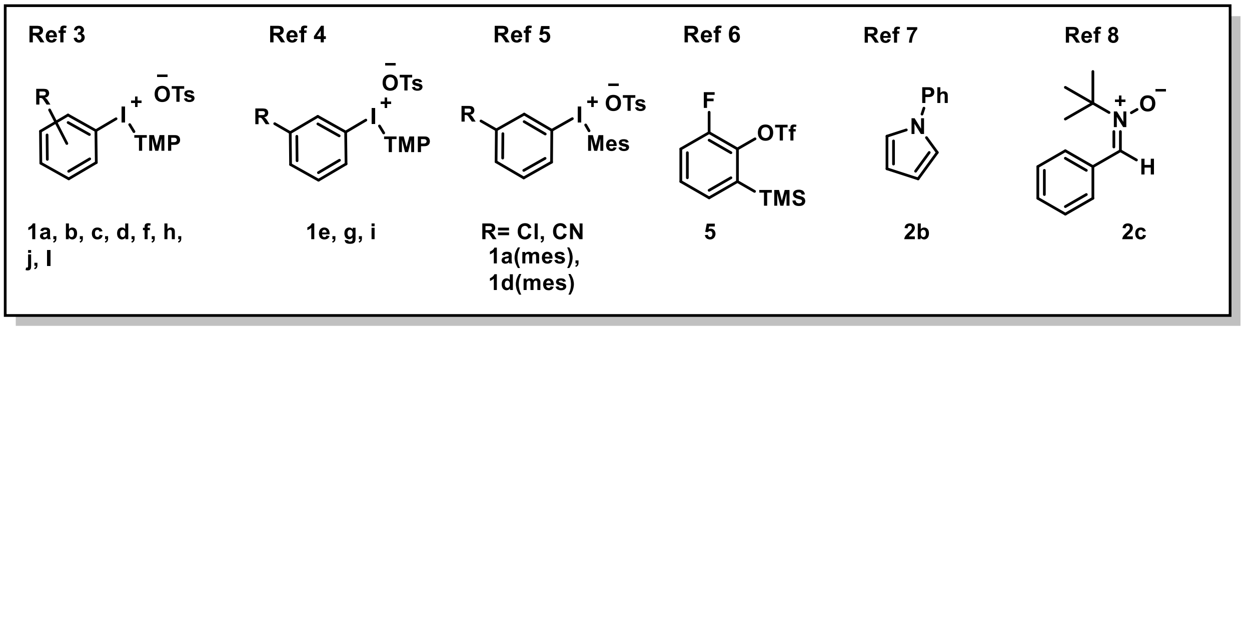

Comparison of Aryne Yields With “Strong Base”

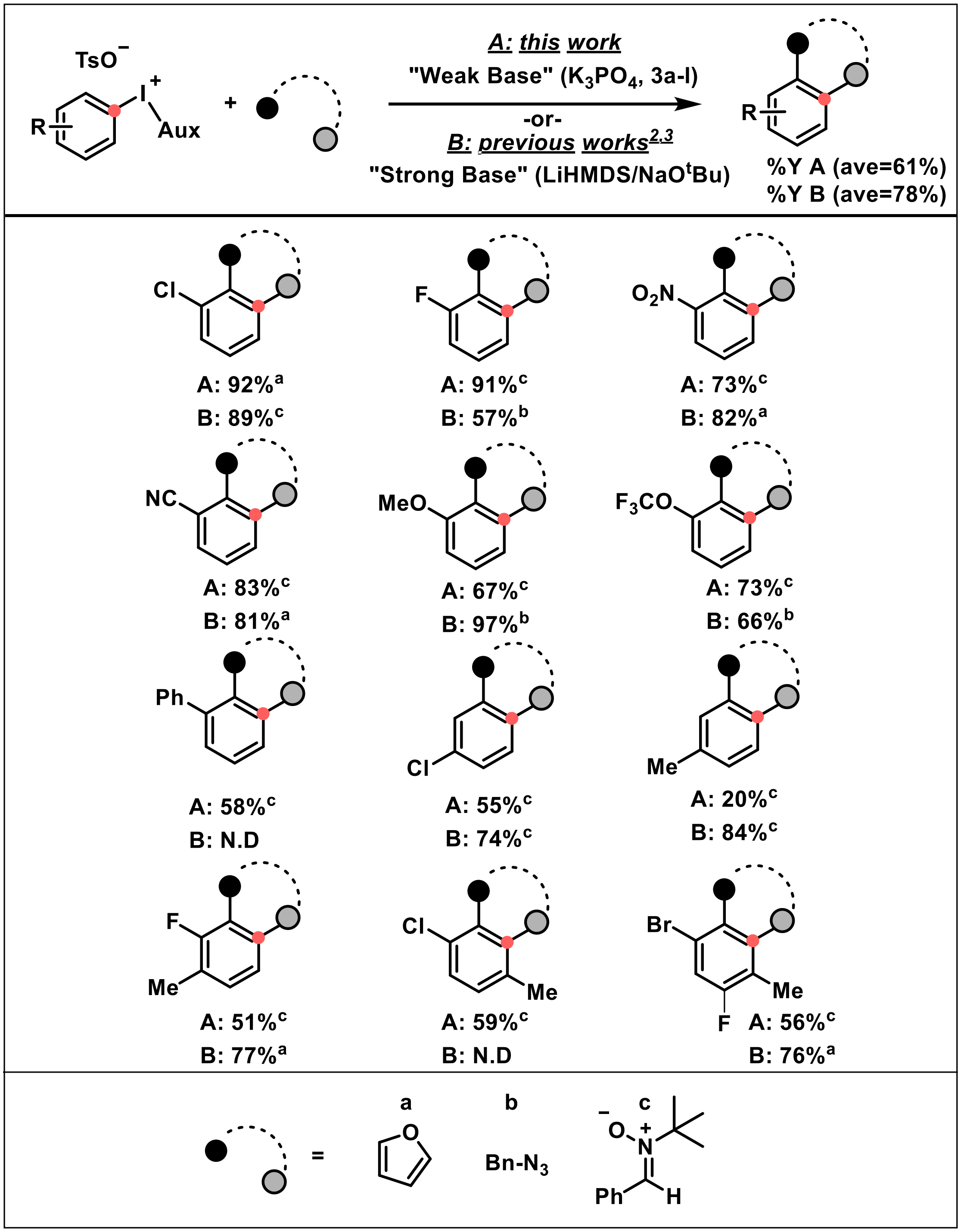

Experimental Design

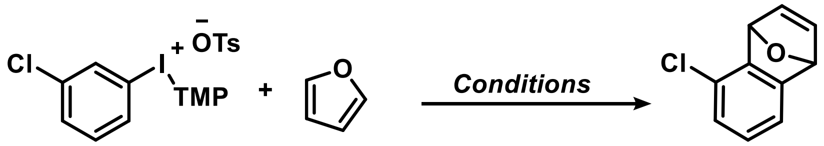

1a (1eq) 2a (5eq) 3aa

Conditions (1) THF, 0.2M, K_3PO_4 (2eq) - this work

Conditions (-1) DCM, 0.125M, K_2CO_3 (1.2eq) ref 9

<u>Constraints</u>

24hr reaction time

Room temperature

Experiment	Base	Solvent	Concentration	Response (%Y 3a)
1	-	-	-	7
2	+	-	-	25
3	-	+	-	Trace (0)
4	+	+	-	54
5	-	-	+	Trace (1)
6	+	-	+	15
7	-	+	+	Trace (0)
8	+	+	+	64

2^3 sign table

i	a	b	c	ab	ac	bc	abc
1	- 1	- 1	-1	1	1	1	-1
1	1	-1	-1	-1	-1	1	1
1	-1	1	-1	-1	1	-1	1
1	1	1	-1	1	-1	-1	-1
1	-1	-1	1	1	-1	-1	1
1	1	-1	1	-1	1	-1	-1
1	-1	1	1	-1	-1	1	-1
1	1	1	1	1	1	1	1

Example Calculation for xA coefficent

xA =1/32 (7(-1)+25(1)+0(-1)+15(1)+0(-1)+64(1)) = 18.9

Response equation

Y=20.6+18.9A+8.9B+(-)0.9C+10.6AB+0.9AC+3.4BC+1.6ABC+e

Table of Control Experiments for Coupling with Nitrone 2c

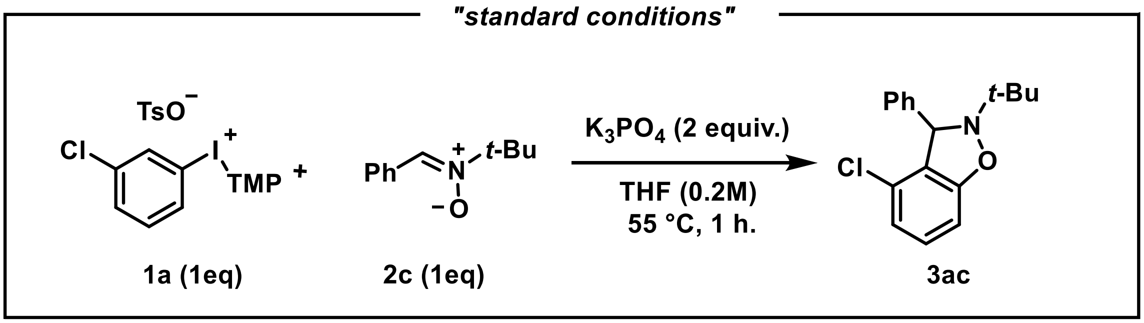

Entry	Deviation from “standard conditions[a]”	Yield 3ac[b]
1	None	88%
2	Mes instead of TMP	79%
3	DMIX instead of TMP	17%
4	TT^+ TfO^- instead of $(TMP)I^+$ TsO^-	9%
5	Cs_2CO_3 instead of K_3PO_4	75%
6	K_2CO_3 instead of K_3PO_4	17%
7	MeCN instead of THF	53%
8	DCM instead of THF	62%
9	Toluene instead of THF	12%
10	r.t. instead of 55 °C	Trace
11	r.t. instead of 55 °C for 24 hours	88%
12	2a (3 equiv.) instead of 2c, r.t., 24 hours	64% 3a

[a] -Conditions: 1a(0.1mmol, 1 equiv.), 2a(0.1mmol, 1 equiv.), K_3PO_4(0.2mmol, 2 equiv.), THF(0.5mL), 55 °C, 1 hour. [b]-Yield determined by ^{1}H-NMR spectroscopy with 2,3,5,6-tetrachloronitrobenzene as an internal standard.

Synthesis of 3-chlorophenyl(3,5-dimethylisoxazole)iodonium tosylate (SI-3)

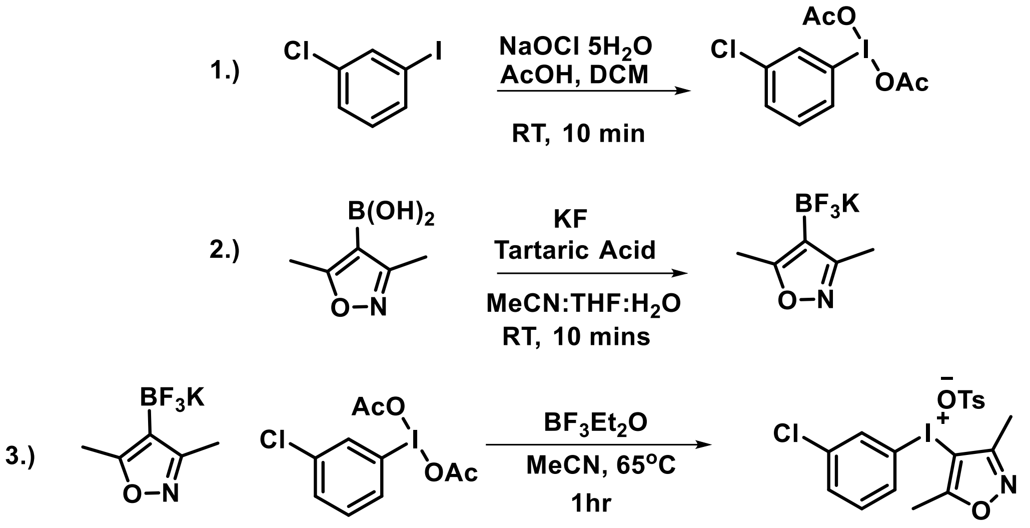

Step 1 was conducted using an adapted literature procedure[10] and spectral data is consistent with previous reports.[11]

NaOCl $5H_2O$ (0.3290g, 2mmol, 1 equiv.) was suspended in DCM (1.5ml) and 3-Chloroiodobenzene (0.4760g, 2mmol, 1 equiv.) was added. AcOH (0.343ml, 3 equiv.) was then added and the mixture was allowed to stir at room temperature for 10 mins. The reaction was then diluted with DCM (ca. 10ml) and filtered. The filtrate was concentrated under reduced pressure and the crude residue was precipitated with diethyl ether to give 3-chloro-diacetoxyiodobenzene in 48% yield (0.3440g, 0.96mmol) in mostly pure form. This solid was recrystallized from AcOH:Ac_2O (10:1) to give analytically pure 3-chloro-diacetoxyiodobenzene (**SI-1**) as translucent polyhedral crystals.

3-Chloroiodobenzene diacetate (SI-1)

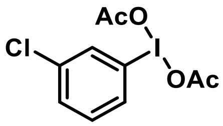

^{1}H NMR (400 MHz, [D6] DMSO) δ = 8.42 (t, J = 1.8 Hz, 1H), 8.21 (d, J = 8.4 Hz, 1H), 7.75 (dd, J_1 = 7.9 Hz, J_2 = 1.4 Hz, 1H), 7.61 (t, J =8.1 Hz, 1H), 1.92 (s, 6H)

^{13}C{^{1}H} NMR (101 MHz, [D6] DMSO) δ = 175.9, 134.9, 134.8, 134.3, 133.0, 132.5, 122.8, 20.6

Step 2 was conducted using an adapted literature procedure.[12]

3,5-dimethylisoxazole boronic acid (0.2818g, 2 mmol, 1 equiv.) was suspended in acetonitrile (8 mL). Potassium fluoride (0.4640g, 8 mmol, 4 equiv.) dissolved in H_2O (0.8 mL) was added at room temperature. With vigorous stirring, tartaric acid (0.6160g, 4.1mmol, 2.05 equiv.) dissolved in THF (1.45ml) was added (the solution was warmed to dissolve tartaric acid in THF, and any remaining solid was transferred to the reaction using 0.2ml H_2O). The reaction was allowed to stir for 10 mins at room temperature after which the mixture was diluted with 10 mL acetonitrile and filtered. The solid was washed with acetonitrile (3 x 10 mL). The filtrate was concentrated under reduced pressure with 10ml of toluene as a cosolvent to give the product (**SI-2**) in 92% yield (0.3735g, 1.84mmol) as a white solid.

Potassium (3,5-dimethylisoxazol-4-yl)trifluoroborate (SI-2)

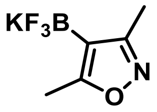

^{1}H NMR (400 MHz, [D6] DMSO) δ = 2.19 (s, 3H), 2.04 (s, 3H)

^{13}C{^{1}H} NMR (101 MHz, [D6] DMSO) δ = 168.0, 163.1, 12.4, 12.1

Note: The ipso-carbon is not observed due to strong quadrupolar relaxation

^{19}F NMR (376 MHz, [D6] DMSO) δ = -134.4 - (-)134.9 (m)

FT-IR 2926, 1609, 1414, 1361, 1260, 1027, 987, 915, 645 cm^{-1}

HRMS (ESI) m/z: $[M - K]^-$ Calculated for $C_5H_6BF_3NO^-$ 164.0495, Found: 164.0489

Melting point decomposition 270 ^oC

Step 3 was conducted using an adapted known literature procedure.[13]

3-Chloro-diacetoxyiodobenzene (0.2200g, 0.62mmol, 1.2 equiv.) was suspended in dry DCM (0.1M) and potassium 3,5-dimethyl-4-(trifluoro-boraneyl)isoxazole (0.102g, 0.514mmol, 1.0 equiv.) was added in one portion. Boron trifluoride diethyl etherate (0.088g, 0.617mmol, 1.2 equiv.) was then added dropwise with stirring. The vial was then sealed and submerged in an oil bath set to 65°C and stirred for 1 hour. The reaction mixture was concentrated under reduced pressure and the crude product was transferred to a separatory funnel using DCM (3 x 5ml). The organic phase was washed with 15% aqueous sodium tosylate (3 x 15ml). The organic phase was dried over $MgSO_4$ and was concentrated under reduced pressure. This material was dissolved in a minimal amount of DCM and was triturated with diethyl ether to afford 3-chlorophenyl(3,5-dimethylisoxazole)iodonium tosylate (**SI-3**) in 63% yield (0.1680g, 0.35mmol) as a white solid.

3-Chlorophenyl(3,5-dimethylisoxazole)iodonium tosylate (SI-3)

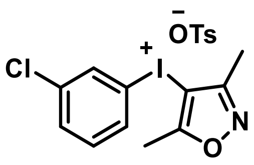

^{1}H NMR (400 MHz, [D6] DMSO) δ = 8.47 (s, 1H), 8.20 (d, *J* = 7.2 Hz, 1H), 7.75 (d, *J* = 6.7 Hz, 1H), 7.55 (t, *J* = 7.6 Hz, 1H), 7.45 (d, *J* = 6.9 Hz, 2H), 7.12 (d, *J* = 6.7 Hz, 2H), 2.71 (s, 3H), 2.35 (s, 3H), 2.28 (s, 3H)

^{13}C{^{1}H} NMR (101 MHz, [D6] DMSO) δ = 176.6, 161.0, 145.7, 138.4, 135.4, 134.5, 134.1, 133.7, 132.8, 128.6, 126.0, 117.7, 86.7, 21.3, 12.8, 11.4

FT-IR 3063, 2972, 2865, 1588, 1571, 1566, 1403, 1379, 1363, 1030, 1005, 783, 679, 561 cm^{-1}

HRMS (ESI) m/z: $[M - OTs]^+$ Calculated for $C_{11}H_{10}ClNO^+$ 333.9490; Found: 333.9467

Melting point 148 - 150 ^{o}C

Synthesis of 3-Chlorophenyl(thianthrenium) triflate (SI-4)

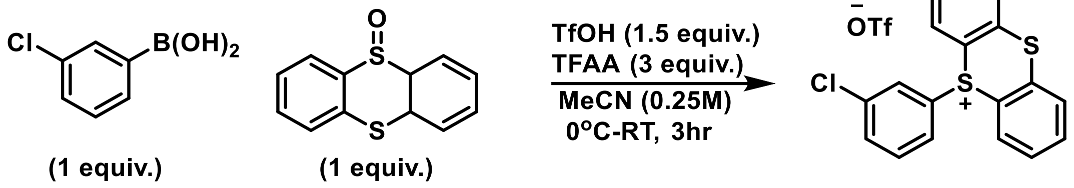

3-chlorophenyl thianthrenium triflate was prepared by adaptation of known literature rocedure.[14]

Thianthrene S-oxide (1.1600g, 5mmol, 1 equiv.) and 3-Chlorophenyl-$B(OH)_2$ (0.7819g, 5mmol, 1 equiv.) were added to a 25ml round bottom flask vial equipped with a magnetic stir bar. MeCN (2ml, 0.25M) was added and the vial was cooled to 0°C in an ice bath. Trifluoromethanesulfonic acid (0.450ml, 7.5mmol, 1.5 equiv.) was added in one portion followed by one portion of trifluoroacetic anhydride (2.1ml, 15mmol, 3 equiv.). The vial was capped and stirred vigorously for 23 hours. Methanol was then added until the reactions color was annihilated. The mixture was then concentrated under reduced pressure to afford an oily residue. This residue was then triturated with diethyl ether and the precipitate was isolated by vacuum filtration and washed by slurry filtration with diethyl ether (3 × 10 mL). After drying under air for 15 minutes, the product (**SI-4**) was obtained in 54% yield (1.2870g, 0.27mmol) as a white solid.

3-Chlorophenyl(thianthrenium) triflate (SI-4)

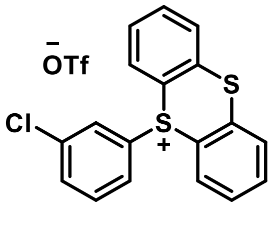

^{1}H NMR (400 MHz, [D6] DMSO) δ = 8.60 (d, *J* = 7.8 Hz, 2H), 8.10 (d, *J* = 7.8 Hz, 2H), 7.94 (t, *J* = 7.6 Hz, 2H), 7.56 (t, *J* = 7.6 Hz, 2H), 7.72 (d, *J* = 8.0 Hz, 1H), 7.55 (t, *J* = 8.1 Hz, 1H), 7.31 (s, 1H), 7.10 (d, *J* = 8.1 Hz, 1H)

^{13}C{^{1}H} NMR (101 MHz, [D6] DMSO) δ = 136.1, 135.6, 135.0, 134.7. 132.8, 132.0, 130.4, 129.7, 127.6, 127.1, 126.9, 121.12 (q, *J* = 322.6 Hz), 119.5

^{19}**F{H} NMR** (376 MHz, [D6] DMSO) δ = -77.7

FT-IR 3067, 1573, 1461, 1258, 1155, 1020, 760, 629, 515, 456 cm^{-1}

HRMS (ESI) m/z: $[M - OTf]^+$ Calculated for $C_{18}H_{12}ClS_2^+$ 327.0063, Found: 327.0034

Melting point 160 - 164 °C

Synthesis of reference standards for LDA and LiTMP mediated reactions (SI-5/6)

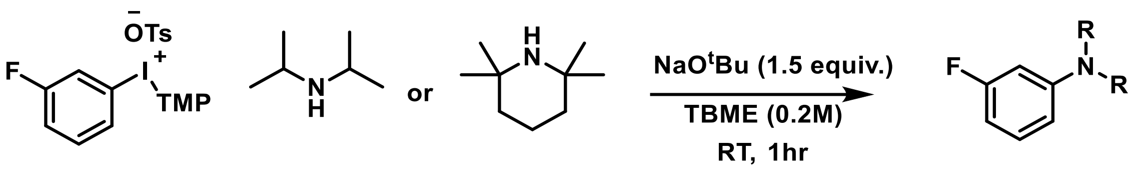

Synthesized by adaptation of known literature procedure.[3]

1b (0.1400g, 0.25mmol, 1 equiv.) was weighed out in air and placed in a 7 mL vial containing a magnetic stir bar. TBME (1.25mL) and amine (0.75mmol, 3 equiv.) were added sequentially via syringe. NaO^{t}Bu (0.0360g, 0.38mmol, 1.5 equiv.) was then added in one portion with constant stirring, the vial was capped, and the reaction allowed to stir for 1 hour at room temperature. The reaction was quenched with a saturated solution of ammonium chloride, the organic layer removed, and the aqueous layer was extracted with ethyl acetate (3 × 5 mL). The combined organic phases were dried with $MgSO_4$ and concentrated under reduced pressure. The crude residue was purified by filtration through a plug of silica eluting with hexanes.

3-fluoro-N,N-diisopropylaniline (SI-5)

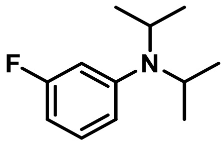

Synthesized according to the procedure above on a 0.25 mmol scale using **1a** and diisoproylamine (0.1058ml, 0.75mmol, 3 equiv.). The desired product (**SI-5**) was obtained in 41% yield (0.0200g, 0.1mmol) as a clear oil.

Rf = 0.65 in 6% EtOAc:Hexanes

^{1}**H NMR** (400 MHz, $CDCl_3$) δ = 7.12-7.00 (m, 1H), 6.60 (dd, J_1 = 8.4 Hz, J_2 = 1.7 Hz, 1H), 6.52 (d, J = 13.4 Hz, 1H), 6.38 (t, J = 8.1Hz, 1H), 3.79 (sep, J = 6.8 Hz, 2H), 1.24 (d, J = 6.8 Hz, 12H)

^{13}**C{^{1}H} NMR** (101 MHz, $CDCl_3$) δ = 163.6 (d, J = 241.3 Hz), 149.9 (d, J = 10.8 Hz), 129.3 (d, J = 10.4 Hz), 112.6 (d, J = 1.5 Hz), 103.7 (d, J = 24.8 Hz), 103.2 (d, J = 21.4 Hz), 47.5, 21.1

^{19}**F{H} NMR** (376 MHz, $CDCl_3$) δ = -113.1

FT-IR 2969, 2927, 2868, 1605, 1577, 1478, 1377, 1362, 1243, 1132, 1036, 1007, 750, 734 cm^{-1}

HRMS (ESI) m/z: $[M + H]^+$ Calculated for $C_{12}H_{19}FN^+$ 196.1496; Found: 196.1482

1-(3-fluorophenyl)-2,2,6,6-tetramethylpiperidine (SI-6)

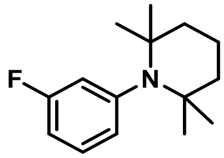

Synthesized according to the procedure above on a 0.25 mmol scale using **1a** and tetramethylpiperidine (0.1276ml, 0.75mmol, 3 equiv.). The desired product (**SI-6**) was obtained in 53% yield (0.0310g, 0.13mmol) as a clear oil.

Rf = 0.9 in 6% EtOAc:Hexanes

^{1}H NMR (400 MHz, $CDCl_3$) δ = 7.17 (m, 1H), 6.98 (d, *J* = 8.3 Hz, 1H), 6.94-6.89 (m, 2H), 1.74-1.68 (m, 2H), 1.55-1.53 (m, 4H), 1.01 (s, 12H)

^{13}C{^{1}H} NMR (101 MHz, $CDCl_3$) δ = 162.7 (d, *J* = 245.5 Hz), 149.2 (d, *J* = 8.7Hz), 130.6 (d, *J* = 8.9 Hz), 130.4 (d, *J* = 2.6 Hz), 121.4 (d, *J* = 17.9 Hz), 113.0 (d, *J* = 20.7 Hz), 54.7, 42.7, 30.1, 18.8

^{19}F{H} NMR (376 MHz, $CDCl_3$) δ = -114.4

FT-IR 2970, 1617, 1571, 1495, 1367, 1296, 1274, 1198, 1139, 826, 753 cm^{-1}

HRMS (ESI) m/z: $[M + H]^+$ Calculated for $C_{13}H_{19}FN^+$ 236.1814; Found: 236.1788

Synthesis of tert-butyl(4-chlorophenoxy)dimethylsilane (15)

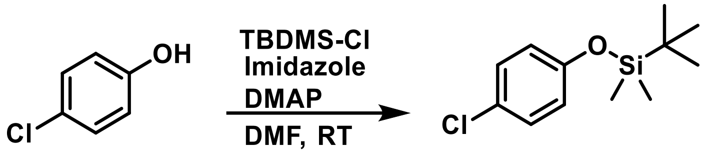

Synthesized by adaptation of known literature procedure and spectral data is consistent with previous reports.[15]

4-Chlorophenol (0.2572g, 2mmol, 1 equiv.) was dissolved in Dry DMF (5.5ml) and tert-butyl-dimethylsilyl chloride (0.4522 g, 3 mmol, 1.5 equiv.), imidazole (0.2720 g, 4 mmol, 2 equiv.) and *N*,*N*-dimethyl-4-aminopyridine (0.0073g, 0.06 mmol, 0.03 equiv.) were added under nitrogen. The mixture was stirred at room temperature until TLC indicated complete conversion of the starting phenol. The reaction mixture was quenched by slow addition of water and the resulting solution was extracted with EtOAc (3X10ml). The combined organic layers were dried over magnesium sulfate and the crude material was purified by filtration through a silica gel plug to yield the desired compound (**15**) in 68% yield (0.3320g, 1.36mmol) as a clear oil.

^{1}H NMR (400 MHz, $CDCl_3$) δ = 7.14 (d, *J* = 8.8 Hz, 2H), 6.76 (d, *J* = 8.8 Hz, 2H), 0.97 (s, 9H), 0.18 (s, 6H)

^{13}C{^{1}H} NMR (101 MHz, $CDCl_3$) δ = 154.5, 129.5, 126.4, 121.6, 25.8, 18.4, -4.3

Synthesis of Aryl Iodides (SI-7/8)

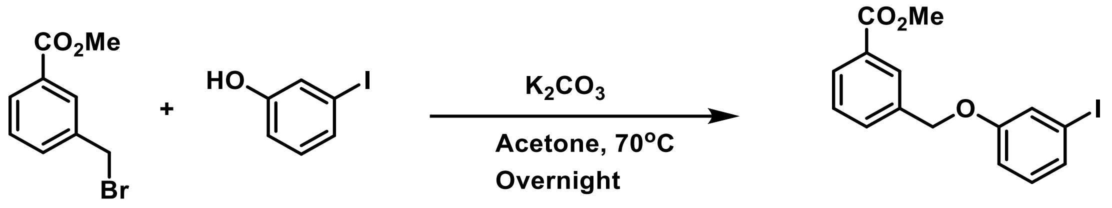

To a 50ml round bottom flask containing a solution of 3-iodophenol (1.1000g, 5mmol) in acetone (0.2M, 10ml) was added Methyl 3-(Bromomethyl)methylbenzoate (1.2600g, 6mmol, 1.2 equiv.) followed by K_2CO_3 (1.3800g, 10mmol, 2 equiv.). The reaction was submerged in an oil bath, a reflux condenser attached, and the temperature set to 70^{0}C. The reaction was allowed to stir at this temperature for 5 hours. Upon completion, the reaction was quenched with saturated sodium bicarbonate (ca. 20ml) and extracted into ethyl acetate. The aqueous phase was extracted with ethyl acetate (3 x 15ml) and the combined organic phases were washed with saturated sodium bicarbonate (15ml) followed by brine (15ml). The organic phase was then dried using $MgSO_4$ and the solution was concentrated under reduced pressure to yield the desired product (**SI-7**) as a white solid in 54% yield (0.9994g, 2.7mmols). The product of this reaction was then used without further purification.

^{1}**H NMR** (400 MHz, $CDCl_3$) δ = 8.10 (s, 1H), 8.02 (d, *J* = 7.8 Hz, 1H), 7.62 (d, *J* = 7.7 Hz, 1H), 7.48 (t, *J* = 7.7 Hz, 1H), 7.35 (d, *J*= 1.8 Hz, 1H), 7.31 (d, *J* = 7.7 Hz, 1H), 7.01 (t, *J* =8.0 Hz, 1H), 9.94 (dd, J_1 = 8.3 Hz, J_2 = 1.6 Hz, 1H), 5.07 (s, 2H), 3.94 (s, 3H)

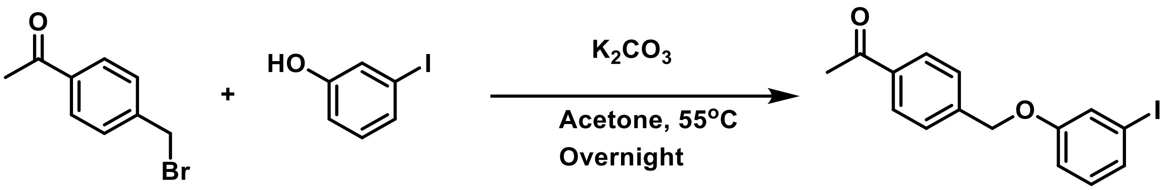

To a 50ml round bottom flask containing a solution of 3-iodophenol (2.200g, 10mmol) in acetone (0.2M, 20ml) was added Methyl 4-(Bromomethyl)acetophenone (2.5570g, 12mmol, 1.2equiv.) followed by K_2CO_3 (2.7600g, 20mmol, 2 equiv.). The reaction was submerged in an oil bath, a reflux condenser attached, and the temperature set to 55^{0}C. The reaction was allowed to stir at this temperature for 24 hours. Upon completion, the reaction was quenched with saturated sodium bicarbonate (ca. 20ml) and extracted into ethyl acetate. The aqueous phase was extracted with ethyl acetate (3 x 15ml) and the combined organic phases were washed with saturated sodium bicarbonate (15ml) followed by brine (15ml). The organic phase was then dried using $MgSO_4$ and the solution was concentrated under reduced pressure to yield the desired product (**SI-8**) as a white solid in 58% yield (2.0416g, 5.8mmols). The product of this reaction was then used without further purification.

^{1}**H NMR** (400 MHz, $CDCl_3$) δ = 8.0 (d, *J* = 8.2 Hz, 2H), 7.51 (d, *J* = 8.1 Hz, 2H), 7.34 (d, *J* = 1.9 Hz, 1H), 7.32 (d, *J* = 8.2 Hz, 1H), 7.01 (t, *J* = 8.0 Hz, 1H), 6.93 (dd, J_1 = 8.3 Hz, J_2 = 2.3 Hz, 1H), 5.10 (s, 2H), 2.62 (s, 3H)

Synthesis of Aryl(TMP)iodonium tosylates GP(A)

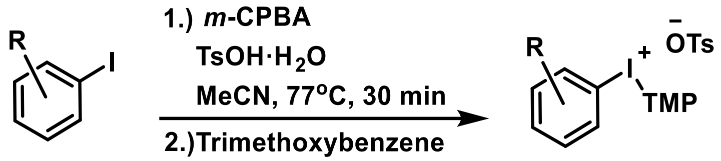

Aryl(2,4,6-trimethoxyphenyl)iodonium tosylates were prepared by known literature procedure.[3]

Aryl iodide (1 equiv.) and acetonitrile (1mL/mmol) were added to an appropriately sized round bottom flask, equipped with a magnetic stir bar. *p* -Toluenesulfonic acid monohydrate (1.1 equiv.) was added in one portion, followed by one portion of m-CPBA (1.1 equiv. active ox). The reaction mixture was submerged in an oil bath set to 77°C and allowed to stir vigorously for 30 minutes. Upon completion of the 30-minute interval, the flask was raised from the oil bath, and 1,3,5- trimethoxybenzene (1.1 equiv.) was added in one portion. The flask was returned to the oil bath and stirring was continued at 77 °C for 5 min. The reaction was removed from heat and the solution was triturated with diethyl ether until precipitation ceased. The precipitate was isolated by vacuum filtration and washed with diethyl ether (3 × 10 mL). After drying under air for 15 minutes the diaryliodonium salt was obtained in analytically pure form. See below for experimental details on new compounds prepared using this method.

Compound 1k

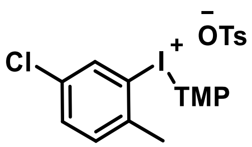

Prepared according to GP(A) from 4-chloro-2-iodo-1-methylbenzene (0.7574g, 3mmol). **1k** was obtained as a white powder in 90% yield (1.6048 g, 2.72 mmol)

^{1}H NMR (400 MHz, [D6] DMSO) δ = 8.12 (d, *J* = 2.2 Hz, 1H), 7.61 (dd, J_1 = 8.2 Hz, J_2 = 2.2 Hz, 1H), 7.54 (d, *J* =8.6 Hz, 1H), 7.46 (d, *J* = 8.0 Hz, 2H), 7.10 (d, *J* = 7.8 Hz, 2H), 6.45 (s, 2H), 3.95 (s, 6H), 3.86 (s, 3H), 2.57 (s, 3H), 2.28 (s, 3H)

^{13}C{^{1}H} NMR (101 MHz, [D6] DMSO) δ = 165.6, 159.8, 146.2, 140.0, 138.0, 136.3, 132.7, 132.6, 131.9, 128.5, 125.9, 121.8, 92.6, 87.3, 57.7, 56.6, 24.6, 21.2

FT-IR 3080, 3037, 2888, 1577, 1494, 1219, 1165, 1032, 1007, 819, 679 cm^{-1}

HRMS (ESI) m/z: [M − OTs]$^+$ Calculated for for $C_{16}H_{17}ClIO_3^+$ 418.9905; Found: 418.9875

Melting point 168 - 170 °C

Compound 1m

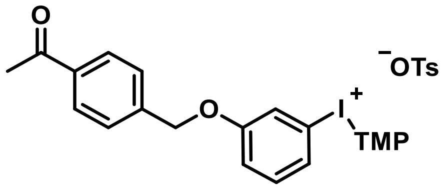

Prepared according to GP(A) from 1-(4-((3-iodophenoxy)methyl)phenyl)ethan-1-one (0.7044, 2mmol) with the following modifications; the reaction was run at room temperature for 2hr for stage 1, and room temperature for 1 hour after addition of TMB. **1m** was obtained in 68% yield (0.9390g, 1.36mmol) as an off-white powder.

^{1}H NMR (400 MHz, [D6] DMSO) δ = 8.03 (d, *J* = 8.0 Hz, 2H), 7.56-7.54 (m, *J* = 8.3 Hz, 3H), 7.47 (m, 7.90 Hz, 3H), 7.39 (t, *J* = 8.0 Hz, 1H), 7.25 (d, *J* = 8.0 Hz, 1H), 7.10 (d, *J* = 7.8 Hz, 2H), 6.45 (s, 2H), 5.25 (s, 2H), 3.93 (s, 6H), 3.87 (s, 3H), 2.58 (s, 3H), 2.28 (s, 3H)

^{13}C{^{1}H} NMR (101 MHz, [D6] DMSO) δ = 197.6, 166.2, 159.4, 159.0, 145.7, 141.6, 137.6, 136.4, 132.4, 128.5, 128.1, 127.5, 126.6, 125.5, 120.4, 118.3, 115.9, 92.0, 86.9, 69.0, 57.3, 56.2, 26.8, 20.8

FT-IR 2974, 1679, 1580, 1471, 1269, 1156, 1063, 1030, 1007, 814, 679 cm^{-1}

HRMS (ESI) m/z: [M − OTs]$^+$ Calculated for $C_{24}H_{24}IO_5^+$ 519.0662 ; Found: 519.0630

Melting point 168 - 170 ^{o}C

Compound 1n

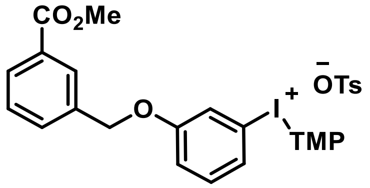

Prepared according to GP(A) from methyl 3-((3-iodophenoxy)methyl)benzoate (0.3682g, 1mmol) with the following modifications; the reaction was run at room temperature for 1hr for stage 1, and room temperature for 1 hour after addition of TMB. **1n** was obtained in 60% yield (0.4240g, 0.6mmol) as an off-white powder.

^{1}H NMR (400 MHz, [D6] DMSO) δ = 8.02 (s, 1H), 7.94 (d, *J* = 7.4 Hz, 1H), 7.71 (d, *J* = 7.5 Hz, 1H), 7.60-7.55 (m, 2H), 7.50-7.45 (m, 3H), 7.40 (t, *J* = 8.1 Hz, 1H), 7.27 (dd, J_1 = 8.3 Hz, J_2 = 1.9 Hz, 1H), 7.11 (d, *J* = 7.9 Hz, 2H), 6.46 (s, 2H), 5.24 (s, 2H), 3.93 (s, 6H), 3.87(3) (s, 3H), 3.87(0) (s, 3H), 2.28 (s, 3H)

^{13}C{^{1}H} NMR (101 MHz, [D6] DMSO) δ = 166.7, 166.5, 159.8, 159.6, 146.2, 138.0, 137.6, 132.9, 132.8, 130.3, 129.6, 129.3, 128.6, 128.5, 127.0, 126.0, 120.9, 118.7, 116.5, 92.5, 87.4, 69.5, 57.8, 56.6, 52.7, 21.2

FT-IR 3093, 2949, 2843, 1715, 1581, 1473, 1456, 1433, 1414, 1284, 1228, 1206, 1007, 711, 562 cm^{-1}

HRMS (ESI) m/z: [M − OTs]$^+$ Calculated for $C_{24}H_{24}IO_6^+$: 535.0612; Found: 535.0603

Melting point 168 - 172 ^{o}C

Synthesis of Nitrones GP(B)

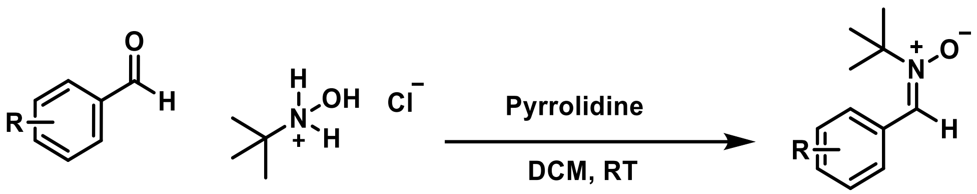

Nitrones were synthesized using known literature procedure.[8]

Aldehyde (1 equiv.) was weighed and added to an appropriately sized round bottom flask equipped with a magnetic stir rod followed by addition of tert-butyl-hydroxylamine hydrochloride (1 equiv.). The mixture was then suspended in DCM (0.33M) and Pyrrolidine (1.2 equiv.) was added via syringe, typically resulting in a homogenous reaction mixture. The reaction was allowed to proceed for 1 hour at room temperature with constant stirring. The reaction mixture was then filtered through a plug of silica, eluting with ethyl acetate. The resulting solution was concentrated under reduced pressure to give the corresponding N-tertbutyl-α-aryl-nitrone which was used without further purification.

Compound 2f

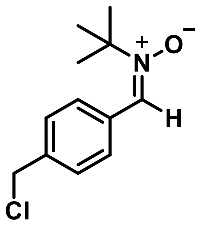

Prepared from 4-(chloromethyl)benzaldehyde (1.6459g, 10mmol) according to GP(B). **2f** was obtained in 72% yield (1.6252g, 7.2mmol) as an off-white solid. Spectral data is consistent with previous reports.[16]

^{1}H NMR (400 MHz, $CDCl_3$) δ = 8.29 (d, *J* = 8.3 Hz, 2H), 7.56 (s, 1H), 7.44 (d, *J* = 8.3 Hz, 2H), 4.60 (s, 2H), 1.62 (s, 9H)

^{13}C{^{1}H} NMR (101 MHz, $CDCl_3$) δ = 139.6, 131.5, 129.8, 129.5, 129.1, 71.5, 46.3, 28.8

Compound 2g

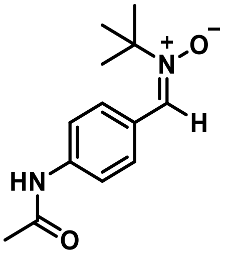

Prepared from 4-acetamidobenzaldehyde (0.8159g, 5mmol) according to GP(B). **2g** was obtained in 89% yield (1.0426g, 4.5mmol) as an off-white solid.

^{1}H NMR (400 MHz, $CDCl_3$) δ = 8.23 (d, *J* = 8.3 Hz, 2H), 8.17-8.08 (br, 1H), 7.58 (d, *J* = 8.7 Hz, 2H), 7.50 (s, 1H), 2.16 (s, 3H), 1.59 (s, 9H)

^{13}C{^{1}H} NMR (101 MHz, $CDCl_3$) δ = 169.2, 140.3, 130.5, 130.4, 127.0, 119.5, 70.5, 28.7, 25.2

FT-IR 3415, 3238, 3160, 3079, 2981, 1690, 1594, 1527, 1507, 1403, 1362, 1309, 1262, 1170, 1095, 1021, 862, 846, 720, 656 cm^{-1}

HRMS (ESI) m/z: $[M + H]^+$ Calculated for $C_{13}H_{19}N_2O_2^+$ 235.1441; Found: 235.1430

Melting point 181 - 186 ^oC

Compound 2h

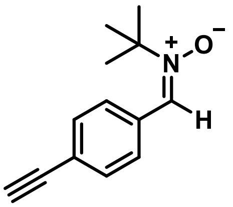

Prepared from 4-ethynylbenzaldehyde (0.3904g, 3mmol) according to GP(B). **2h** was obtained in 73% yield (0.4408g, 2.19mmol) as yellow solid.

^{1}H NMR (400 MHz, $CDCl_3$) δ = 8.24 (d, *J* = 8.4 Hz, 2H), 7.53 (d, *J* = 4.5 Hz, 2H), 7.51 (s, 1H), 3.17 (s, 1H), 1.64 (s, 9H)

^{13}C{^{1}H} NMR (101 MHz, $CDCl_3$) δ = 132.6, 131.7, 129.7, 128.9, 123.9, 84.0, 79.4, 71.7, 28.8

FT-IR 3174, 2976, 2360, 2093, 1567, 1411, 1363, 1187, 1116, 906, 851, 724, 692, 660 cm^{-1}

HRMS (ESI) m/z: $[M + H]^+$ Calculated for $C_{13}H_{16}NO^+$ 202.1226; Found: 202.1215

Melting point 147 - 151 ^oC

Compound 2i

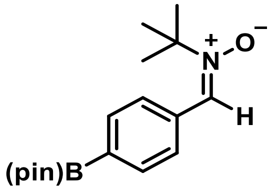

Prepared from 4-(4,4,5,5-tetramethyl-1,3,2-dioxaborolan-2-yl)benzaldehyde (1.1604g, 5mmol) according to GP(B). **2i** was obtained in 81% yield (1.2279g, 4.05mmol) as an off-white solid.

^{1}H NMR (400 MHz, $CDCl_3$) δ = 8.26 (d, *J* = 8.2 Hz, 2H), 7.85 (d, *J* = 8.1 Hz, 2H), 7.57 (s, 1H), 1.62 (s, 9H), 1.35 (s, 12H)

^{13}C{^{1}H} NMR (101 MHz, $CDCl_3$) δ = 135.3, 130.7, 130.5, 128.2, 84.4, 71.7, 28.8, 25.3

Note *One signal is missing due to strong quadrupolar relaxation

FT-IR 2978**,** 1605, 1563, 1355, 1319, 1140, 1118, 1102, 1019, 962, 906, 856, 667, 654 cm^{-1}

HRMS (ESI) m/z: $[M + H]^+$ Calculated for $C_{17}H_{27}BNO_3^+$ 304.2079, Found: 304.2072

Melting point 201 - 204 °C

Compound 2j

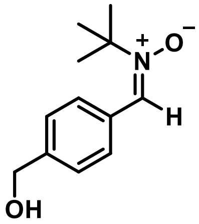

Prepared from 4-acetamidobenzaldehyde (1.2838g, 9.57mmol) according to GP(B). **2j** was obtained in 86% yield (1.7059g, 8.23mmol) as an off-white solid.

^{1}H NMR (400 MHz, $CDCl_3$) δ = 8.18 (d, *J* = 8.2 Hz, 2H), 7.52 (s, 1H), 7.33 (s, *J* = 8.2 Hz, 2H), 4.66 (s, 2H), 2.96 (br, 1H), 1.59 (s, 9H)

^{13}C{^{1}H} NMR (101 MHz, $CDCl_3$) δ = 144.2, 130.8, 130.2, 129.5, 127.0, 71.2, 65.0, 28.7

FT-IR 3293, 2980, 2922, 2864, 1586,1486, 1406, 1390, 1360, 1193, 1173, 1113, 1050, 855, 837, 698, 628, 511

HRMS (ESI) m/z: $[M + H]^+$ Calculated for $C_{12}H_{18}NO_2^+$ 208.1332, found 208.1319

Melting point 85 - 92 °C

Compound 2l

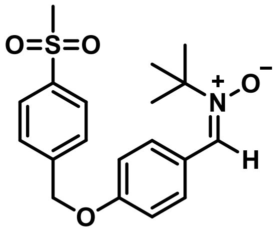

Prepared from 4-((4-(methylsulfonyl)benzyl)oxy)benzaldehyde (0.3670g, 1.26mmol) according to GP(B). **2l** was obtained in 52% yield (0.2370g, 0.66mmol) as an off-white solid.

^{1}H NMR (400 MHz, $CDCl_3$) δ = 8.31 (d, *J* = 8.9 Hz, 2H), 7.97 (d, *J* = 8.3 Hz, 2H), 7.64 (d, *J* = 8.1 Hz, 2H), 7.54 (s, 1H), 7.01 (d, *J* = 8.9 Hz, 2H), 5.22 (s, 2H), 3.07 (s, 3H), 1.62 (s, 9H)

^{13}C{^{1}H} NMR (101 MHz, $CDCl_3$) δ = 159.2, 143.0, 140.1, 130.8, 129.3, 127.8(4), 127.8(0), 124.8, 114.6, 70.3, 69.8, 44.6, 28.3

FT-IR 2981, 2925, 1598, 1502, 1410, 1289, 1236, 1147, 1111, 951, 855, 762, 523 cm^{-1}

HRMS (ESI) m/z: $[M + H]^+$ Calculated for $C_{19}H_{24}NO_4S^+$ 362.1426, found 362.1417

Melting point 174 - 177 ^oC

Synthesis of N-Aryl Pyrrole 2k

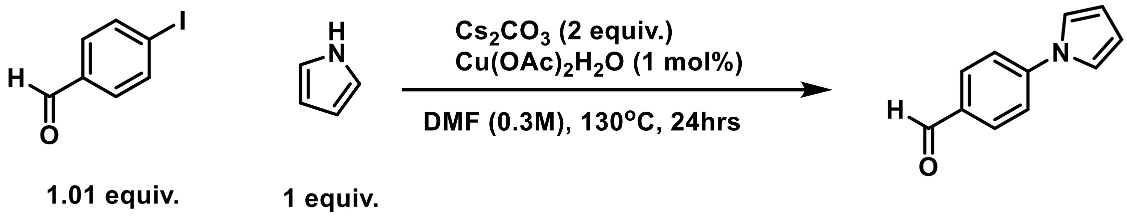

4-iodobenzaldehyde (1.01 equiv.) was weighed and added to an appropriately sized round bottom flask equipped with a magnetic stir rod followed by addition DMF (0.3M) and Pyrrolidine (1.0 equiv. 20mmol). Cs_2CO3 (2 equiv.) was then added followed by $Cu(OAc)_2$ H_2O (1 mol%). The round bottom flask was affixed with a reflux condenser, heated to 130°C in an oil bath and the reaction allowed to proceed for 24 hours with constant stirring. The reaction mixture was allowed to cool to room temperature and was diluted with water and extracted with EtOAc (3x). The combined organic phases were dried over $MgSO_4$ and concentrated under reduced pressure. This crude material was purified by flash chromatography on silica (R_f = 0.5 in 20% EtOAc:Hex). **2k** was isolated after chromatography in 39% yield (1.33g, 7.8mmol) as a yellow solid. Spectral data is consistent with previous reports.[21]

^{1}H NMR (400 MHz, $CDCl_3$) δ = 10.00 (s, 1H), 7.95 (d, *J* = 8.6 Hz, 2H), 7.54 (d, *J* = 8.6 Hz, 2H), 7.20-7.19 (m, 2H), 6.41-6.40 (m, 2H)

^{13}C{^{1}H} NMR (101 MHz, $CDCl_3$) δ = 190.9, 145.0, 133.3, 131.5, 119.7, 119.0, 111.9,

Synthesis of Aryne-Nitrone Cycloadducts GP(C)

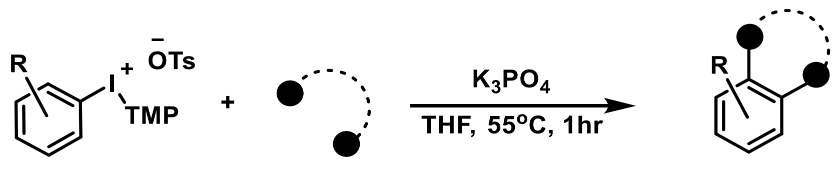

The iodonium salt (0.5mmol, 1 equiv.) was weighed directly into a 12ml glass vial in air and a magnetic stir bar was added. THF (2.5ml, 0.2M) was then added to the vial followed by the arynophile (0.5-1.5mmol, 1-3 equiv.). Anhydrous potassium phosphate tribasic (0.2127g, 1mmol, 2 equiv.) was then weighed out in air and added to the vial in one portion. The vial was then placed in a preheated aluminum block at 55°C with maximum stirring to achieve vigorous mixing of the slurry. The reaction was then allowed to proceed for 1 hour. Upon completion, the reaction was quenched with brine (7ml) and the organic layer removed and filtered through $MgSO_4$. The aqueous phase was then extracted with ethyl acetate (3 X 5ml) and the organic layers were filtered through $MgSO_4$. The combined organic layers were then concentrated under reduced

pressure and the crude reaction mixture was purified by flash chromatography on silica gel using EtOAc or Diethyl ether in hexanes.

Compound 3aa

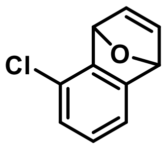

Compound **3a** was prepared according to GP(C) from compound **1a** (0.2884g, 0.5mmol, 1 equiv.) and **2a** (0.1021g, 1.5mmol, 3 equiv.) with the following modification: The reaction was run for 24 hours at room temperature. **3a** was obtained in 60% yield (0.0534g, 0.3mmol) as a white crystalline solid containing a 3% trimethoxybenzene contaminant. Q-NMR gives a purity of 95% for this material. Spectral data is consistent with previous reports.[3]

^{1}H NMR (400 MHz, $CDCl_3$) δ = 7.13-7.11 (m, 1H), 7.09-7.04 (m, 2H), 6.92-6.91 (m, 2H), 5.88 (s, 1H), 5.75 (s, 1H)

^{13}C{^{1}H} NMR (101 MHz $CDCl_3$) δ = 151.5, 147.2, 143.4, 142.5, 126.9, 125.8, 118.5, 82.9, 81.3

Compound 3ab

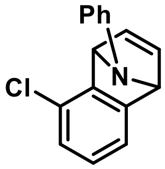

Compound **3ab** was prepared according to GP(C) from compound **1a** (0.2884g, 0.5mmol, 1 equiv.) and **2b** (0.21481g, 1.5mmol, 3 equiv.) with the following modification: The reaction was run for 24 hours at room temperature. **3ab** was obtained in 55% yield (0.0710g, 0.28mmol) as yellow oil. Spectral data is consistent with previous reports.[17]

^{1}H NMR (400 MHz, $CDCl_3$) δ = 7.17 (t, J = 7.9 Hz, 2H), 7.10 (dd, J_1 = 6.1 Hz, J_2 = 1.1 Hz, 1H), 7.0 (m, 2H), 6.89-6.81 (m, 5H), 5.61 (s, 1H), 5.45 (s, 1H)

^{13}C{^{1}H} NMR (101 MHz $CDCl_3$) δ = 150.9, 146.7, 146.4, 142.5, 141.5, 128.9, 128.0, 126.8, 125.6, 121.2, 119.9, 117.9, 70.3, 67.6

Compound 3ac

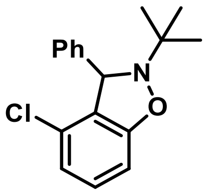

Compound **3ac** was prepared according to GP(C) from compound **1a** (0.2884g, 0.5mmol, 1 equiv.) and **2c** (0.0875g, 0.5mmol, 1 equiv.). **3ac** was obtained in 92% yield (0.1324g, 0.46mmol) as a white crystalline solid containing a 1:10 mixture of regioisomers. This compound was also obtained in 89%

isolated yield using 3 equivalents **2c** at room temperature for 24 hours conditions. Characterization of the major regioisomer is provided below. Spectral data is consistent with previous reports.[3]

^{1}H NMR (400 MHz, $CDCl_3$) δ = 7.33-7.24 (m, 5H, overlaps with solvent residual signal), 7.13 (t, *J* = 8.0 Hz, 1H), 6.80 (d, *J* = 7.9 Hz, 1H), 6.75 (d, *J* = 8.1 Hz, 1H), 5.6 (s, 1H), 1.18 (s, 9H)

^{13}C{^{1}H} NMR (101 MHz $CDCl_3$) δ = 159.0, 142.2, 130.7, 130.0, 128.9, 128.4, 128.1, 127.9, 121.8, 105.8, 67.0, 62.1, 25.8

Compound 3ad

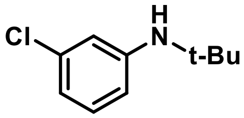

Compound **3ad** was prepared according to GP(C) from compound **1a** (0.2884g, 0.5mmol, 1 equiv.) and **2d** (0.1071g, 1.5mmol, 3 equiv.) with the following modification: The reaction was run for 24 hours at room temperature. **3ad** was obtained in 70% yield (0.0643g, 0.35mmol) as clear oil with a 3% trimethoxybenzene contaminant. Q-NMR gives a purity of 97% for this material. Spectral data is consistent with previous reports.[17]

^{1}H NMR (400 MHz, $CDCl_3$) δ = 7.04 (t, *J* = 8.0 Hz, 1H), 7.00 (d, *J* = 1.8 Hz, 1H), 6.67 (d, *J* = 8.6 Hz, 1H), 6.57 (dd, J_1 = 8.2 Hz, J_2 = 1.4 Hz, 1H), 2.50-4.00 (br, 1H), 1.34 (s 9H)

^{13}C{^{1}H} NMR (101 MHz $CDCl_3$) δ = 148.1, 134.6, 129.9, 117.6, 116.0, 114.7, 51.5, 29.9

Compound 3ae

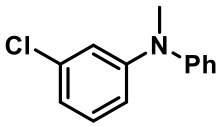

Compound **3ae** was prepared according to GP(C) from compound **1a** (0.2884g, 0.5mmol, 1 equiv.) and **2e** (0.1607g, 1.5mmol, 3 equiv.) with the following modification: The reaction was run for 24 hours at room temperature. **3ae** was obtained in 87% yield (0.0944g, 0.44mmol) as clear oil. Spectral data is consistent with previous reports.[17]

^{1}H NMR (400 MHz, $CDCl_3$) δ = 7.33 (t, *J* = 7.7 Hz, 2H), 7.14-7.10 (m, 4H), 6.88 (s, 1H), 6.82 (d, *J* = 7.8 Hz, 1H), 6.77 (d, *J* = 8.3 Hz, 1H), 3.29 (s, 3H)

^{13}C{^{1}H} NMR (101 MHz $CDCl_3$) δ = 150.3, 148.2, 134.8, 129.9, 129.6, 123.5, 123.4, 119.5, 117.4, 115.8, 40.3

Compound 3bc

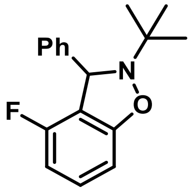

Compound **3bc** was prepared according to GP(C) from **1b** (0.2802g, 0.5mmol, 1 equiv.) and **2c** (0.0886g, 0.5mmol, 1 equiv.). **3bc** was obtained in 91% yield (0.1235g, 0.45mmol) as a white solid. Spectral data is consistent with previous reports.[18]

^{1}H NMR (400 MHz, $CDCl_3$) δ = 7.41 (d, *J* = 7.5 Hz, 2H), 7.32 (t, *J* = 7.4 Hz, 2H), 7.26-7.23 (m, 1H), 7.15-7.09 (m, 1H), 6.61 (d, *J* = 8.1 Hz, 1H), 6.51 (t, *J* = 8.5 Hz, 1H), 5.72 (s, 1H), 1.17 (s, 9H)

^{13}C{^{1}H} NMR (101 MHz, $CDCl_3$) δ = 159.9 (d, *J* = 8.1 Hz), 158.6 (d, *J* = 248.1 Hz) 142.7, 131.0 (d, *J* = 8.5 Hz), 129.0, 128.0, 127.7, 116.8 (d, *J* = 20.3 Hz), 108.3 (d, *J* = 20.3 Hz), 103.2 (d, *J* = 3.4 Hz), 64.9 (d, *J* = 2.3 Hz), 61.9, 25.8

^{19}F NMR (376 MHz, $CDCl_3$) δ = -117.75 (dd, J_1 = 8.5 Hz, J_2 = 5.1 Hz)

Compound 3cc

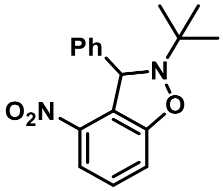

Compound **3c** was prepared according to GP(C) from **1c** (0.2937g, 0.5mmol, 1 equiv.) and **2c** (0.0886g, 0.5mmol, 1 equiv.) with the following modification, the reaction was run at room temperature for 24 hours and **3cc** was obtained in 73% yield (0.1089g, 0.37 mmol) as a yellow crystalline solid. An isolated yield of 54% was obtained when an analogous reaction was run at 55°C for 1 hour.

R_f = 0.27 in 6% EtOAc:Hexanes

^{1}H NMR (400 MHz, $CDCl_3$) δ = 7.71 (d, *J* = 8.2 Hz, 1H), 7.41 (t, *J* = 8.1 Hz, 1H), 7.30-7.17 (m overlaps with solvent residual signal, 6H), 6.17 (s, 1H), 1.20 (s, 9H)

^{13}C{^{1}H} NMR (101 MHz, $CDCl_3$) δ = 160.0, 144.0, 141.1, 130.2, 128.7, 127.8, 127.6, 125.4, 116.6, 113.0, 66.8, 62.0, 25.3

FT-IR 2971, 2937, 2866, 1624,1602, 1533, 1456, 1333, 1207, 1060, 1031, 732, 735, 697 cm $^{-1}$

HRMS (ESI) m/z: $[M + H]^+$ Calculated for $C_{17}H_{19}N_2O_3^+$ 299.1390; Found: 299.1375

Melting point 124 - 125 ^{o}C

Compound 3dc

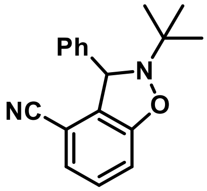

Compound **3dc** was prepared according to GP(C) with the following modification, due to coelution of the TMP-I byproduct with **3dc**, **1d(Mes)** (0.2597g, 0.5mmol, 1 equiv.) was used with **2c** (0.0886g, 0.5mmol, 1 equiv.). Compound **3dc** was obtained in 56% yield (0.0780g, 0.280 mmol) as a 1:5 mixture of regioisomers appearing as white crystalline solid. Characterization of the major regioisomer is described below. This compound gives 57% assay yield with the corresponding TMP salt. Using GP(C) and using the TMP salt at room temperature for 24hrs gives an assay yield of 83% **3dc**.

R_f = 0.30 in 10% EtOAc:Hexanes

^{1}H NMR (400 MHz, $CDCl_3$) δ = 7.41-7.33 (m, 4H), 7.30-7.28 (m, 1H), 7.25 (t, overlaps with solvent residual signal, *J* = 8.0 Hz, 1H), 7.09 (d, *J* = 7.6 Hz, 1H), 7.04 (d, *J* = 8.1 Hz, 1H), 5.73 (s, 1H), 1.18 (s, 9H)

^{13}C{^{1}H} NMR (101 MHz, $CDCl_3$) δ = 157.3, 141.4, 132.6, 129.7, 128.7, 128.1 127.9, 125.0, 121.2, 111.6, 107.8, 66.6, 61.7, 25.3

FT-IR 3063, 3033, 2974, 2226, 1581, 1447, 1366, 1263, 1235, 1206, 982, 770, 725, 697 cm^{-1}

HRMS (ESI) m/z: $[M + Na]^+$ Calculated for $C_{18}H_{18}N_2ONa^+$ 301.1311, Found: 301.1305

Melting point 51 - 67 ^oC

Compound 3ec

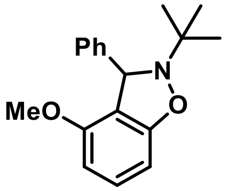

Compound **3ec** was prepared according to GP(C) from **1e** (0.2862g, 0.5mmol, 1 equiv.) and **2c** (0.0886g, 0.5mmol, 1 equiv.). **3ec** was obtained in 67% yield (0.0949g, 0.335 mmol) as a white crystalline solid.

R_f = 0.31 in 6% EtOAc:Hexanes

^{1}H NMR (400 MHz, $CDCl_3$) δ = 7.36 (d, *J* = 8.7 Hz, 2H) 7.30-7.26 (m, overlaps with solvent residual signal, 2H), 7.22-7.19 (m, 1H), 7.13 (t, *J* = 8.1 Hz, 1H), 6.50 (d, *J* = 8.0 Hz, 1H), 6.36 (d, *J* = 8.1 Hz, 1H), 5.61 (s, 1H), 3.66 (s, 3H), 1.17 (s, 9H)

^{13}C{^{1}H} NMR (101 MHz, $CDCl_3$) δ = 158.8, 155.5, 143.1, 130.1, 128.2, 127.5, 127.0, 116.3, 103.3, 99.9, 64.9, 61.3, 55.4, 25.4

FT-IR 3029, 3009, 2980, 2937, 2866, 1621, 1599, 1494, 1390, 1365, 1332, 1059, 1032, 846, 709, 679 cm^{-1}

HRMS (ESI) m/z: $[M + H]^+$ Calculated for $C_{18}H_{22}NO_2^+$ 284.1645; Found: 284.1624

Melting point 89 - 91 ^oC

Compound 3fc

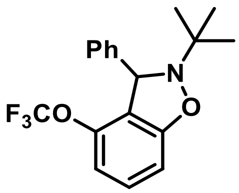

Compound **3fc** was prepared according to GP(C) from **1f** (0.3132g, 0.5mmol, 1 equiv.) and **2c** (0.0886g, 0.5mmol, 1 equiv.). **3fc** was obtained in 73% yield (0.1231g, 0.37mmol) as a slightly yellow crystalline solid. **3fc** was also prepared on a 1.5 mmol scale and a yield of 72% was obtained.

R_f = 0.45 in 6% EtOAc:Hexanes

^{1}H NMR (400 MHz, $CDCl_3$) δ = 7.28-7.17 (m, overlaps with solvent residual signal, 5H), 7.12 (t, J = 8.1 Hz, 1H), 6.70 (d, J = 8.0 Hz, 1H), 6.70 (dd, J_1 = 8.1 Hz, J = 1.1 Hz, 1H), 5.60 (s, 1H), 1.10 (s, 9H)

^{13}C{^{1}H} NMR (101 MHz, $CDCl_3$) δ = 159.0, 144.6 (q appearing as doublet, J = 1.4 Hz), 141.9, 130.4, 128.5, 127.6, 127.2, 121.9, 120.7 (q, J = 240.1 Hz), 112.6, 105.5, 65.2, 61.6, 25.3

^{19}F{^{1}H} NMR (376 MHz, $CDCl_3$) δ = -57.48

FT-IR 3069, 3035, 2982, 2937, 1610, 1456, 1390, 1365, 1327, 1236, 1170, 1076, 1035, 884, 773, 695 cm^{-1}

HRMS (ESI) m/z: $[M + H]^+$ Calculated for $C_{18}H_{19}F_3NO_2^+$ 338.1362, Found: 338.1337

Melting point 42 - 48 ^oC

Compound 3gc

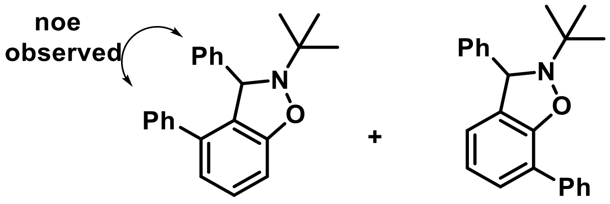

1.5 **1**

Compound **3gc** was prepared according to GP(C) from **1g** (0.1546g, 0.25mmol, 1 equiv.) and **2c** (0.0440 g, 0.25mmol, 1 equiv.) with the following modifications, the reaction time was 24 hours. The material after column purification was further purified by prep TLC. **3gc** was obtained in 58% yield (0.047g, 0.145 mmol) as a yellow oil containing a 1:1.5 mixture of regioisomers. (M (major) or m*(minor)).

R_f = 0.1 in 100% hexanes

^{1}H NMR (400 MHz, $CDCl_3$) δ = 7.76 (d, *J* = 7.3 Hz, 1H m*), 7.59-7.56 (m, 1H m*), 7.46-7.31 (m, 10H m*), 7.25-7.23 (m, 4H M), 7.10-7.05 (m, 5H M), 7.05-7.02 (m, 1H, m*) 6.91-6.84 (m, 3H M), 6.80 (d, *J* = 7.5 Hz, 1H M), 5.66 (s, 1H, M), 5.65 (s, 1H m*), 1.21 (s, 9H), 1.20 (s, 9H)

^{13}C{^{1}H} NMR (101 MHz, $CDCl_3$) δ = Major Isomer resonances: 157.6, 143.1, 139.8, 138.7, 129.1, 128.2, 128.1, 128.0(9), 127.4, 127.3(6), 127.2, 126.9, 121.8, 105.7, 66.1, 61.3, 25.5 (overlaps with major isomer)

Minor Isomer Resonances: 143.9, 140.4, 136.7, 130.9, 130.1, 128.8, 128.6, 128.4, 122.7, 121.2,122.1, 121.5, 117.7, 117.6, 66.9, 61.0, 25.5 (overlaps with major isomer)

FT-IR 3030, 2973, 1585, 1582, 1493, 1472, 1453, 1426, 1364, 1265, 1205, 1108, 1074, 1028, 910, 755, 696 cm^{-1}

HRMS (ESI) m/z: $[M + H]^{+}$ Calculated for $C_{23}H_{24}NO^{+}$ 330.1852, Found: 330.1843

Compound 3hc

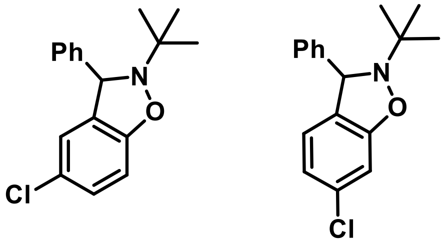

2 : **1**

Compound **3hc** was prepared according to the GP(C) from **1h** (0.2884g, 0.5mmol, 1 equiv.) and **2c** (0.0886g, 0.5mmol, 1 equiv.) with the following modifications, the reaction was allowed to proceed for 3 hours at 55 °C, **3hc** was obtained in 55% yield (0.0806g, 0.28mmol) as a 1:2 mixture of regioisomers appearing as a white solid. **3hc** was also obtained in 74% yield in an analogous fashion using 1.5 equivalents of sodium tert-butoxide in TBME (0.2M) for 1 hour at room temperature. Characterization of the regioisomeric mixture is provided below. (M (major) or m*(minor)).

R_f = 0.6 in 5% EtOAc:Hexanes

^{1}H NMR (400 MHz $CDCl_3$) δ = 7.39-7.31 (m, 4H M, 4H m*), 7.29-24 (m, 1H M, 1H m*, overlaps with solvent residual signal), 7.09 (dd, J_1 = 8.5 Hz J_2 = 2.2 Hz, 1H M), 6.83 (d, *J* = 2.4 Hz, 1H, M), 6.79-6.77 (m, 3H, m*), 6.71 (d, *J* = 8.5 Hz, 1H M), 5.55 (s, 1H, M), 5.54 (s, 1H, m*), 1.16 (s, 9H M, 9H m*)

^{13}C{^{1}H} NMR (101 MHz, $CDCl_3$) δ = 157.5, 155.3, 143.8, 143.6, 134.6, 132.2, 129.3, 129.2(1), 129.2(0), 129.1, 129.2, 128.1, 127.9, 127.7, 125.8, 124.7, 124.2, 121.4, 108.3, 107.9., 67.3, 67.1, 61.7(3), 61.7(0), 25.9 – 1 peak is missing due to overlap

FT-IR 2973, 2933, 1595, 1472, 1390, 1232, 1204, 1172, 1064, 911, 834, 696 cm^{-1}

HRMS (ESI) m/z: $[M + H]^{+}$ Calculated for $C_{17}H_{19}ClNO^{+}$ 288.1150, Found: 288.1142

Melting point 68 - 74 °C

Compound 3ic

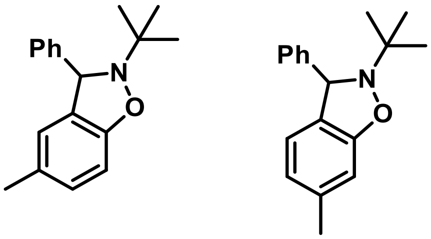

1 : 1

Compound **3ic** was prepared from **1i** (0.2782g, 0.5mmol) and **2c** (0.0886g, 0.5mmol, 1 equiv.) using sodium tert-butoxide (0.1442g, 1.5 equiv.) and TBME (2.5ml, 0.2M) as solvent for 1 hour at room temperature. **3ic** was obtained in 84% Yield (0.1123, 0.42mmol) as a 0.56:0.44 mixture of regioisomers appearing as a white solid. An assay yield of 20% **3ic** (1:1 mixture of isomers) was obtained using GP(C) for 24 hours. Characterization of the mixture of isomers is provided below.

R_f = 0.8 in 20% EtOAc:Hexanes

^{1}H NMR (400 MHz $CDCl_3$) δ = 7.41-7.37 (m, 2H), 7.35-7.30 (m, 2H), 7.27-7.22 (m, 1H, overlaps with solvent residual signal), 6.84 (d, *J* = 8.3Hz, 0.5H), 6.76 (d, *J* = 7.7 Hz, 0.5H), 6.70-6.61 (m, 2H), 5.54 (s, 1H), 2.30 (s, 1.5H), 2.20 (s, 1.5H), 1.17 (s, 9H)

^{13}C{^{1}H} NMR (101 MHz, $CDCl_3$) δ = 156.5, 154.2, 144.1, 144.0, 138.9, 130.0, 129.9, 129.1, 128.6(0), 128.5(8), 127.4, 127.3, 127.1, 124.0, 123.2, 121.5, 107.3, 106.3, 67.1, 66.8, 61.0, 60.9(8), 25.5, 21.5, 20.7

FT-IR 3035, 2973, 2864, 1596, 1487, 1455, 1477, 1255, 1204, 1153, 1074, 1031, 943, 902, 853, 794, 776, 718, 697 cm^{-1}

HRMS (ESI) m/z: $[M + H]^+$ Calculated for $C_{18}H_{22}NO^+$ 268.1701 , Found: 268.1683

Melting point 79 - 85 ^oC

Compound 3jc

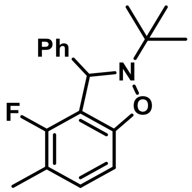

Compound **3jc** was prepared according to GP(C) from **1j** (0.2954g, 0.5mmol, 1 equiv.) and **2c** (0.0886g, 0.5mmol, 1 equiv.). **3jc** coeluted with a benzaldehyde contaminant and was ultimately obtained in 51% yield (0.0890 g, 0.30 mmol) as a white crystalline solid after washing with $NaHSO_3$ (3 x 3ml).

R_f = 0.66 in 10% EtOAc:Hexanes

^{1}H NMR (400 MHz, $CDCl_3$) δ = 7.41 (d, *J* = 7.4 Hz, 2H), 7.32 (t, *J* = 7.4 Hz, 2H), 7.26-7.23 (m, 1H), 6.96 (t, *J* = 7.8 Hz, 1H), 6.51 (d, *J* = 8.1 Hz, 1H), 5.69 (s, 1H), 2.12 (d, *J* = 1.8 Hz, 3H), 1.17 (s, 9H)

$^{13}C\{^1H\}$ NMR (101 MHz, $CDCl_3$) δ = 157.4 (d, *J* = 13.4 Hz), 156.1 (d, *J* = 253.0 Hz), 142.4, 131.8 (d, *J* = 5.6 Hz), 128.5, 127.5, 127.3, 116.8 (d, *J* = 16.5 Hz), 116.2 (d, *J* = 20.8 Hz), 102.1 (d, *J* = 3.7 Hz), 64.7 (d, *J* = 2.3 Hz), 61.4, 25.4, 13.7 (d, *J* = 3.7 Hz)

^{19}F NMR (376 MHz, $CDCl_3$) δ = -121.8 (d, *J* = 7.3 Hz)

FT-IR 3031, 2981, 2920, 1654, 1630, 1481, 1252, 1039, 1027, 980, 948, 801, 774, 672cm^{-1}

HRMS (ESI) m/z: $[M + H]^+$ Calculated for $C_{18}H_{21}FNO^+$ 286.1607 , Found: 268.1597

Melting Point 83 - 87 ^oC

Compound 3kc

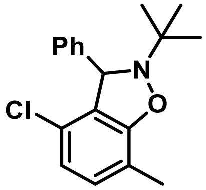

Compound **3kc** was prepared according to GP(C) from **1k** (0.2954g, 0.5mmol, 1 equiv.) and **2c** (0.0886g, 0.5mmol, 1 equiv.). **3kc** was obtained in 59% yield (0.0890 g, 0.30 mmol) as a white crystalline solid.

R_f = 0.44 in 6% EtOAc:Hexanes

^1H NMR (400 MHz, $CDCl_3$) δ = 7.30-7.23 (m overlaps with solvent residual signal, 5H), 6.92 (d, *J* = 8.0 Hz, 1H), 6.72 (d, *J* = 8.0 Hz, 1H), 5.60 (s, 1H), 2.24 (s, 3H), 1.17 (s, 9H)

$^{13}C\{^1H\}$ NMR (101 MHz, $CDCl_3$) δ = 156.8, 141.8, 131.2, 128.5, 128.0, 127.6, 126.5, 126.3, 121.3, 116.0, 67.0, 61.6, 25.3, 15.0

FT-IR 3031, 2972, 2937, 1591, 1473, 1453, 1409, 1364, 1203, 1133, 1052, 988, 851, 692, 571 cm^{-1}

HRMS (ESI) m/z: $[M + H]^+$ Calculated for $C_{18}H_{21}ClNO^+$ 302.1306, Found: 302.1289

Melting point 73 - 76 ^oC

Compound 3lc

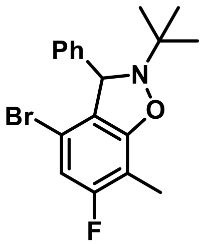

Compound **3lc** was prepared according to GP(C) from **1l** (0.3266g, 0.5mmol, 1 equiv.) and **2c** (0.0886g, 0.5mmol, 1 equiv.). **3lc** was obtained in 56% yield (0.1020g, 0.28mmol) as 1:10 mixture of regioisomers appearing as a white crystalline solid after chromatography. Characterization for the major regioisomer is described below.

R_f = 0.39 in 6% EtOAc:Hexanes

^{1}H NMR (400 MHz, $CDCl_3$) δ = 7.39-7.23 (m overlaps with solvent residual signal, 5H), 6.81 (d, *J* = 9.2 Hz, 1H), 5.53 (s, 1H), 2.23 (s, 3H), 1.17 (s, 9H)

^{13}C{^{1}H} NMR (101 MHz, $CDCl_3$) δ = 153.9 (d, *J* = 238.9),152.4 (d, *J* = 1.7 Hz), 141.2, 129.2, 128.3(3) (d, *J* = 42.57 Hz), 128.3(1) (d, *J* = 76.7 Hz), 127.7, 117.3(4) (d, *J* = 24.6 Hz), 117.3(0), 101.2 (d, *J* = 24.9 Hz), 68.3 (d *J* = 1.9 Hz), 61.6, 25.3, 15.2

^{19}F{^{1}H} NMR (376 MHz, $CDCl_3$) δ = -121.26

FT-IR 3035, 2972, 2865, 1594, 1467, 1390, 1363, 1198, 1015, 876, 707, 691 cm^{-1}

HRMS (ESI) m/z: $[M + H]^+$ Calculated for $C_{18}H_{20}BrFNO^+$ 364.0707, Found: 364.0699:

Melting point 97 - 99 ^oC

Compound 3bf

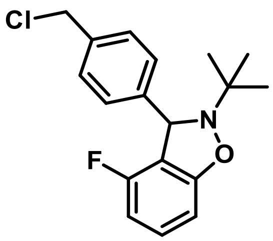

Compound **3bf** was prepared according to GP(C) from **1b** (0.2802g, 0.5mmol, 1 equiv.) and **2f** (0.3836g, 1.5mmol, 3 equiv.). **3bf** was obtained in 77% yield (0.1231g, 0.39mmol) as a white crystalline solid.

R_f = 0.73 in 20% EtOAc:Hexanes

^{1}H NMR (400 MHz, $CDCl_3$) δ = 7.42 (d, *J* = 8.0 Hz, 2H), 7.35 (d, *J* = 8.1 Hz, 2H), 7.17-7.10 (m, 1H), 6.61 (d, *J* = 8.1 Hz, 1H), 6.52 (t, *J* = 8.6 Hz, 1H), 5.73 (s, 1H), 4.56 (s, 2H), 1.17 (s, 9H)

^{13}C{^{1}H} NMR (101 MHz, $CDCl_3$) δ = 159.3 (d, *J* = 11.9 Hz), 158.5 (d, *J* = 252.5 Hz), 142.9, 137.1, 131.1 (d, *J* = 8.6 Hz), 129.3, 128.1, 116.5 (d, *J* = 20.0 Hz), 108.3 (d, *J* = 20.4 Hz), 103.3 (d, *J* = 3.5 Hz), 64.5 (d, *J* = 2.6 Hz), 61.9, 46.4, 25.8

^{19}F{^{1}H} NMR (376 MHz, $CDCl_3$) δ = -117.7

FT-IR 2974, 1621, 1604, 1483, 1365, 1268, 1236, 1203, 1021, 985, 830, 801, 776, 672 cm^{-1}

HRMS (ESI) m/z: $[M + H]^+$ Calculated for $C_{18}H_{20}ClFNO^+$ 320.1212, found 320.1189

Melting point 100 - 105 ^oC

Compound 3bg

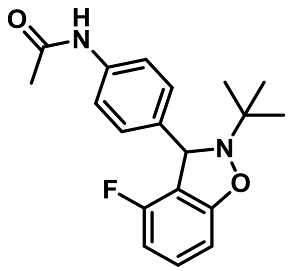

Compound **3bg** was prepared according to GP(C) from **1b** (0.2802g, 0.5mmol, 1 equiv.) and **2g** (0.3514g, 1.5mmol, 3 equiv.). **3bg** was obtained in 64% yield (0.1051g, 0.32mmol) as a white crystalline solid.

R_f = 0.14 in 40% EtOAc in Hexanes

^{1}H NMR (400 MHz, $CDCl_3$) δ = 7.45 (d, *J* = 8.5 Hz, 2H), 7.35 (d, overlaps with NH, *J* = 8.2 Hz, 3H), 7.15-7.10 (m, 1H), 6.60 (d, *J* = 8.0 Hz, 1H), 6.51 (t, *J* = 8.6 Hz 1H), 5.69 (s, 1H), 2.14 (s, 3H), 1.17 (s, 9H)

^{13}C{^{1}H} NMR (101 MHz, $CDCl_3$) δ = 168.8, 159.7 (d, *J* = 3.6 Hz), 158.5 (d, *J* = 244.2 Hz), 138.6, 137.7, 131.0, (d, *J* = 8.4 Hz), 128.4, 120.5, 116.6 (d, *J* = 20.0 Hz), 108.3 (d, *J* = 20.1 Hz), 103.2 (d, *J* = 3.4 Hz), 64.5, (d, *J* = 2.1 Hz), 61.9, 25.8, 25.0

^{19}F{^{1}H} NMR (376 MHz, $CDCl_3$) δ = -117.79

FT-IR 3309, 3124, 2976, 1664, 1601, 1532, 1458, 1411, 1366, 1316, 1278, 1237,1205, 1025, 823, 769 cm^{-1}

HRMS (ESI) m/z: $[M + H]^+$ Calculated for $C_{19}H_{22}FN_2O_2^+$ 329.1660, Found: 329.1653

Melting point 142 - 151 ^oC

Compound 3bh

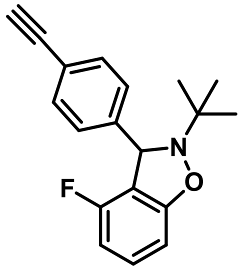

Compound **3bh** was prepared according to GP(C) from **1b** (0.2802g, 0.5mmol, 1 equiv.) and **2h** (0.3019g, 1.5mmol, 3 equiv.). **3bh** was obtained in 71% yield (0.1049g, 0.35mmol) as a white crystalline solid.

R_f = 0.58 in 10% EtOAc:Hexanes

^{1}H NMR (400 MHz, $CDCl_3$) δ = 7.46 (d, *J* = 8.2 Hz, 2H), 7.39 (d, *J* = 8.2 Hz, 2H), 7.17-7.11 (m, 1H), 6.6 (d, *J* = 8.1Hz, 1H), 6.52 (t, *J* = 8.6 Hz, 1H), 5.72 (s, 1H), 3.04 (s, 1H), 1.17 (s, 9H)

$^{13}C\{^1H\}$ NMR (101 MHz, $CDCl_3$) δ = 159.3, 158.0 (d, *J* = 242.1 Hz), 142.9, 132.4, 130.7 (d, *J* = 8.4Hz), 127.3, 121.3, 115.8 (d, *J* = 20.4 Hz), 107.9 (d, *J* = 20.0 Hz), 102.9 (d, *J* = 3.5 Hz), 85.5, 77.2, 64.2 (d, *J* = 2.6 Hz), 61.5, 25.3,

$^{19}F\{^1H\}$ NMR (376 MHz, $CDCl_3$) δ = -117.7

FT-IR 3284, 2981, 2360, 1619, 1605, 1485, 1457, 1364, 1236, 1212, 1069, 1025, 856, 823, 775, 650, 609 cm^{-1}

HRMS (ESI) m/z: $[M + H]^+$ Calculated for $C_{19}H_{19}FNO^+$ 296.1445, Found: 296.1430

Melting point 60 - 63 ^oC

Compound 3bi

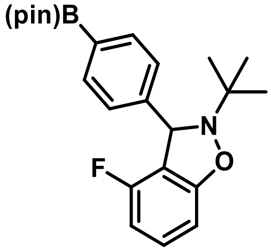

Compound **3bi** was prepared according to GP(C) from **3b** (0.2802g, 0.5mmol, 1 equiv.) and **2i** (0.4759g, 1.5mmol, 3 equiv.). **3bi** was obtained in 47% yield (0.0914g, 0.24mmol) as a white crystalline solid. This compound suffers loss in yield on isolation due to affinity for stationary phase. The assay yield for this reaction is 83%.

R_f = 0.43 in 10% EtOAc:Hexanes

^1H NMR (400 MHz, $CDCl_3$) δ = 7.79 (d, *J* = 7.9 Hz, 2H), 7.44 (d, *J* = 7.8 Hz, 2H), 7.14-7.10 (m, 1H), 6.60 (d, *J* = 8.1Hz, 1H), 6.49 (t, *J* = 8.5 Hz, 1H), 5.73 (s, 1H), 1.32 (s, 12H), 1.17 (s, 9H)

$^{13}C\{^1H\}$ NMR (101 MHz, $CDCl_3$) δ = 159.1 (d, J = 7.5 Hz), 158.1 (d, *J* = 248.3 Hz), 145.3, 135.1, 130.5 (d, *J* = 8.6 Hz), 126.7, 116.2 (d, *J* = 20.2 Hz), 107.8 (d, *J* = 20.2 Hz), 102.8 (d, *J* = 3.5 Hz), 83.7, 64.7 (d, *J* = 2.3 Hz), 61.4, 25.3, 24.9
Note-The C-B carbon is not observed due to strong quadrupolar relaxation.

$^{19}F\{^1H\}$ NMR (376 MHz, $CDCl_3$) δ = -117.82

FT-IR 2972, 1624, 1609, 1460, 1356, 1275, 1208, 1137, 1086, 1025, 859, 735, 655 cm^{-1}

HRMS (ESI) m/z: $[M + H]^+$ Calculated for $C_{23}H_{30}BFNO_3^+$ 398.2297, Found: 398.2285

Melting point 112 - 118 ^oC

Compound 3bj

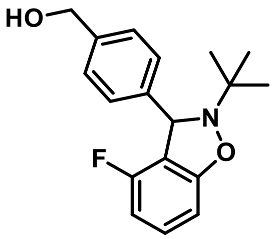

Compound **3bj** was prepared according to GP(C) from **1b** (0.2802g, 0.5mmol, 1 equiv.) and **2j** (0.3109g, 1.5mmol, 3 equiv.). **3bj** was obtained in 48% yield (0.7233g, 0.24mmol) as a white crystalline solid. This reaction gives an assay yield of 63%.

R_f = 0.19 in 20% EtOAc:Hexanes

^{1}H NMR (400 MHz, $CDCl_3$) δ = 7.41 (d, *J* = 8.0 Hz, 2H), 7.33 (d, *J* = 7.3 Hz, 2H), 7.16-7.11 (m, 1H), 6.62 (d, *J* = 8.1 Hz, 1H), 6.52 (t, *J* = 8.6 Hz, 1H), 5.73 (s, 1H), 4.67 (s, 2H), 1.18 (s, 9H)

^{13}C{^{1}H} NMR (101 MHz, $CDCl_3$) δ = 159.4, 158.1 (d, *J* = 248.2 Hz), 141.8, 140.2, 130.6 (d, *J* = 8.4 Hz), 127.5, 127.3, 116.2 (d, *J* = 20.1 Hz), 107.8 (d, *J* = 20.2 Hz), 102.8 (d, *J* = 3.6 Hz), 65.6, 64.3 (d, *J* = 2.3 Hz), 61.5, 25.3

^{19}F{^{1}H} NMR (376 MHz, $CDCl_3$) δ = -117.9

FT-IR 3291, 2975, 1622, 1606, 1515, 1486, 1369,1280, 1262, 1200, 1045, 1021, 972, 700, 636, 600 cm^{-1}

HRMS (ESI) m/z: $[M + H]^+$ Calculated for $C_{18}H_{21}FNO_2^+$ 302.1551, found 302.1523

Melting point 95 - 101 ^oC

Compound 3mc

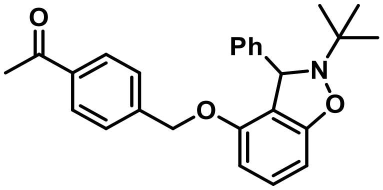

Compound **3mc** was prepared according to GP(C) from **1m** (0.3453g, 0.5mmol, 1 equiv.) and **2c** (0.0886g, 0.5mmol, 1 equiv.). **3mc** was obtained in 77% yield (0.1546g, 0.39mmol) as a white crystalline solid.

R_f = 0.35 in 20% EtOAc:Hexanes

^{1}H NMR (400 MHz, $CDCl_3$) δ = 7.83 (d, *J* = 8.2 Hz, 2H), 7.37-7.28 (m, 5H), 7.11 (t, *J* = 8.1 Hz, 1H), 7.03 (d, *J* = 8.1 Hz, 2H), 6.50 (d, *J* = 8.1 Hz, 1H), 6.34 (d, *J* = 8.1 Hz, 1H) 5.64 (s, 1H), 5.00 (d, *J* = 12.8 Hz, 1H), 4.93 (d, *J* = 12.7 Hz, 1H), 2.59 (s, 3H), 1.18 (s, 9H)

^{13}C{^{1}H} NMR (101 MHz, $CDCl_3$) δ = 198.2, 159.1, 154.5, 142.6, 142.5, 136.9, 130.7, 128.9, 128.8, 128.4, 127.7, 127.4, 117.4, 104.6, 100.9, 69.4, 66.0, 61.9, 27.1, 25.8

FT-IR 2977, 2929, 2854, 1682, 1604, 1491, 1451, 1364, 1288, 1267, 1250, 120, 852, 1050, 960, 821, 697 cm^{-1}

HRMS (ESI) m/z: $[M + Na]^+$ Calculated for $C_{26}H_{27}NNaO_3^+$ 424.1889, Found: 424.1867

Melting point 129 - 134 ^oC

Compound 3nc

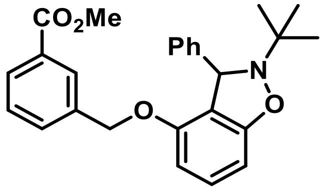

Compound **3nc** was prepared according to GP(C) from **1n** (0.3548g, 0.5mmol, 1 equiv.) and **2c** (0.0886g, 0.5mmol, 1 equiv.). Compound **3nc** was obtained in 68% yield (0.1418g, 0.34 mmol) as a white crystalline solid.

R_f = 0.3 in 10% EtOAc:Hexanes

^{1}H NMR (400 MHz, $CDCl_3$) δ = 7.94 (d, *J* = 7.7 Hz, 1H), 7.90 (s, 1H), 7.37-7.21 (m, overlaps with solvent residual signal, 6H), 7.11 (t, *J* = 8.1 Hz, 1H), 7.06 (br d, *J* = 7.7 Hz, 1H), 6.50 (d, *J* = 8.0Hz, 1H), 6.38 (d, *J* = 8.0 Hz, 1H), 5.64 (s, 1H), 4.98 (d, *J* = 12 Hz, 1H), 4.89 (d, *J* = 12 Hz, 1H), 3.93 (s, 3H), 1.17 (s, 9H)

^{13}C{^{1}H} NMR (101 MHz, $CDCl_3$) δ = 166.8, 158.6, 154.2, 143.2, 137.1, 131.8, 130.2, 129.0, 128.5, 128.3, 128.2, 127.8, 127.1, 117.1, 104.3, 100.3, 69.2, 65.9, 65.4, 52.2, 25.3

*1 carbon signal missing due to peak overlap

FT-IR 3027**,** 2980, 1724, 1603, 1447,1390, 1284,1198, 1098, 1081, 774, 744, 717, 702, 596

HRMS (ESI) m/z: $[M + H]^+$ Calculated for $C_{26}H_{28}NO_4^+$ 418.2013, Found: 418.2004

Melting point 129 - 134 ^oC

Compound 3mf

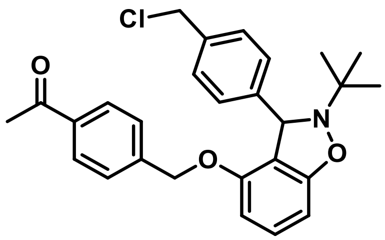

Compound **3mf** was prepared according to GP(C) from **1m** (0.3453g, 0.5mmol, 1 equiv.) and **2f** (0.3109g, 1.5mmol, 3 equiv.). **3mf** was obtained in 63% yield (0.1417g, 0.32 mmol) as a white crystalline solid.

R_f = 0.31 in 20% EtOAc:Hexanes

^{1}H NMR (400 MHz, $CDCl_3$) δ = 7.86 (d, *J* = 8.2 Hz, 2H), 7.37-7.32 (m, 4H), 7.16 (t, *J* = 8.1 Hz, 1H), 7.03 (d, *J* = 8.1 Hz, 2H), 6.49 (d, *J* = 8.1 Hz, 1H), 6.34 (d, *J* = 8.2 Hz, 1H), 5.65 (s, 1H), 5.00 (d, *J* = 13.1 Hz, 1H), 4.94 (d, *J* = 12.7 Hz, 1H), 4.60 (s, 2H), 2.59 (s, 3H), 1.17 (s, 9H)

^{13}C{^{1}H} NMR (101 MHz, $CDCl_3$) δ = 197.8, 158.6, 154.0, 143.6, 141.9, 136.6, 136.5, 130.4, 128.7, 128.5, 128.3, 127.0, 116.7, 104.2, 100.4, 69.0, 65.1, 61.4, 46.2, 26.7, 25.3

FT-IR 2972, 2922, 2864, 1674, 1609, 1492, 1390, 1377, 1292, 1267, 1241, 1095, 1050, 1032, 1015, 731, 717 cm^{-1}

HRMS (ESI) m/z: $[M + H]^+$ Calculated for $C_{27}H_{29}ClNO_3^+$ 450.1836, found 450.1830

Melting point decomposition 190 ^oC

Compound 3bl

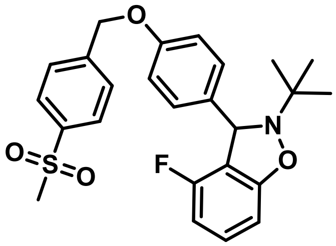

Compound **3bl** was prepared according to GP(C) from **1b** (0.1440g, 0.25mmol, 1 equiv.) and **2l** (0.1130g, 0.31mmol, 1.25 equiv.) and was obtained in 50% yield (0.0570g, 0.13 mmol) as a viscous oil.

R_f = 0.20 in 30% EtOAc:Hexanes

^{1}H NMR (400 MHz, $CDCl_3$) δ = 7.95 (d, *J* = 8.2 Hz, 2H), 7.62 (d, *J* = 8.2 Hz, 2H), 7.32 (d, *J* = 8.5 Hz, 2H), 7.16-7.11 (m, 1H), 6.91 (d, *J* = 8.6 Hz, 2H), 6.61 (d, *J* = 8.1 Hz, 1H), 6.52 (t, *J* = 8.5 Hz, 1H), 5.69 (s, 1H), 5.13 (s, 2H), 3.05 (s, 3H), 1.17 (s, (H)

^{13}C{^{1}H} NMR (101 MHz, $CDCl_3$) δ = 159.4 (d, *J* = 17.4 Hz), 158.1 (d, *J* = 257.6 Hz), 157.6, 143.5, 139.9, 135.4, 130.5 (d, *J* = 8.6 Hz), 128.6, 127.8, 127.7, 116.3 (d, *J* = 20.3 Hz), 114.8, 107.8 (d, *J* = 20.3 Hz), 102.8 (d, *J* = 3.5 Hz), 68.8, 63.9 (d, *J* = 2.6 Hz), 61.4, 44.6, 25.3

^{19}F{^{1}H} NMR (376 MHz, $CDCl_3$) δ = -117.97

FT-IR cm^{-1} 2973, 2928, 1623, 1607, 1508, 1458, 1303, 1235, 1146,1023, 954, 823, 758, 517

HRMS (ESI) m/z: $[M + H]^+$ Calculated for $C_{25}H_{27}FNO_4S^+$, 456.1645 found 456.1636

Compound 3bk

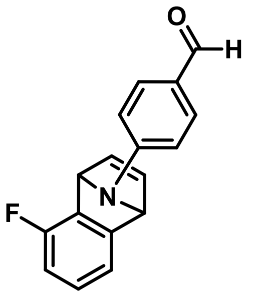

Compound **3bk** was prepared according to GP(C) from **1b** (0.2880g, 0.5mmol, 1 equiv.) and **2k** (0g, 1.5mmol, 3 equiv.) with the following modification: the reaction was run at room temperature for 24 hours. **3bk** was obtained in 54% yield (0.0716g, 0.27 mmol) as a yellow oil.

R_f = 0.27 in 20% EtOAc:Hexanes

^{1}H NMR (400 MHz, $CDCl_3$) δ = 9.76 (s, 1H), 7.68 (d, *J* = 8.6 Hz, 2H), 7.07 (d, *J* = 6.9 Hz, 1H), 7.02 (br, 2H), 6.94-6.88 (m, 3H), 6.65 (t, *J* = 8.3 Hz, 1H), 5.80 (s, 1H), 5.57 (s, 1H)

^{13}C{^{1}H} NMR (101 MHz, $CDCl_3$) δ = 190.7, 157.1 (d, *J* = 246.6 Hz), 151.6 (d, *J* = 4.5 Hz), 151.5, 142.4, 141.7, 132.5 (d, *J* = 20.6 Hz), 131.4, 129.6, 127.6 (d, *J* = 6.1 Hz), 117.7 (d, *J* = 2.8 Hz), 116.9, 113.8 (d, *J* = 22.0 Hz), 68.7 (d, *J* = 0.90 Hz), 64.9

^{19}F{^{1}H} NMR (376 MHz, $CDCl_3$) δ = -120.98

FT-IR 2807, 2735, 2360, 1666, 1592, 1508, 1467, 1311, 1217, 1163, 907, 761, 723, 699 cm^{-1}

HRMS (ESI) m/z: [M + H]$^+$ Calculated for $C_{17}H_{13}FNO^+$ 266.0976, found 266.0970

Limitations of the K_3PO_4 Aryl(TMP)iodonium tosylate system

>20% Yield with 2c

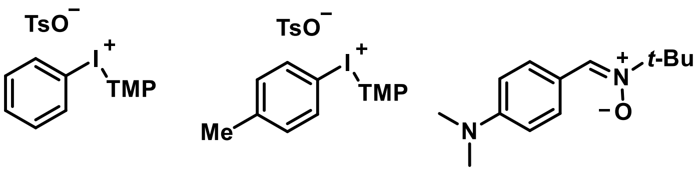

Complex mixture with 1b

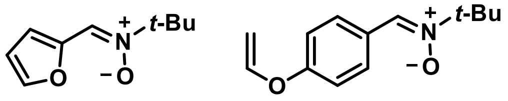

Deuterium Incorporation Experiment

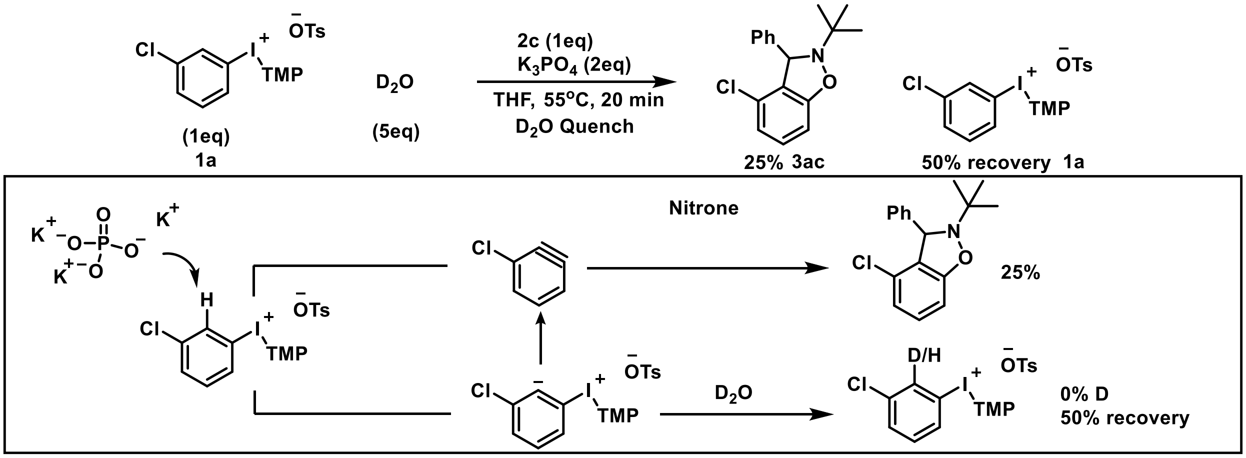

1a (0.5760g, 1mmol) was added to a 50ml round bottom flash followed by **2c** (0.1770g, 1mmol), deuterium oxide (90uL, 5mmol, 5 equiv.) and THF (5ml, 0.2M). The flask was submerged in an oil bath preheated to 55°C and potassium phosphate (0.4240g, 2mmol, 2 equiv.) was added in one portion with vigorous stirring. After 20 minutes, the reaction was quenched with deuterium oxide. The organic layer was removed, and the aqueous layer extracted with DCM (3x15ml). The starting material was triturated from DCM with diethyl ether. The precipitate was collected by vacuum filtration to give 50% (0.2880g, 0.5mmol) of recovered starting material. The filtrate was also analyzed to verify the presence of the trapped aryne product. An analogous reaction was run on 0.1mmol scale and the mixture was analyzed by ^{1}H-NMR showing an identical result.

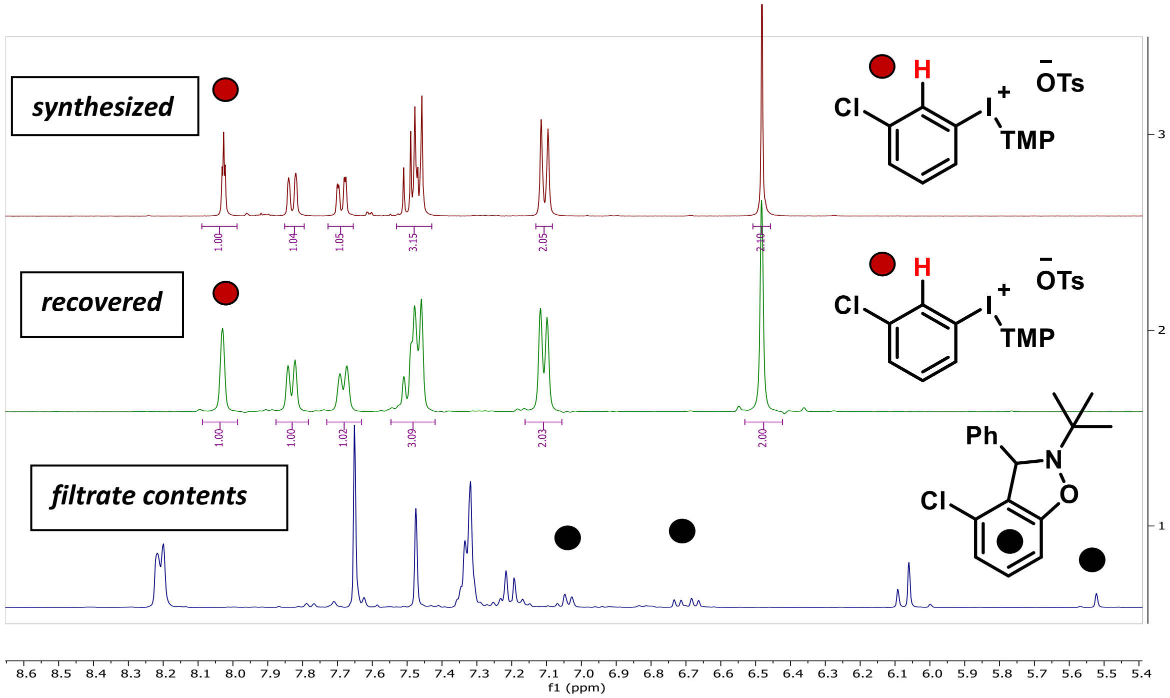

Experiments with halonium salts and literature conditions

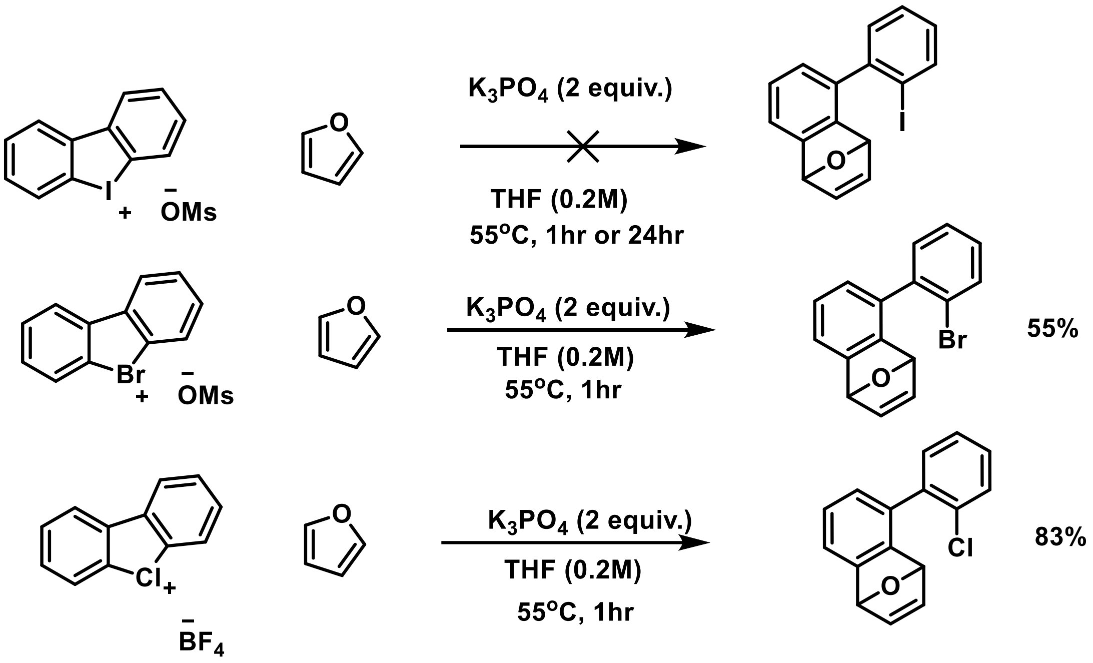

Acyclic λ^3-halonium salts failed to give any aryne adducts under our conditions

The reactions above were conducted according to GP(C) with the following modification, 5 equivalents of **2a** (0.0363ml, 0.5mmol) were used in place of **2c**, the reaction was conducted on 0.1mmol scale. The yields above represent assay yields using trimethoxybenzene as an internal standard.

Application of literature methods to acyclic iodonium salts[19]

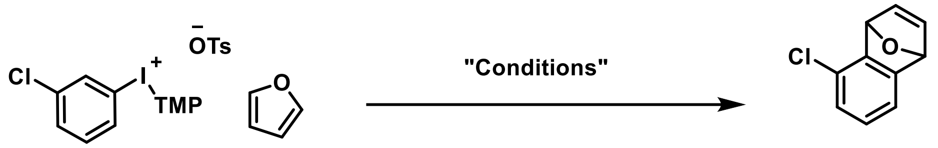

1a 2a 3aa

Conditions (ref 19)

Experiment	Solvent	Conc.	Base (eq)	2a (eq)	Temp	Time	Yield 3aa
1.	DCM	0.1M	Cs_2CO_3 (2eq)	1.2eq	R.T	16hr	11%
2.	MeCN	0.05M	K_3PO_4 (5eq)	5eq	R.T	16hr	25%
3.	DCM	0.04M	Cs_2CO_3 (4eq)	10eq	R.T	16hr	trace
4.	t-butanol	0.1M	Cs_2CO_3 (3eq)	1.5eq	120°C	4hr	34%

Reactions conducted on a 0.1mmol scale using tetrachloronitrobenzene as an internal standard.

Robustness Analysis

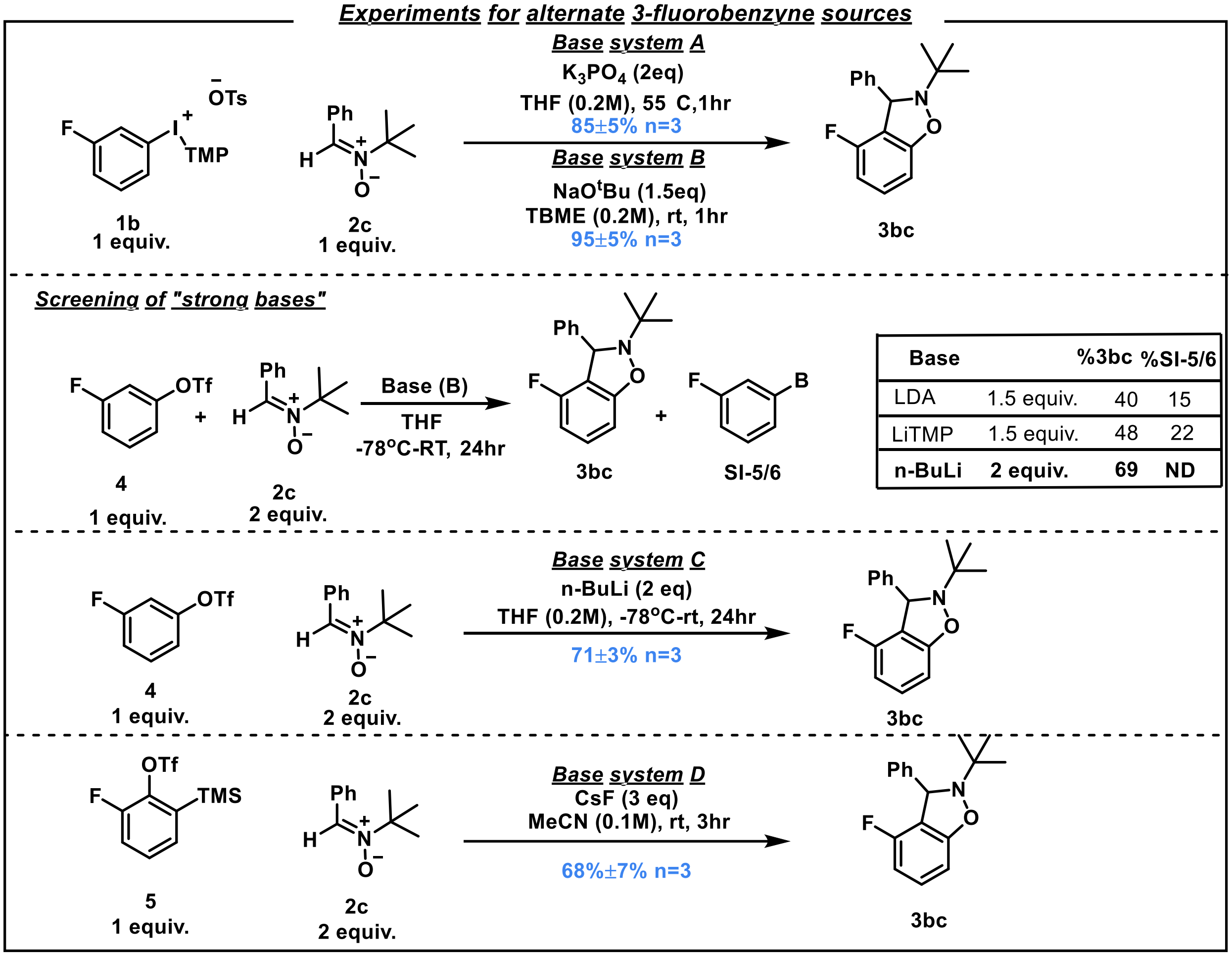

Base		%3bc	%SI-5/6
LDA	1.5 equiv.	40	15
LiTMP	1.5 equiv.	48	22
**n-BuLi**	**2 equiv.**	**69**	**ND**

Base system A general procedure:

1b (0.0560g, 0.1mmol, 1 equiv.) was weighed directly into a 3ml vial under ambient conditions. **2c** (0.0177g, 0.1mmol, 1 equiv.) was weighed and added to the vial followed by THF (0.5ml, 0.2M) and the additive (0.1mmol, 1 equiv.). The additive was weighed and added if solid or added via a micropipette if liquid. The vial was then equipped with a magnetic stir bar, sealed with a cap, and placed in an aluminum block preheated to 55°C with gentle stirring. Anhydrous potassium phosphate tribasic (0.0424g, 0.2mmol, 2 equiv.) was weighed out under ambient conditions and was quickly added to the vial in one portion. The vial was then resealed, stirring adjusted to maximize mixing and the reaction was allowed to proceed for 1 hour. Upon completion, the reaction was quenched with brine (2ml), tetrachloronitrobenzene (~0.1mmol) was added and the mixture was extracted with EtOAc (3x1ml). The organic layers were dried over magnesium sulfate and concentrated under reduced pressure. $CDCl_3$ (0.8ml) was used to fully solubilize the crude material and the ^{1}H-NMR spectrum was recorded with d1=30s

Base system B general procedure

1b (0.0560g, 0.1mmol, 1 equiv.) was weighed directly into a 3ml vial under ambient conditions. **2c** (0.0177g, 0.1mmol, 1 equiv.) was weighed and added to the vial followed by MTBE (0.5ml, 0.2M) and the additive (0.1mmol, 1 equiv.). The additive was weighed and added if solid or added via a micropipette if liquid. The vial was then equipped with a magnetic stir bar and NaO^{t}Bu (0.0144g, 0.15mmol, 1.5 equiv.) was weighed out under ambient conditions and was added to the vial in one portion with constant stirring. The vial was sealed, and the reaction was allowed to proceed for 1 hour at room temperature (ca. 25°C). Upon completion, the reaction was quenched with saturated ammonium chloride (2ml), tetrachloronitrobenzene (~0.1mmol) was added and the mixture was extracted with EtOAc (3x1ml). The organic layers were dried over magnesium sulfate and concentrated under reduced pressure. $CDCl_3$ (0.8ml) was used to fully solubilize the crude material and the ^{1}H-NMR spectrum was recorded with d1=30s

Base system C general procedure

3-fluorophenyl trifluoromethanesulfonate (**4**) (0.0244g, 0.1mmol, 1 equiv.), **2c** (0.0354g, 0.2mmol, 2 equiv.), and additive (0.1mmol, 1 equiv.) were added to an oven dried 3ml vial equipped with a magnetic stir bar sequentially, followed by 0.39ml of anhydrous THF. The vial was then purged with nitrogen gas and cooled to -78°C in a dry ice-acetone bath. n-BuLi (1.75M, 0.114ml, 0.2mmol, 2 equiv.) was added dropwise to the reaction with vigorous stirring under a nitrogen atmosphere. The dry Ice acetone bath was further insulated with aluminum foil and the reaction was allowed to continue for 24 hours slowly returning to room temperature. After completion, the reaction was quenched with saturated ammonium chloride (2ml), tetrachloronitrobenzene (~0.1mmol) was added and the mixture was extracted with EtOAc (3x1ml). The organic layers were dried over magnesium sulfate and concentrated under reduced pressure. $CDCl_3$ (0.8ml) was used to fully solubilize the crude material and the ^{1}H-NMR spectrum was recorded with d1=30s

Base system D general procedure

2-fluoro-6-(trimethylsilyl)phenyl trifluoromethanesulfonate (**5**) (0.0316g, 0.1mmol, 1 equiv.) **2c** (0.0354g, 0.2mmol, 2 equiv.), and additive (0.1mmol, 1 equiv.) was added to an oven dried 3ml vial equipped with a magnetic stir bar sequentially, followed by anhydrous MeCN (1ml, 0.1M). Cesium fluoride (0.045g, 0.3mmol. 3 equiv.) was weighed out under ambient conditions and was added to the vial quickly in one portion. The vial was sealed and allowed to stir intensely at room temperature for 3 hours. Upon completion, the reaction was diluted with EtOAc, tetrachloronitrobenzene (~0.1mmol) was added and the resulting mixture was filtered through a pipette containing a small pad a silica and celite, eluting first with EtOAc (3X1ml) then 5% methanol in DCM (3X1ml). The crude mixture was concentrated under reduced pressure, $CDCl_3$ (0.8ml) was used to fully solubilize the crude material and the ^{1}H-NMR spectrum was recorded with d1=30s

Additives used in this study

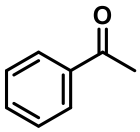 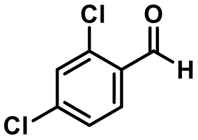 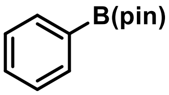 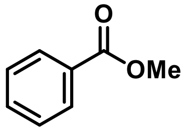

MFG	Acros Organics	Sigma Aldrich	Oakwood	Alfa Aesar
Lot #	A014073301	MKCG3287	010827121N	10214456
Purity	99%	99%	98%	99%
M.W=	120.15100	175.00800	204.07	136.15000

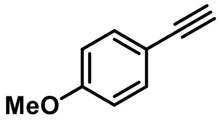 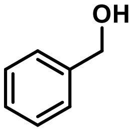 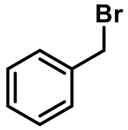 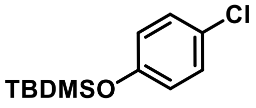

MFG	Alfa Aesar	TCI	Sigma Aldrich	Synthesized
Lot #	10193636	5S4KO-BJ	SHBC0722V	
Purity	99%	98%	98%	
M.W=	132.16200	171.19055	171.03700	242.81800

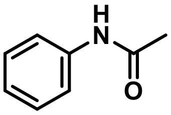

Synthesized (ref 20)
M.W= 135.16600

Internal standard used in this study

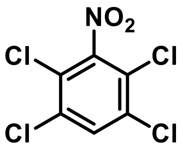

MFG	TCI
Lot #	Z7AKG-LY
Purity	99%
M.W=	260.87900

Overlays of ^{1}H-NMR Spectra for Robustness Screen

Additive: Acetophenone

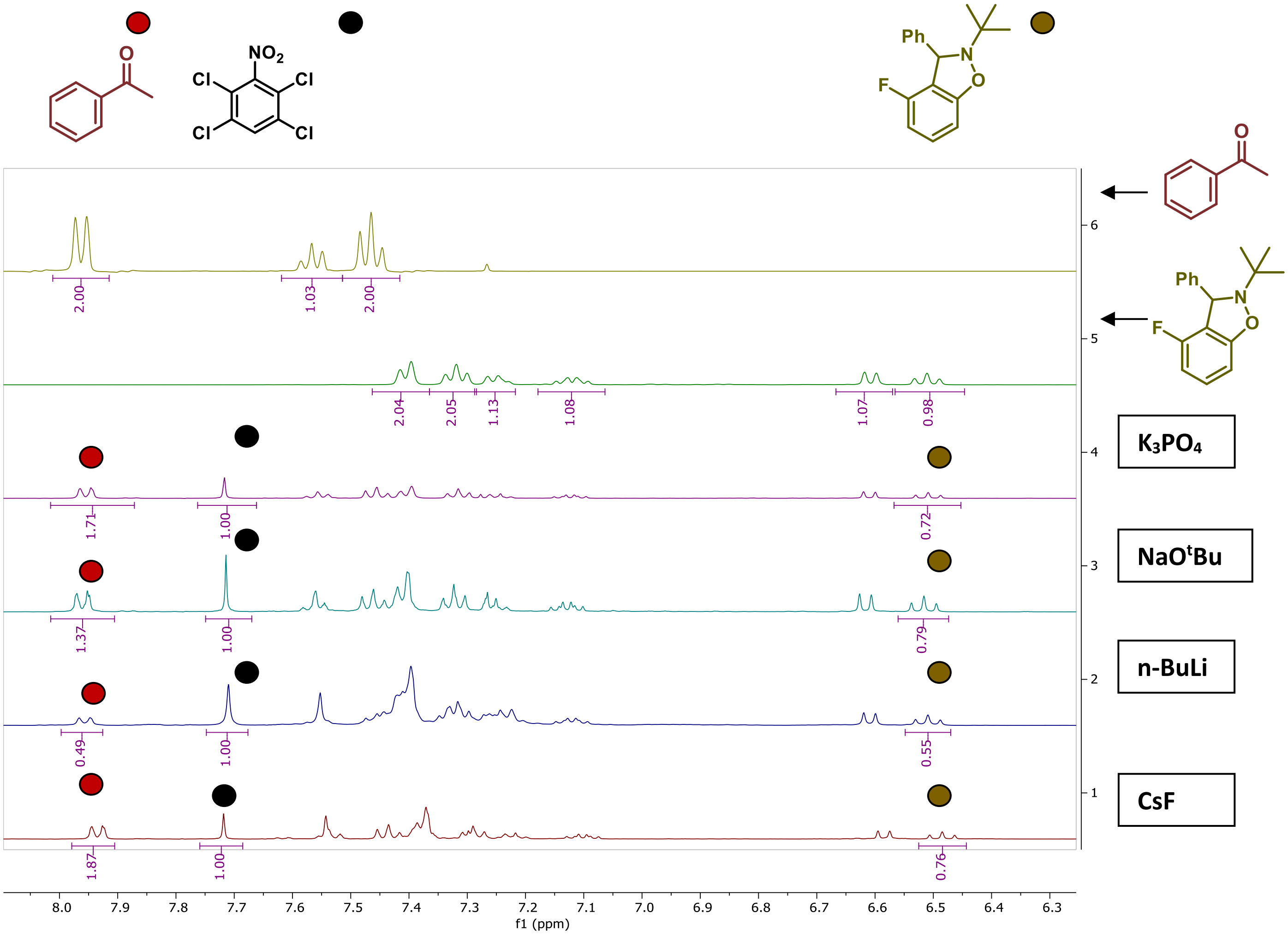

Base	Precursor Mass (mg)	Additive Mass (mg)	Mass TCNB (mg)	TCNB integral (1H)	Product Integral (1H)	Additive Integral (2H)	%Yield Adduct	%Additive Remaining
K_3PO_4	56.5	12.0	25.5	1	0.72	1.71	70	84
NaO^{t}Bu	56.2	12.0	21.6	1	0.775	1.37	64	57
n-BuLi	24.4	12.0	28.4	1	0.55	0.49	60	27
CsF	31.6	12.0	24.3	1	0.76	1.87	71	87

These reactions were run in triplicate and are represented in the manuscript as an average with standard deviation. The spectra above represent one of the triplicated experiments.

Additive: Acetanilide

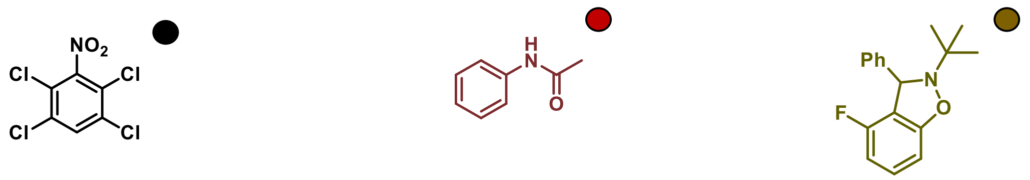

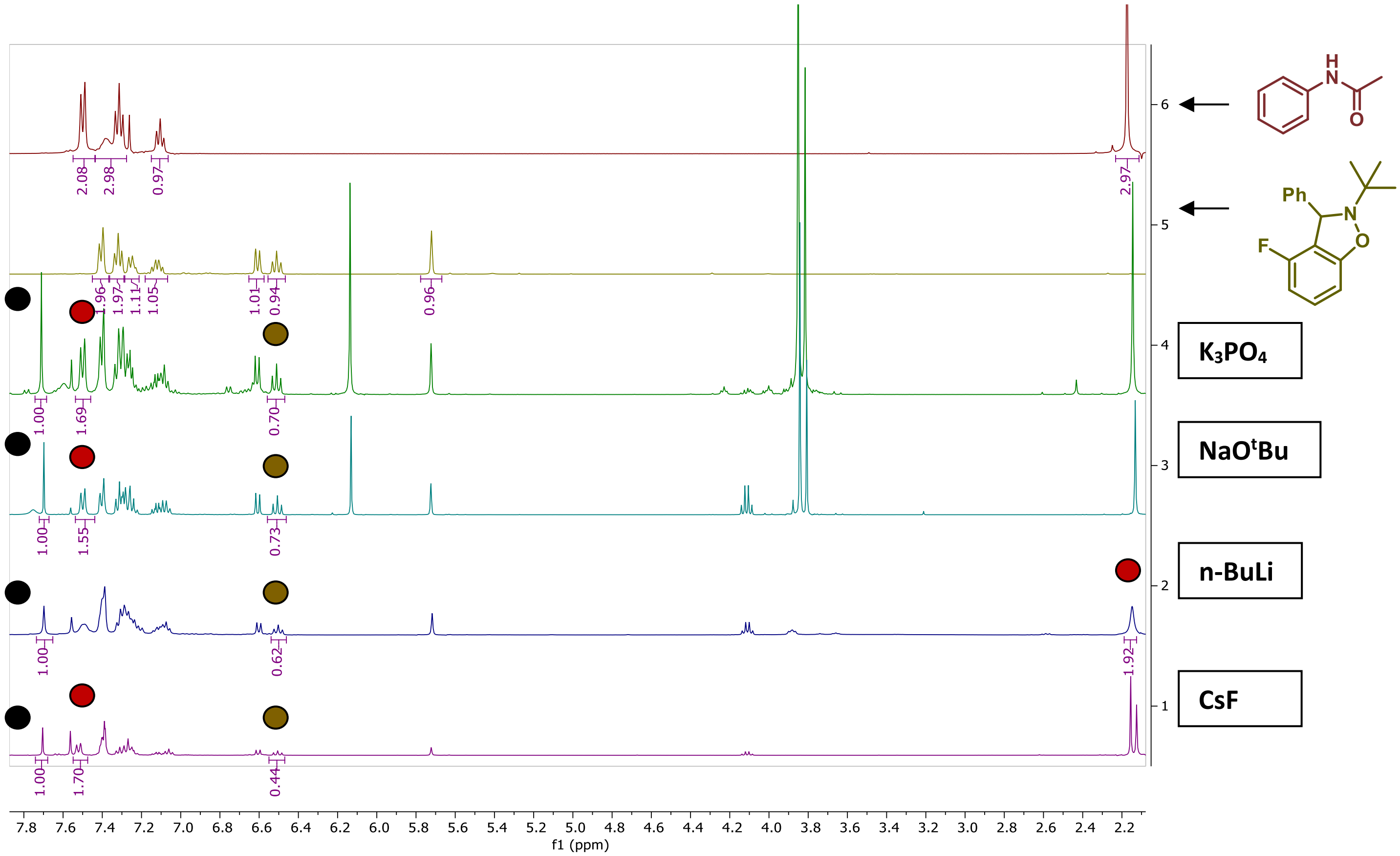

Base	Precursor Mass (mg)	Additive Mass (mg)	Mass TCNB(mg)	TCNB integral	Product Integral	Additive Integral	%Y Adduct	Additive Remaining
K_3PO_4	55.3	13.8	26.8	1	0.70	1.69(2H)	73	85
NaO^tBu	56.7	14	29.4	1	0.73	1.55(2H)	81	84
n-BuLi	24.4	13.7	24.4	1	0.62	1.92(3H)	58	59
CsF	31.6	13.5	29.9	1	0.44	1.70(2H)	50	97

Note* for the reaction with n-BuLi, due to broad and overlapping aromatic peaks, the alpha carbon hydrogens were used to calculate additive remaining. An additional spiking experiment was performed to verify the correct chemical shift to integrate.

<u>**Additive: 2,4-dichlorobenzaldehyde**</u>

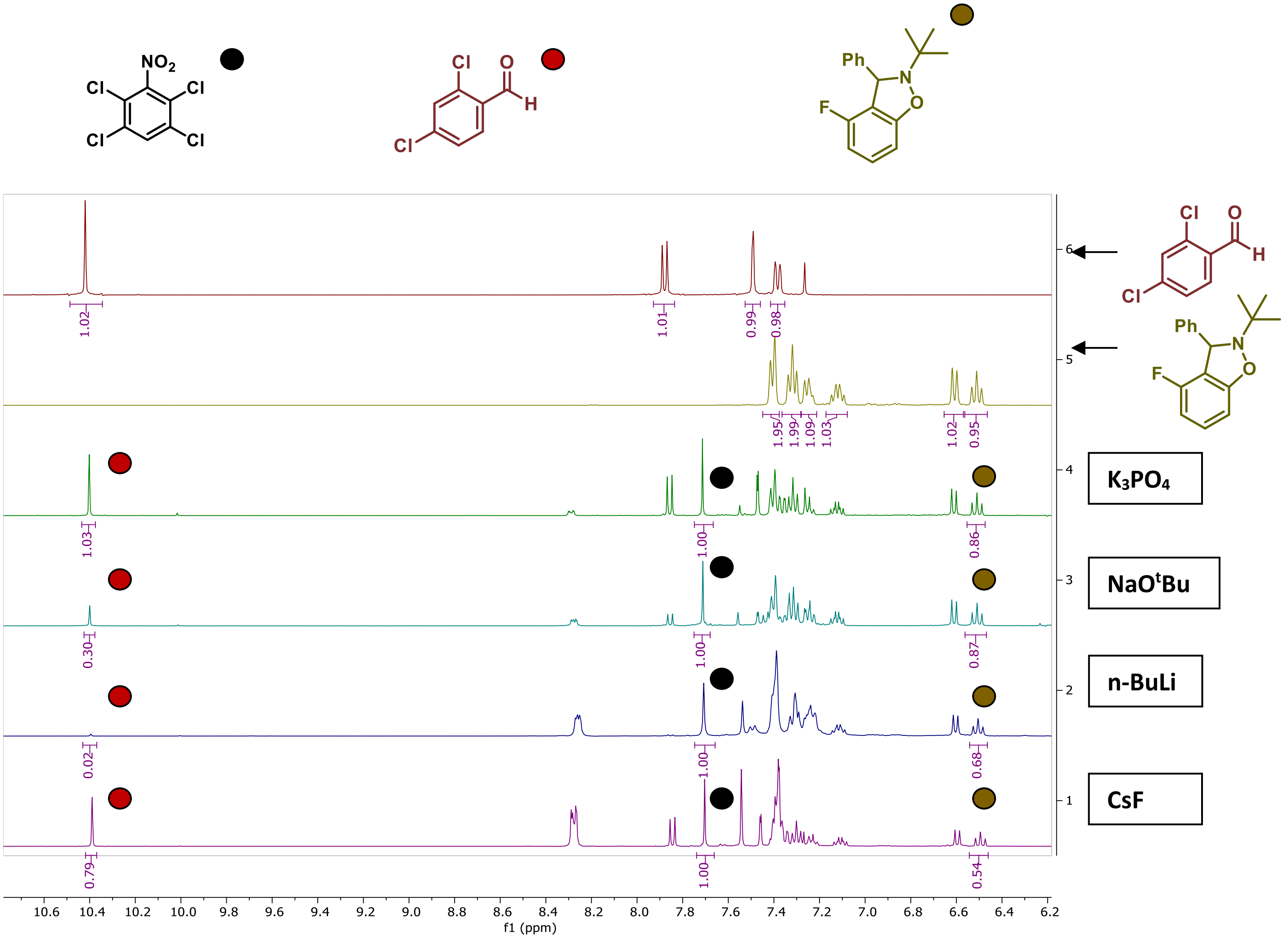

Base	Precursor Mass (mg)	Additive Mass (mg)	Mass TCNB(mg)	TCNB integral	Product Integral	Additive Integral (1H)	%Y Adduct	Additive Remaining
K_3PO_4	56.1	18.5	23.6	1	0.86	1.03	78	88
NaO^{t}Bu	56.4	17.6	21.4	1	0.87	0.3	71	24
n-BuLi	24.4	17.2	25.1	1	0.68	0.02	65	2
CsF	31.6	17.3	24.9	1	0.54	0.79	52	76

Additive: Phenyl-B(pin)

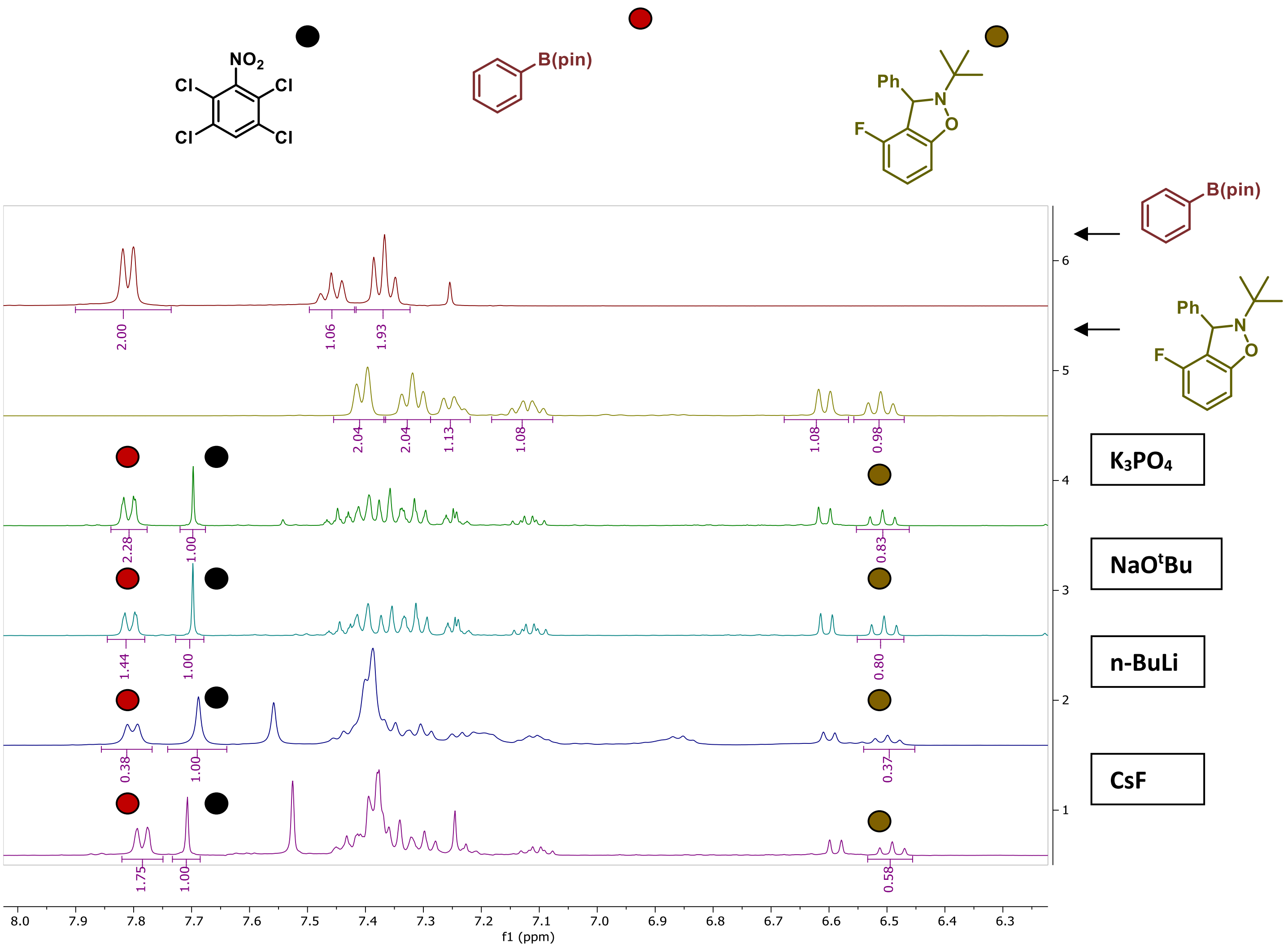

Base	Precursor Mass (mg)	Additive Mass (mg)	Mass TCNB(mg)	TCNB integral	Product Integral	Additive Integral (1H)	%Y Adduct	Additive Remaining
K_3PO_4	57.4	22.7	23.6	1	0.83	2.15	73	87
NaO^{t}Bu	56.8	20.8	25.8	1	0.8	1.44	78	70
n-BuLi	24.4	22.1	30.6	1	0.37	0.38	43	21
CsF	31.6	22.6	22.8	1	0.58	1.75	51	69

Additive: 4-ethynyl Anisole

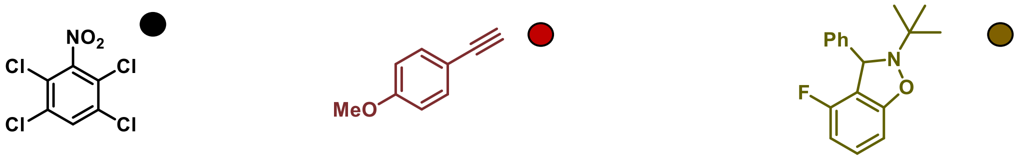

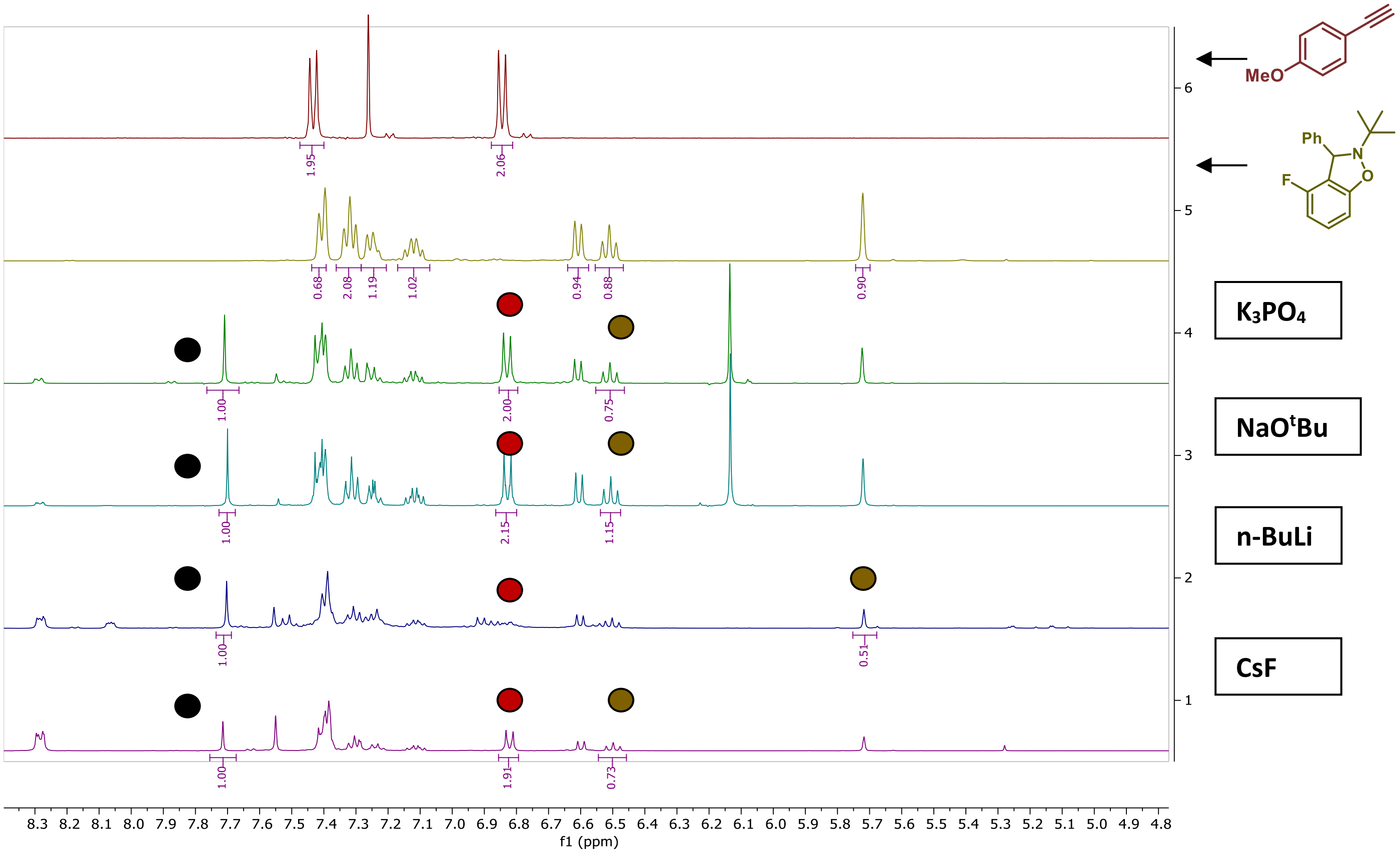

Base	Precursor Mass (mg)	Additive Mass (mg)	Mass TCNB(mg)	TCNB integral	Product Integral	Additive Integral (2H)	%Y Adduct	Additive Remaining
K_3PO_4	55.7	13.2	23.7	1	0.74	2.0	68	91
NaO^tBu	56.9	13.2	20.8	1	1.14	2.15	92	86
n-BuLi	24.4	13.2	25.0	1	0.55	N.D	49	0
CsF	31.6	13.2	21.2	1	0.72	1.91	59	78

Additive: Benzyl Bromide

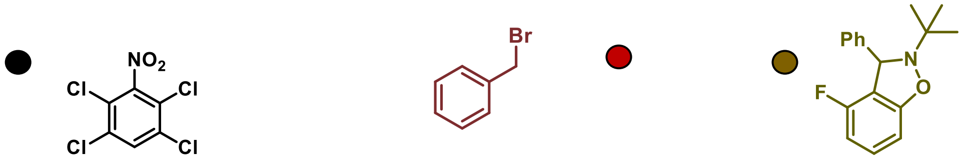

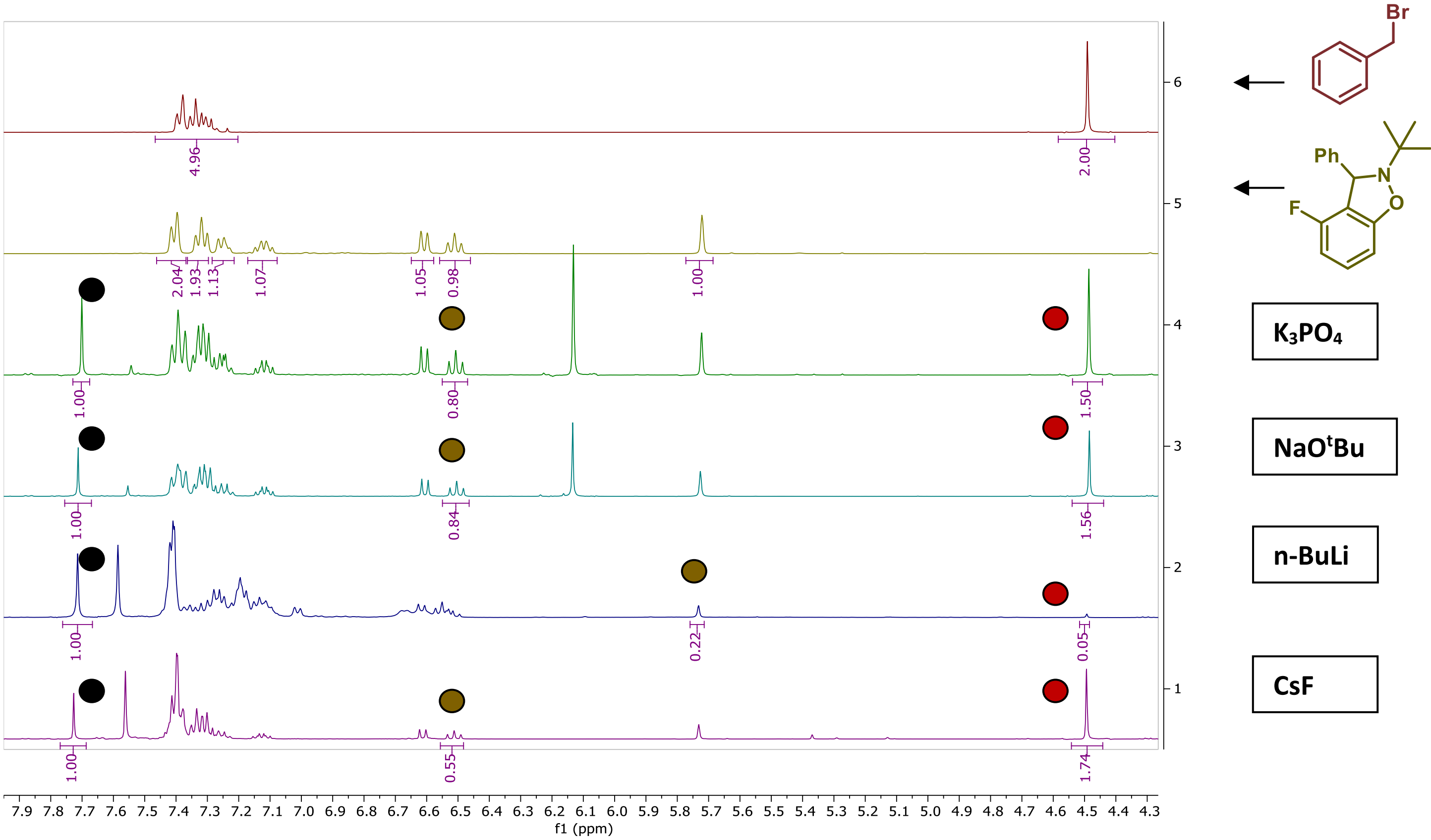

Base	Precursor Mass (mg)	Additive Mass (mg)	Mass TCNB(mg)	TCNB integral	Product Integral	Additive Integral (1H)	%Y Adduct	Additive Remaining
K_3PO_4	56.0	17.1	27.0	1	0.8	1.5	83	78
NaO^tBu	56.8	17.1	22.5	1	0.84	1.56	71	67
n-BuLi	24.4	17.1	23.2	1	0.22	0.05	20	2
CsF	31.6	17.1	22.2	1	0.55	1.74	47	74

Additive: OTBS-4-Cl-Ph

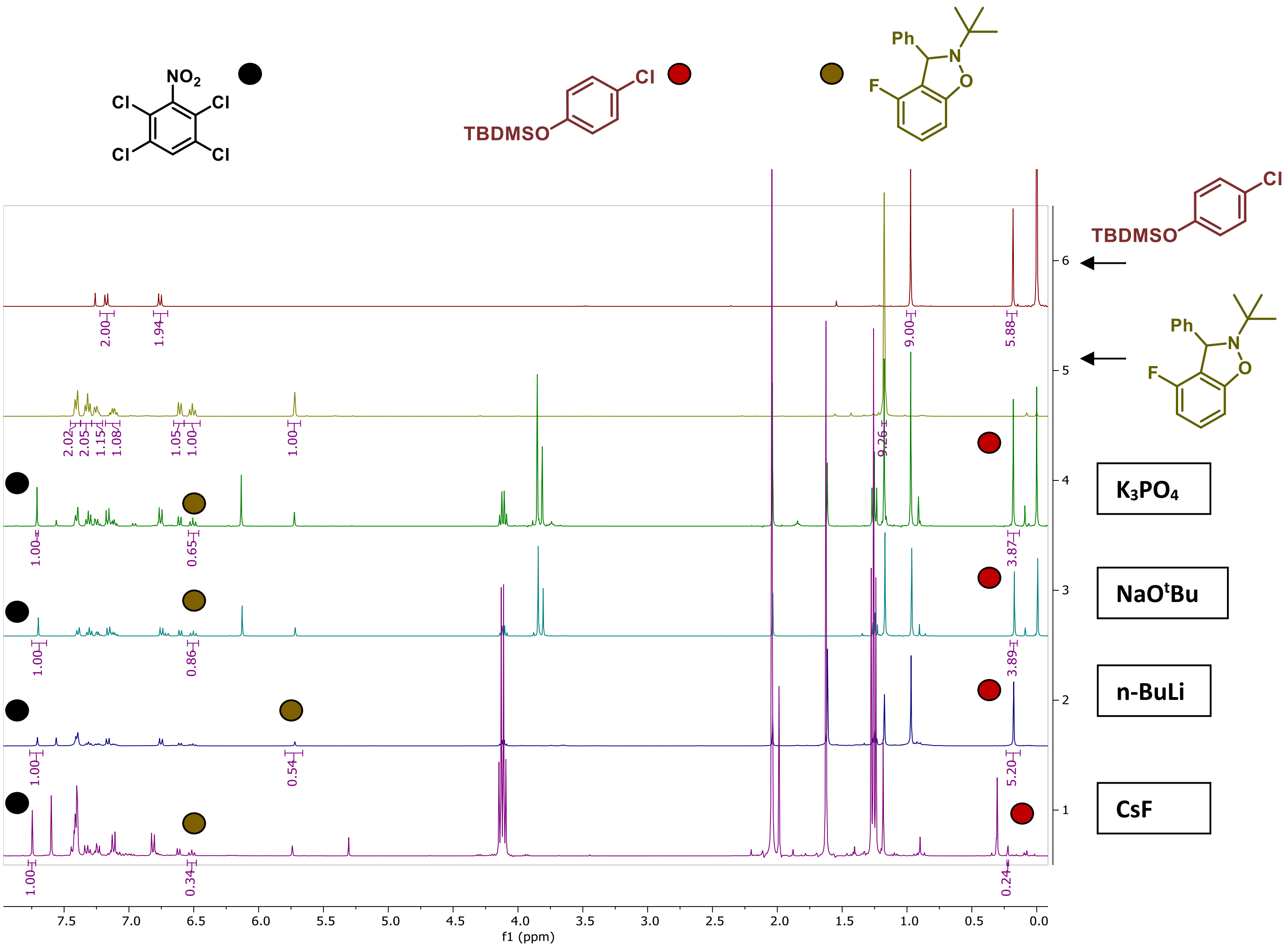

Base	Precursor Mass (mg)	Additive Mass (mg)	Mass TCNB(mg)	TCNB integral	Product Integral	Additive Integral (6H)	%Y Adduct	Additive Remaining
K_3PO_4	54.7	23.9	30.5	1	0.65	3.87	78	76
NaO^{t}Bu	56.8	24.2	26.0	1	0.86	3.89	85	65
n-BuLi	24.4	22.7	24.4	1	0.54	5.2	51	86
CsF	31.6	24.2	26.7	1	0.34	0.24	35	4

Additive: Benzyl alcohol

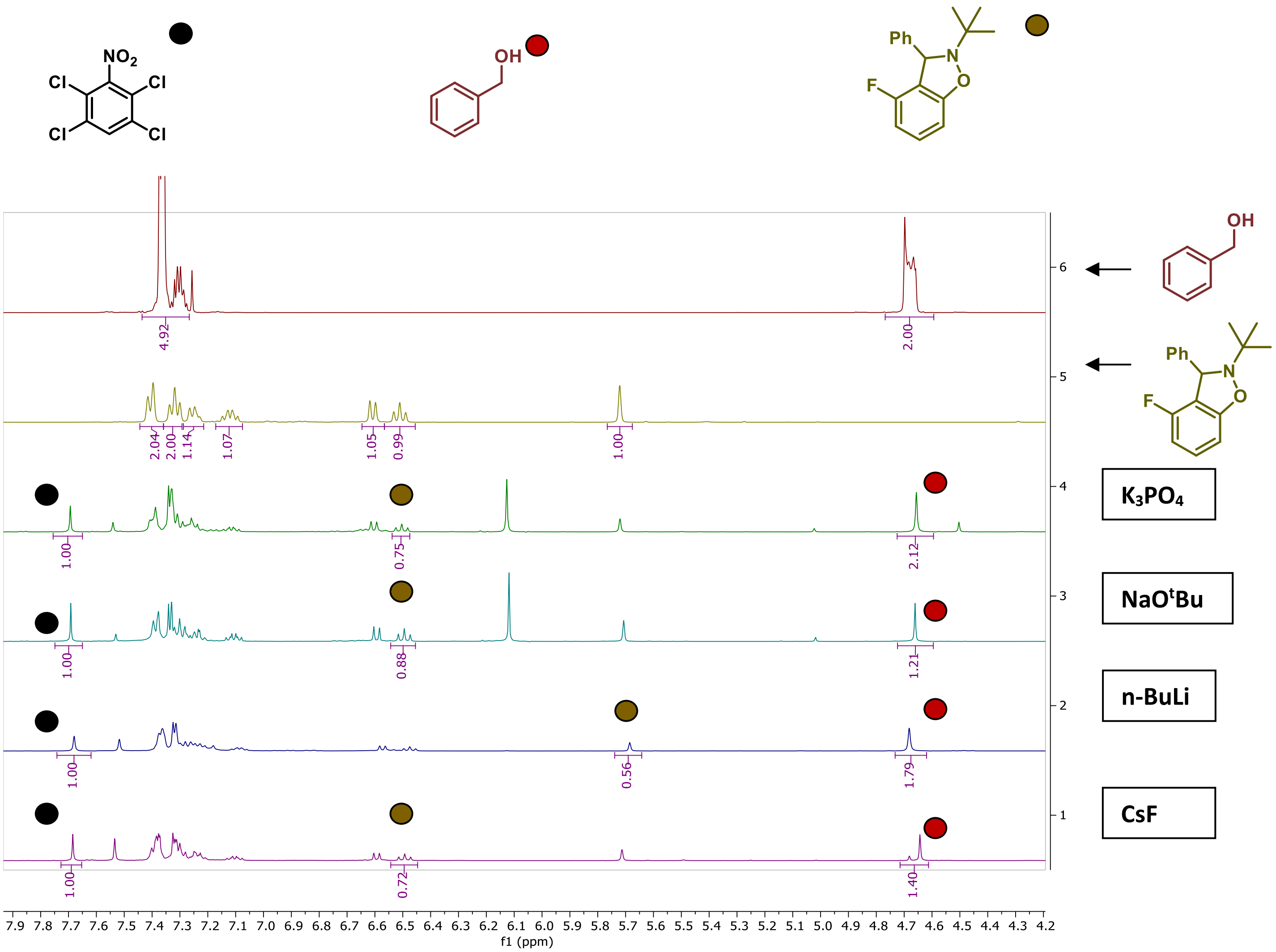

Base	Precursor Mass (mg)	Additive Mass (mg)	Mass TCNB(mg)	TCNB integral	Product Integral	Additive Integral (2H)	%Y Adduct	%Additive Remaining
K_3PO_4	55.4	10.8	20.8	1	0.75	2.12	60	85
NaO^{t}Bu	56.4	10.8	24.3	1	0.86	1.21	80	56
n-BuLi	24.4	10.8	24.1	1	0.56	1.79	52	83
CsF	31.6	10.8	27.5	1	0.72	1.4	76	74

Note- the additive remaining calculated for the CsF reaction may be falsely high due to a neighboring peak that has been included in the integral. This peak likely corresponds to the benzyl alcohol additive and appears shifted due to H bonding interactions in solution.

Additive: methyl benzoate

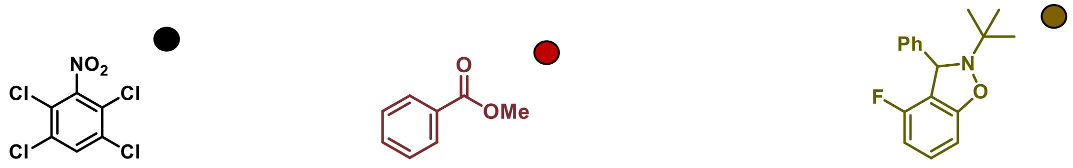

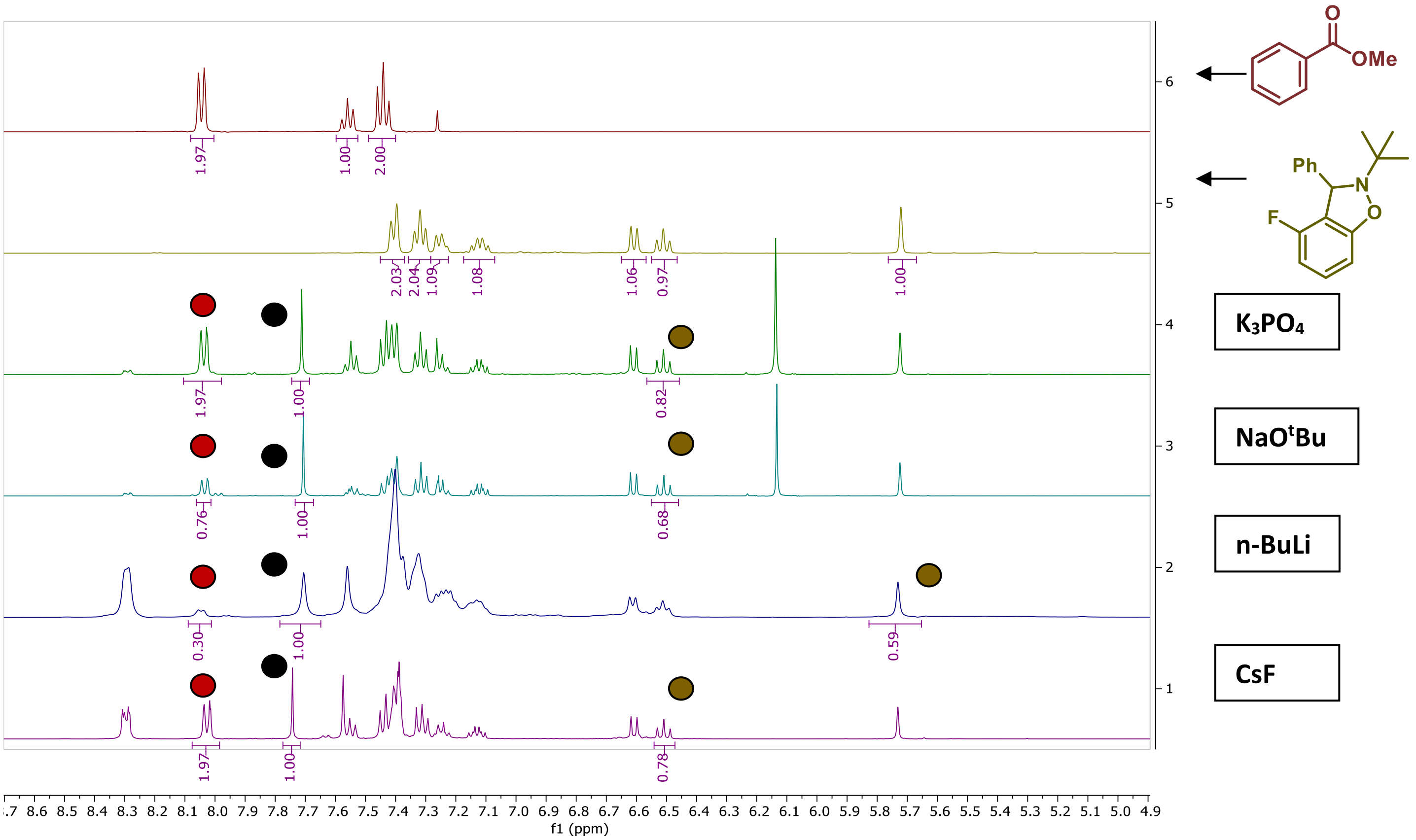

Base	Precursor Mass (mg)	Additive Mass (mg)	Mass TCNB(mg)	TCNB integral	Product Integral	Additive Integral (2H)	%Y Adduct	Additive Remaining
K_3PO_4	57.1	13.6	24.2	1	0.82	1.97	75	91
NaO^{t}Bu	55.5	13.6	31.3	1	0.68	0.76	82	46
n-BuLi	24.4	13.6	27.3	1	0.59	0.30	62	16
CsF	31.6	13.6	25.1	1	0.72	1.97	69	95

^{1}H, $^{13}C\{^{1}H\}$, and $^{19}F\{^{1}H\}$ NMR Spectra

^{1}H NMR of 3-Chloroiodobenzene diacetate (SI-1) in DMSO-*d6* at 400 MHz, 298K

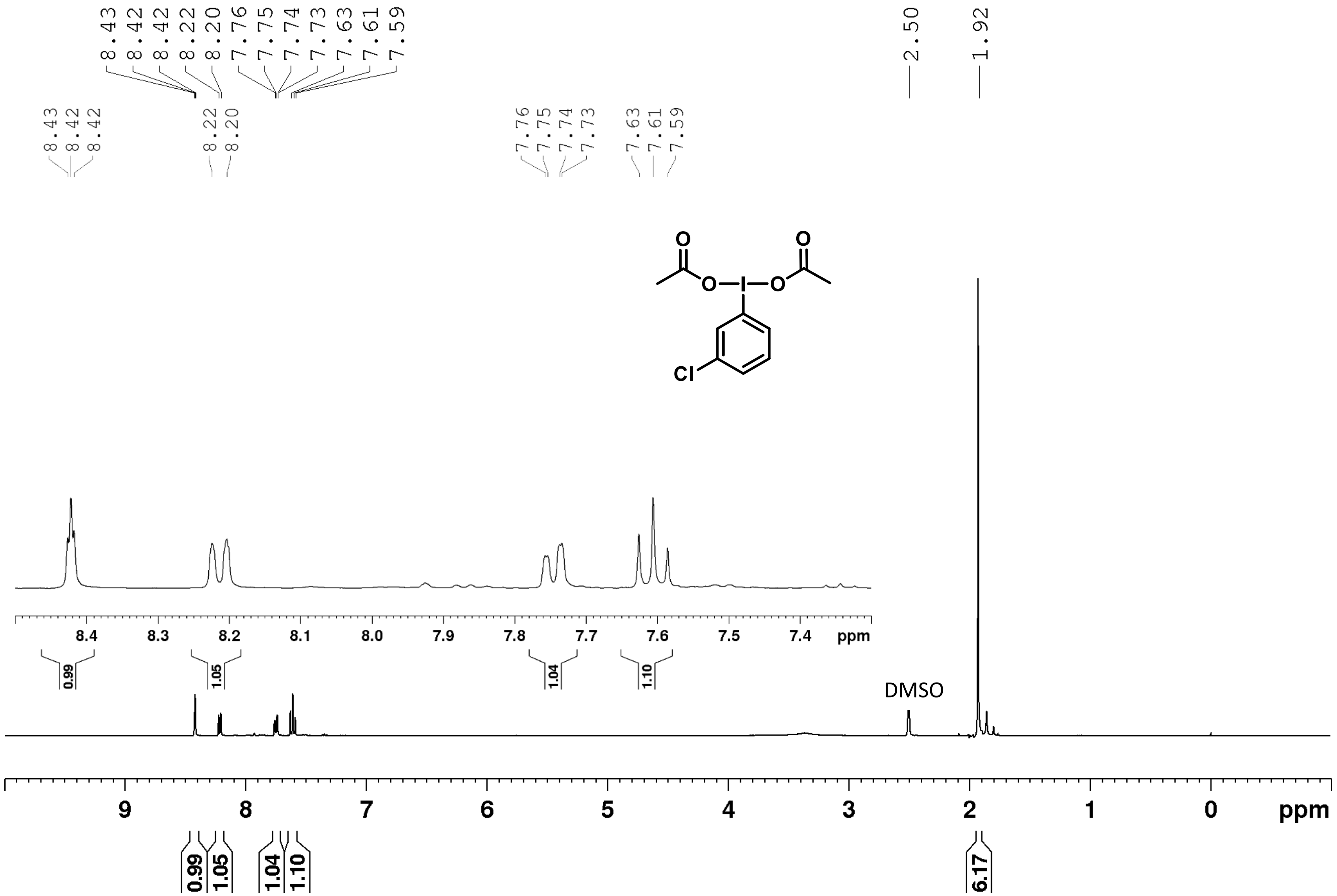

$^{13}C\{^{1}H\}$ NMR of 3-Chloroiodobenzene diacetate (SI-1) in DMSO-*d6* at 101 MHz, 298K

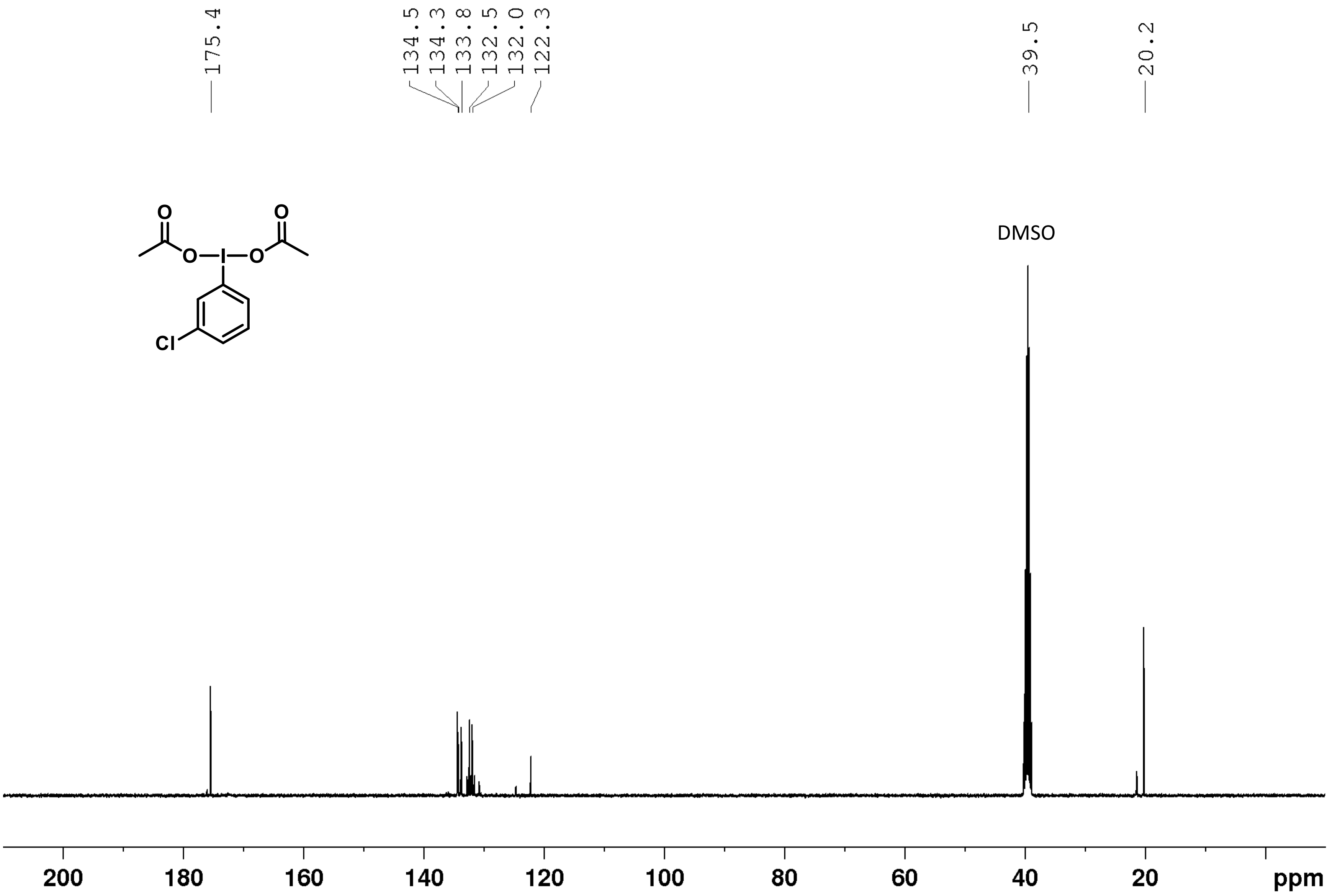

^{1}H NMR of Potassium (3,5-dimethylisoxazol-4-yl)trifluoroborate (SI-2) in DMSO-*d6* at 400 MHz, 298K

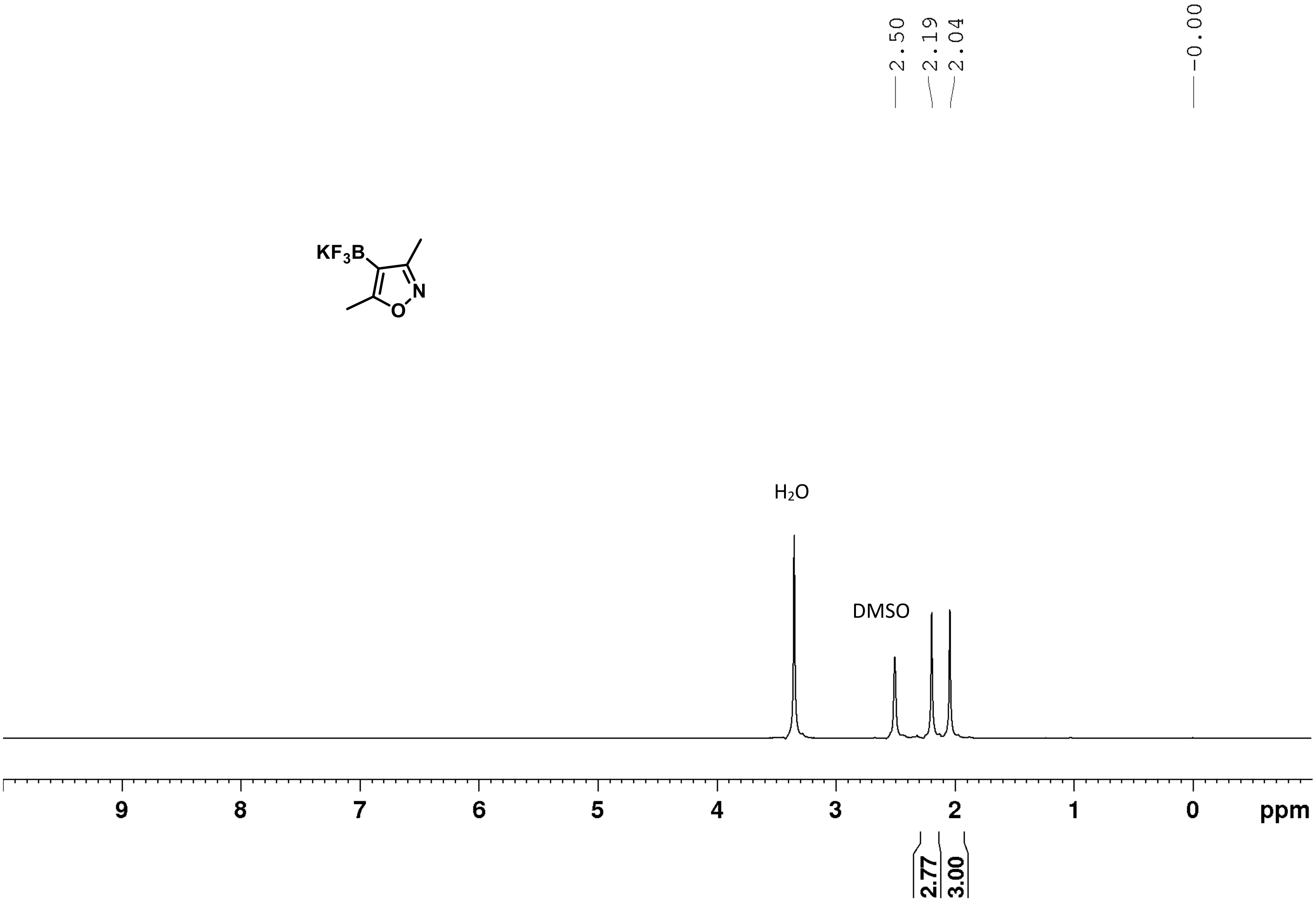

$^{13}C\{^1H\}$ NMR of Potassium (3,5-dimethylisoxazol-4-yl)trifluoroborate (SI-2) in DMSO-*d6* at 101 MHz, 298K

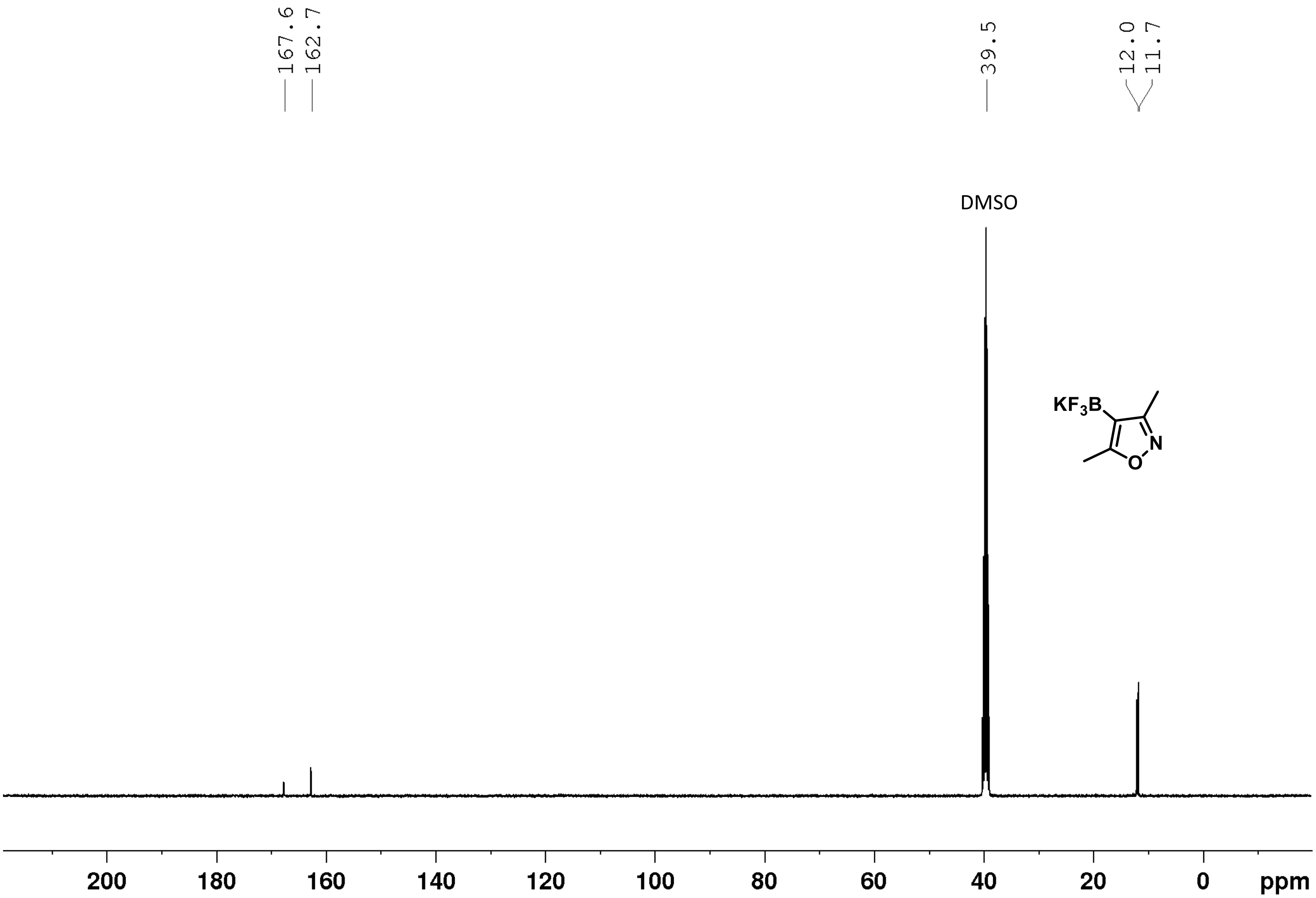

^{19}F-NMR of Potassium (3,5-dimethylisoxazol-4-yl)trifluoroborate (SI-2) DMSO-*d6* at 376 MHz, 298K

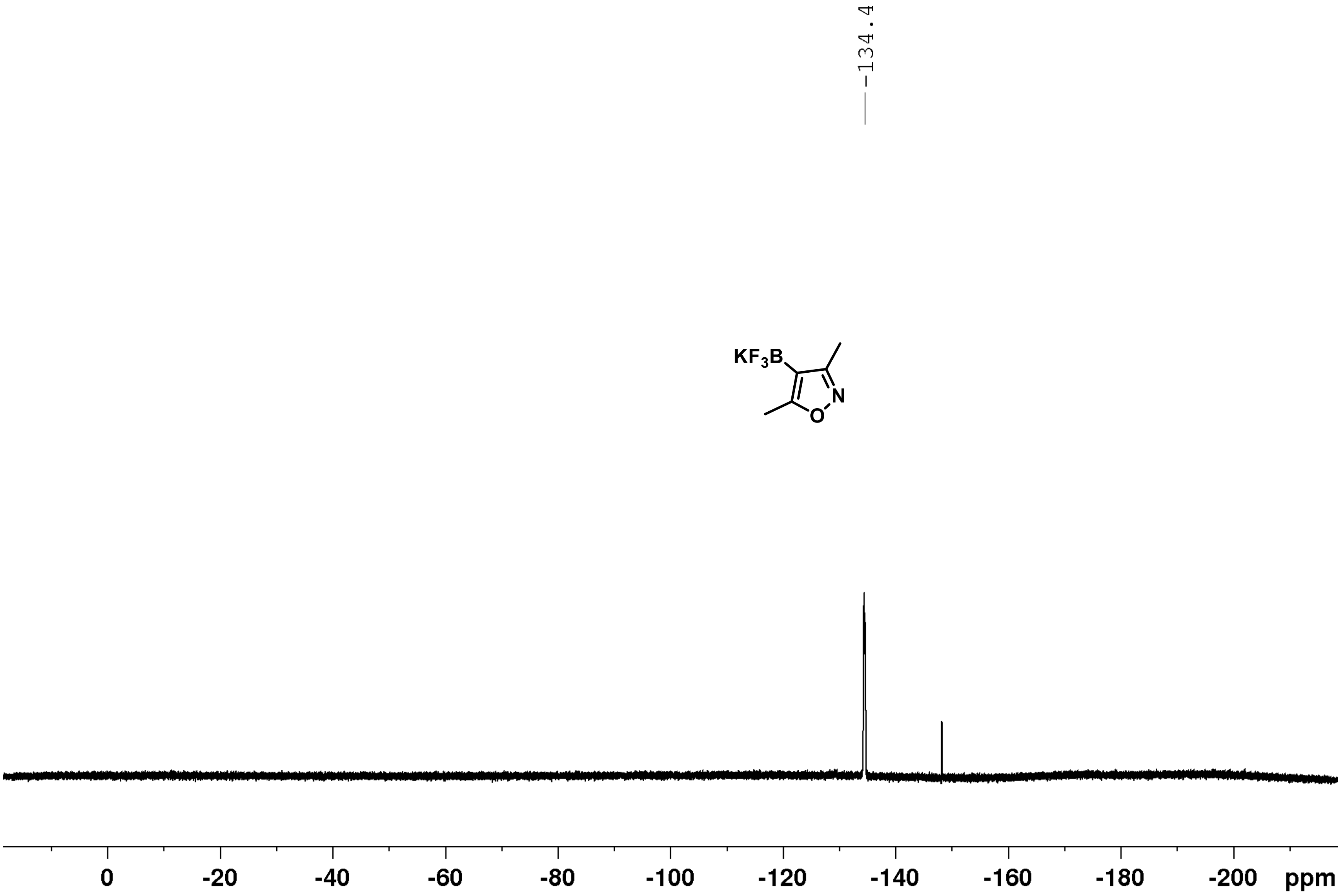

^{1}H NMR of Compound 3-chlorophenyl(3,5-dimethylisoxazole)iodonium tosylate (SI-3) in DMSO-*d6* at 400 MHz, 298K

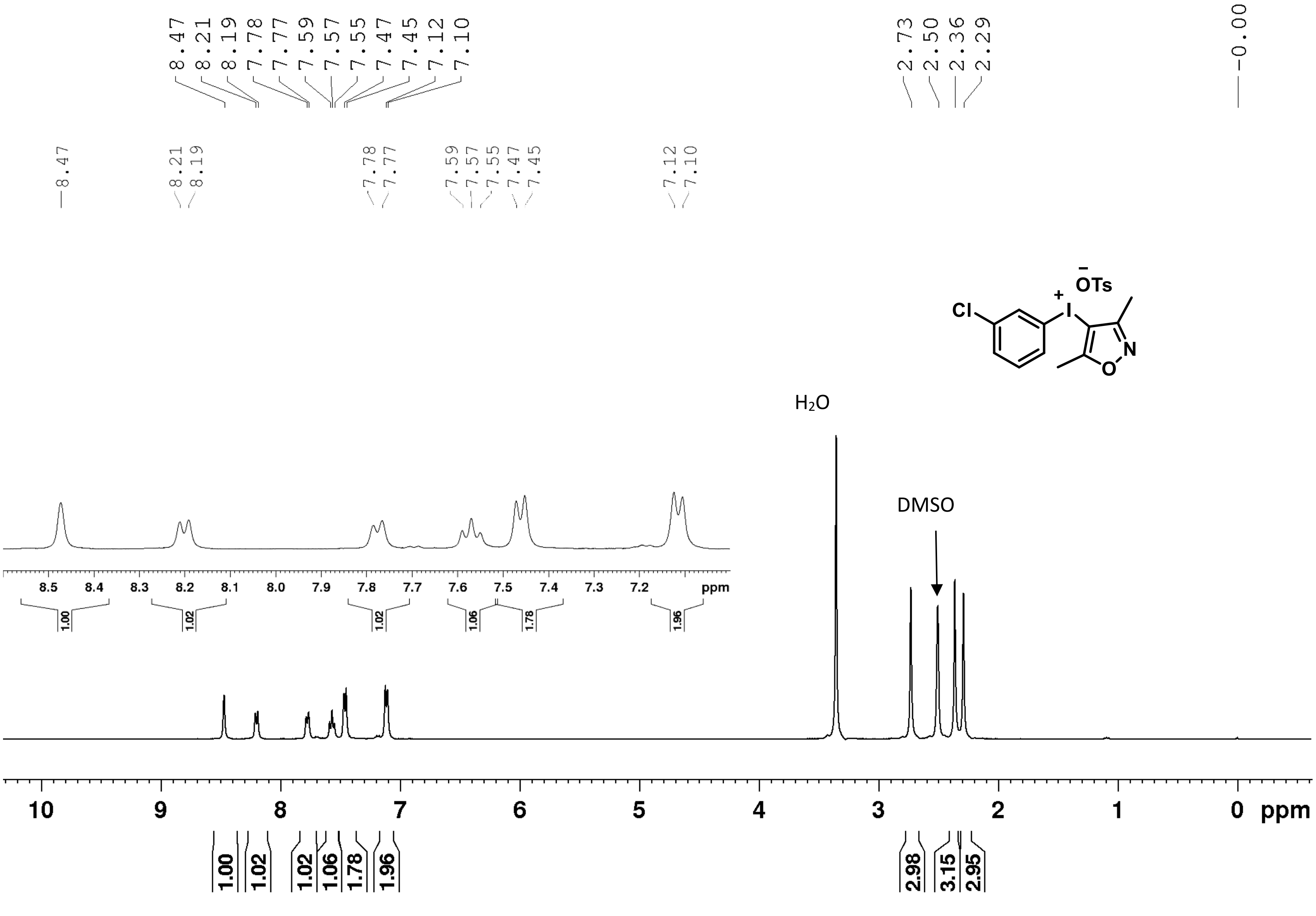

$^{13}C\{^1H\}$ NMR of 3-chlorophenyl(3,5-dimethylisoxazole)iodonium Tosylate (SI-3) in DMSO-*d6* at 101 MHz, 298K

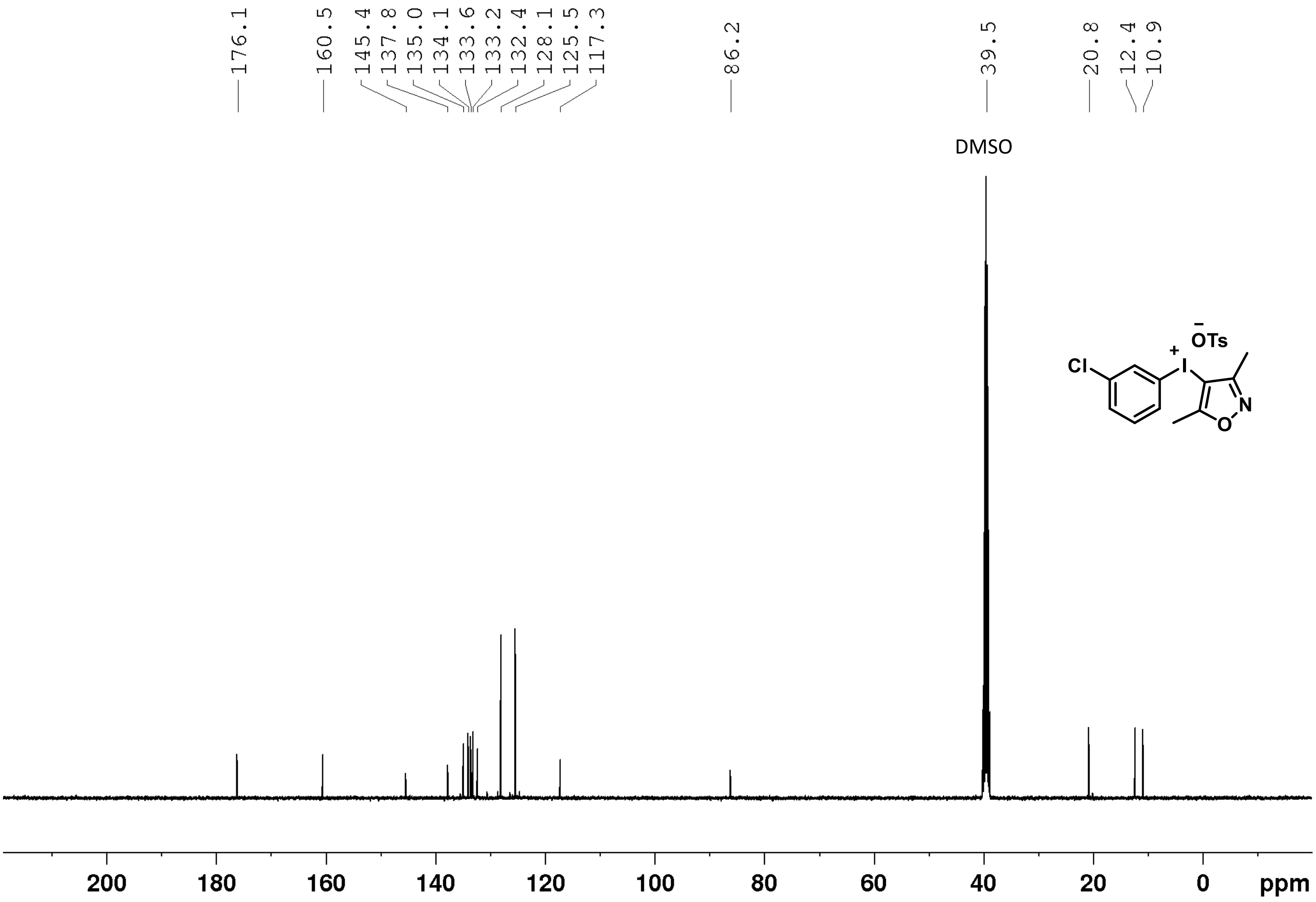

^{1}H-NMR of Compound 3-chlorophenyl-thianthrenium triflate (SI-4) in DMSO-*d6* at 400 MHz, 298K

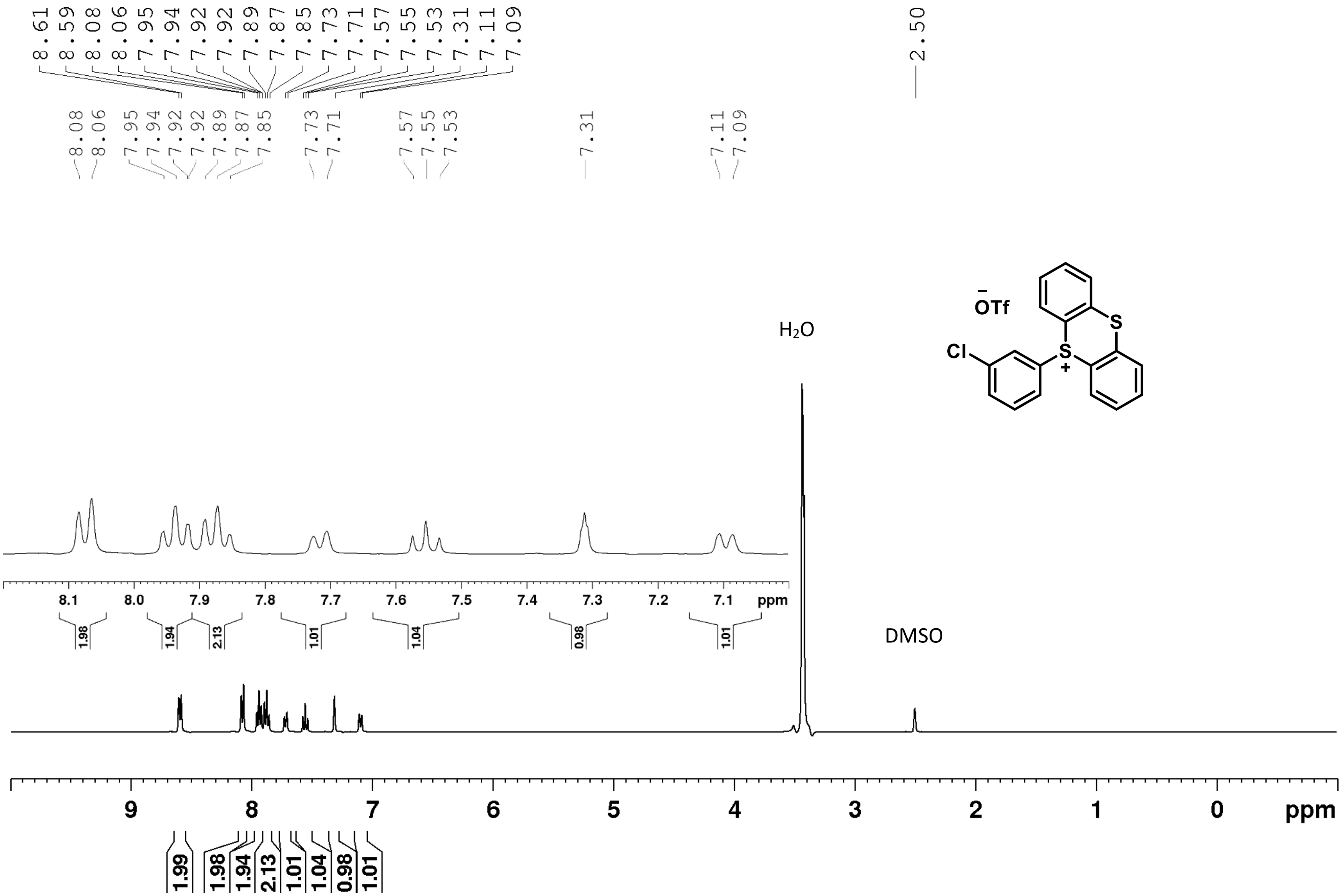

<u>^{13}C{^{1}H} NMR of 3-chlorophenyl-thianthrenium triflate (SI-4) in DMSO-*d6* at 100 MHz, 298K</u>

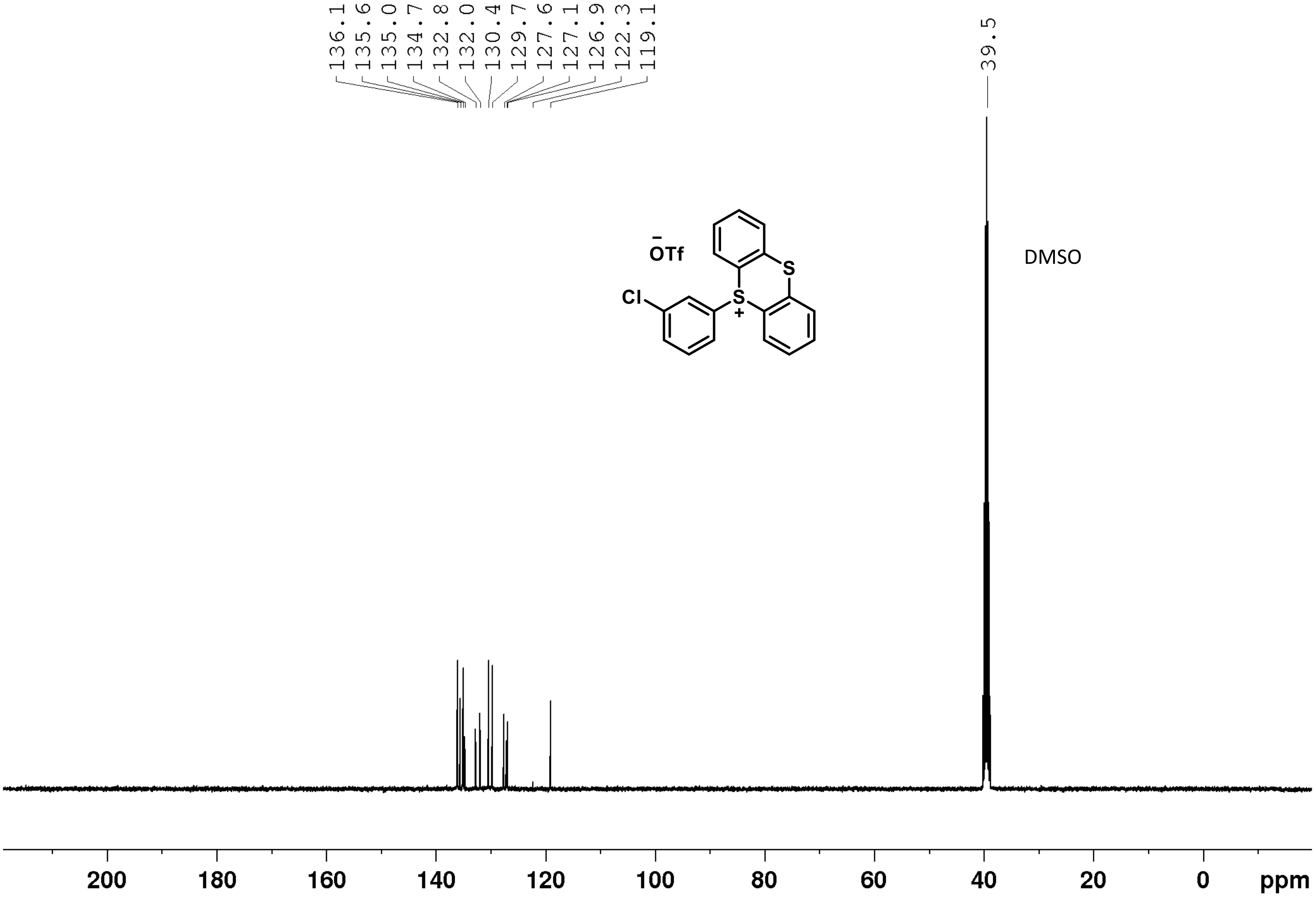

$^{19}F\{^{1}H\}$ NMR of 3-chlorophenyl-thianthrenium triflate (SI-4) in DMSO at 376 MHz, 298K

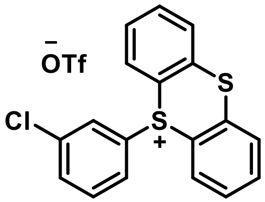

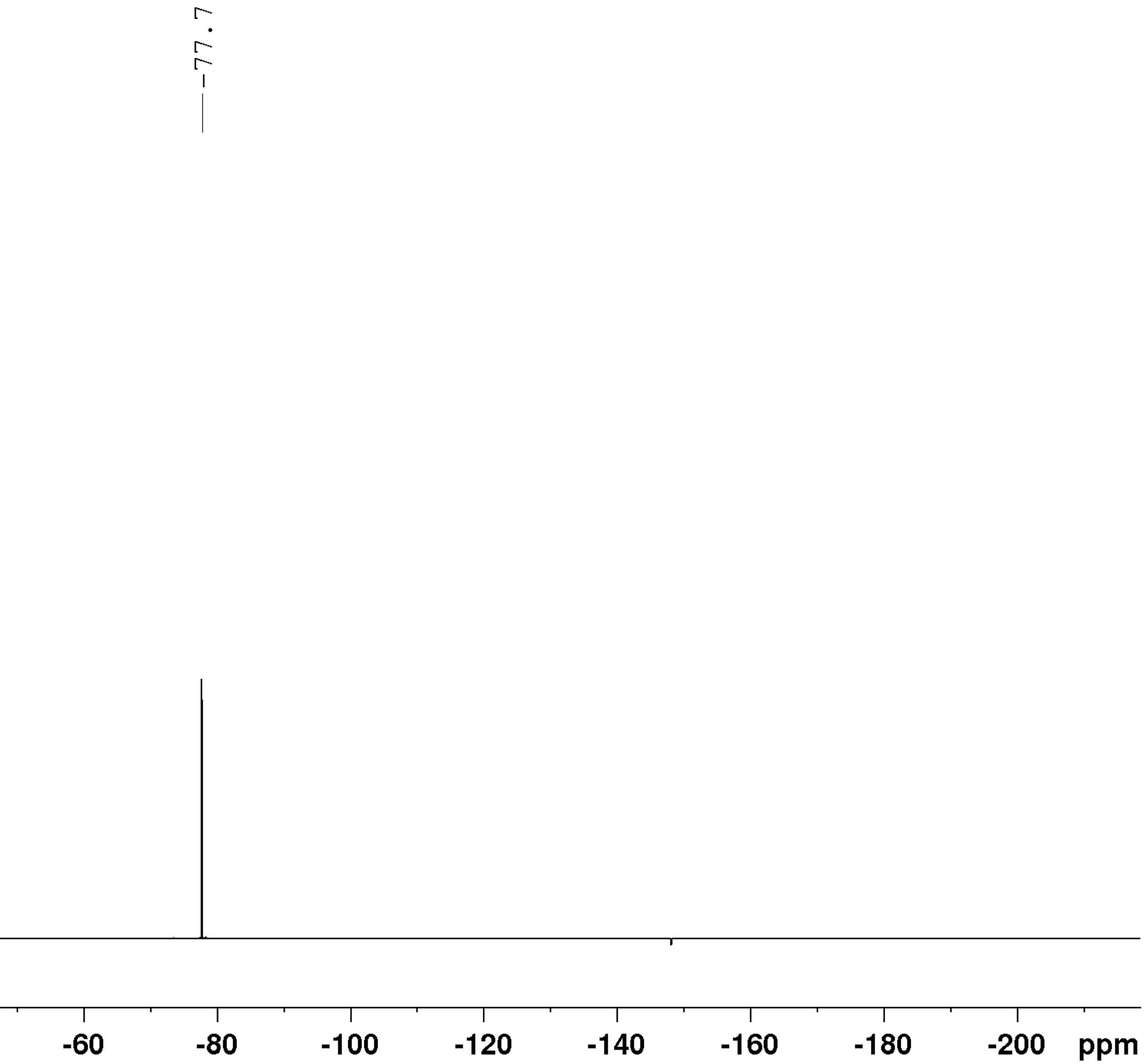

^{1}H-NMR of 1-(3-fluorophenyl)-2,2,6,6-tetramethylpiperidine (SI-5) in $CDCl_3$ at 400 MHz, 298K

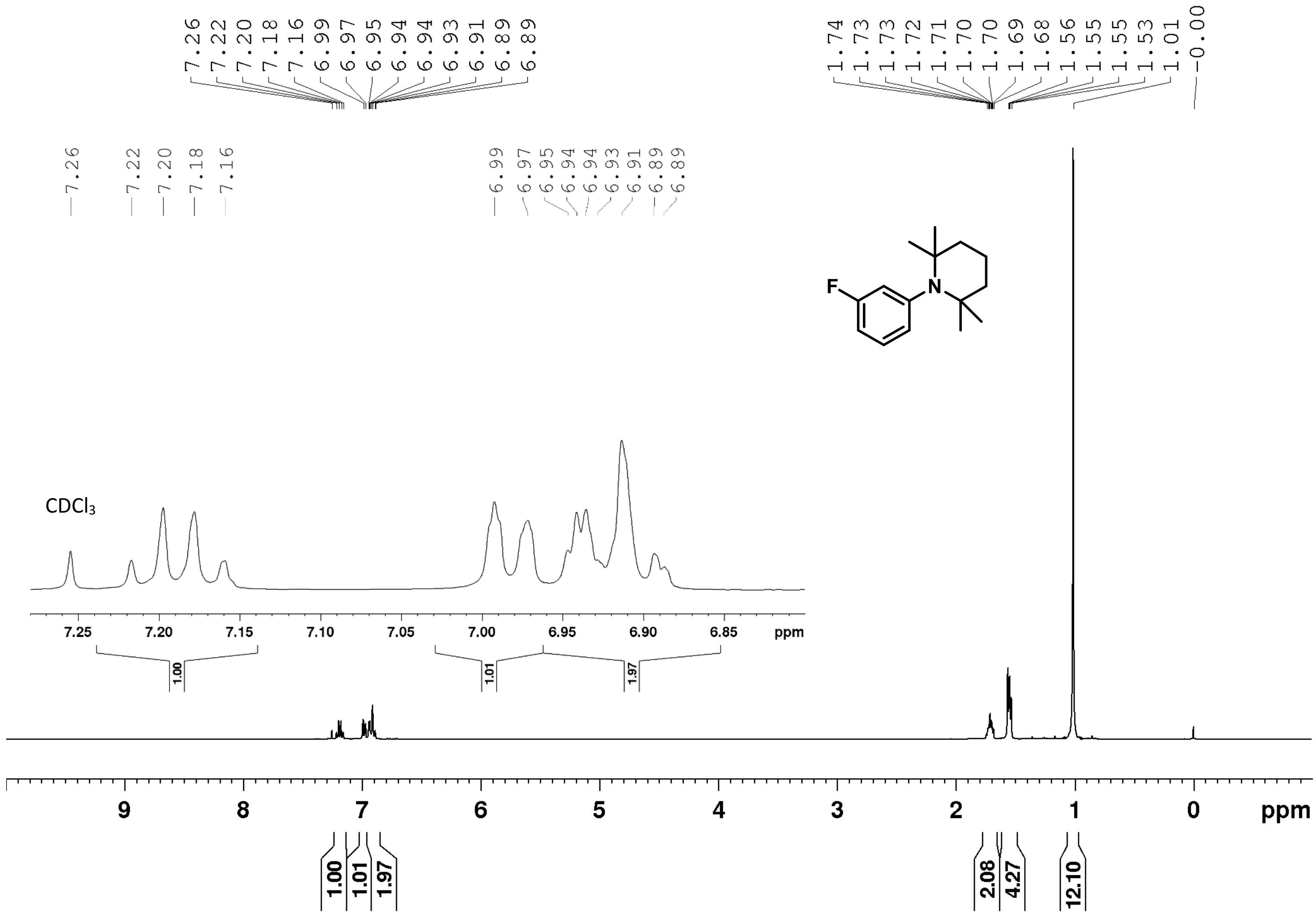

$^{13}C\{^1H\}$ NMR of 1-(3-fluorophenyl)-2,2,6,6-tetramethylpiperidine (SI-5) in $CDCl_3$ at 100 MHz, 298K

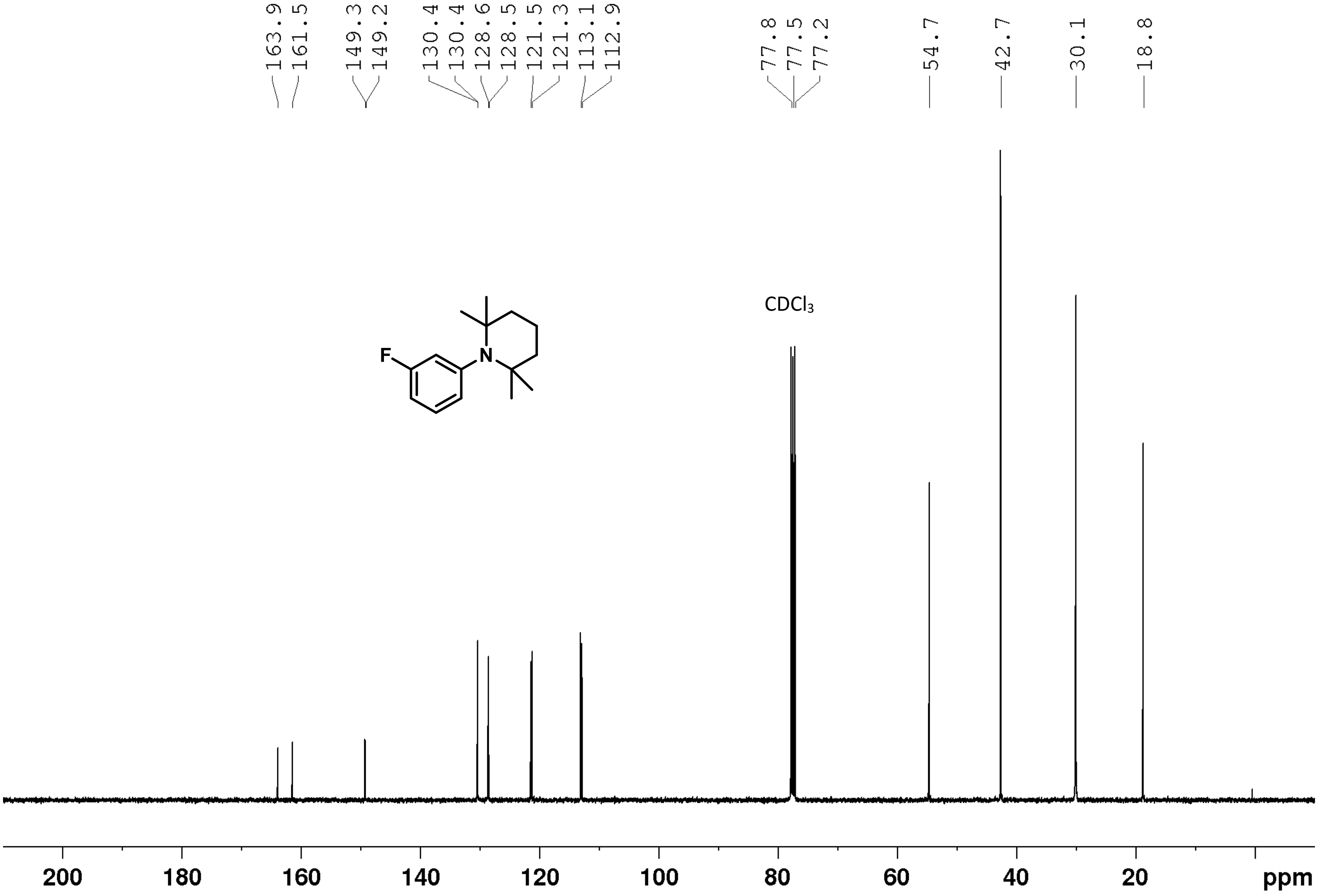

$^{19}F\{^{1}H\}$ NMR of 1-(3-fluorophenyl)-2,2,6,6-tetramethylpiperidine (SI-5) in $CDCl_3$ at 376MHz, 298K

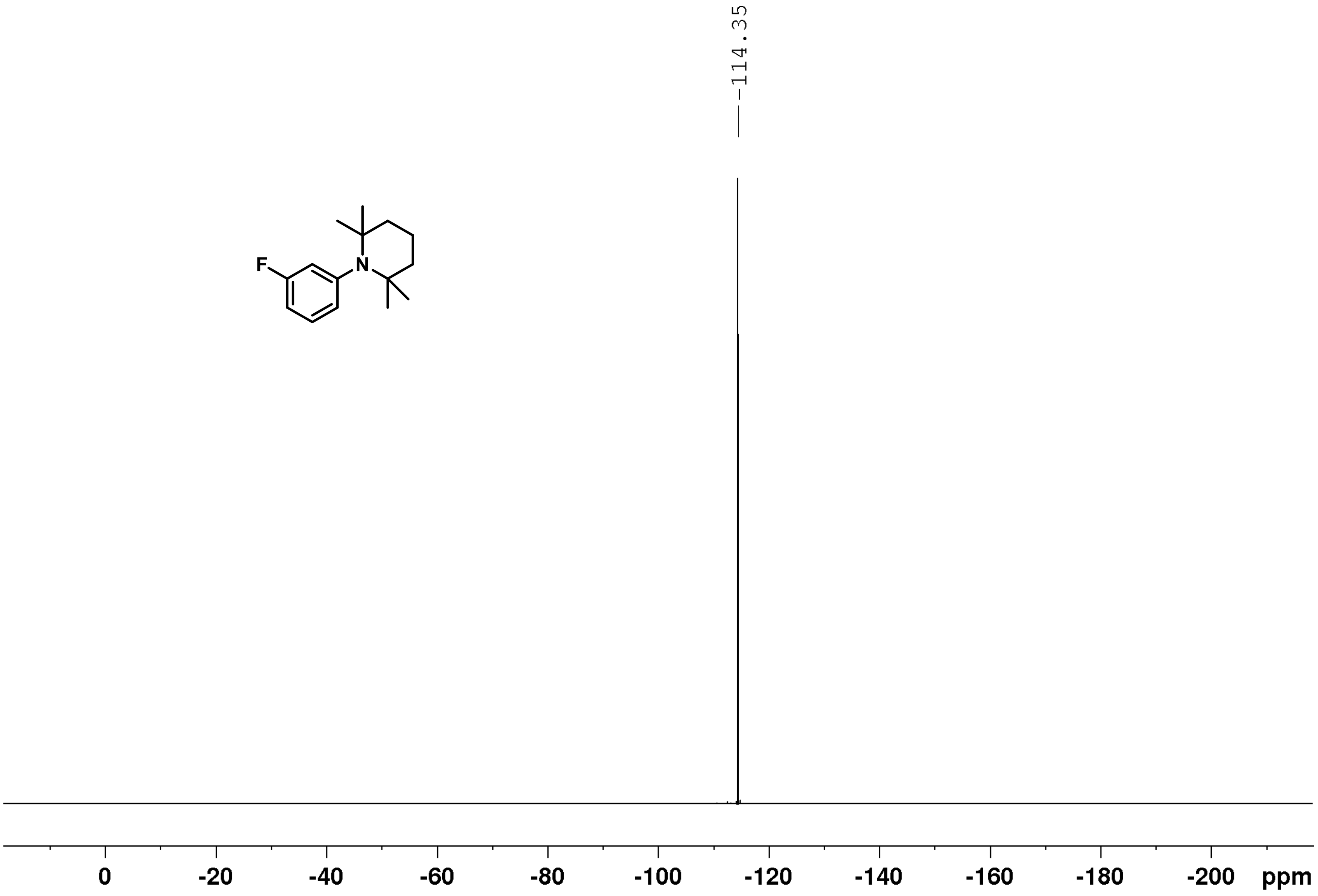

^{1}H-NMR of 3-fluoro-N,N-diisopropylaniline in $CDCl_3$ (SI-6) at 400 MHz, 298K

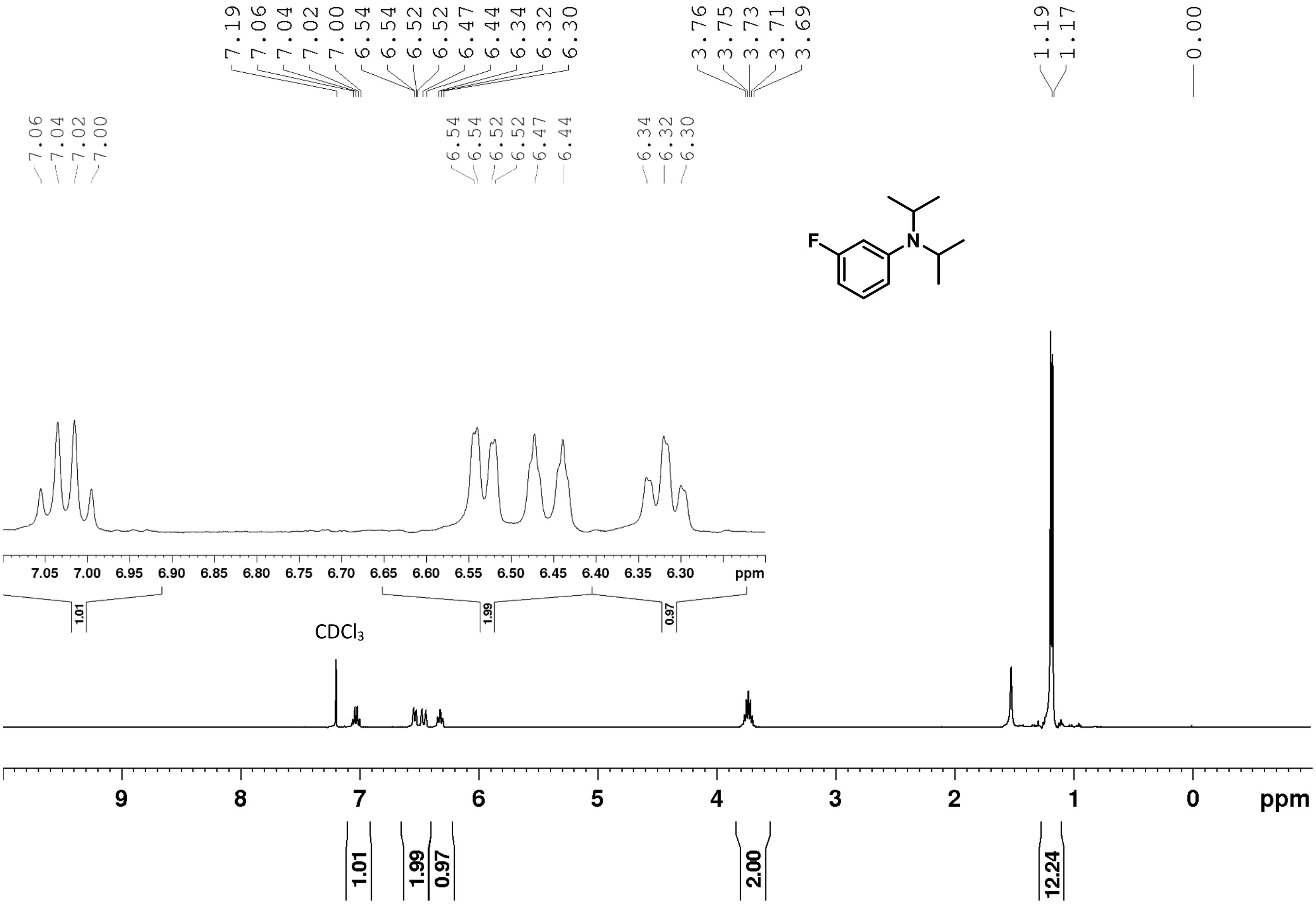

$^{13}C\{^{1}H\}$ NMR of 3-fluoro-N,N-diisopropylaniline (SI-6) in $CDCl_3$ at 100 MHz, 298K

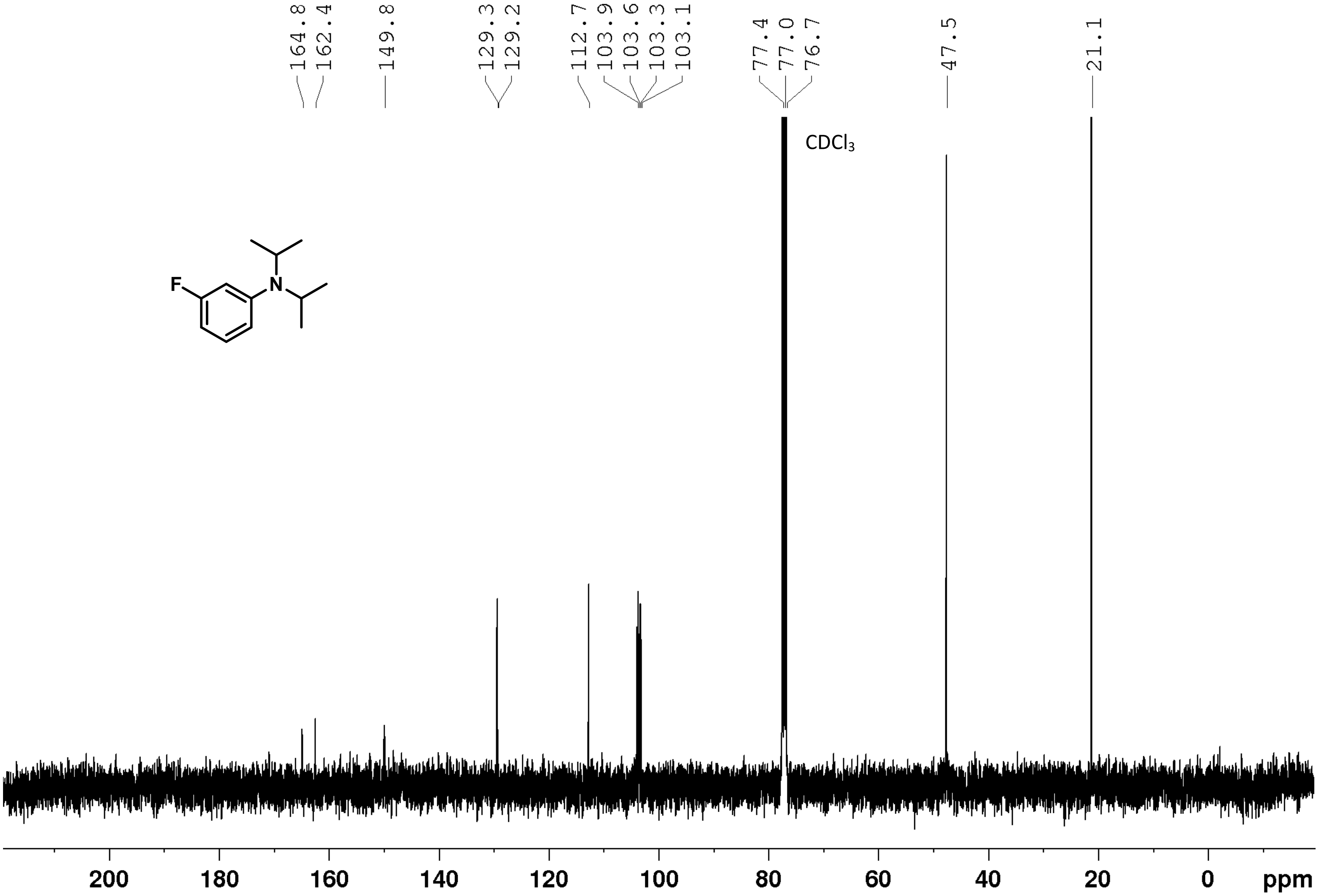

$^{19}F\{^{1}H\}$ NMR of 3-fluoro-N,N-diisopropylaniline (SI-6) in $CDCl_3$ at 376 MHz, 298K

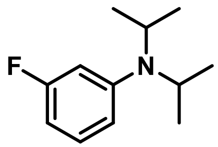

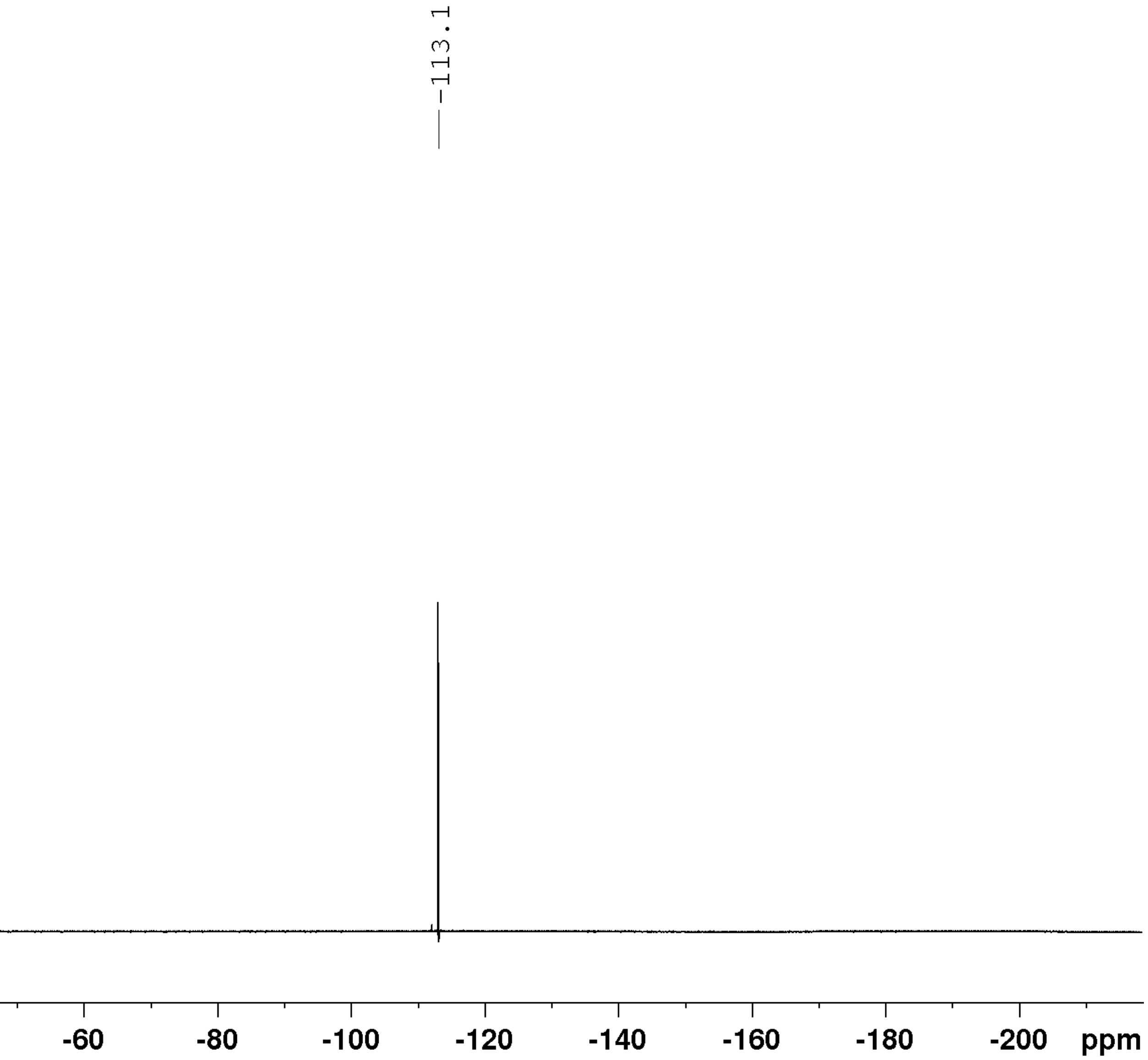

^{1}H-NMR of tert-butyl(4-chlorophenoxy)dimethylsilane (15) in $CDCl_3$ at 400 MHz, 298K

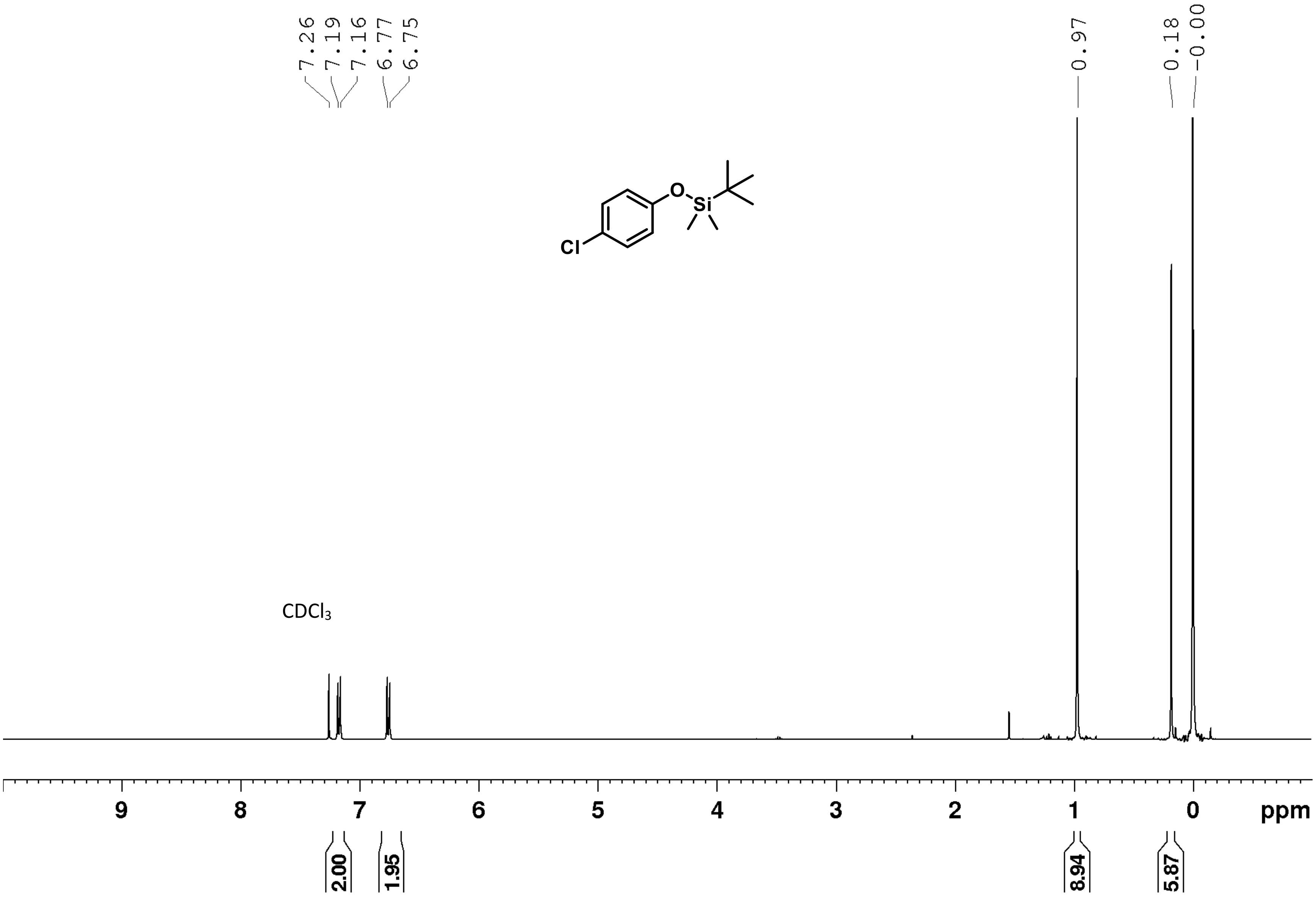

$^{13}C\{^{1}H\}$ NMR of tert-butyl(4-chlorophenoxy)dimethylsilane (15) in $CDCl_3$ at 100 MHz, 298K

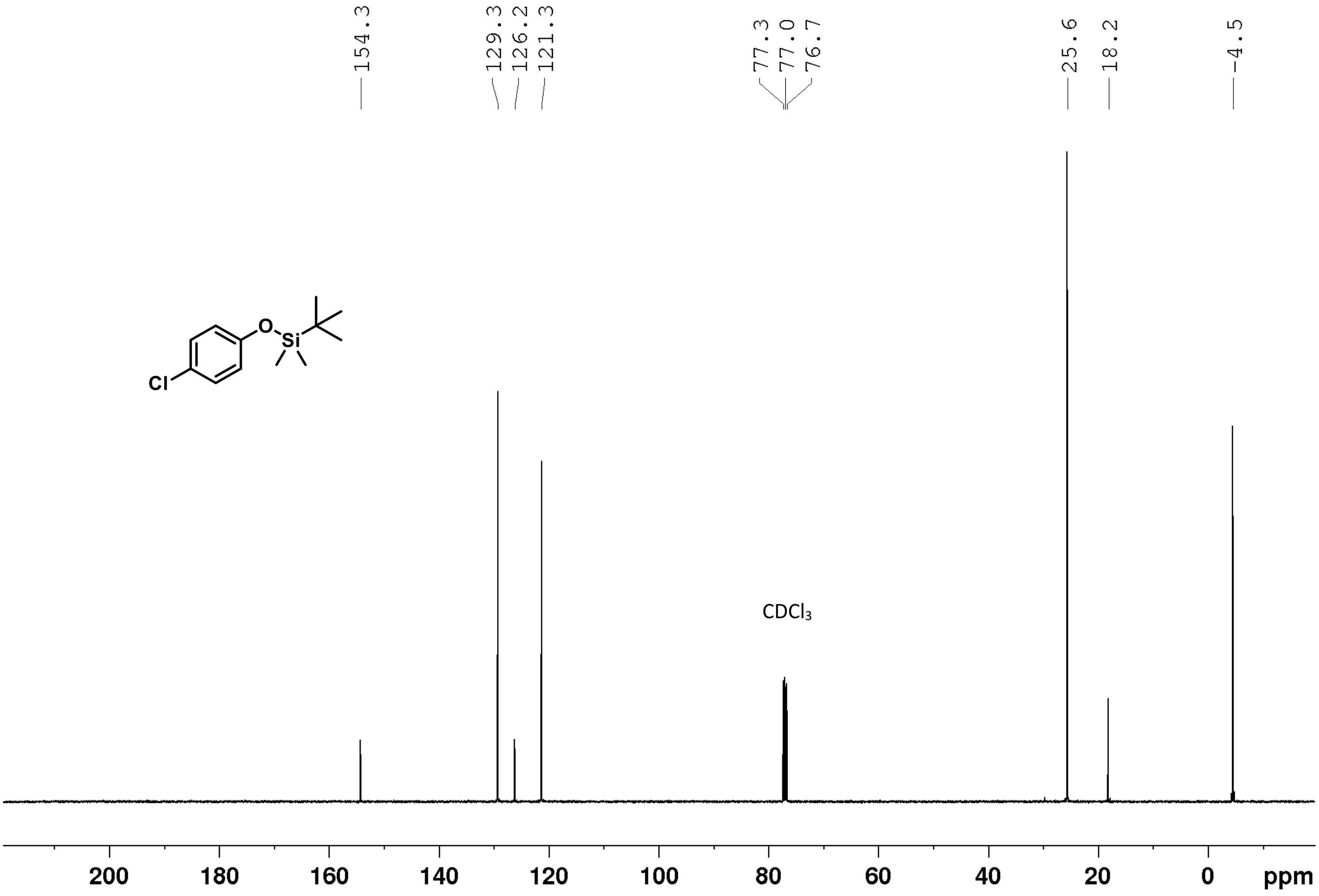

^{1}H-NMR of methyl 3-((3-iodophenoxy)methyl)benzoate (SI-7) in $CDCl_3$ at 400 MHz, 298K

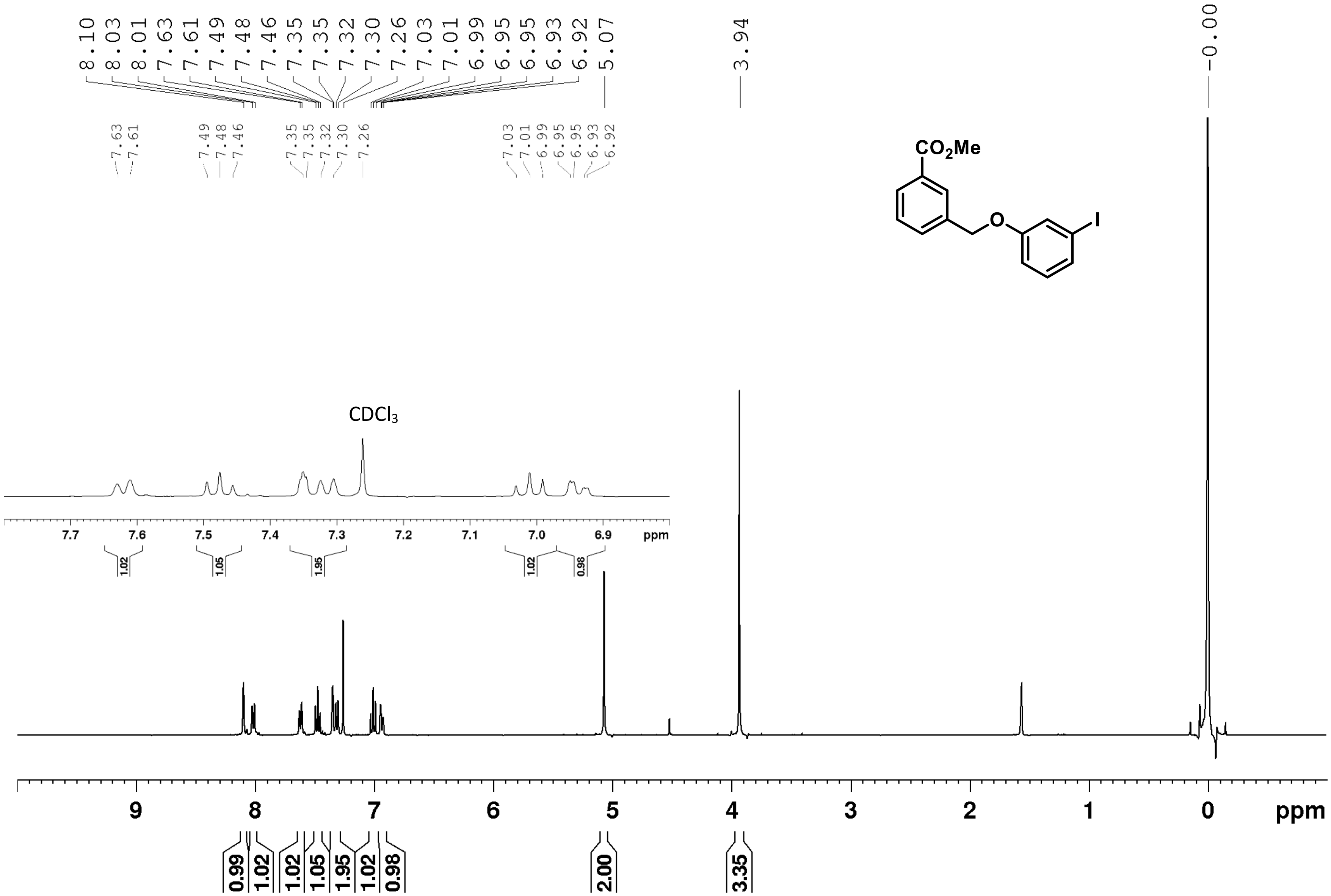

^{1}H-NMR of 1-(4-((3-iodophenoxy)methyl)phenyl)ethan-1-one (SI-8) in $CDCl_3$ at 400 MHz, 298K

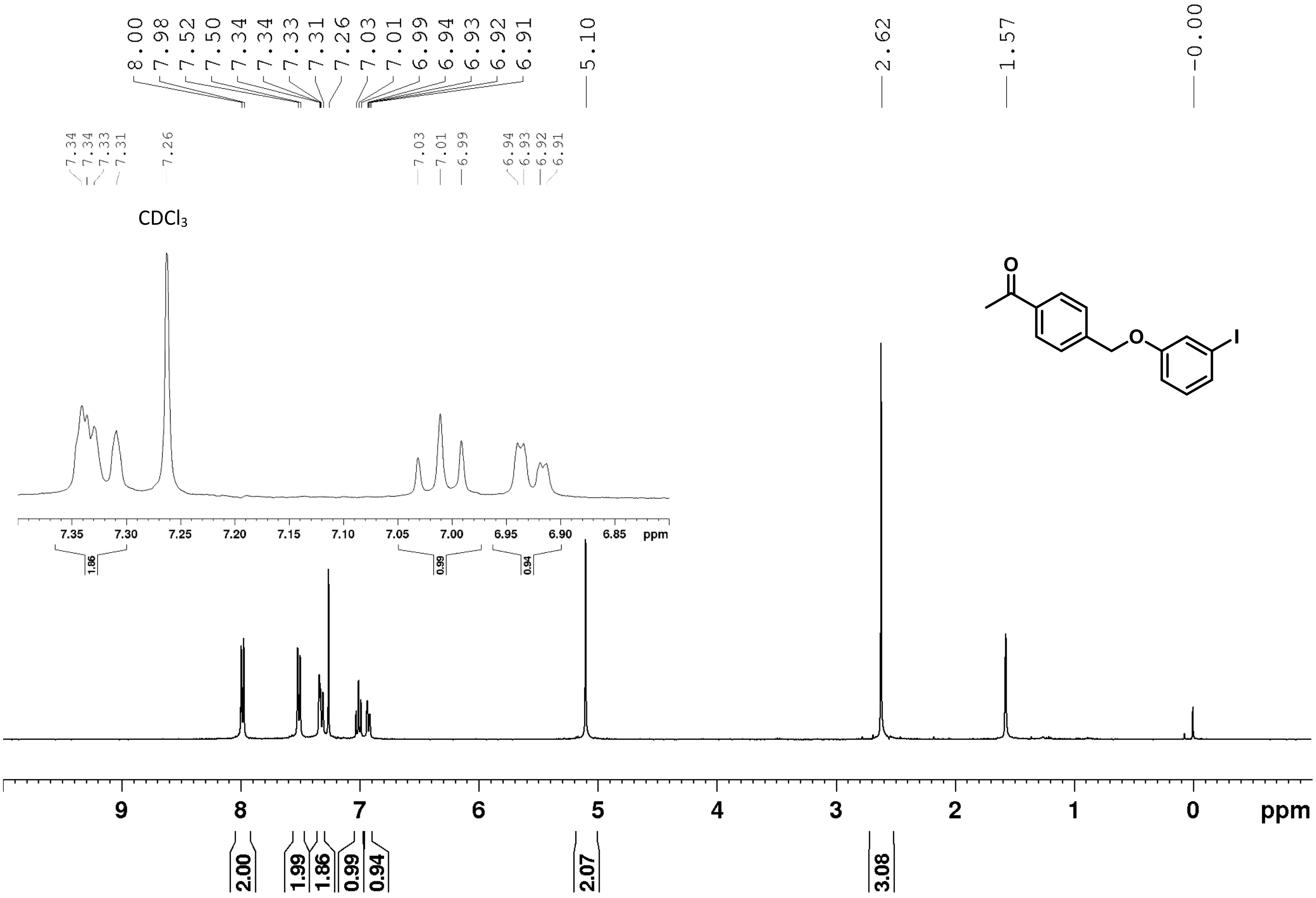

^{1}H-NMR of Compound 1k in DMSO-*d6* at 400 MHz, 298K

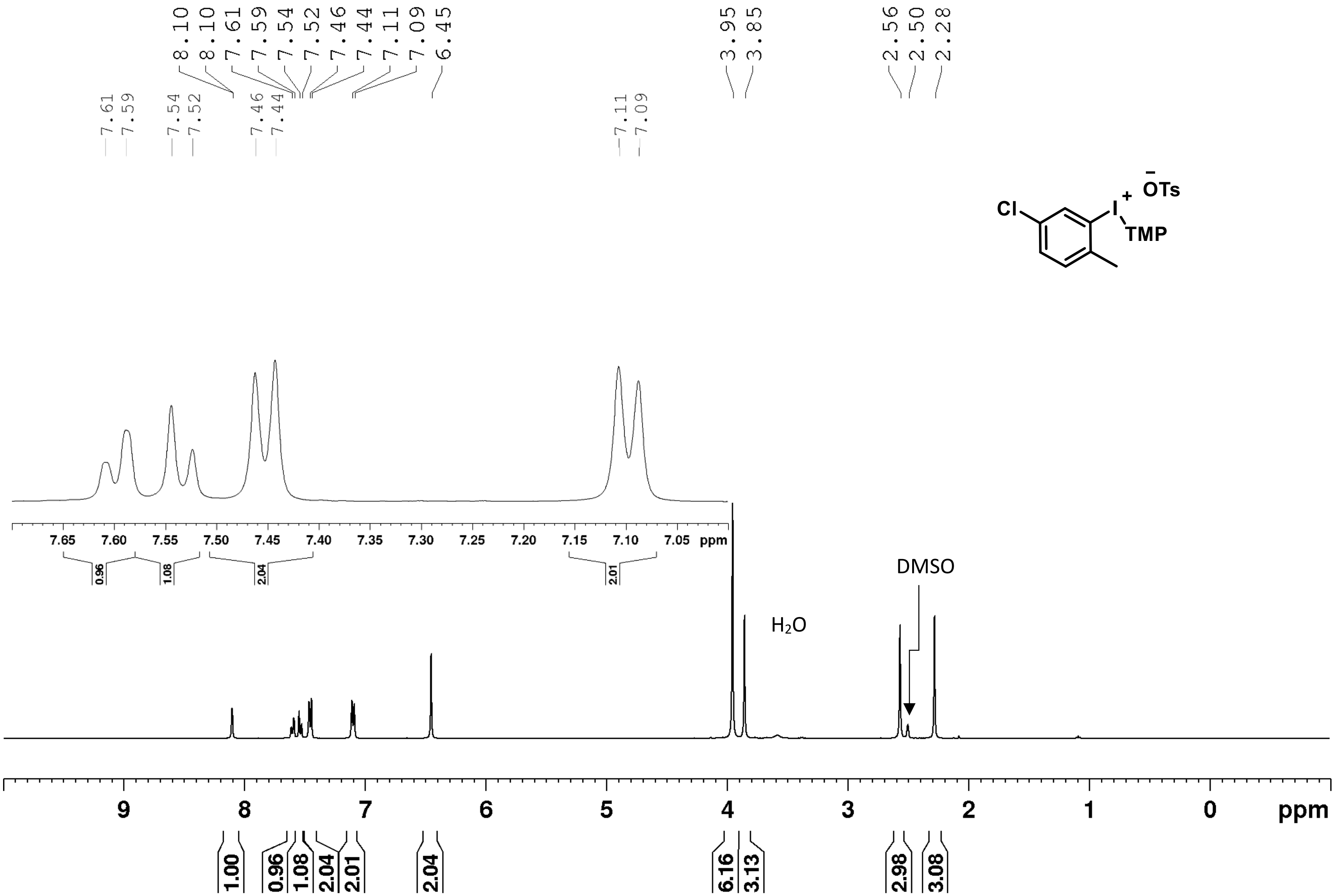

$^{13}C\{^{1}H\}$ NMR of Compound 1k in DMSO-*d6* at 100 MHz, 298K

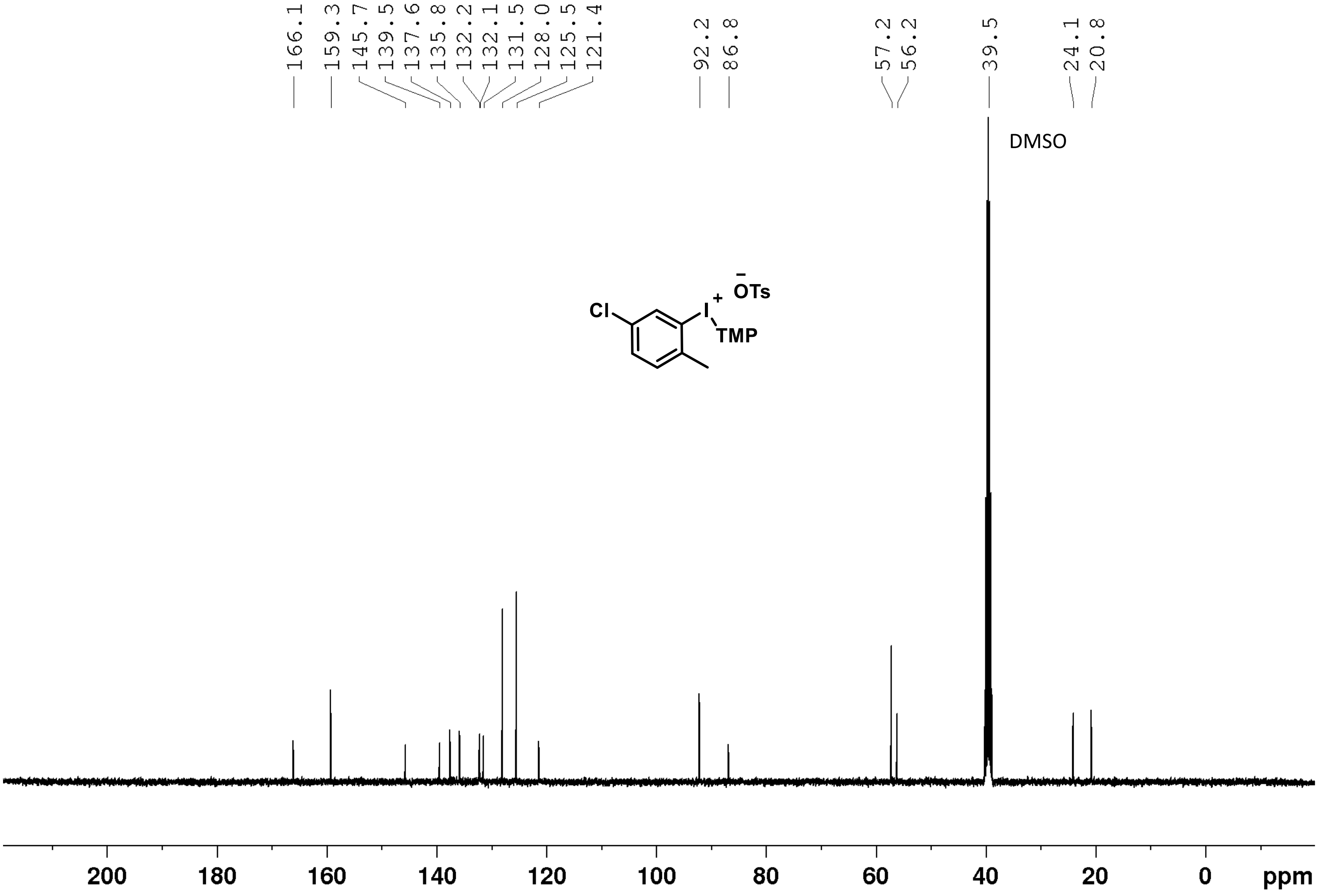

^{1}H-NMR of Compound 1m in DMSO-*d6* at 400 MHz, 298K

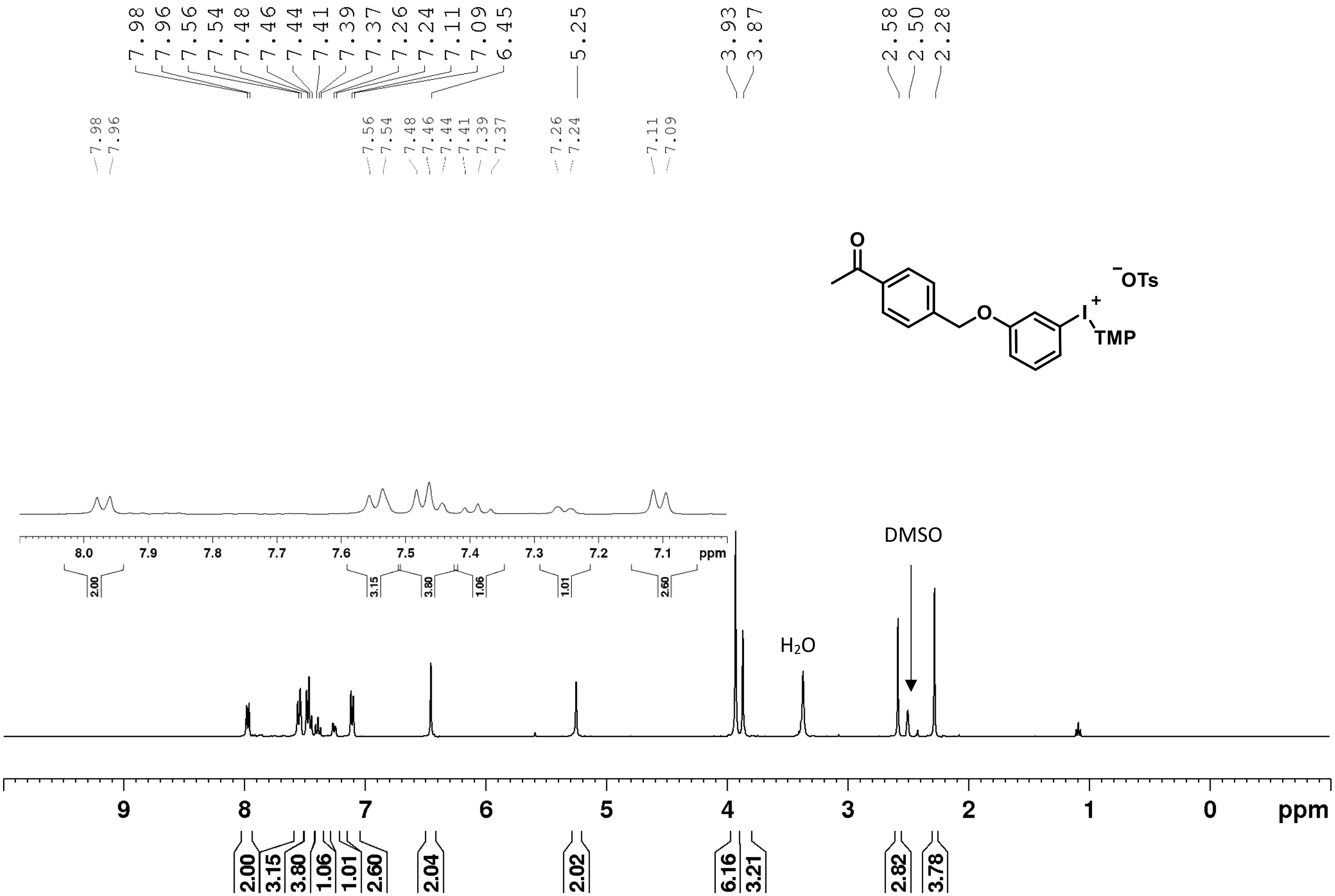

$^{13}C\{^{1}H\}$ NMR of Compound 1m in DMSO-*d6* at 100 MHz, 298K

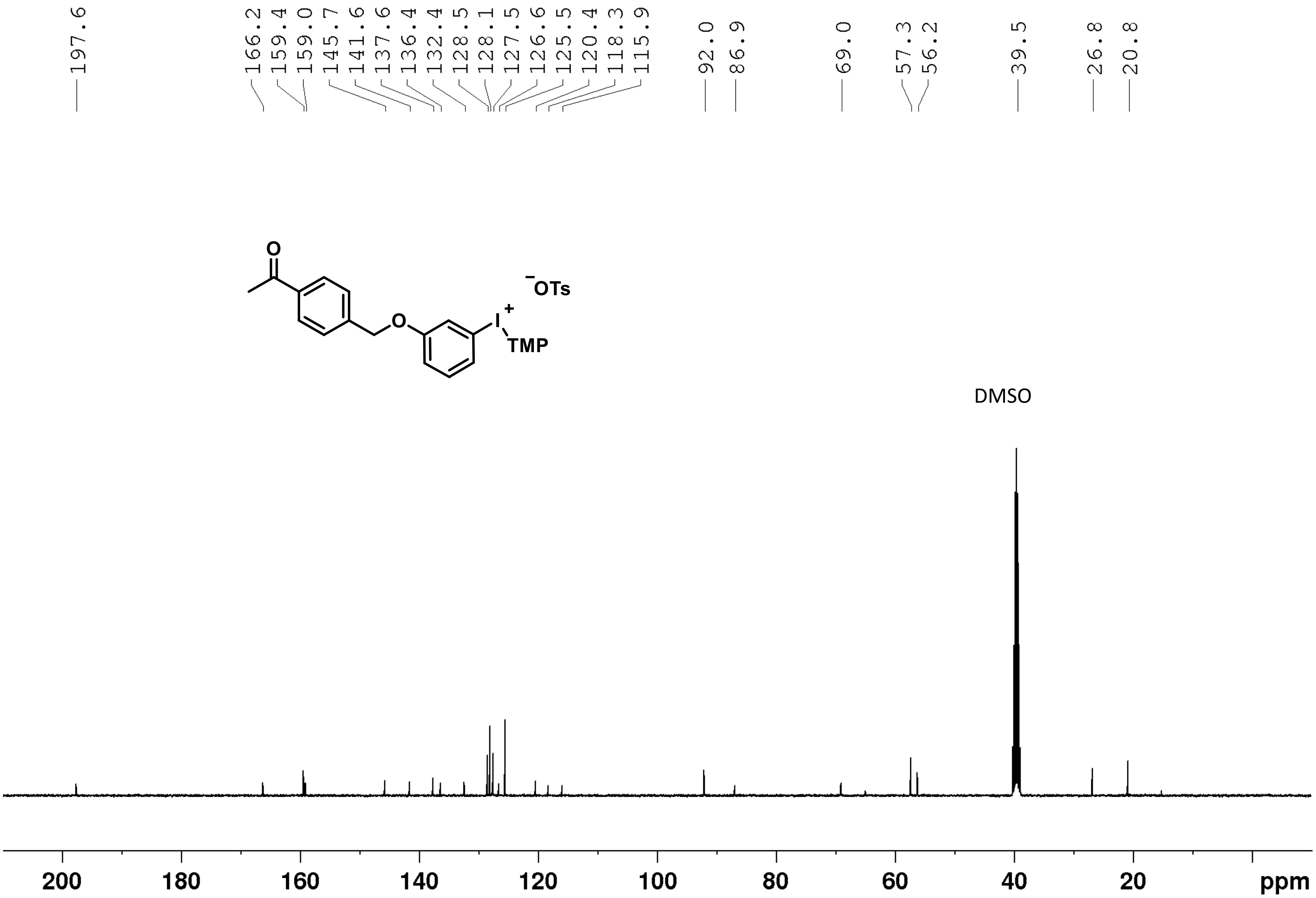

^{1}H-NMR of Compound 1n in DMSO-*d6* at 400 MHz, 298K

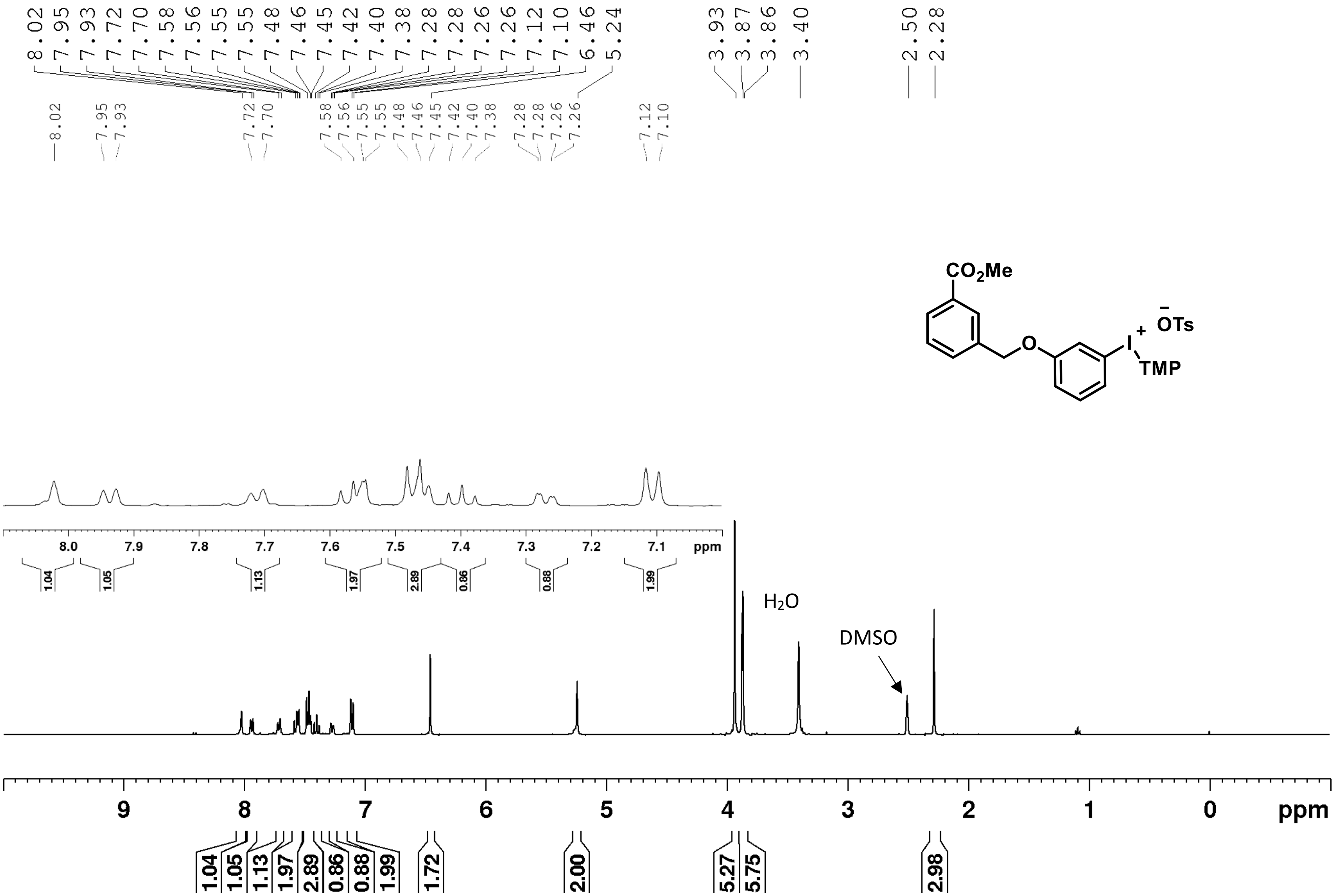

$^{13}C\{^1H\}$ NMR of Compound 1n in DMSO-*d6* at 100 MHz, 298K

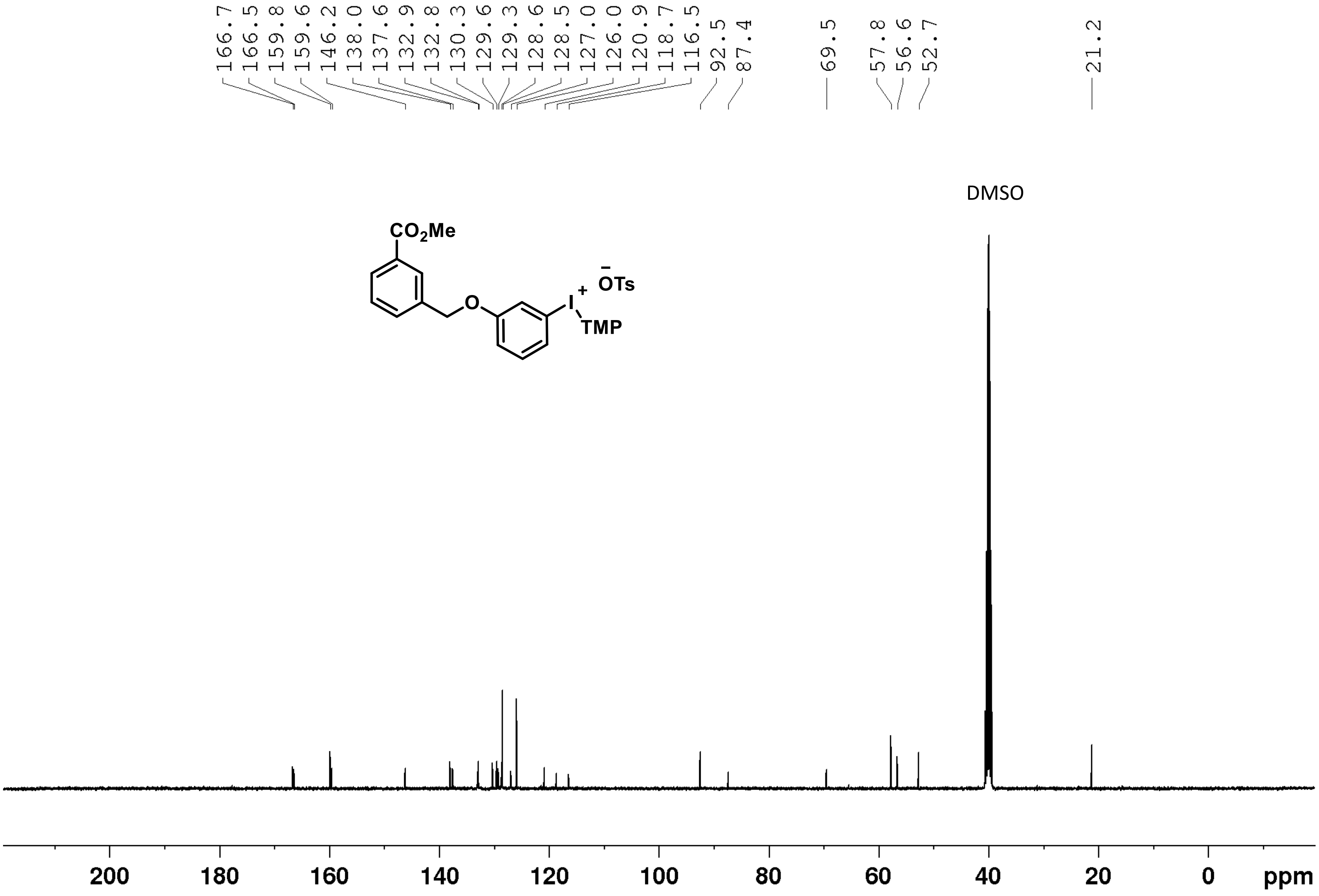

^{1}H-NMR of Compound 2f in $CDCl_3$ at 400 MHz, 298K

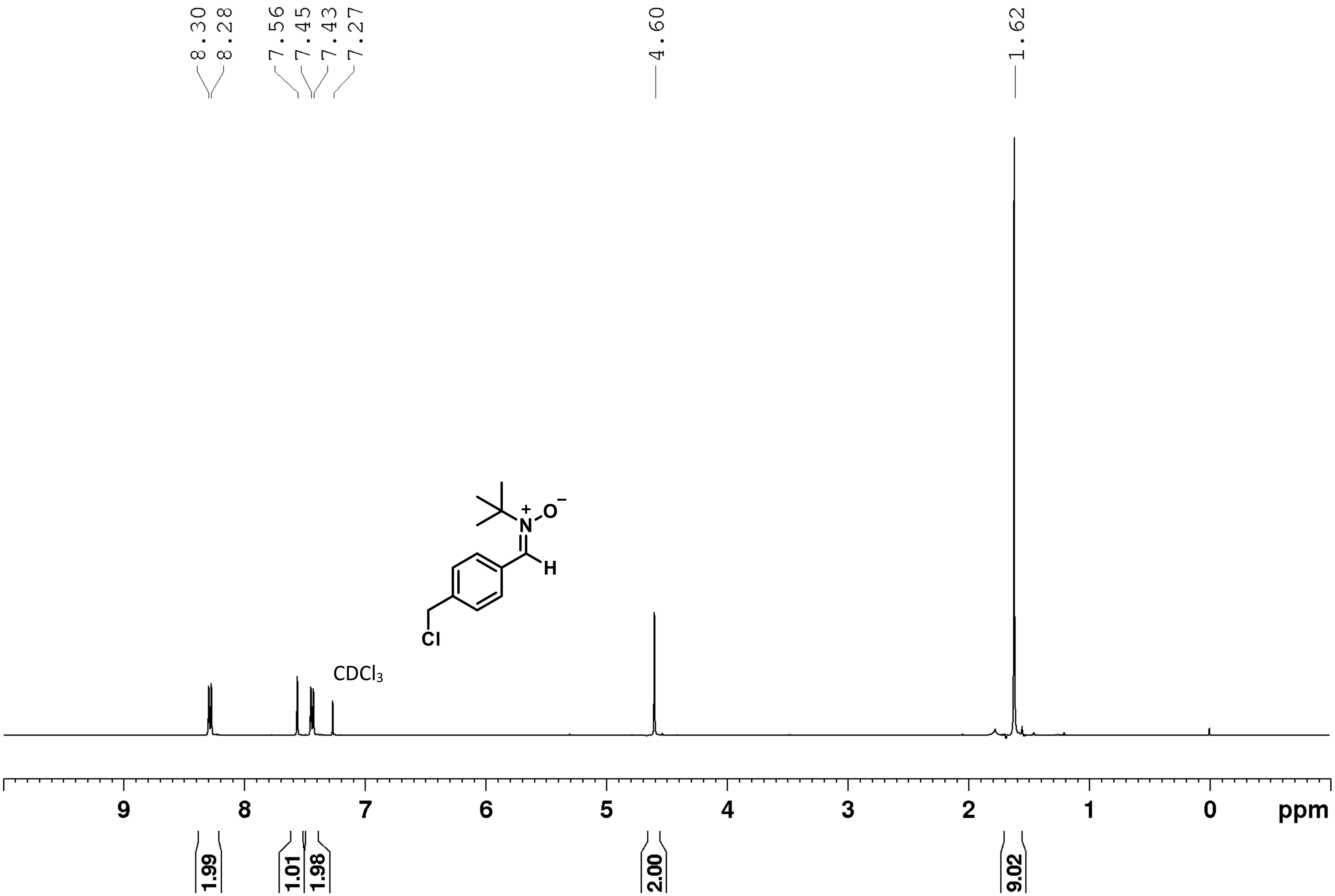

$^{13}C\{^{1}H\}$ NMR of Compound 2f in $CDCl_3$ at 100 MHz, 298K

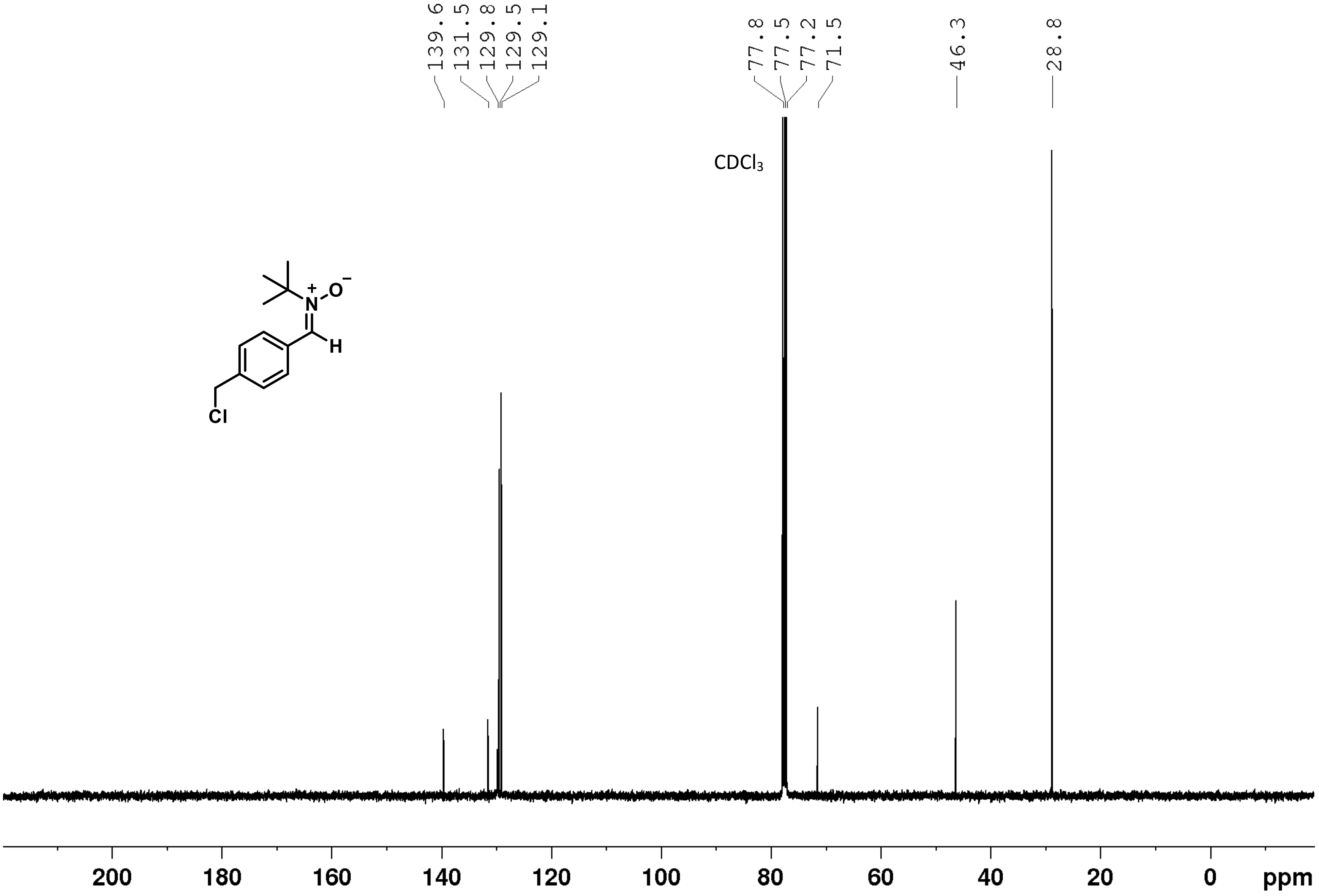

^{1}H-NMR of Compound 2g in $CDCl_3$ at 400 MHz, 298K

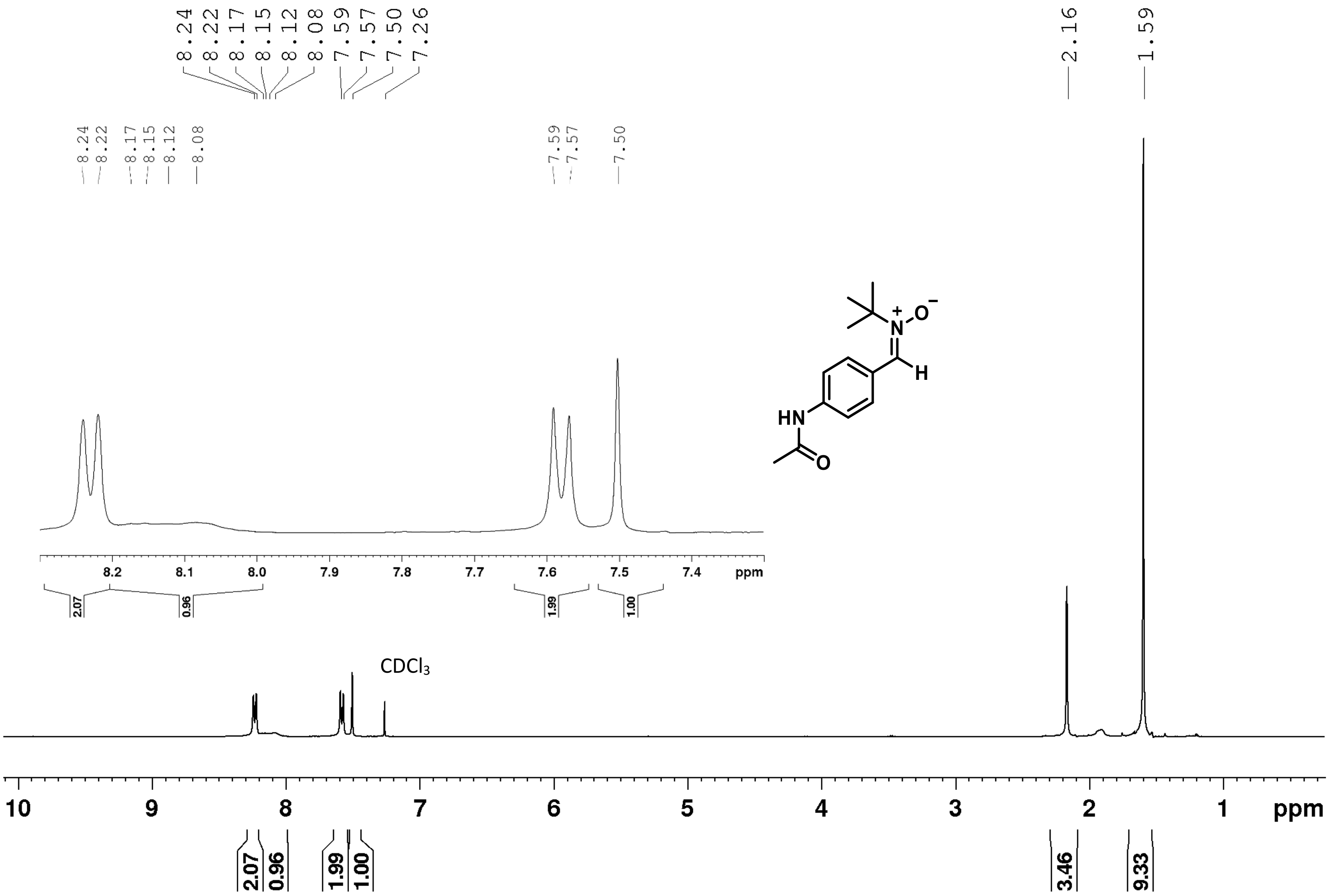

$^{13}C\{^{1}H\}$ NMR of Compound 2g in $CDCl_3$ at 100 MHz, 298K

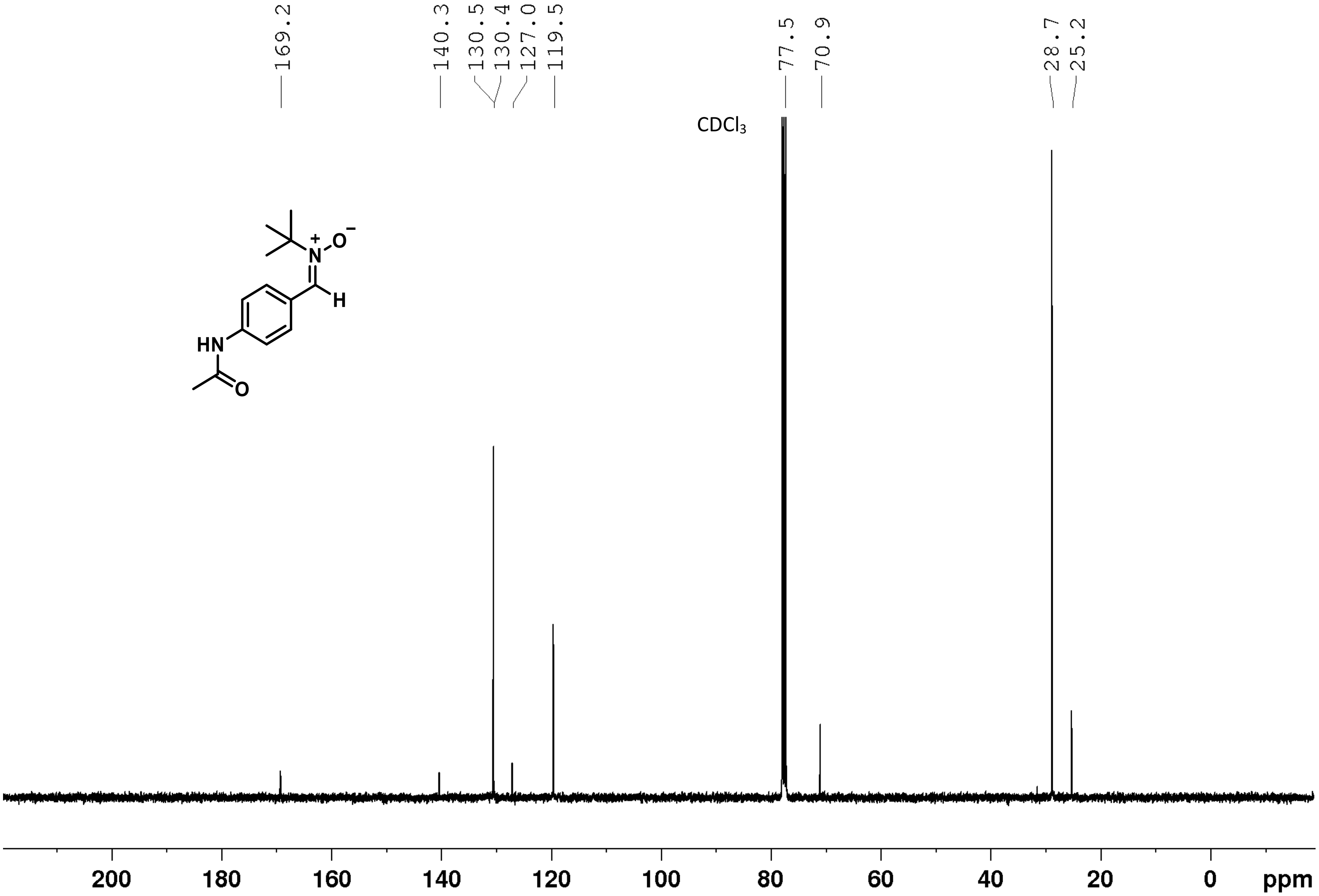

^{1}H-NMR of Compound 2h in $CDCl_3$ at 400 MHz, 298K

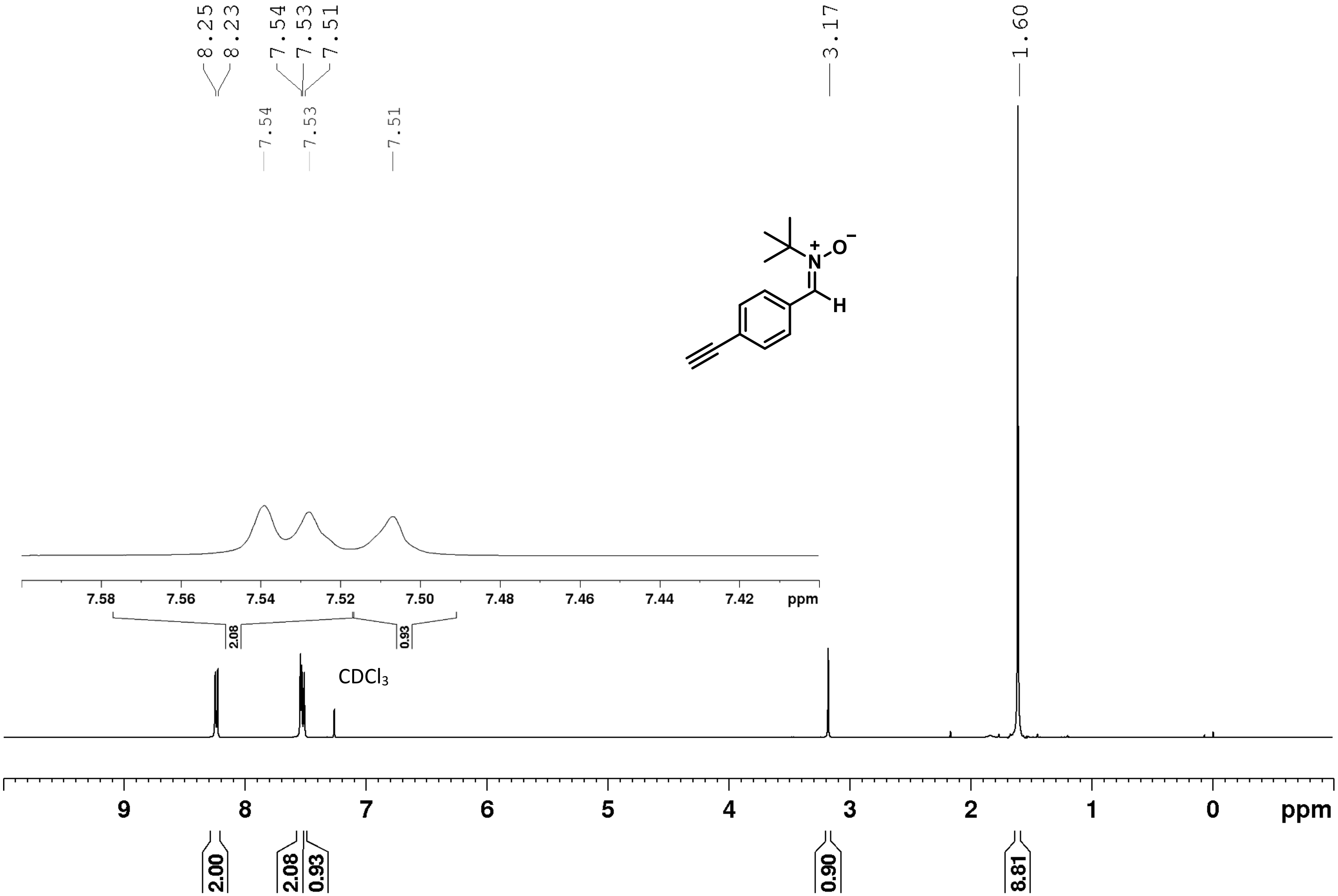

$^{13}C\{^{1}H\}$ NMR of Compound 2h in $CDCl_3$ at 100 MHz, 298K

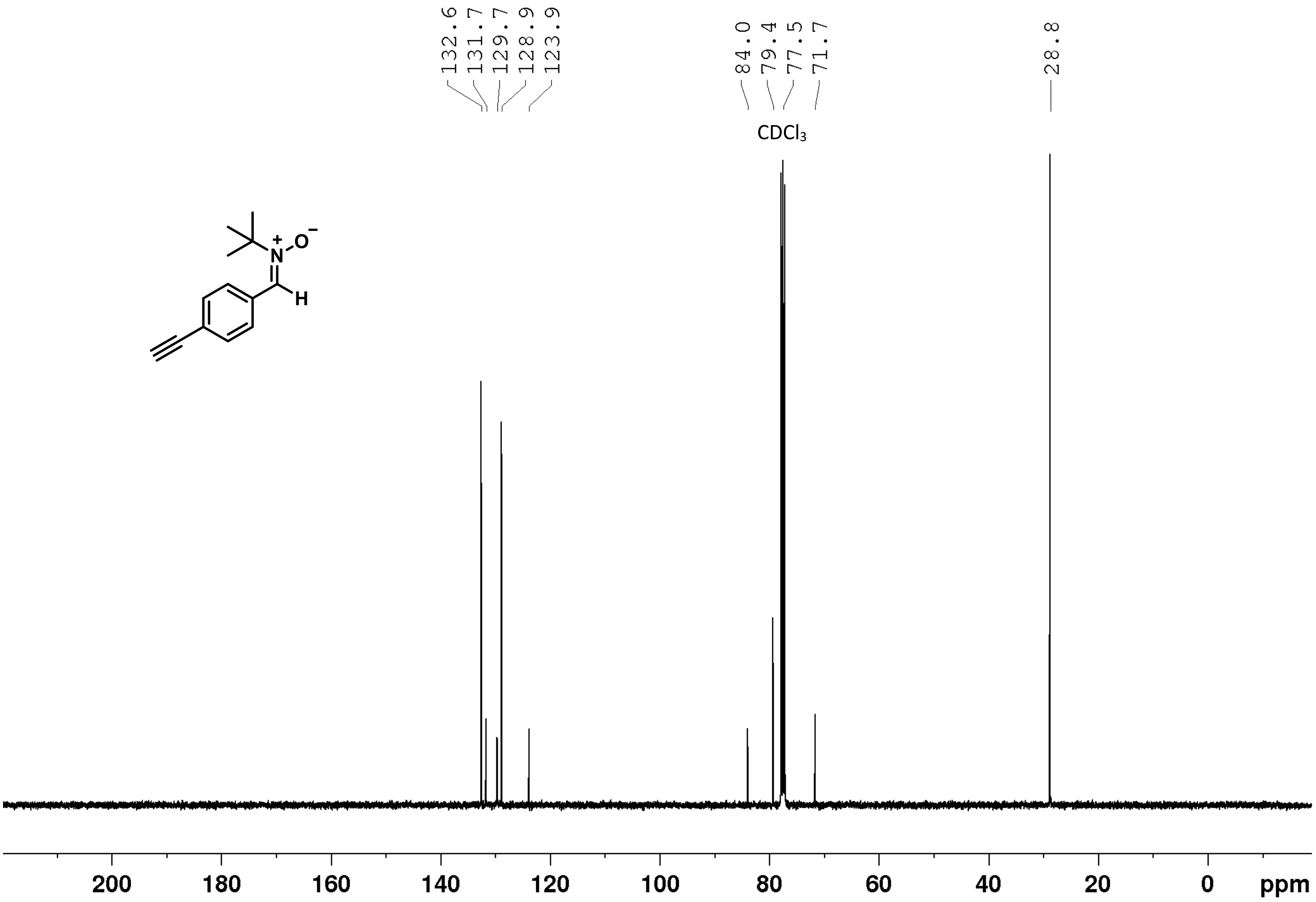

^{1}H-NMR of Compound 2i in $CDCl_3$ at 400 MHz, 298K

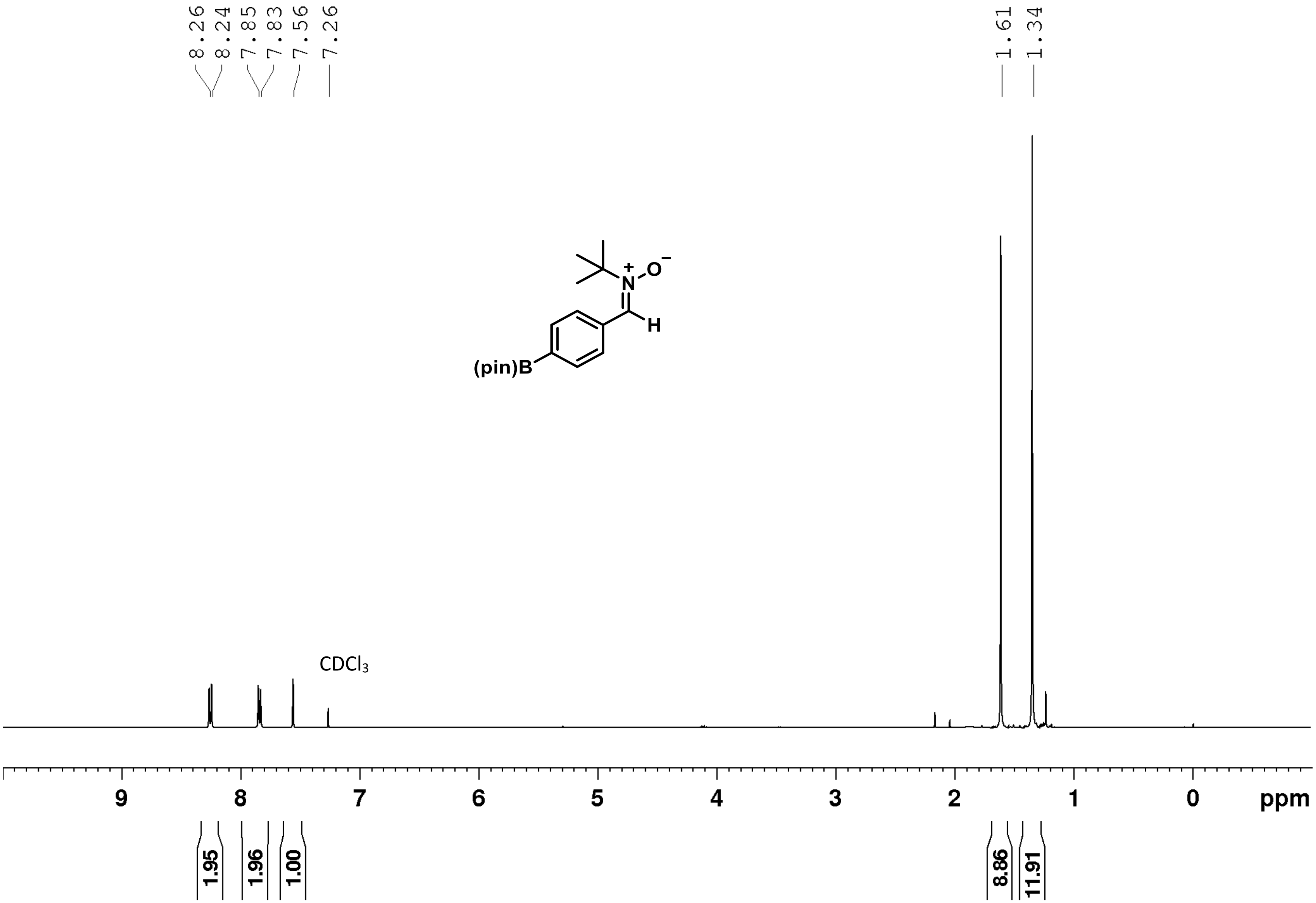

$^{13}C\{^{1}H\}$ NMR of Compound 2i in $CDCl_3$ at 100 MHz, 298K

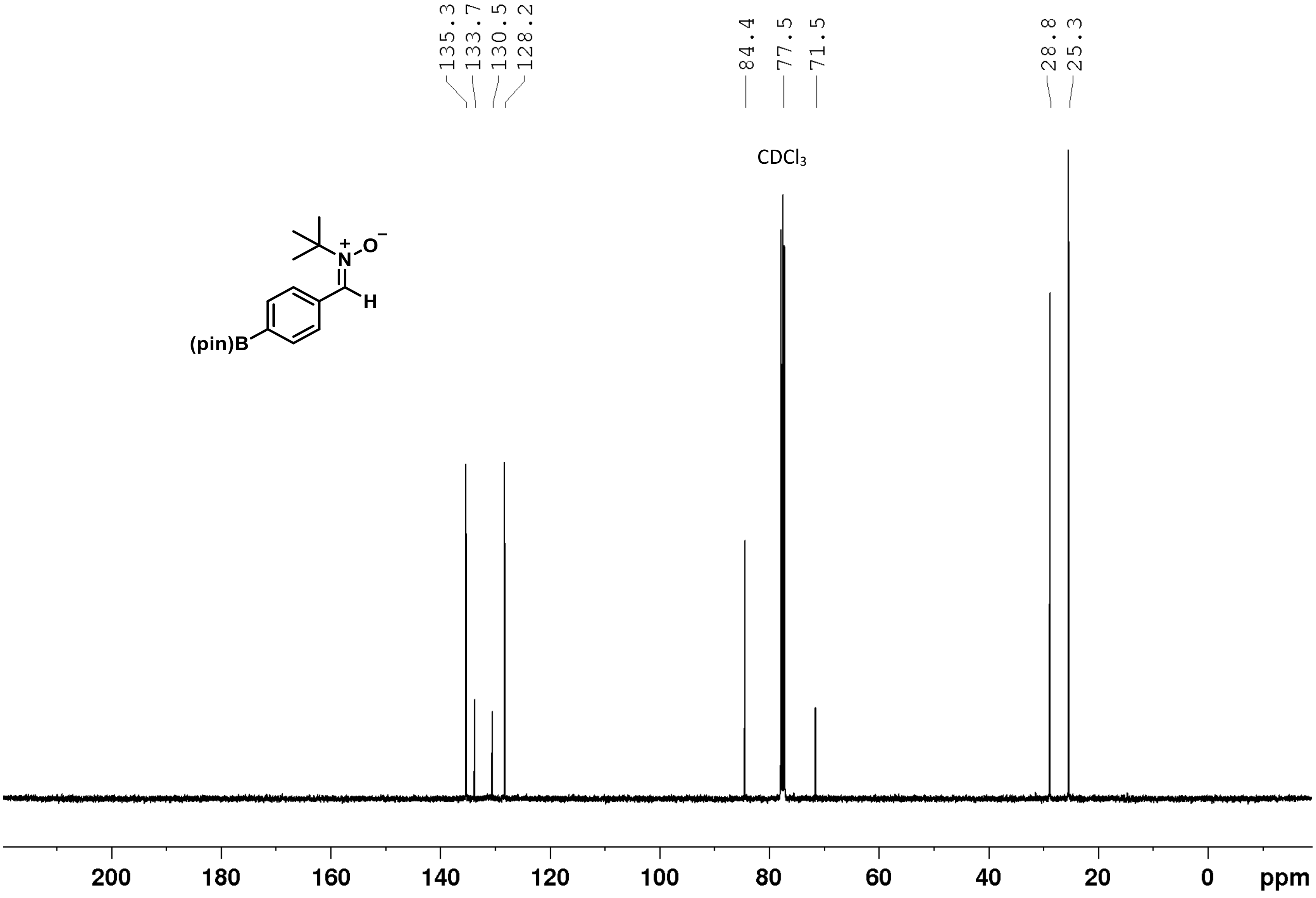

^{1}H-NMR of Compound 2j in $CDCl_3$ at 400 MHz, 298K

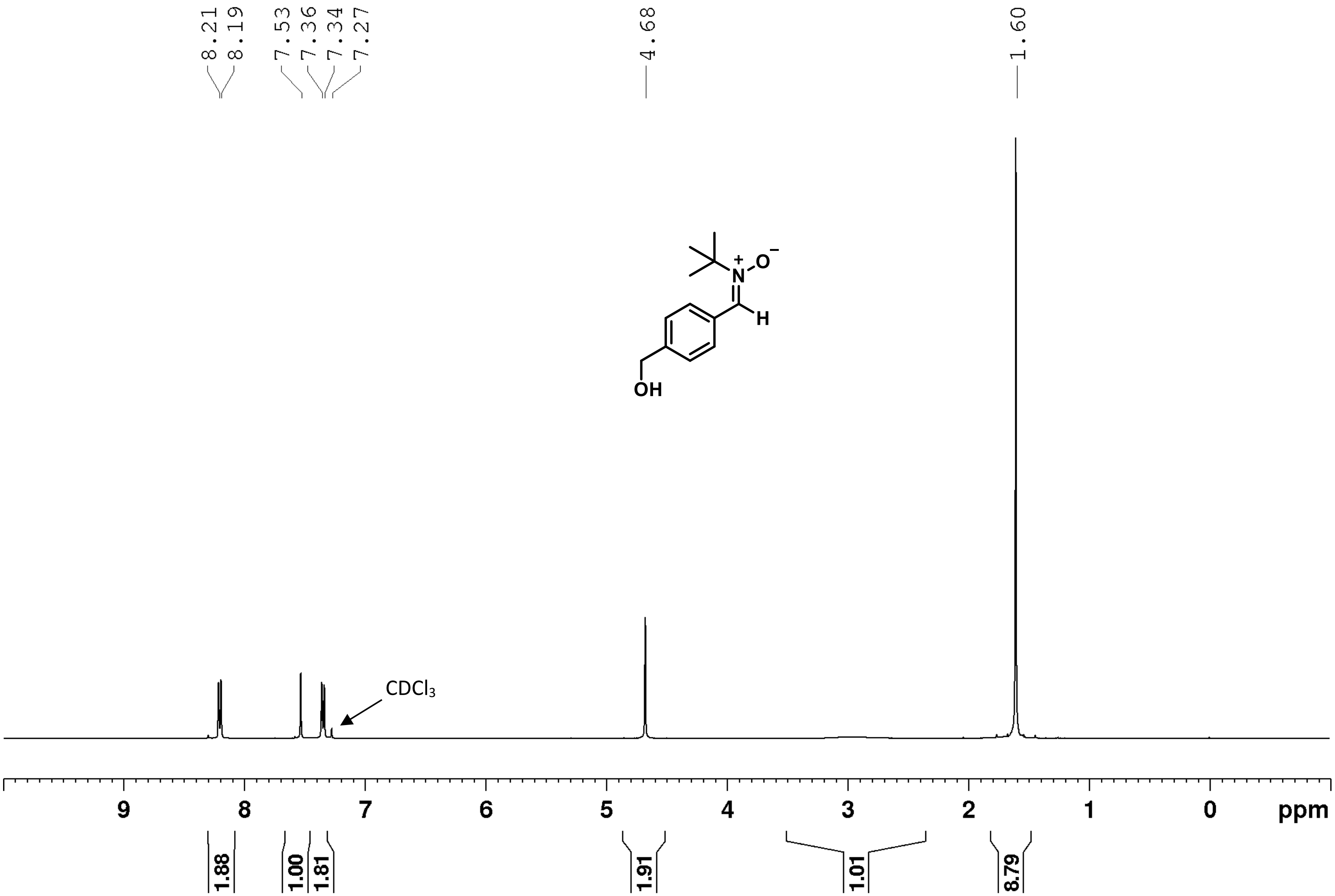

$^{13}C\{^{1}H\}$ NMR of Compound 2j in $CDCl_3$ at 100 MHz, 298K

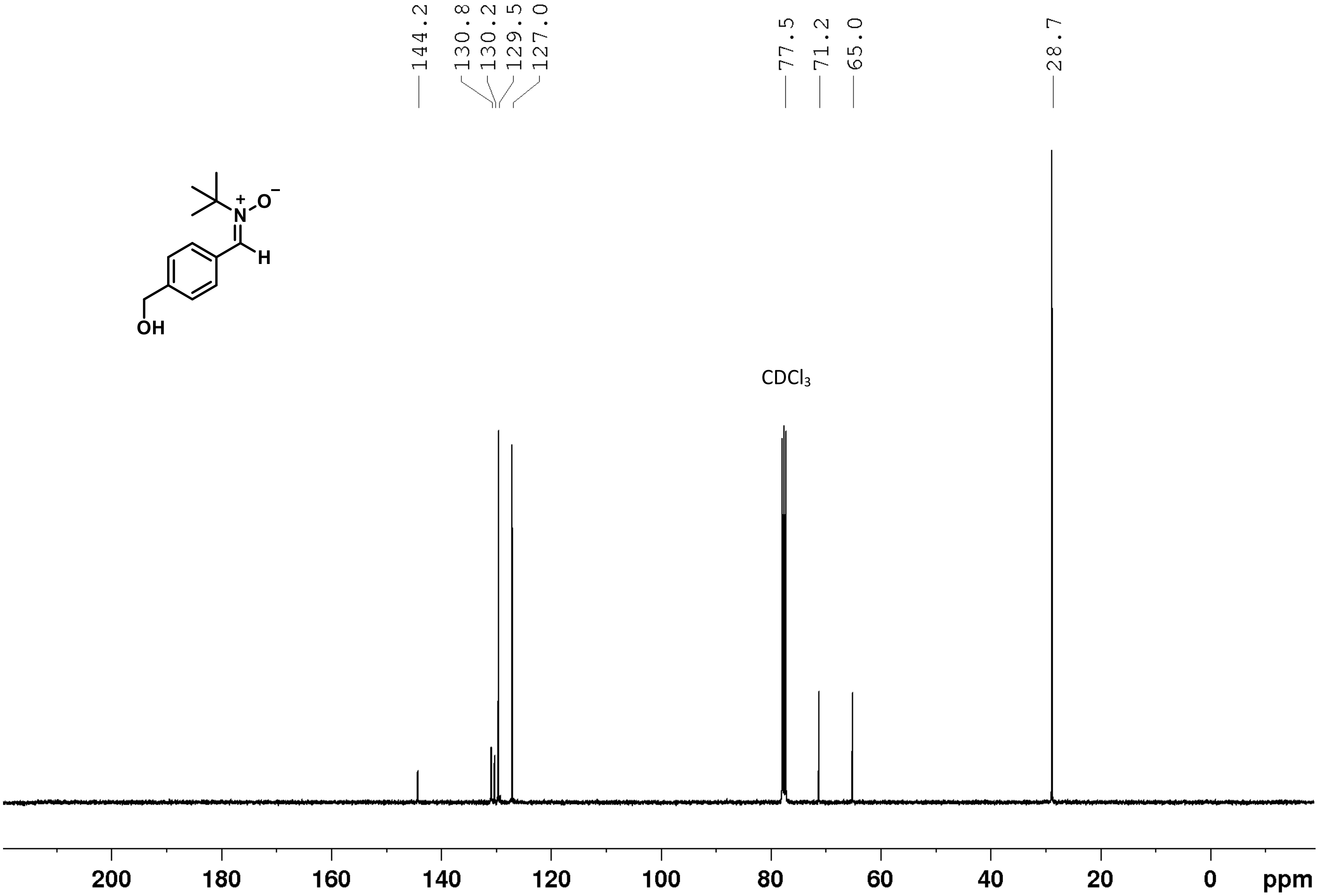

^{1}H-NMR of Compound 2l in $CDCl_3$ at 400 MHz, 298K

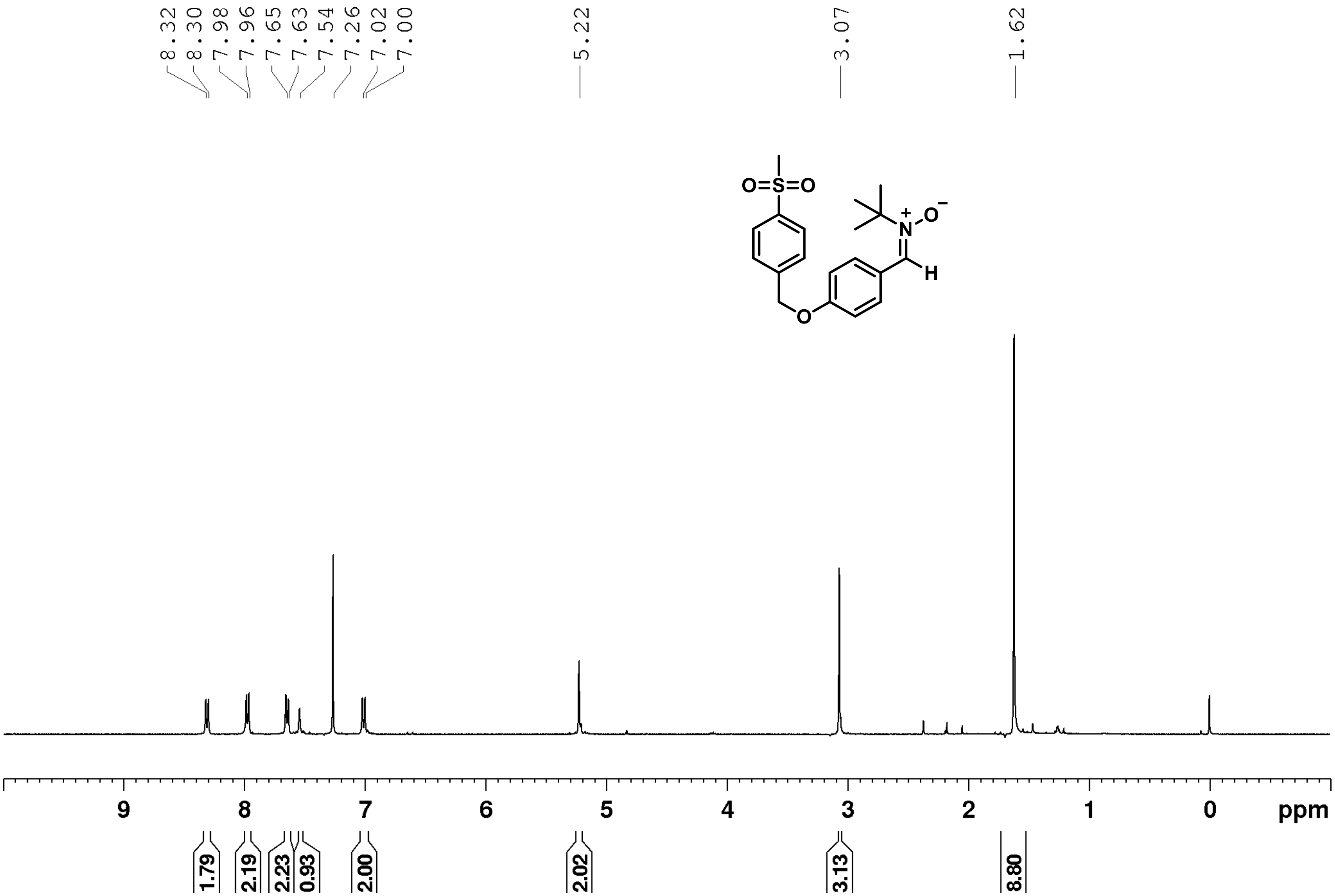

$^{13}C\{^{1}H\}$ NMR of Compound 2l in $CDCl_3$ at 100 MHz, 298K

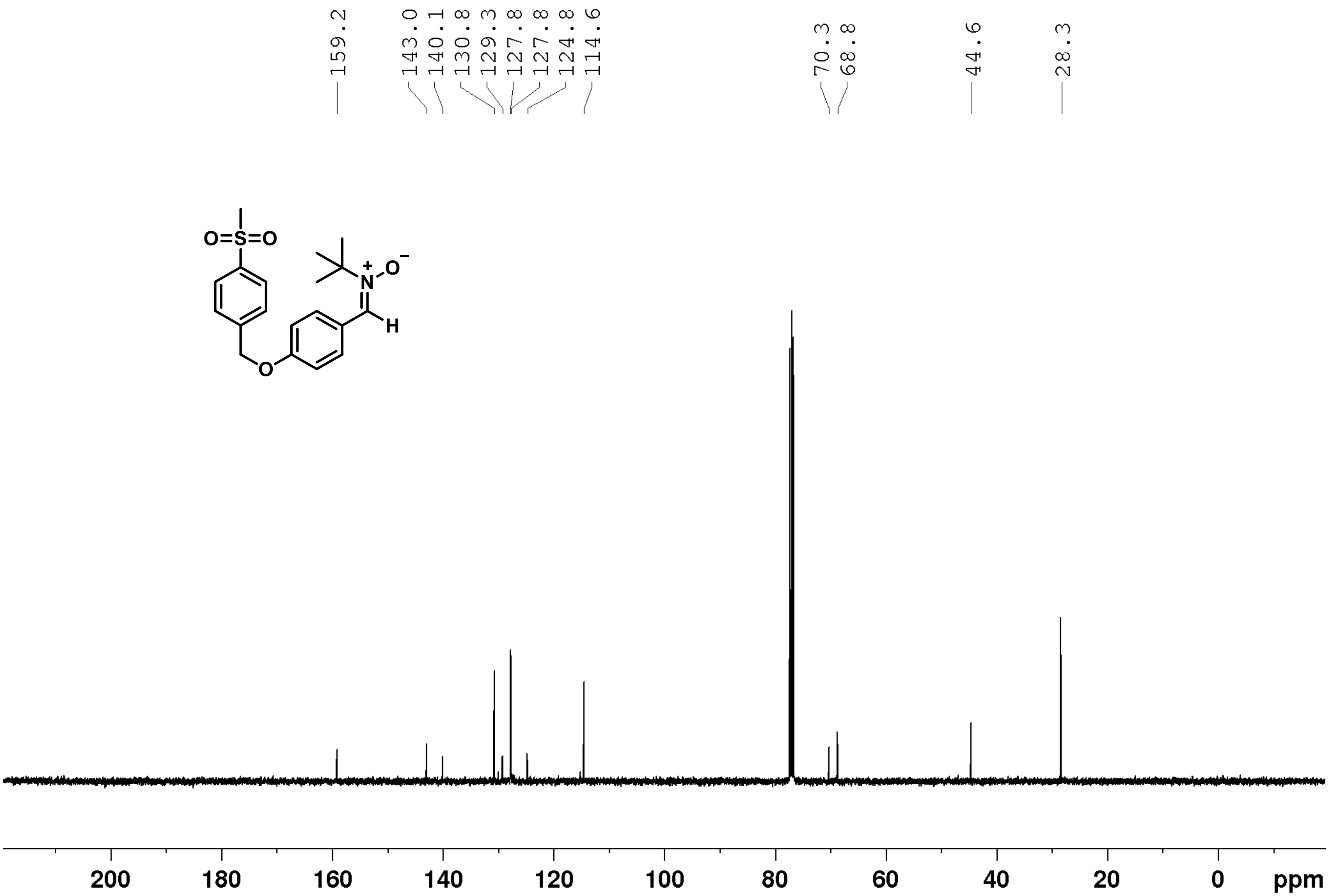

^{1}H-NMR of Compound 2k in $CDCl_3$ at 400 MHz, 298K

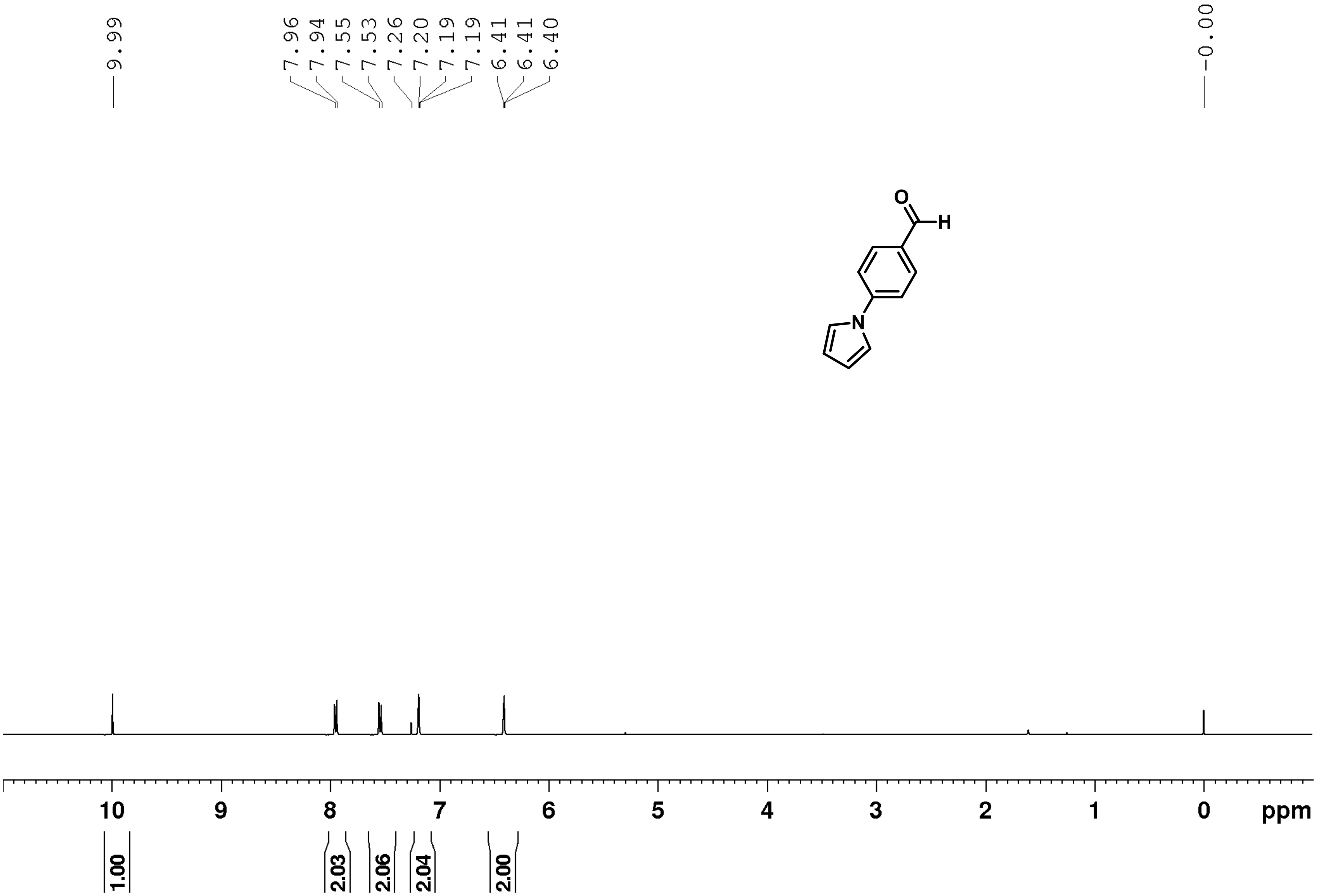

$^{13}C\{^{1}H\}$ NMR of Compound 2k in $CDCl_3$ at 100 MHz, 298K

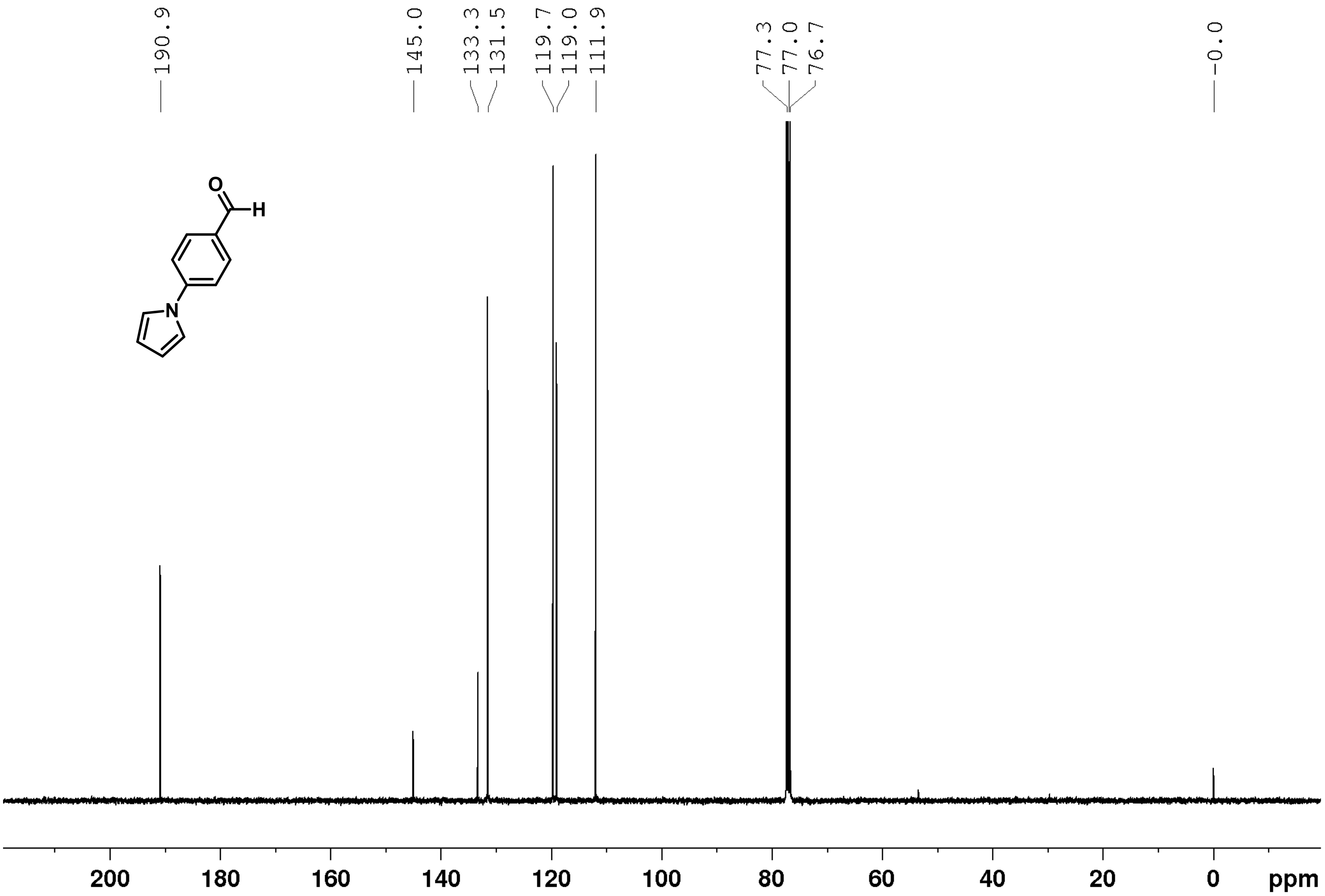

^{1}H-NMR of Compound 3aa in $CDCl_3$ at 400 MHz, 298K

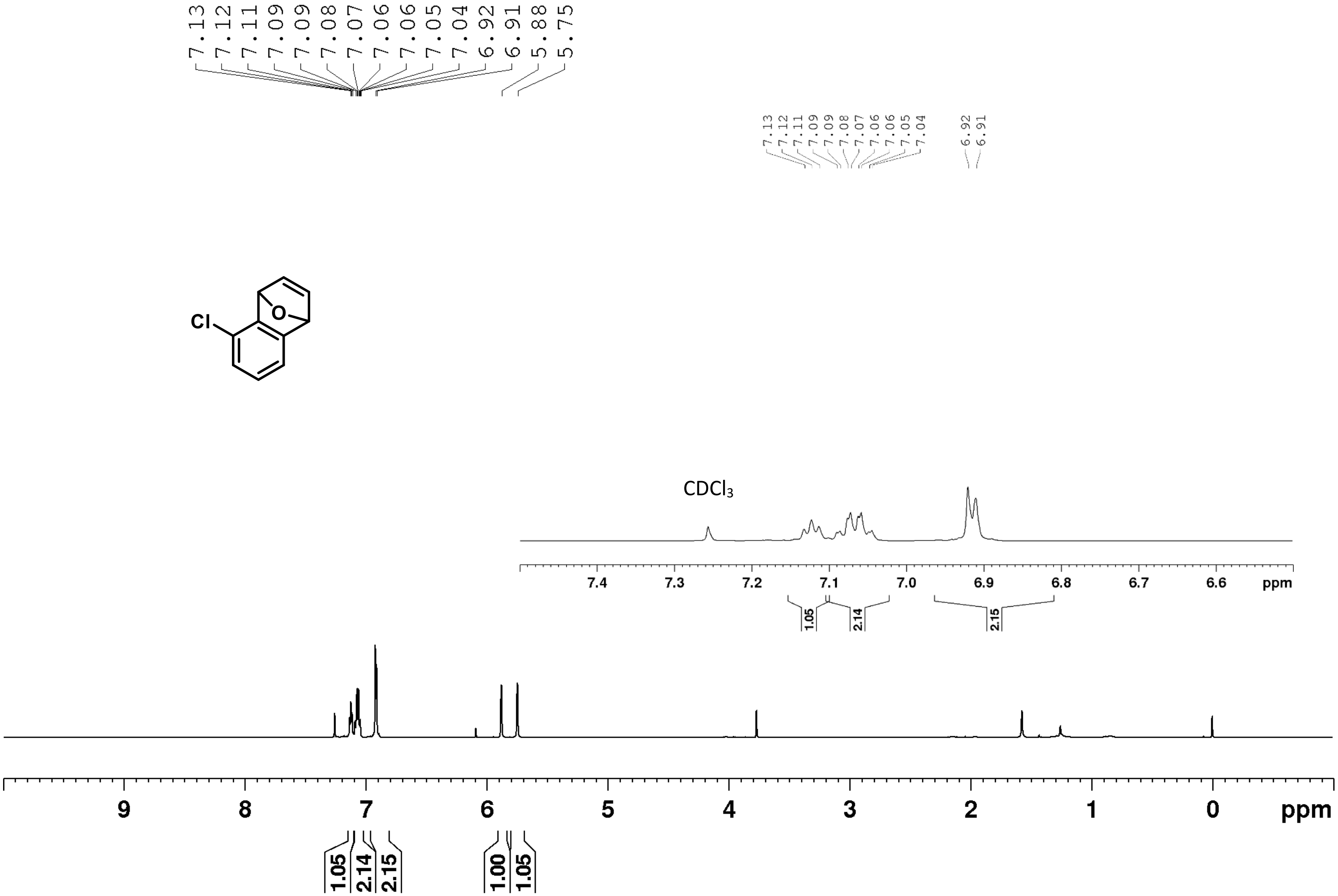

^{13}C-NMR of Compound 3a in $CDCl_3$ at 101 MHz, 298K

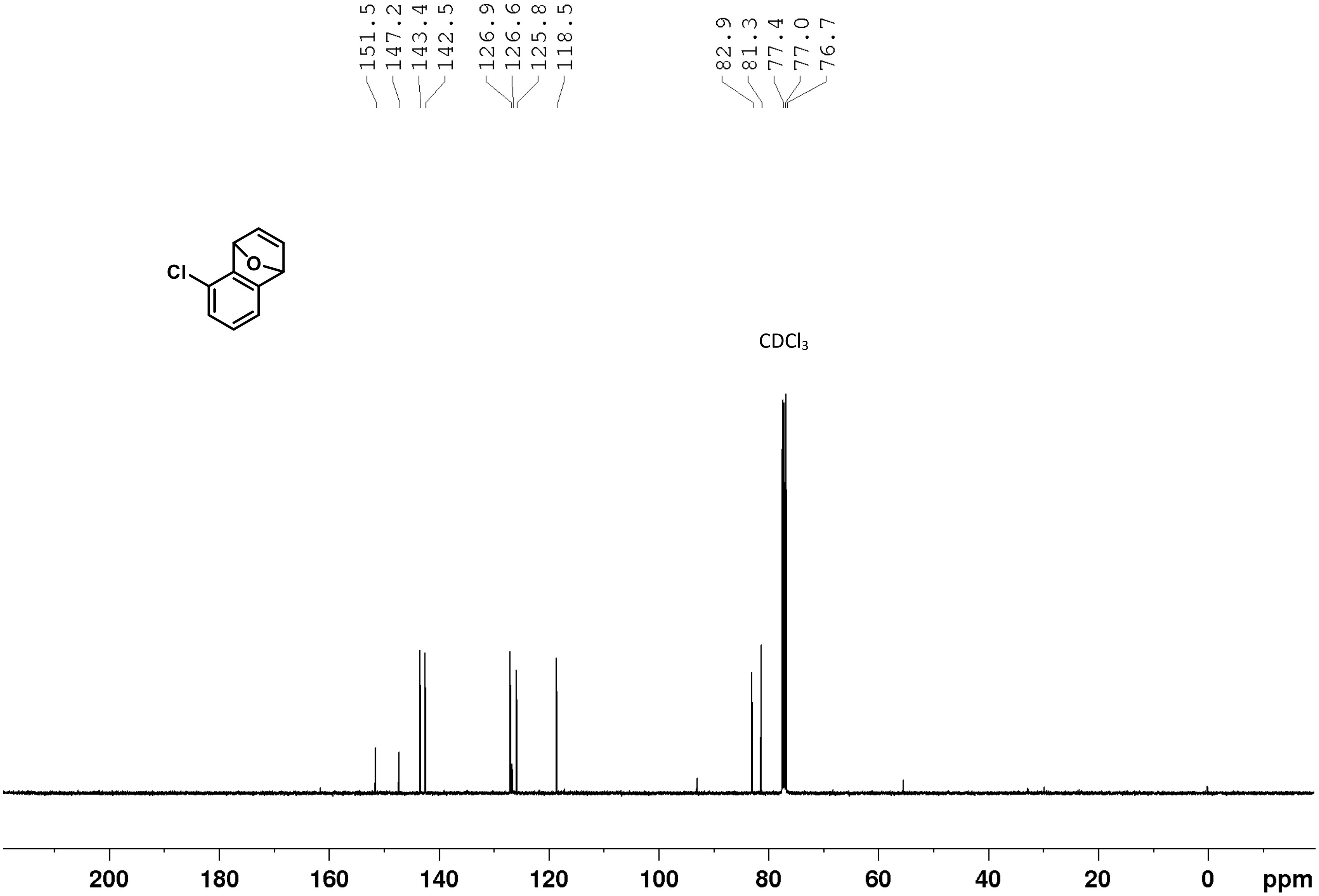

^{1}H-NMR of Compound 3ab in $CDCl_3$ at 400 MHz, 298K

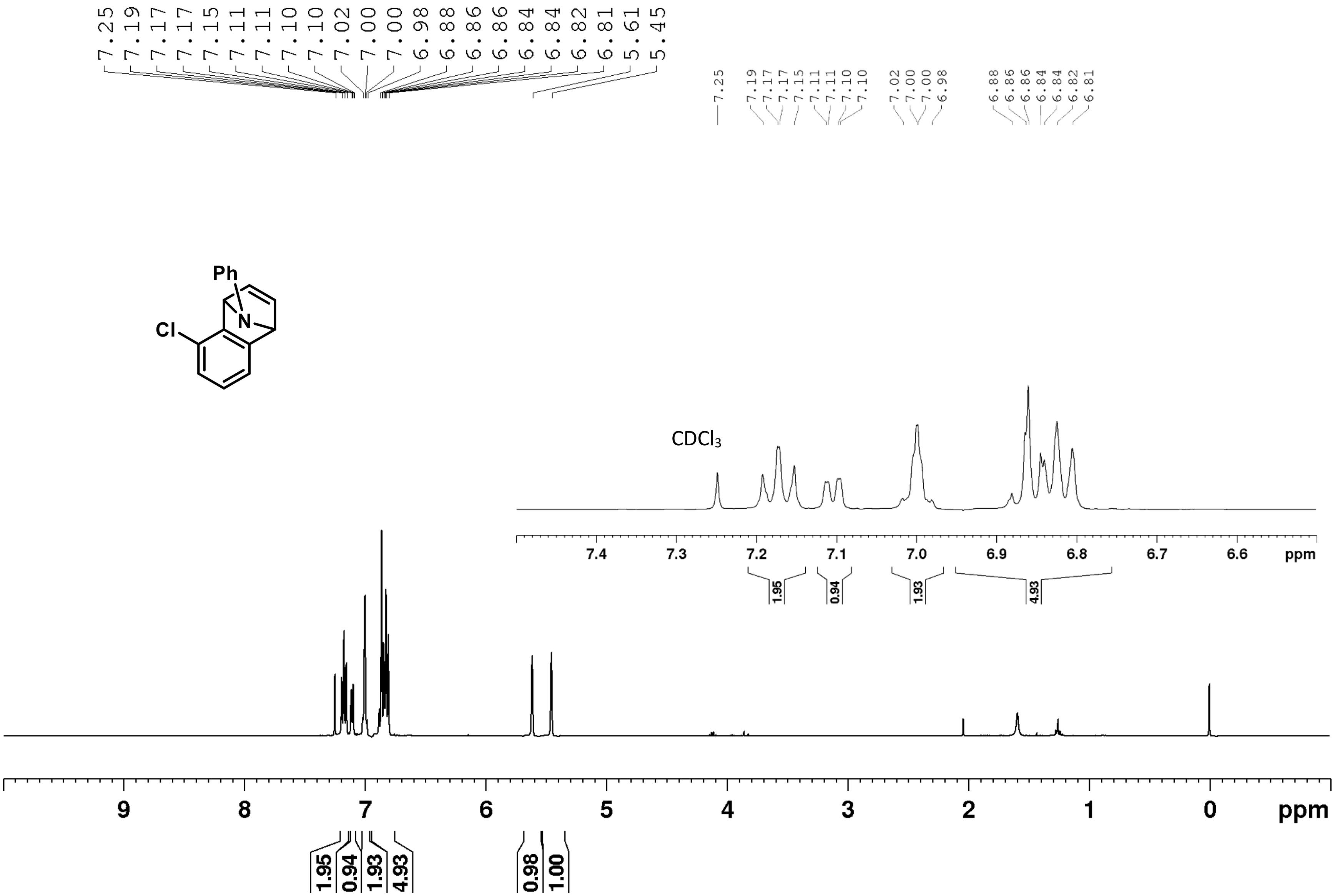

^{13}C-NMR of Compound 3ab in $CDCl_3$ at 101 MHz, 298K

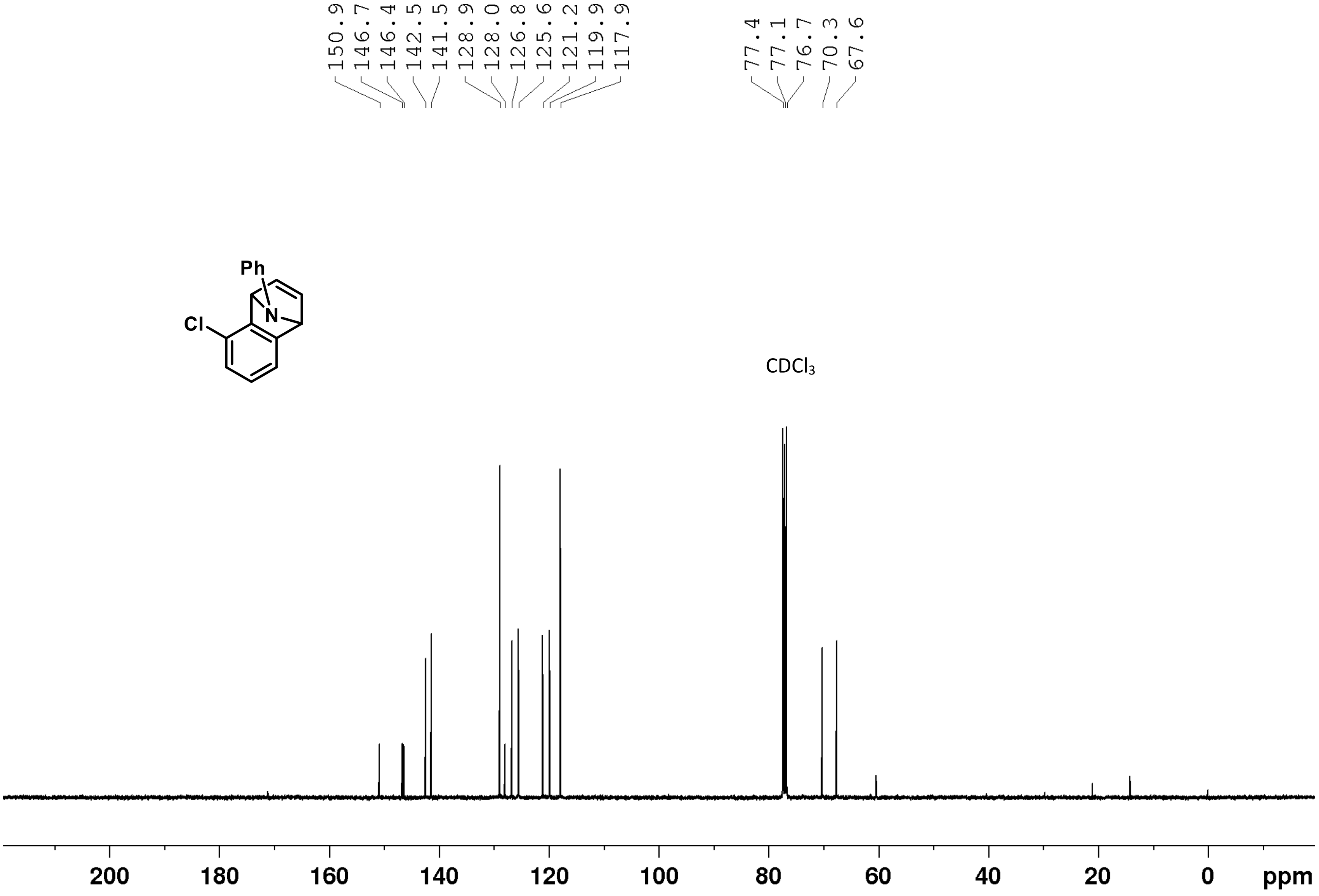

^{1}H-NMR of Compound 3ac in $CDCl_3$ at 400 MHz, 298K

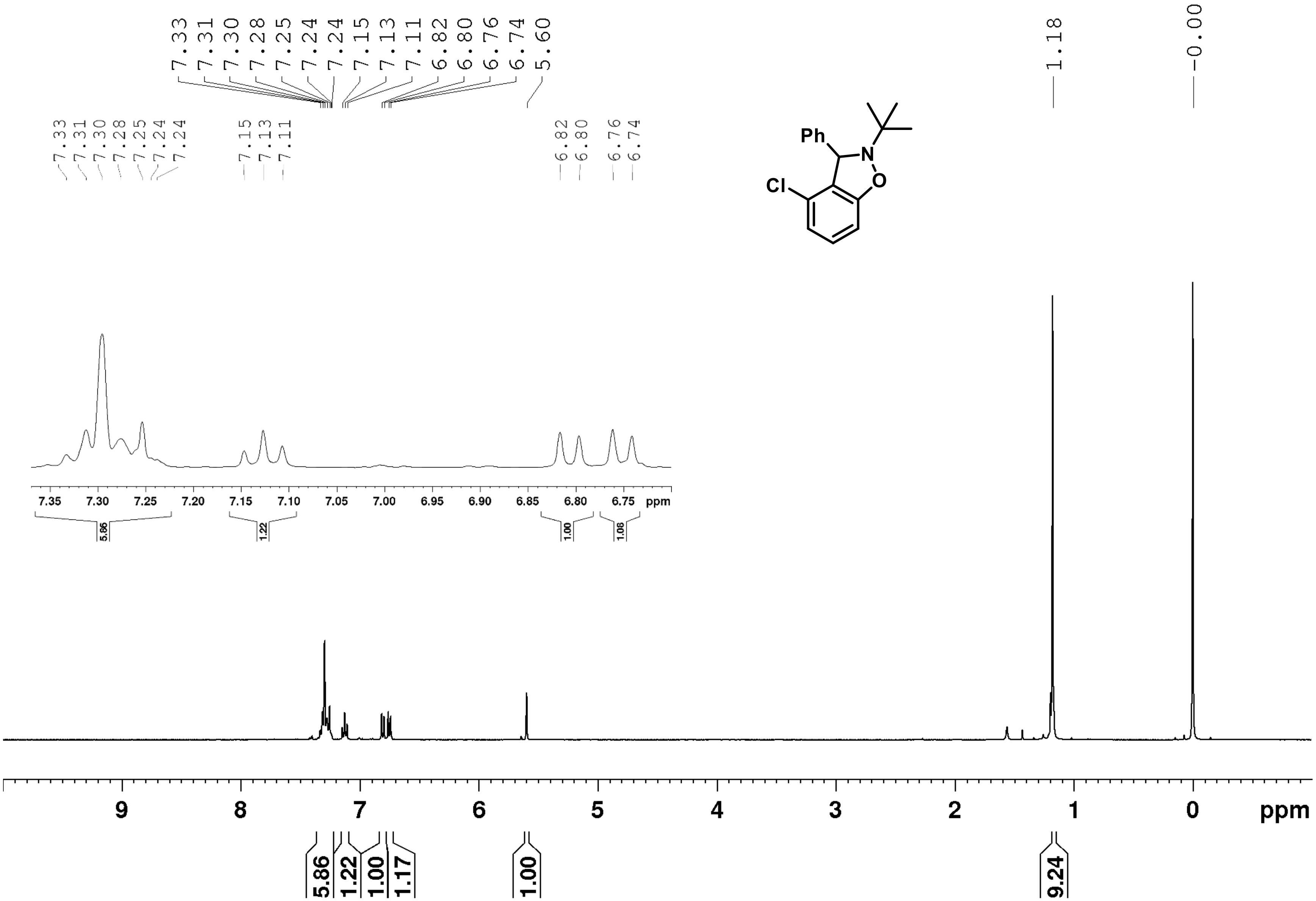

$^{13}C\{^{1}H\}$ NMR of Compound 3ac in $CDCl_3$ at 100 MHz, 298K

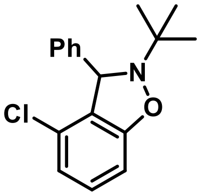

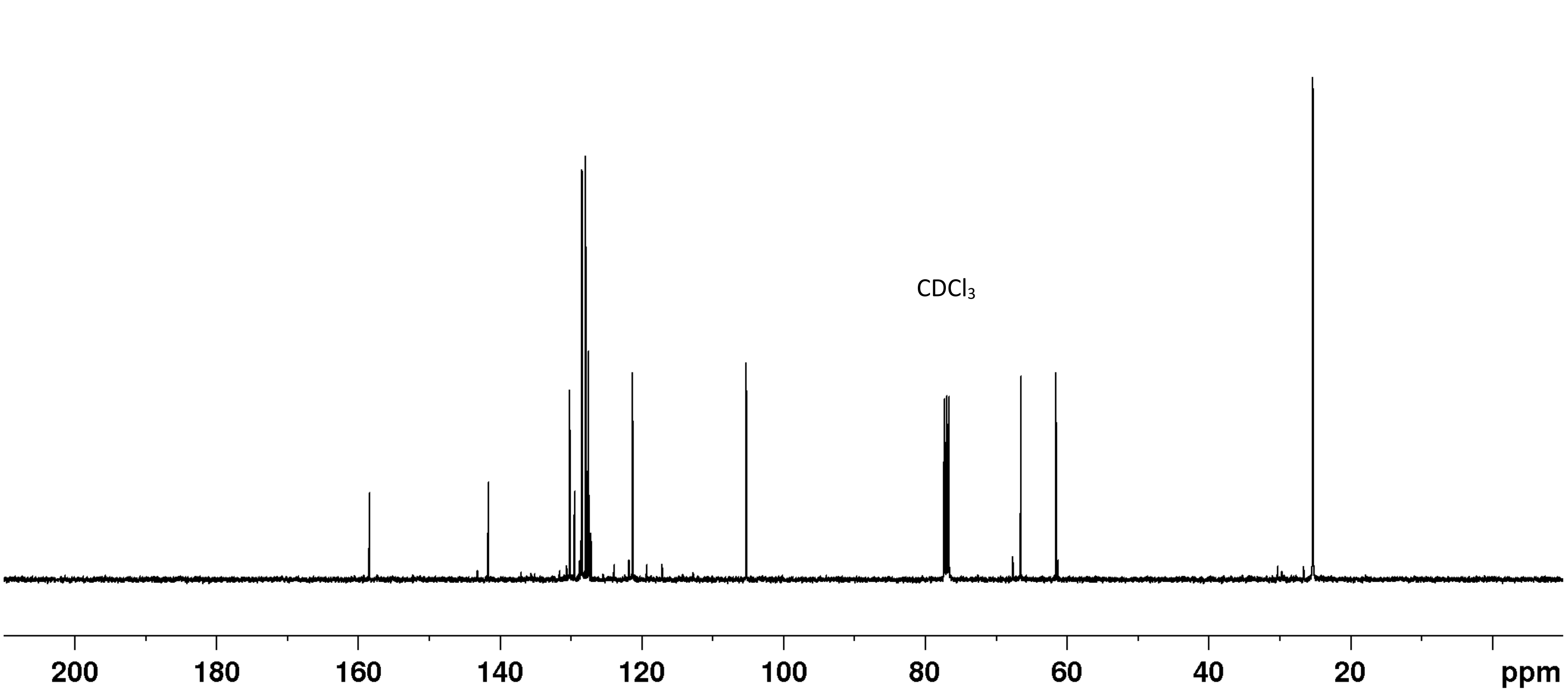

^{1}H-NMR of Compound 3ad in $CDCl_3$ at 400 MHz, 298K

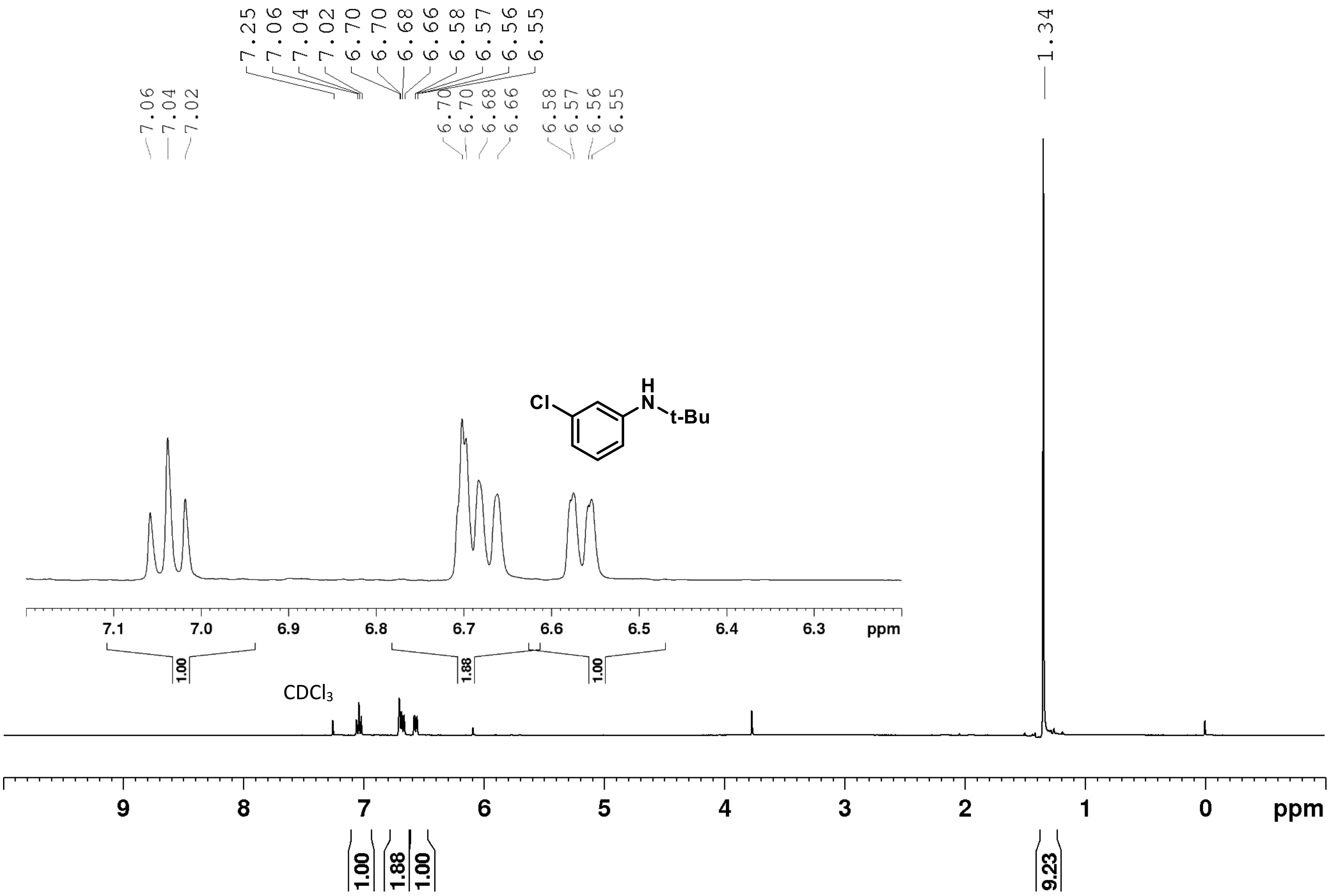

^{13}C-NMR of Compound 3ad in $CDCl_3$ at 101 MHz, 298K

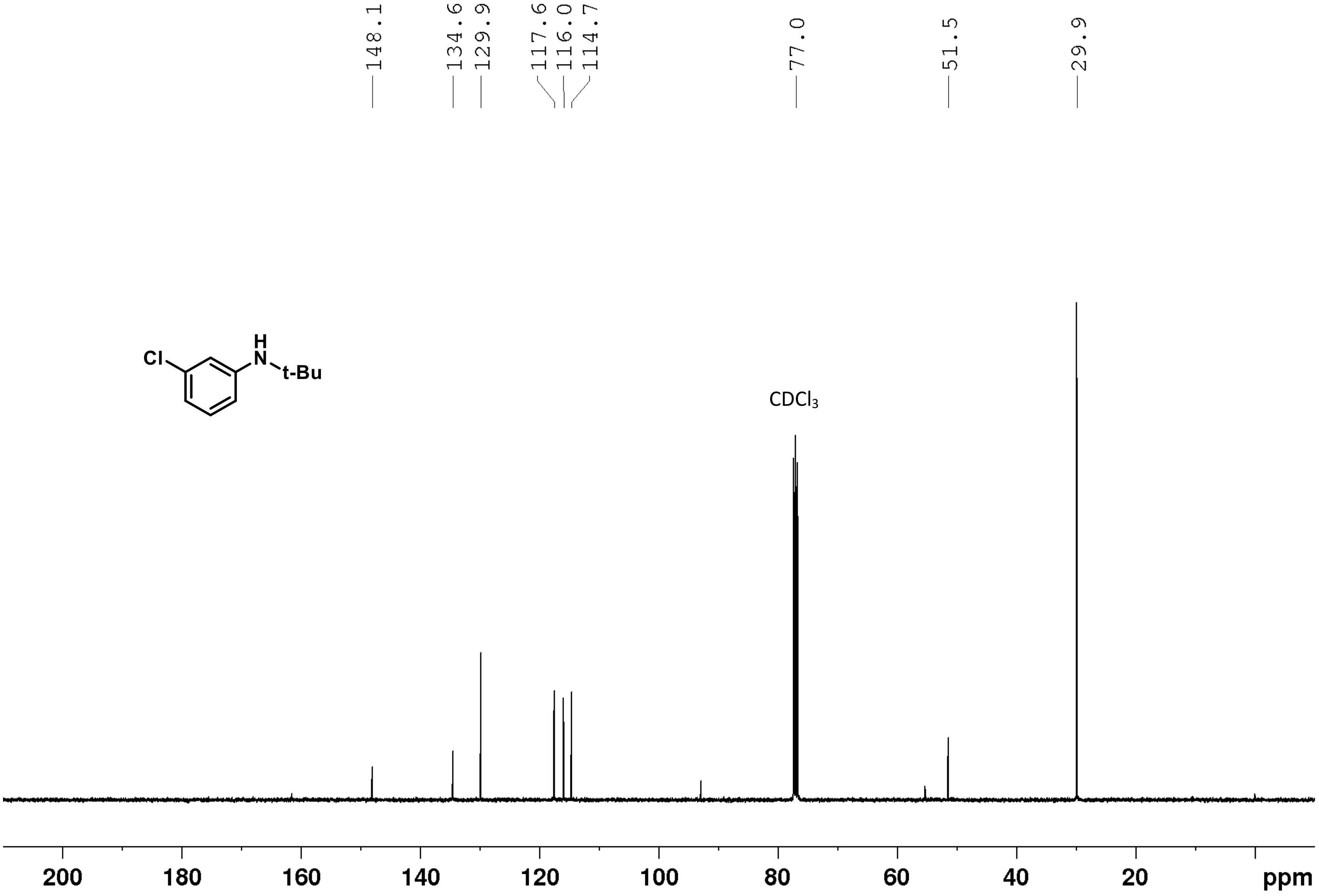

^{1}H-NMR of Compound 3ae in $CDCl_3$ at 400 MHz, 298K

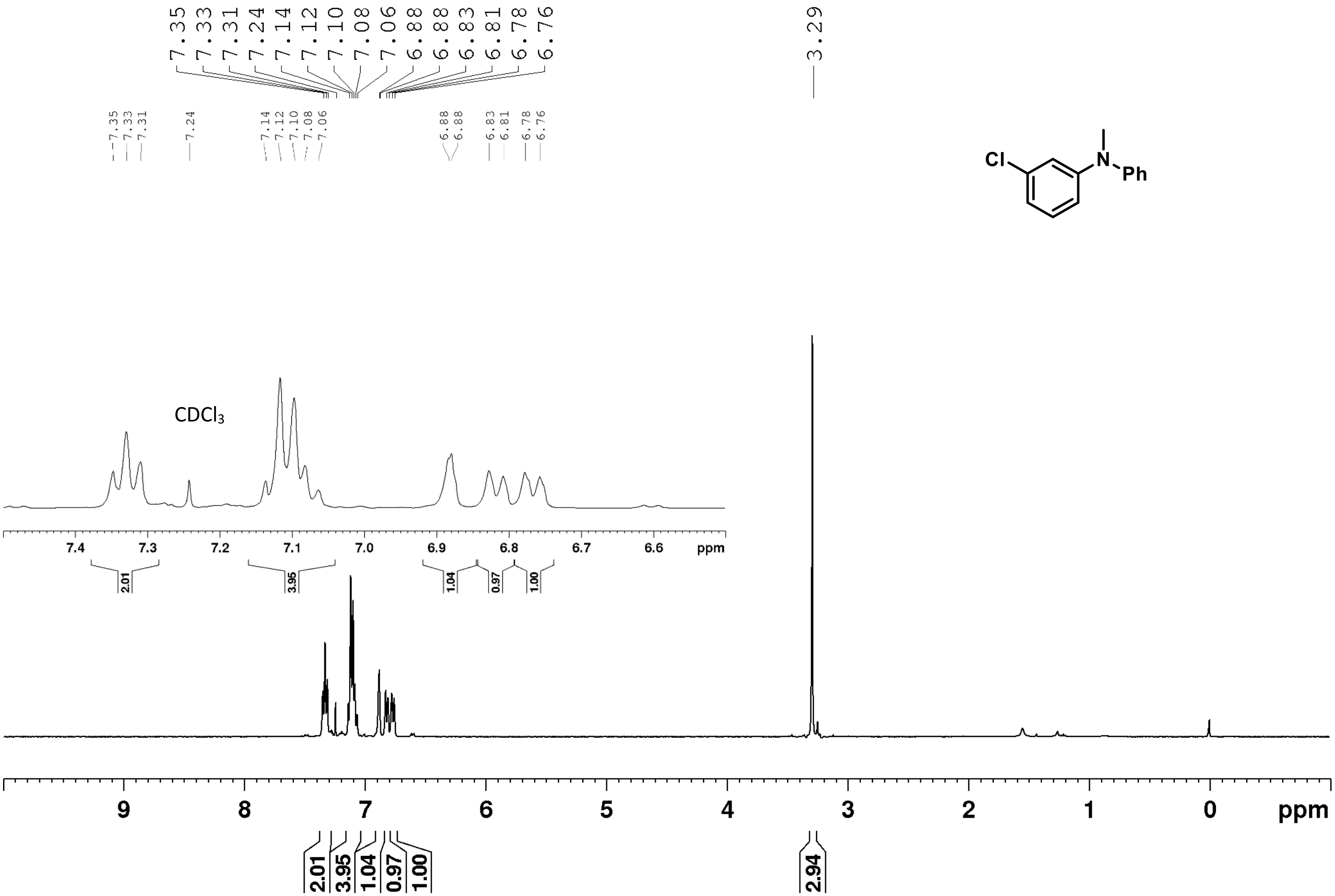

$^{13}C\{^1H\}$-NMR of Compound 3ae in $CDCl_3$ at 400 MHz, 298K

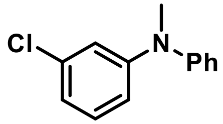

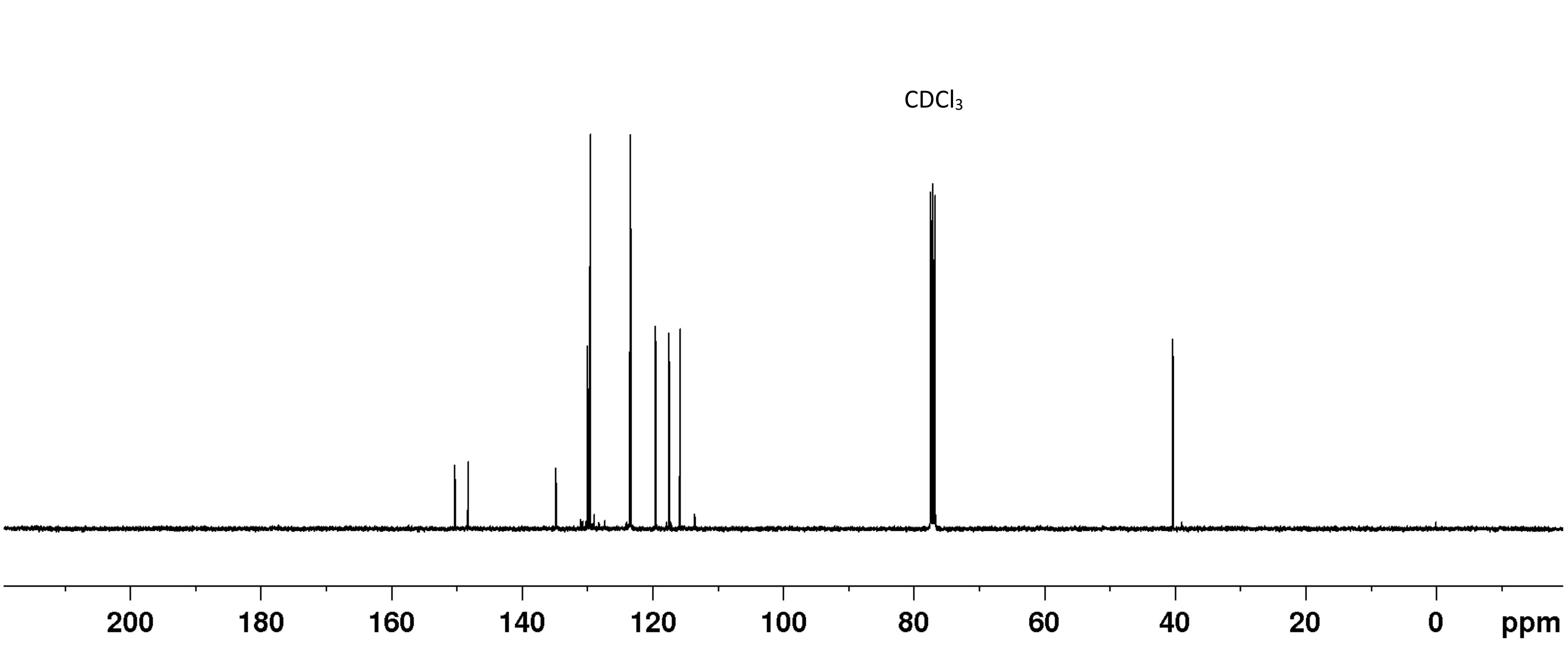

^{1}H-NMR of Compound 3bc in $CDCl_3$ at 400 MHz, 298K

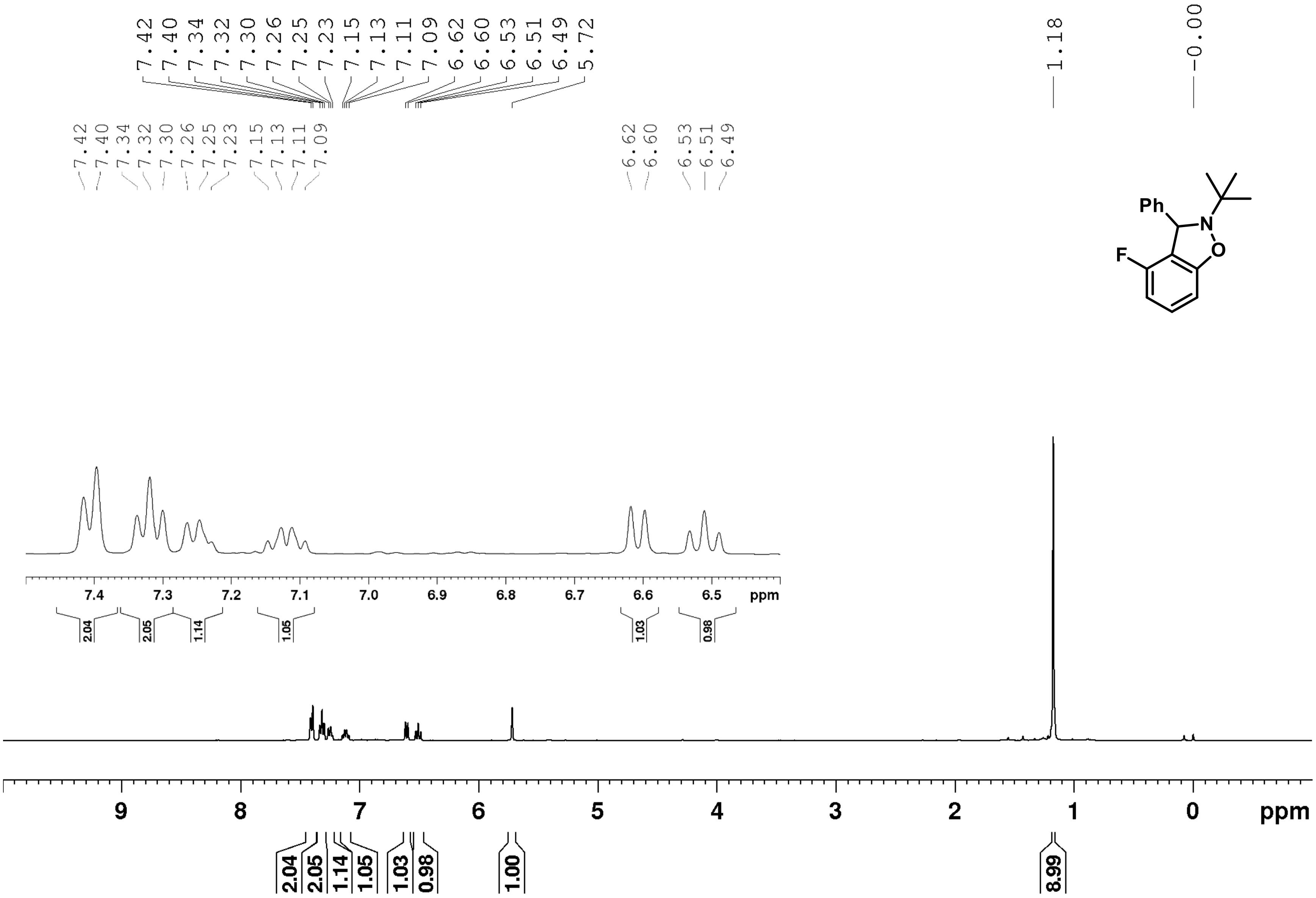

$^{13}C\{^1H\}$ NMR of Compound 3bc in $CDCl_3$ at 100 MHz, 298K

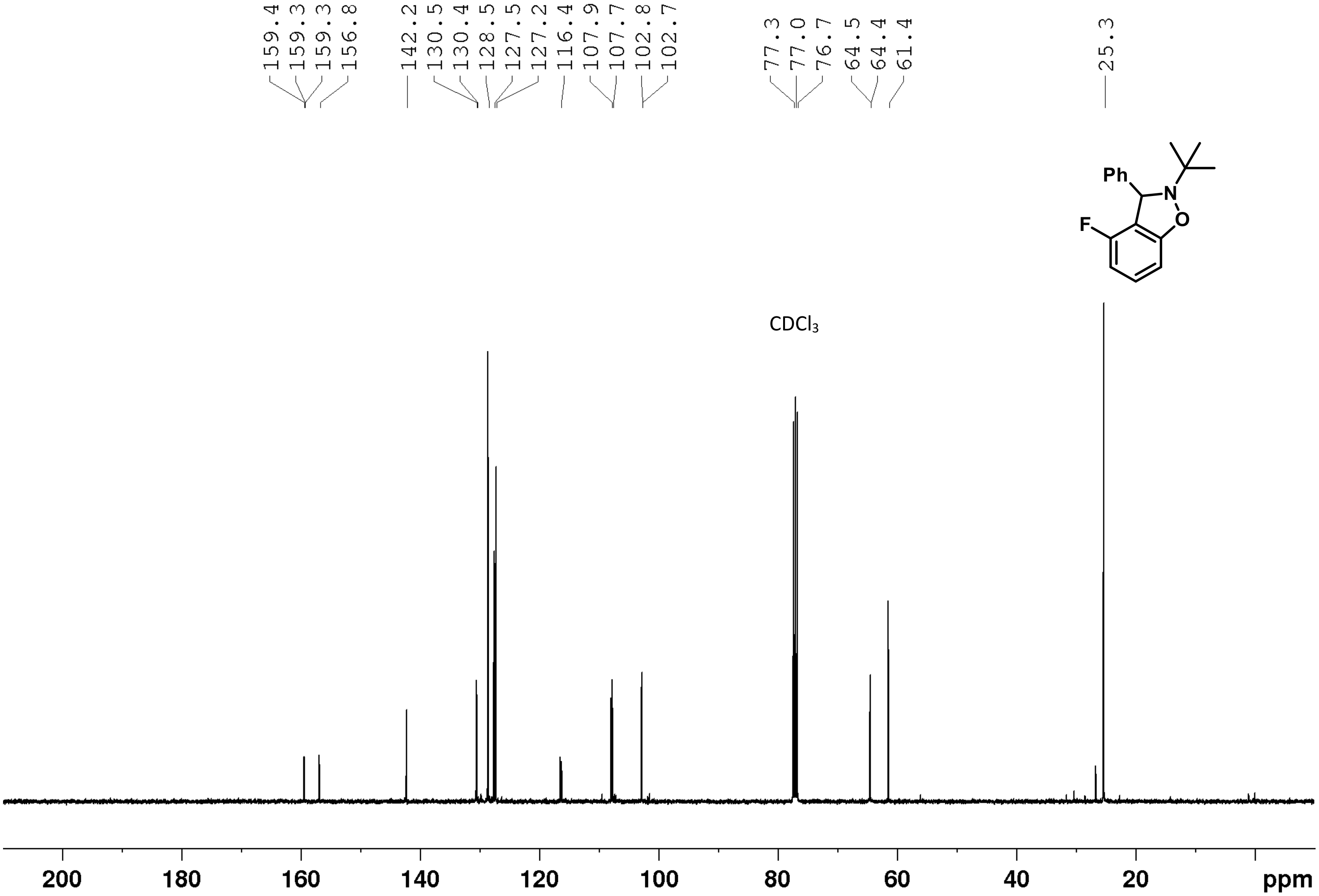

$^{19}F\{^{1}H\}$ NMR of compound 3bc in $CDCl_3$ at 376 MHz, 298K

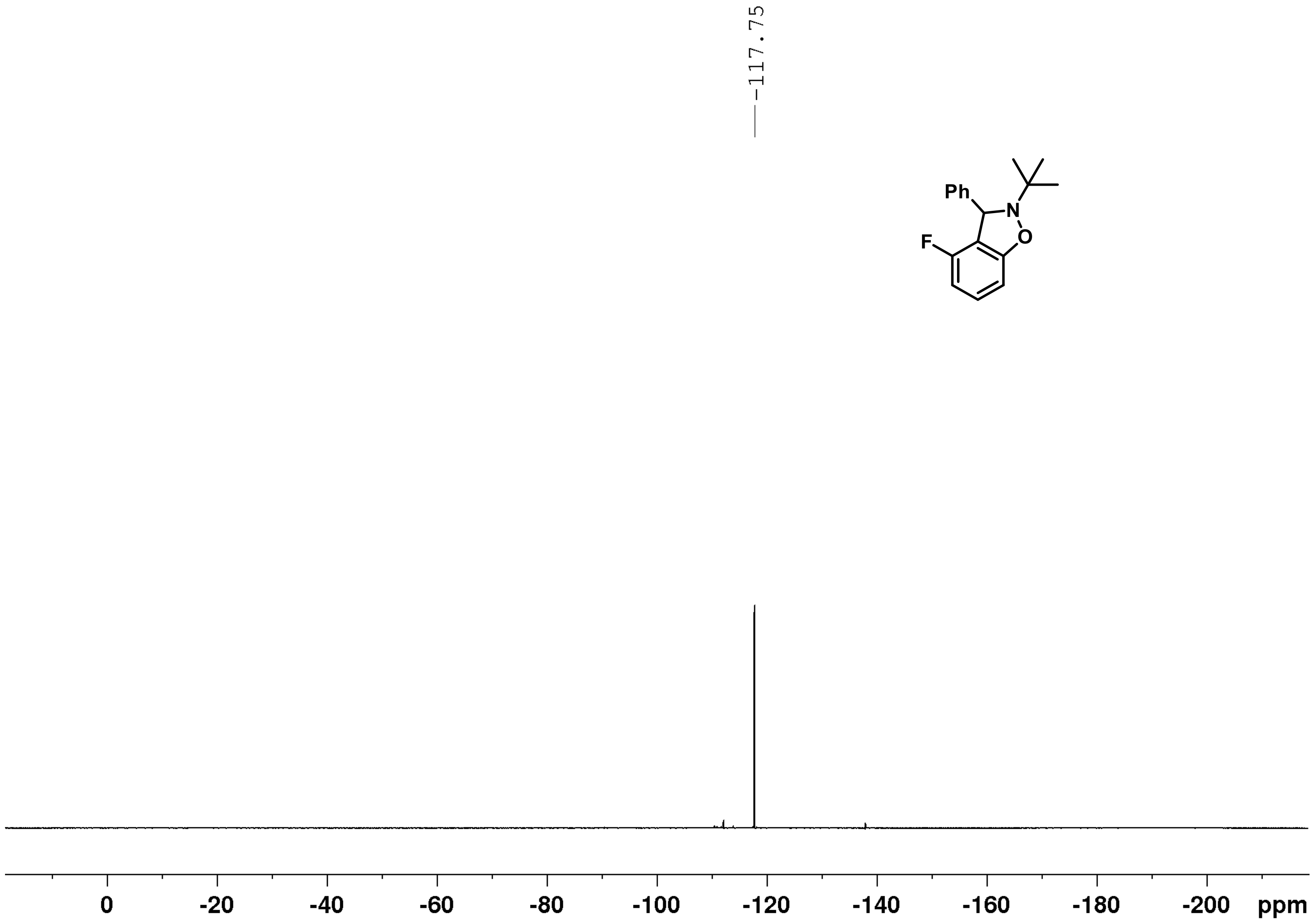

^{1}H-NMR of Compound 3cc in $CDCl_3$ at 400 MHz, 298K

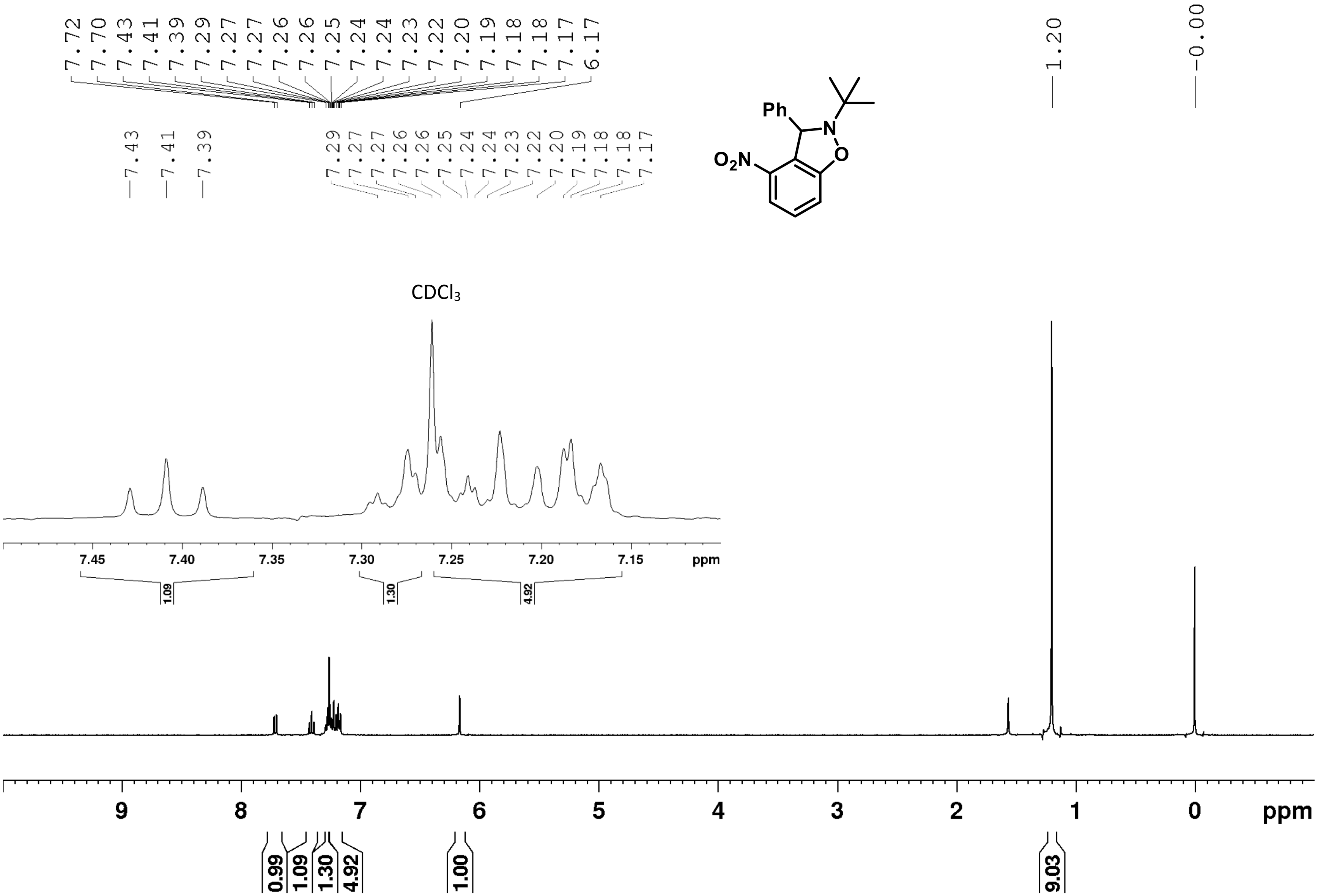

$^{13}C\{^1H\}$ NMR of Compound 3cc in $CDCl_3$ at 100 MHz, 298K

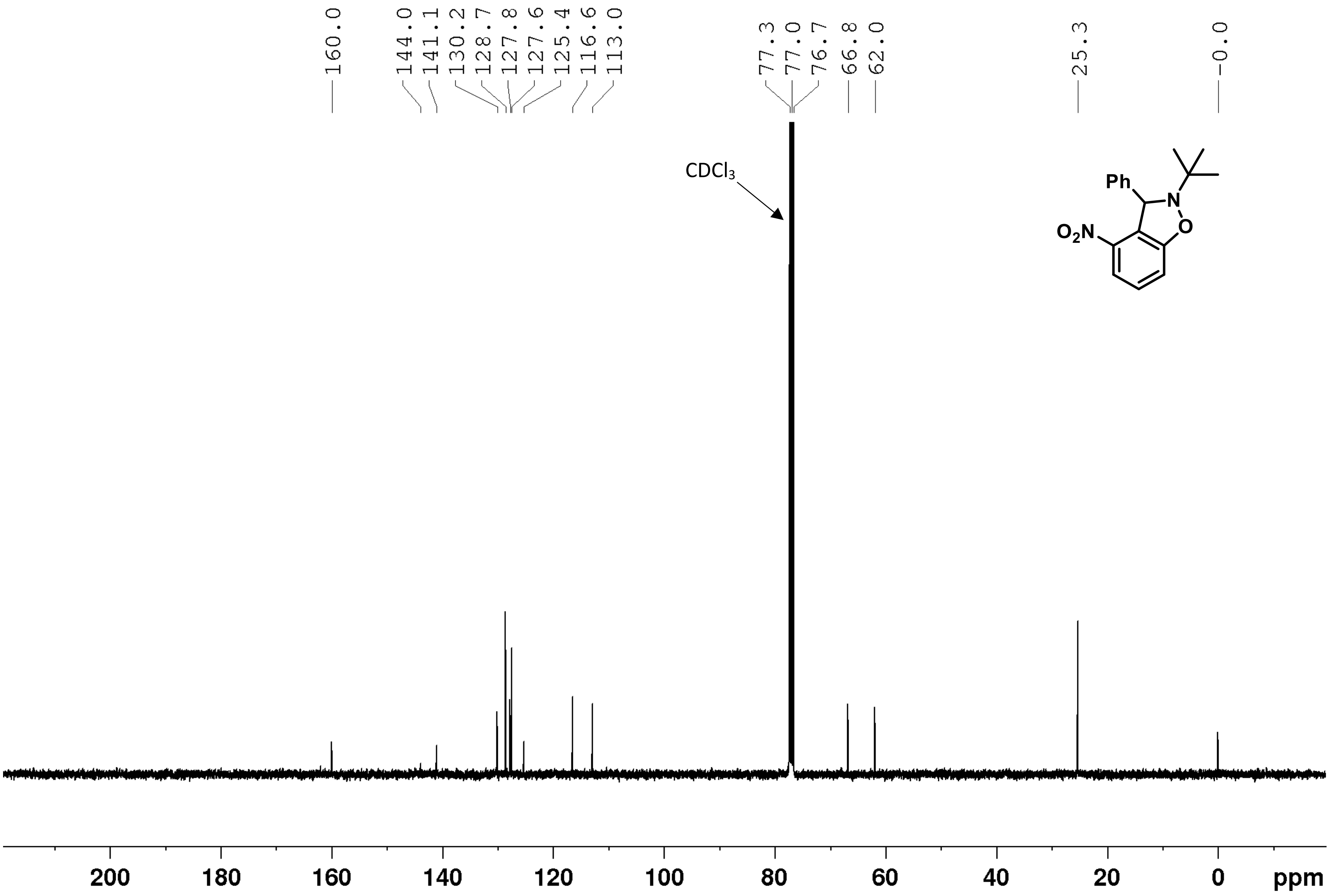

^{1}H-NMR of Compound 3dc in $CDCl_3$ at 400 MHz, 298K

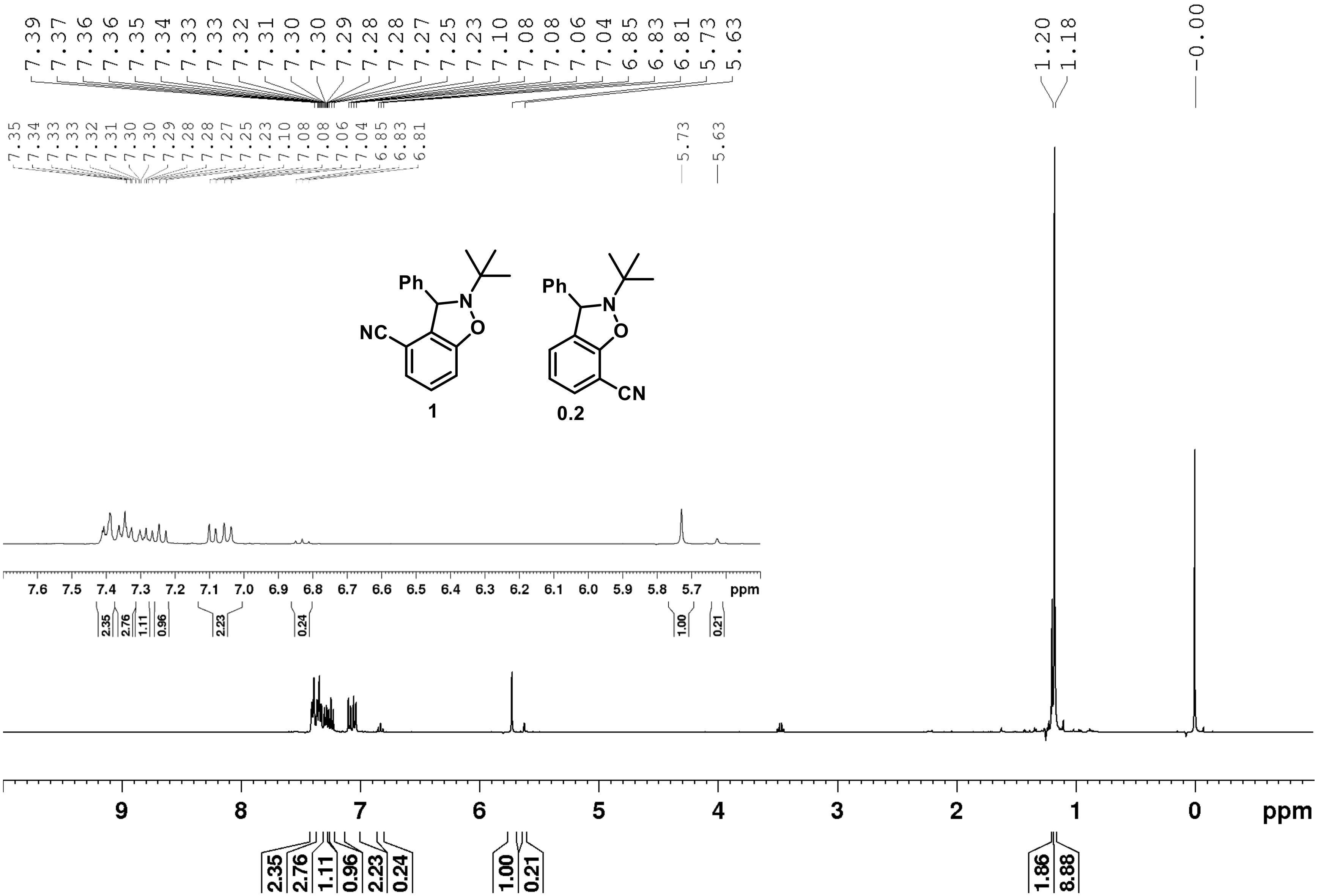

$^{13}C\{^{1}H\}$ NMR of Compound 3dc in $CDCl_3$ at 100 MHz, 298K

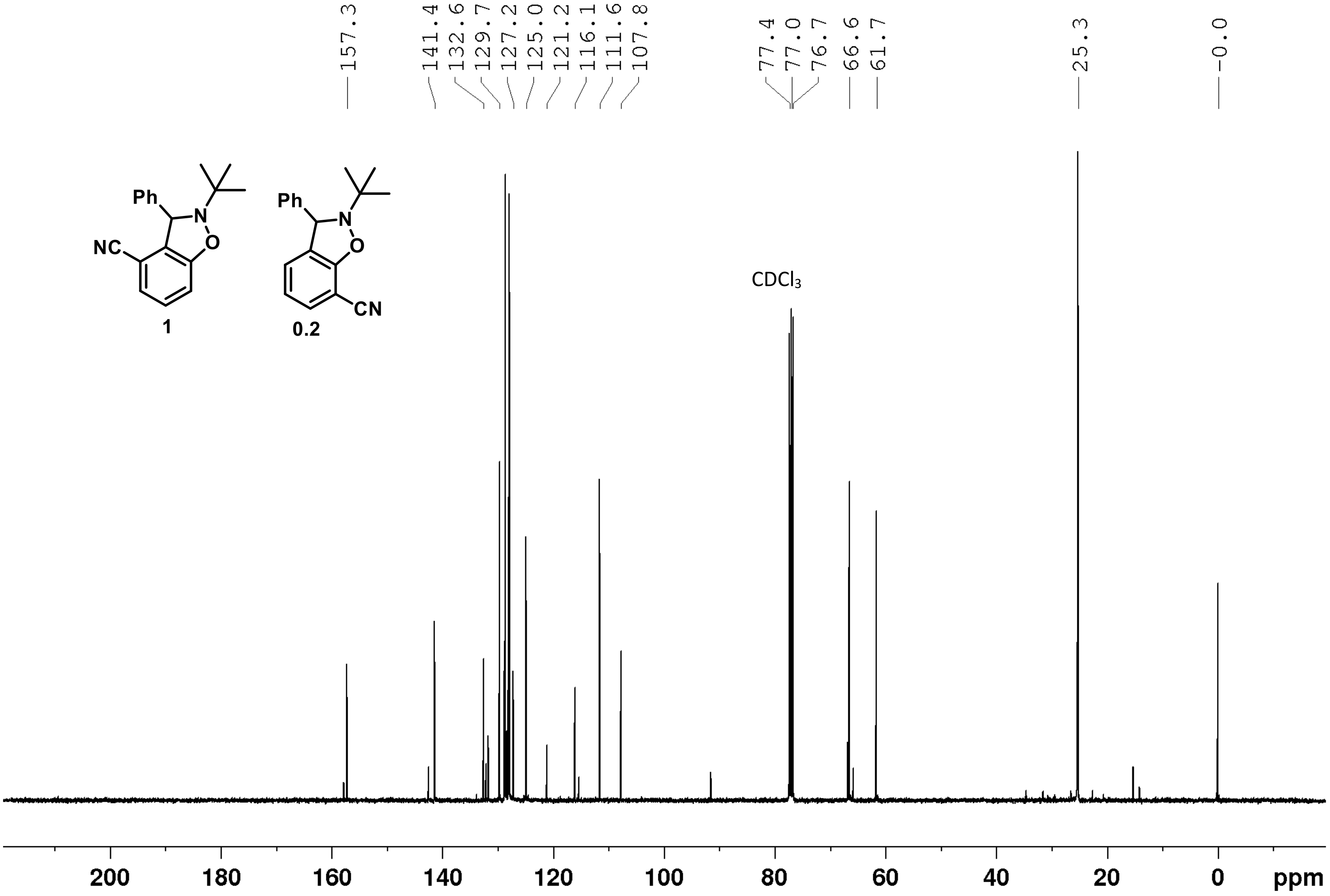

^{1}H-NMR of Compound 3ec in $CDCl_3$ at 400 MHz, 298K

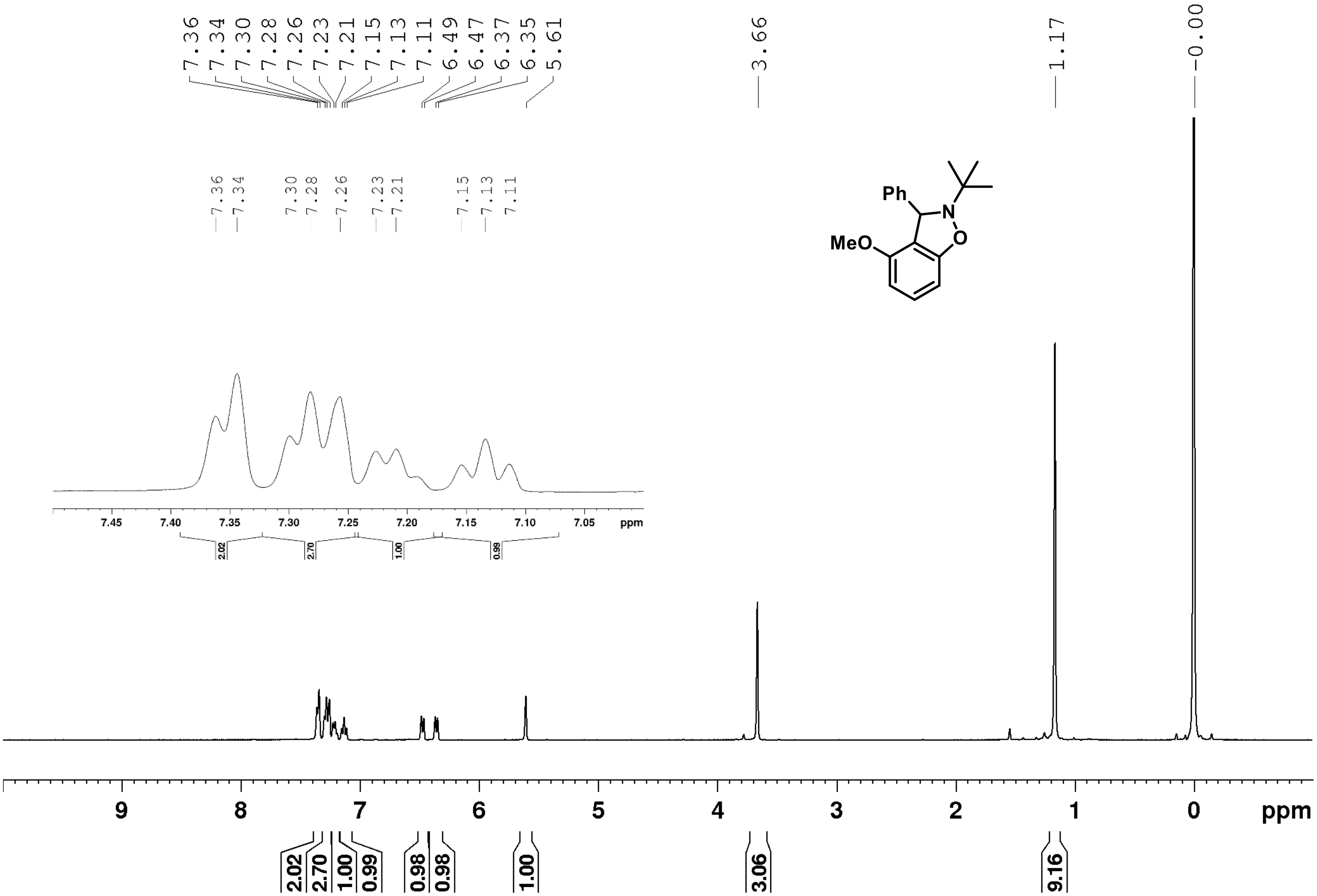

$^{13}C\{^1H\}$ NMR of Compound 3ec in $CDCl_3$ at 100 MHz, 298K

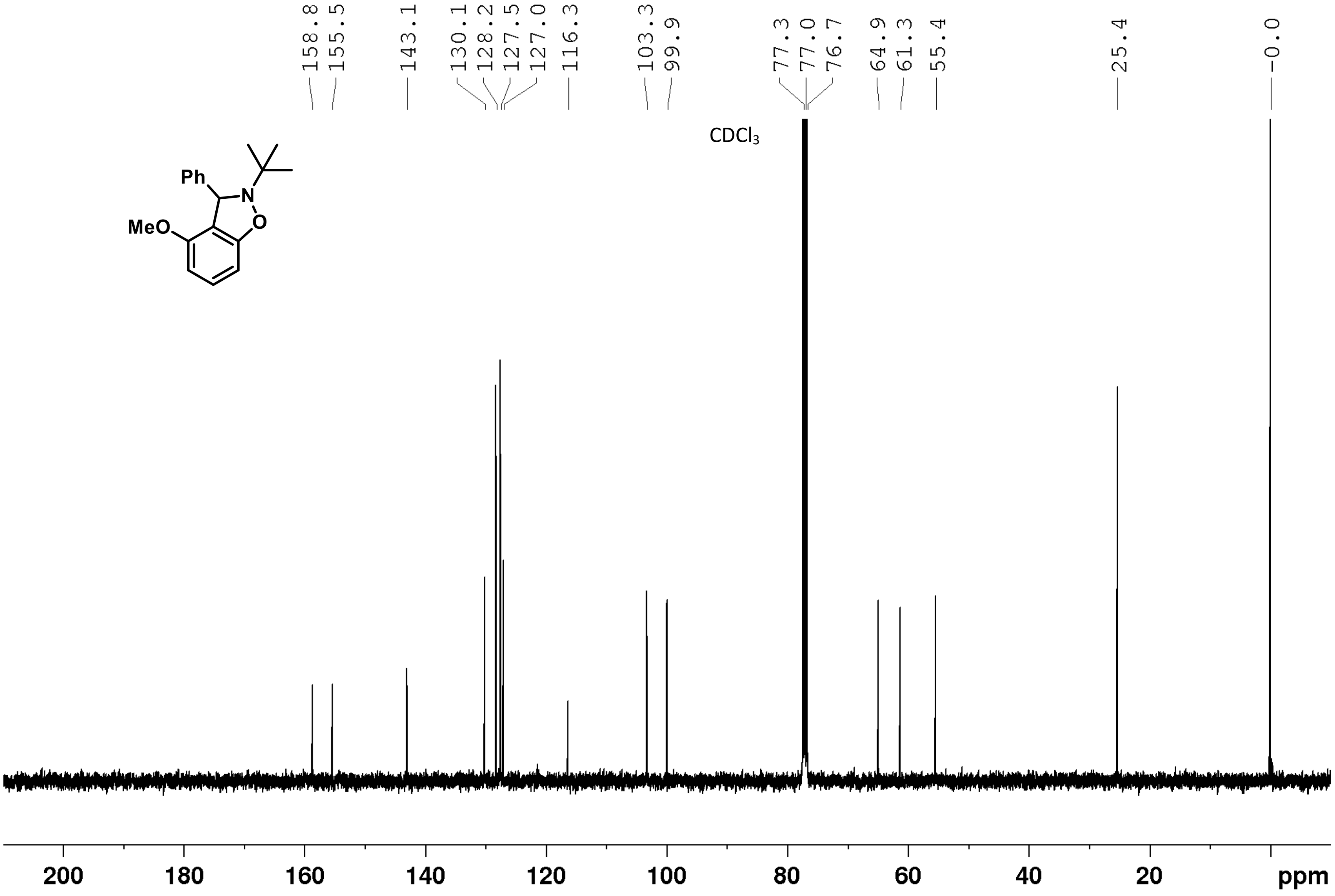

^{1}H-NMR of Compound 3fc in $CDCl_3$ at 400 MHz, 298K

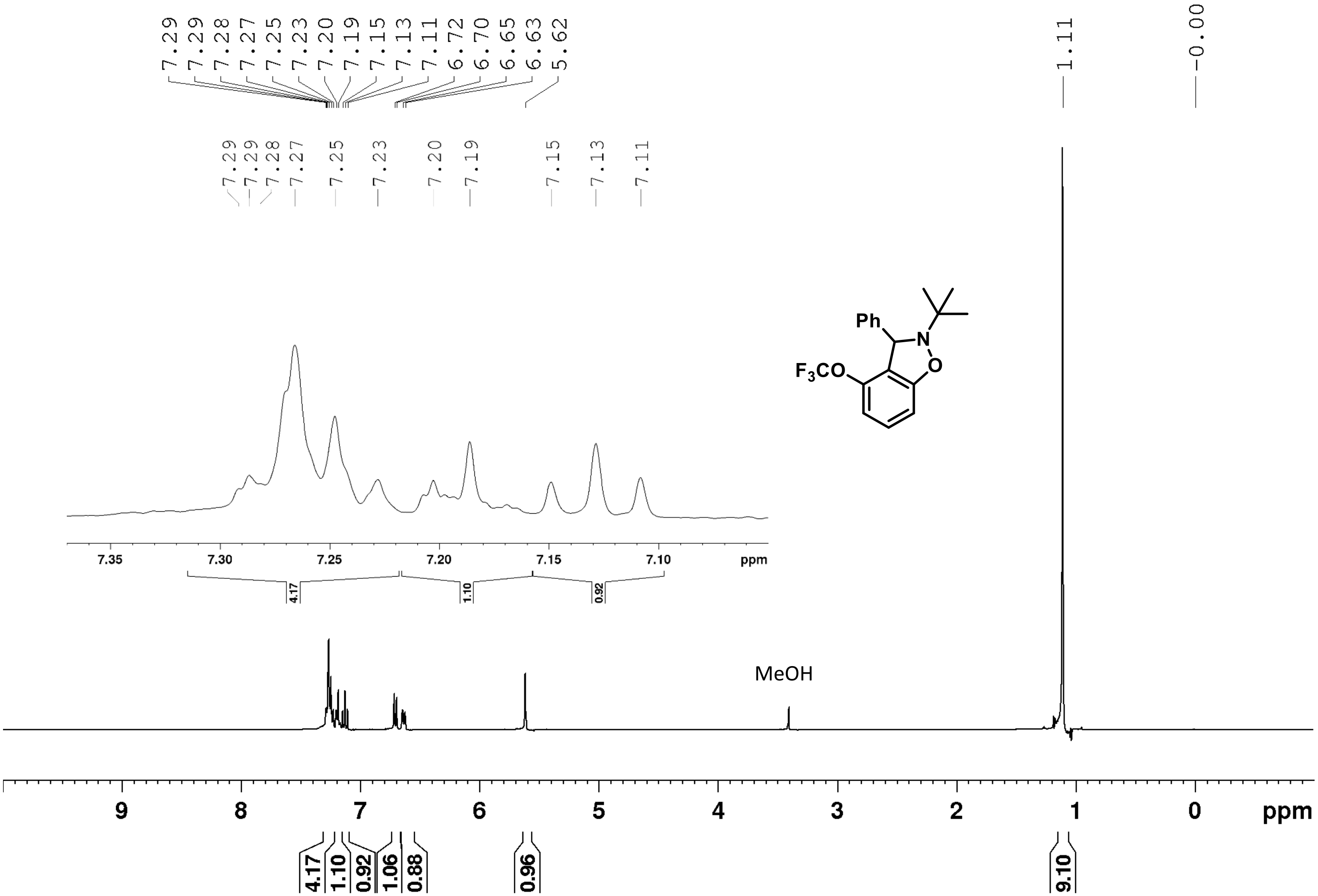

$^{13}C\{^1H\}$ NMR of Compound 3fc in $CDCl_3$ at 100 MHz, 298K

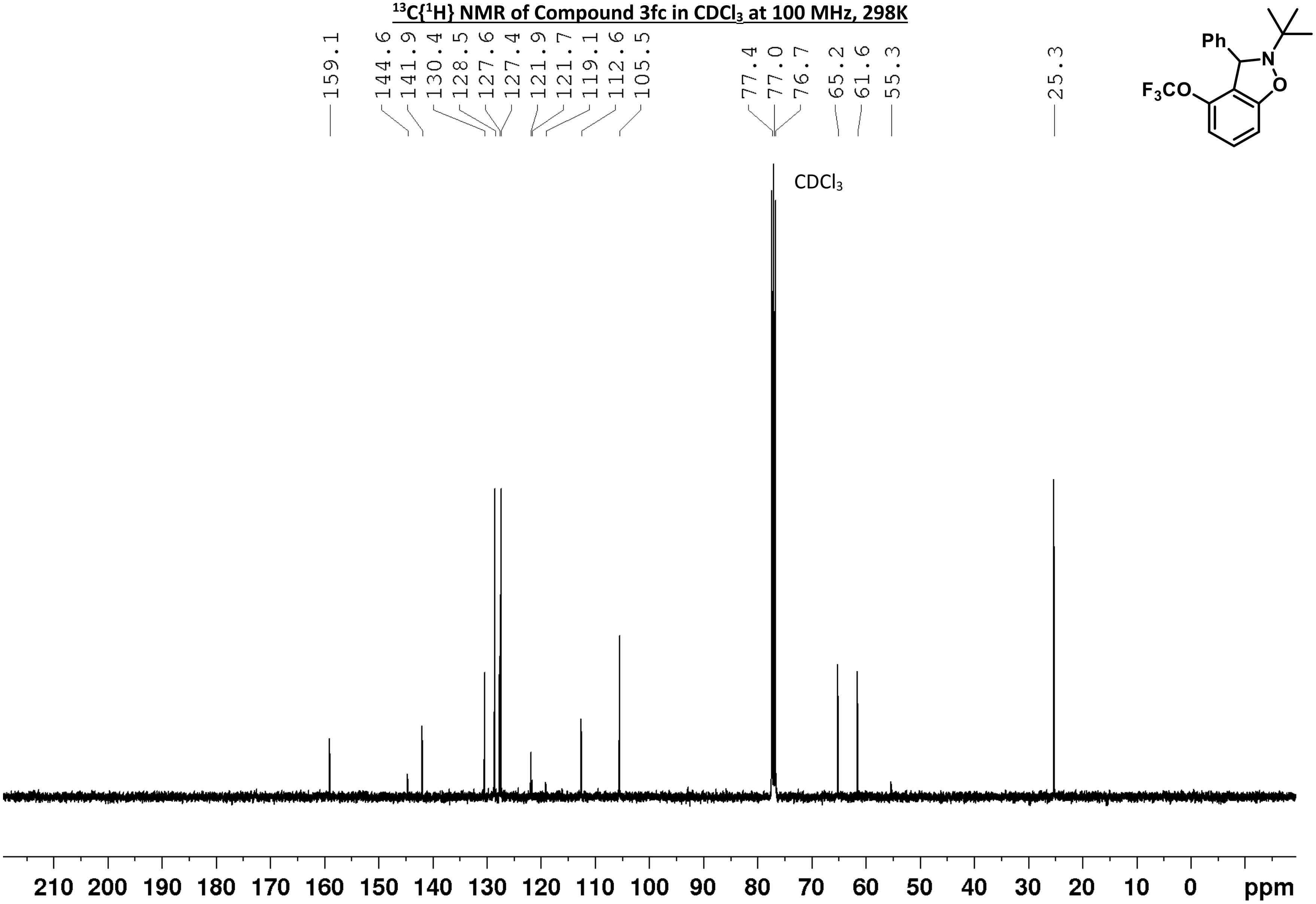

$^{19}F\{^{1}H\}$ NMR of compound 3fc in $CDCl_3$ at 376 MHz, 298K

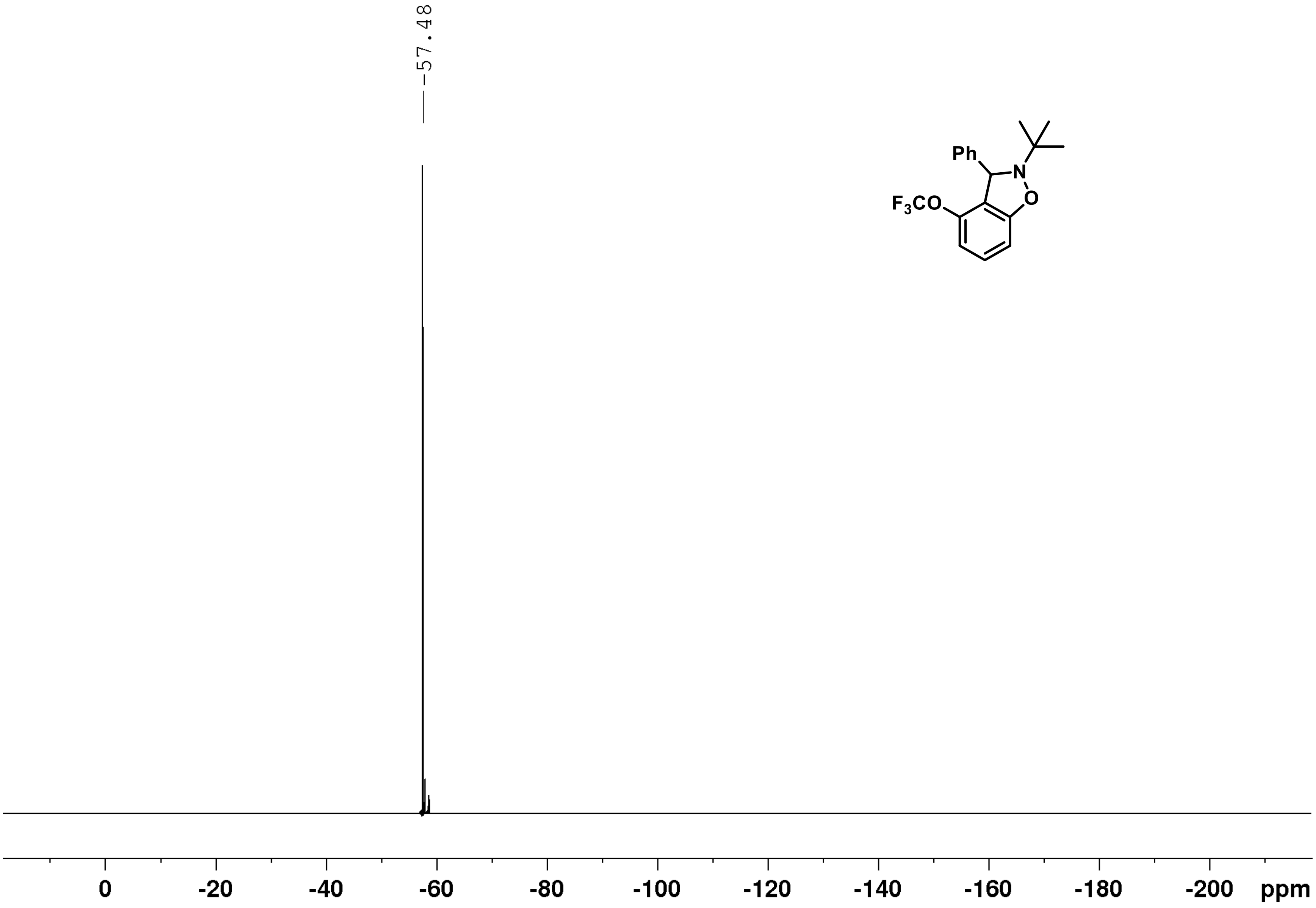

<u>^{1}H-NMR of Compound 3gc in $CDCl_3$ at 400 MHz, 298K</u>

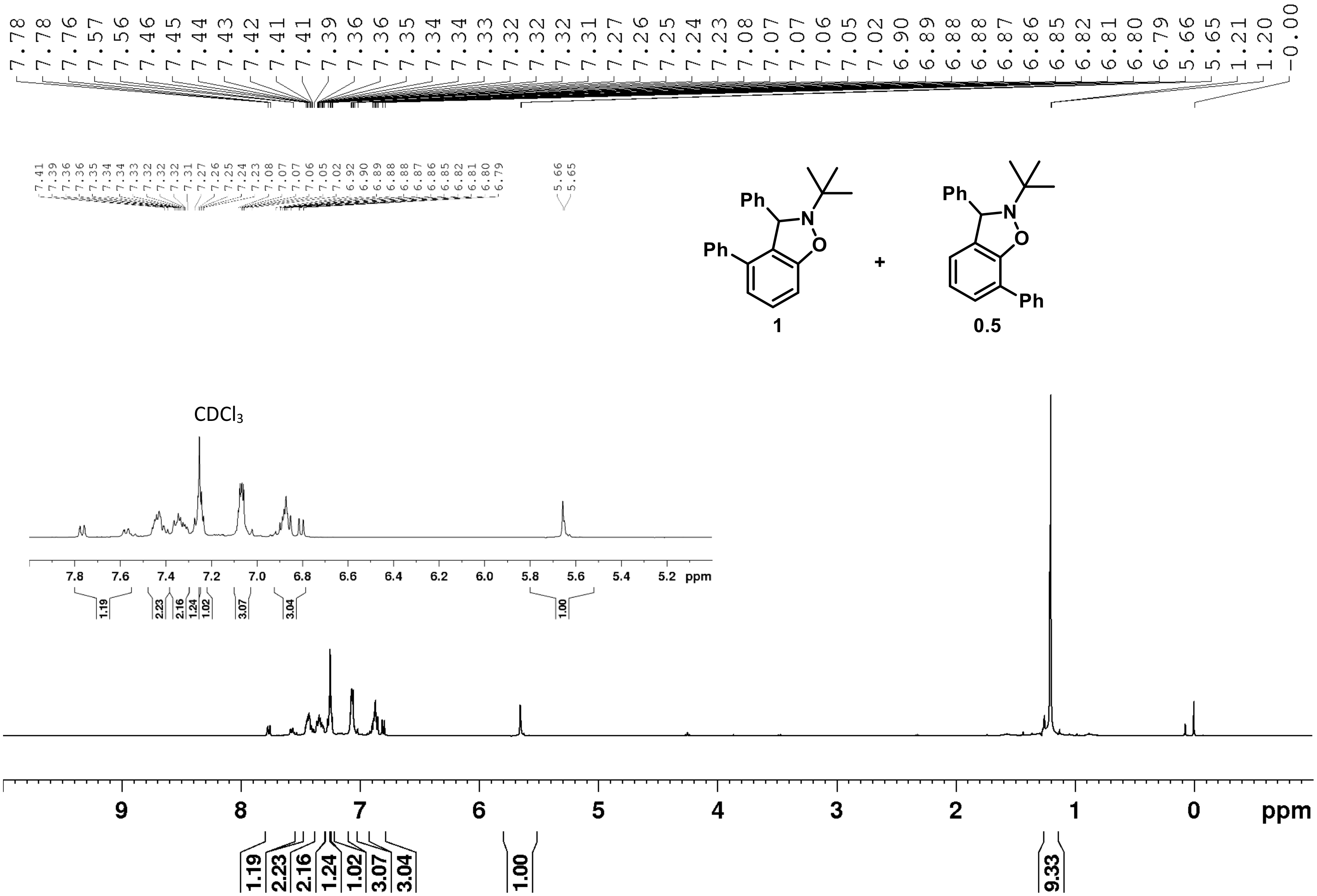

$^{13}C\{^1H\}$ NMR of Compound 3gc in $CDCl_3$ at 100 MHz, 298K

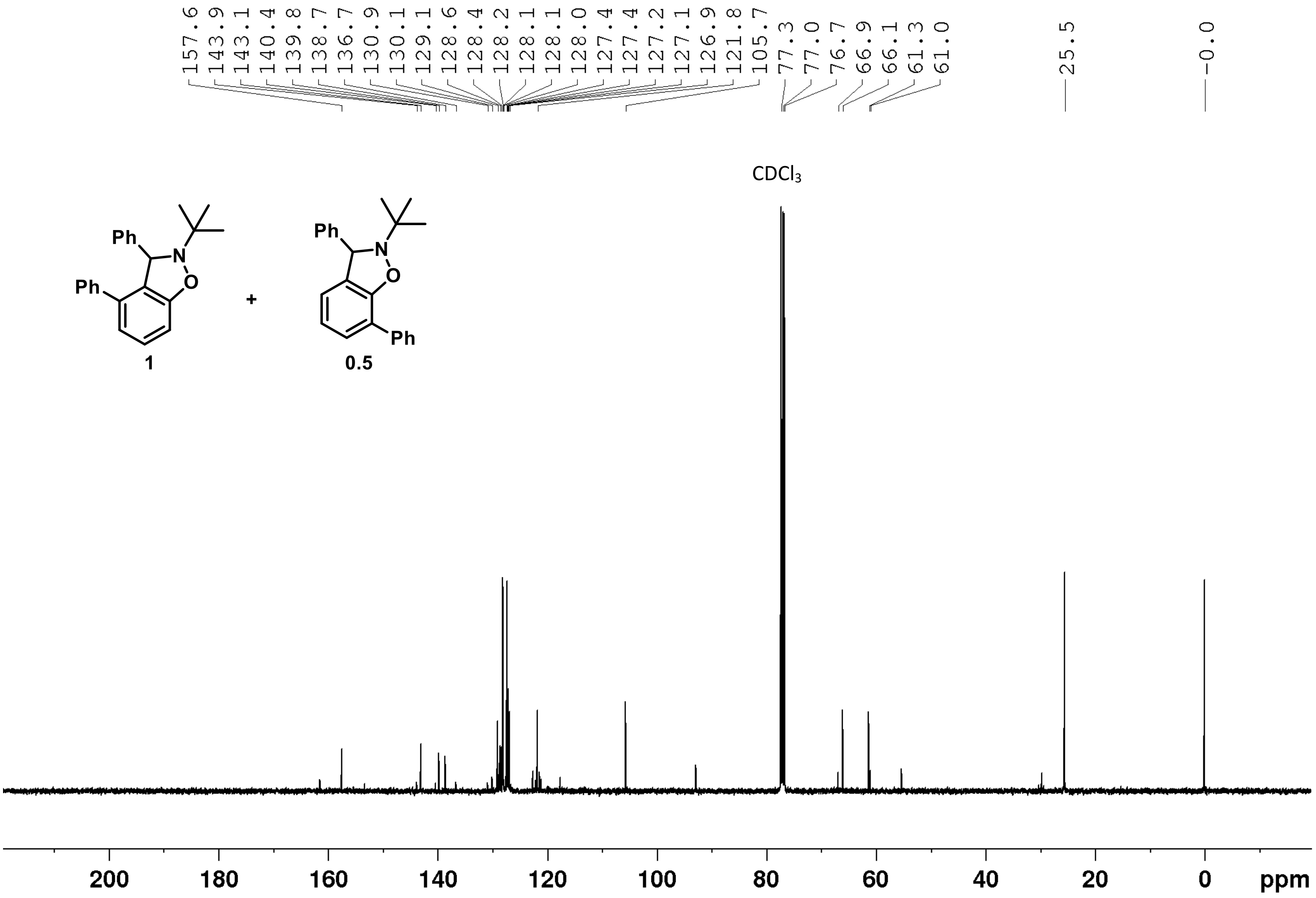

NOESY NMR of purified 3gc (major) in $CDCl_3$ at 400MHz, 298K

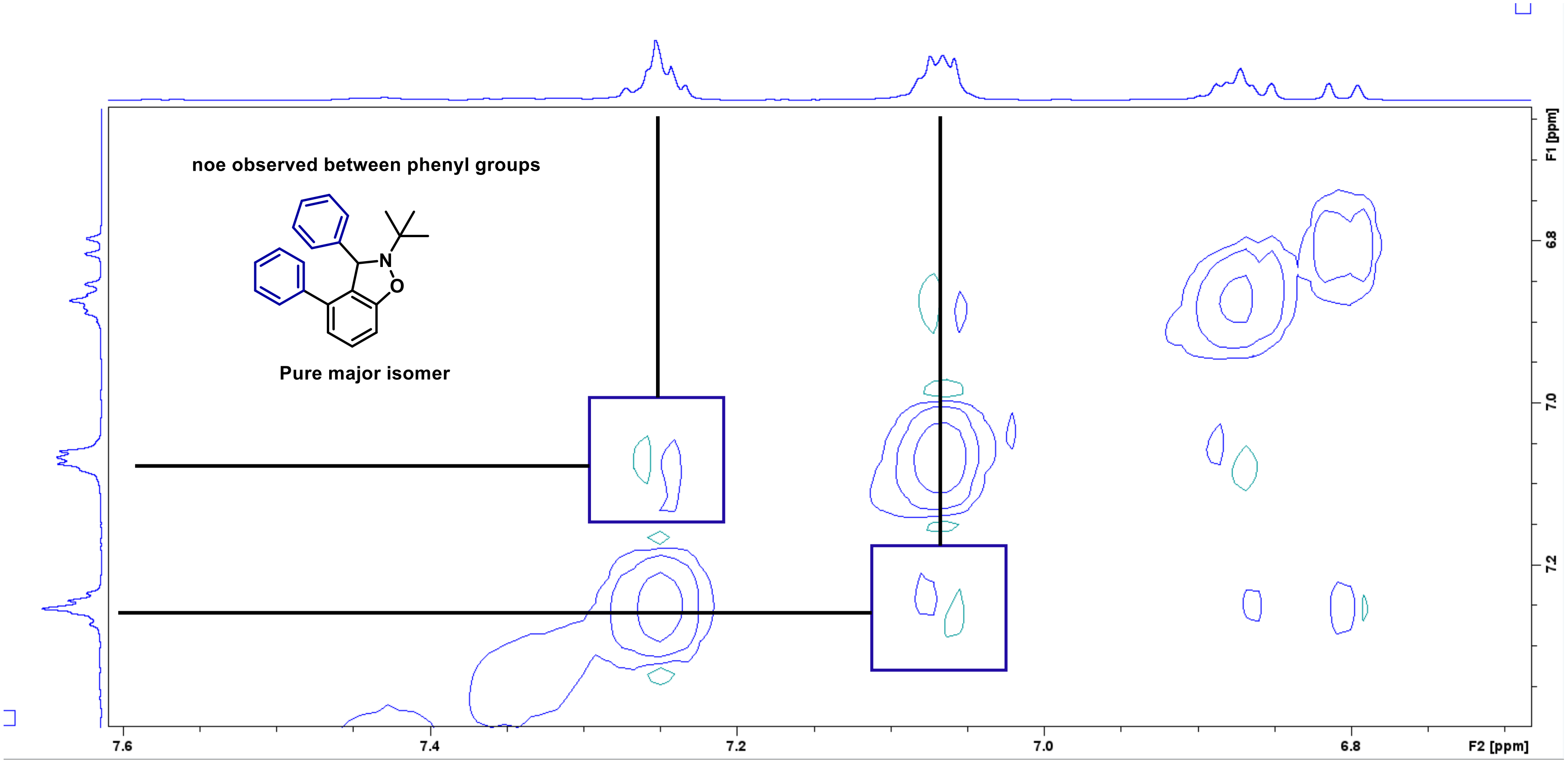

^{1}H-NMR of Compound 3hc in $CDCl_3$ at 400 MHz, 298K

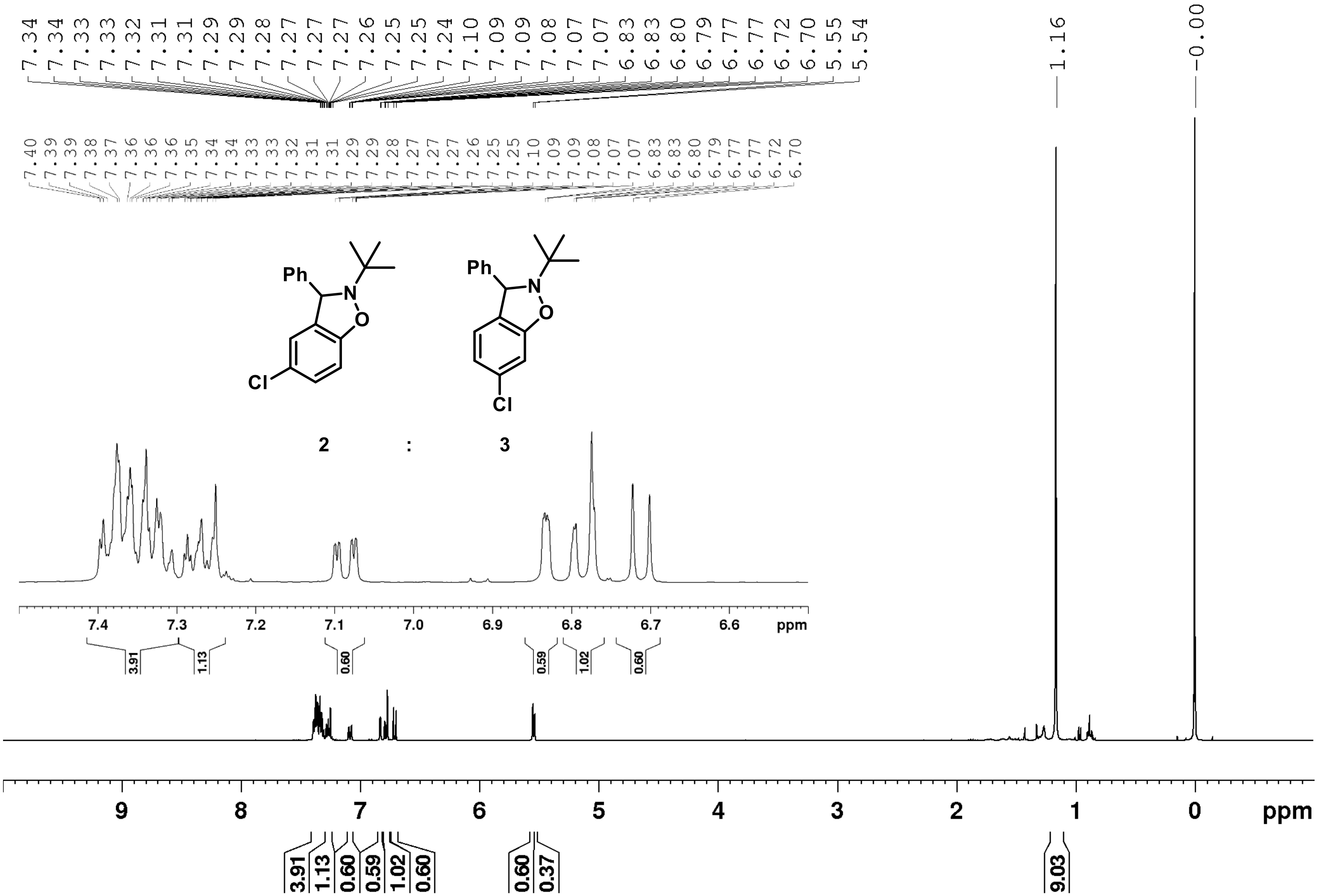

$^{13}C\{^{1}H\}$ NMR of Compound 3hc in $CDCl_3$ at 100 MHz, 298K

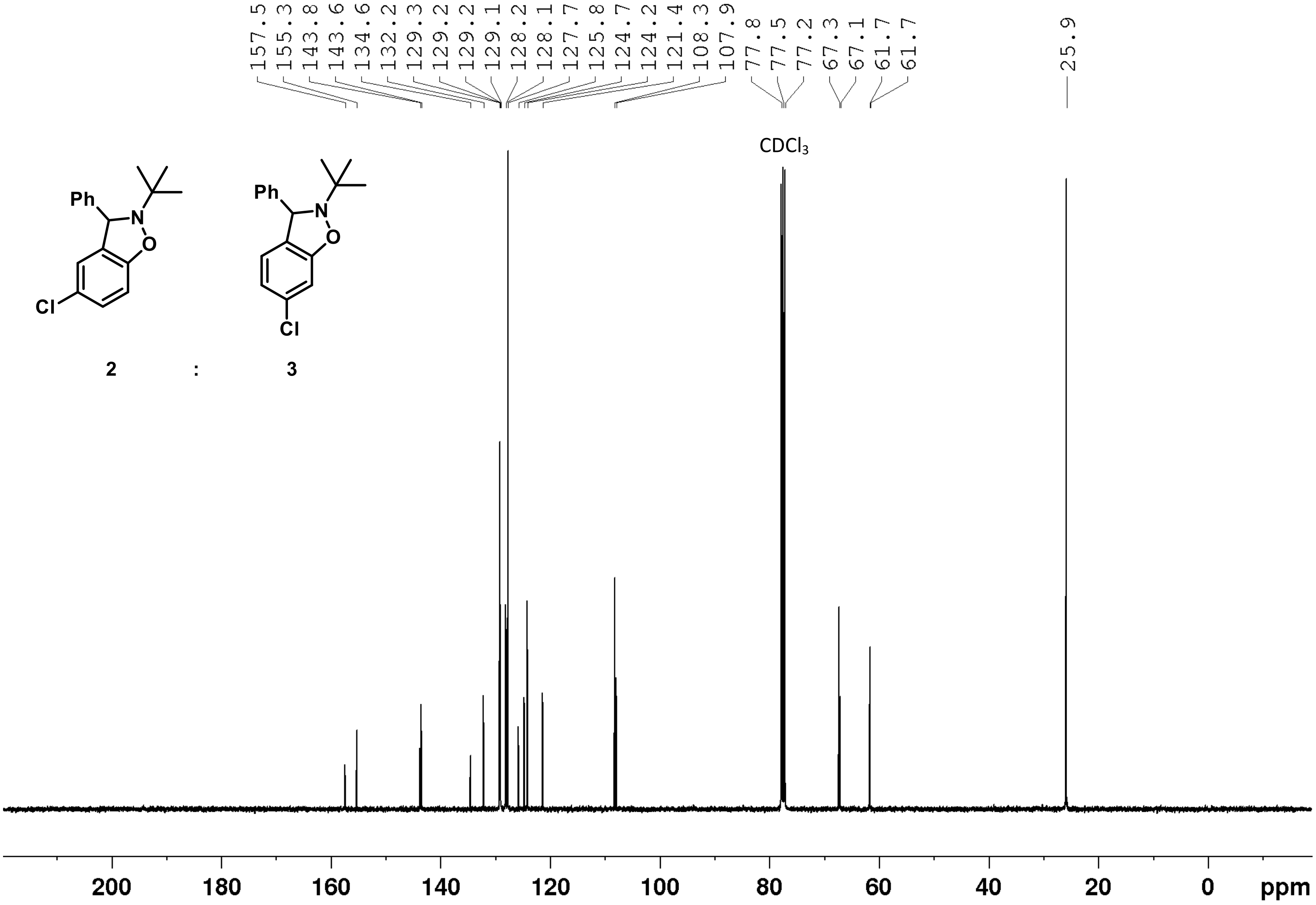

^{1}H-NMR of Compound 3ic in $CDCl_3$ at 400 MHz, 298K

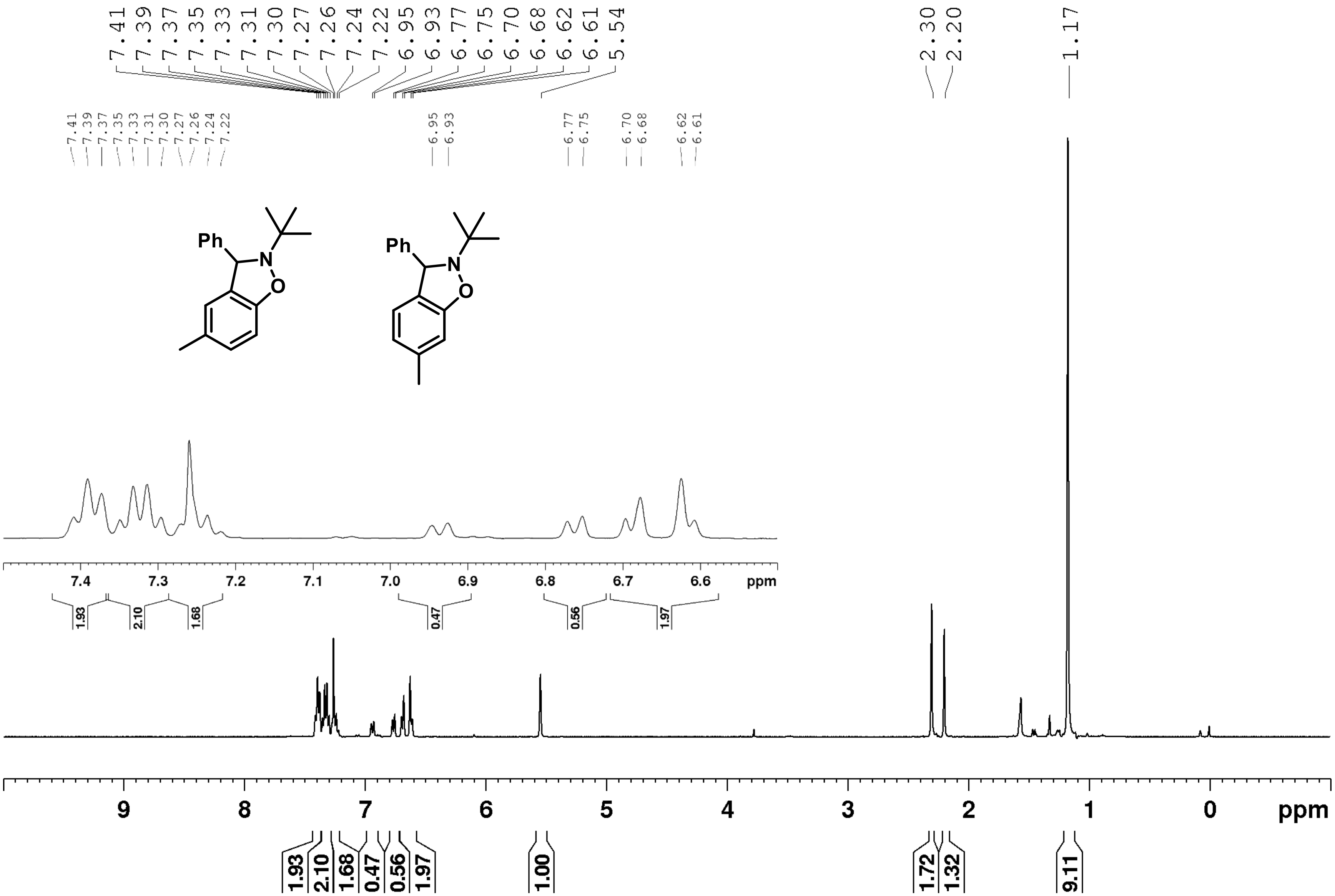

$^{13}C\{^1H\}$ NMR of Compound 3ic in $CDCl_3$ at 100 MHz, 298K

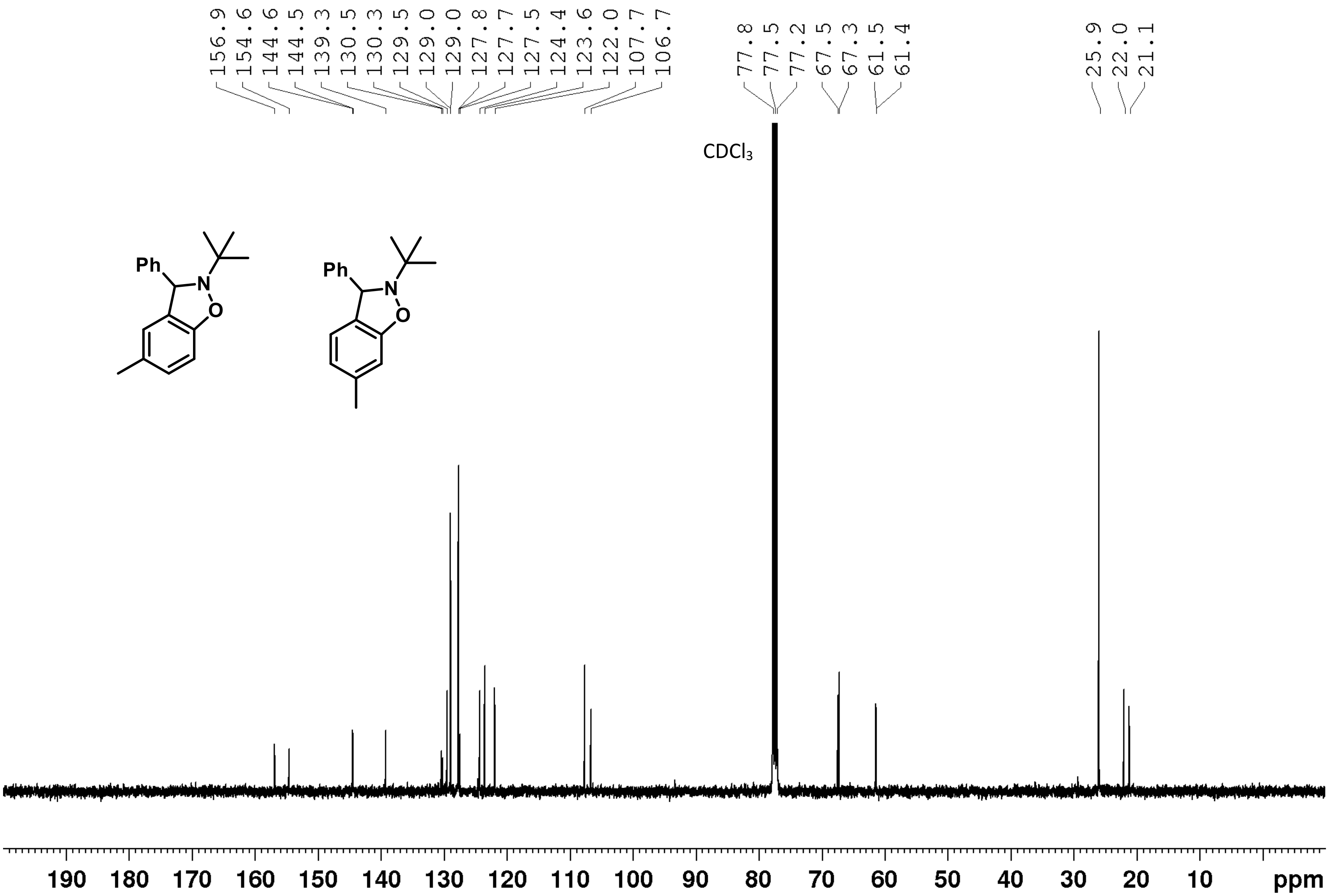

^{1}H-NMR of 3jc in $CDCl_3$ at 400 MHz, 298K

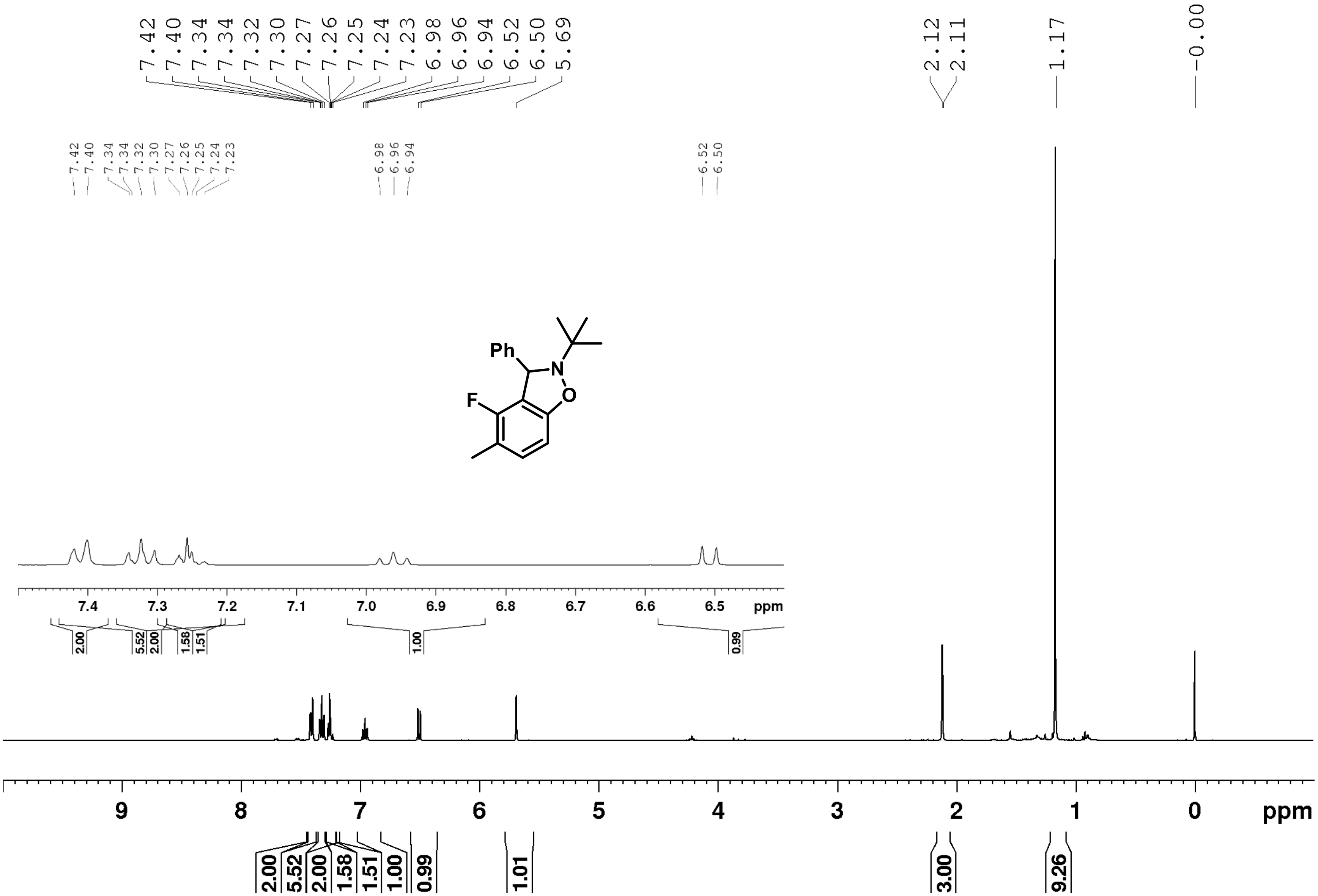

$^{13}C\{^{1}H\}$ NMR of Compound 3jc in $CDCl_3$ at 100 MHz, 298K

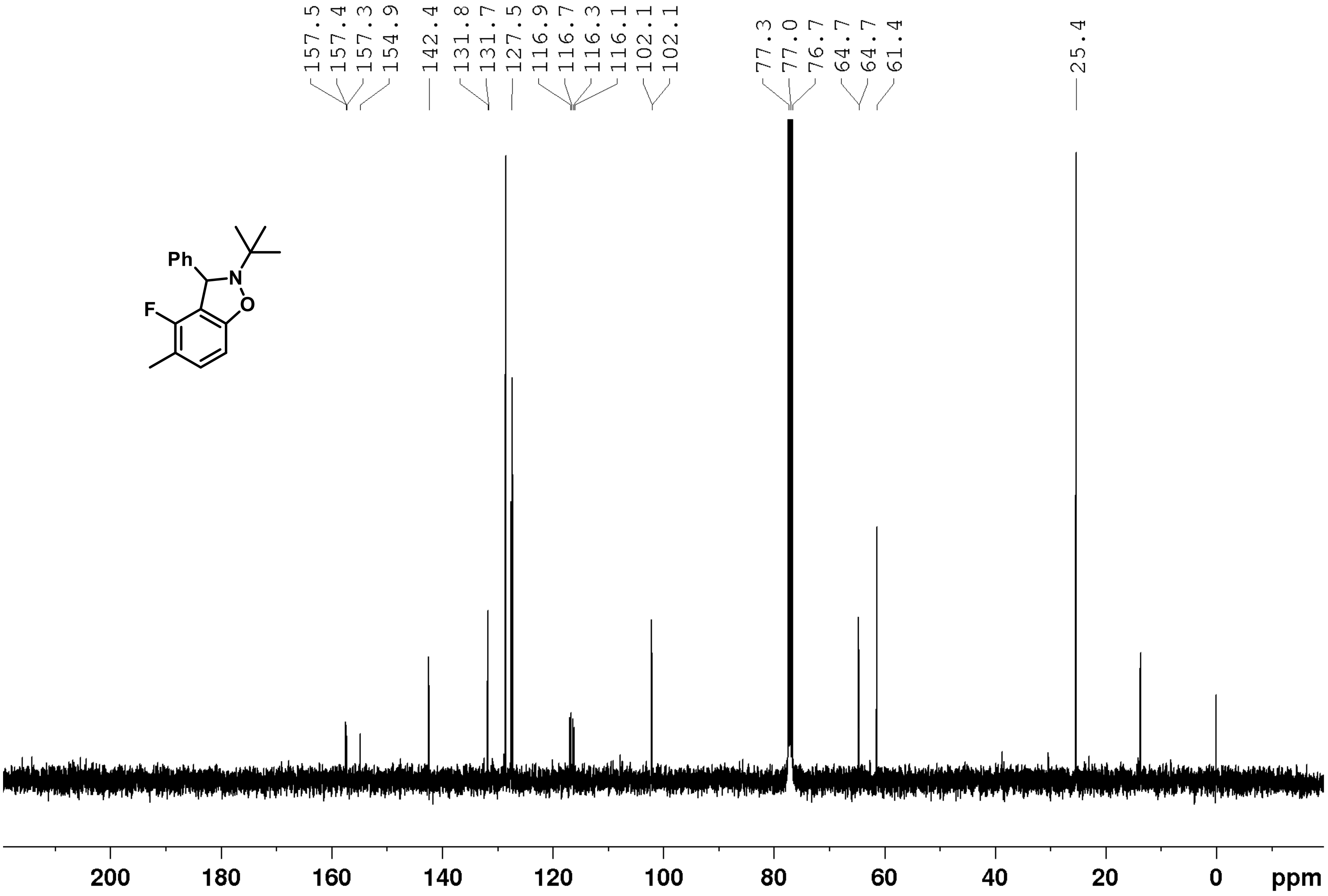

^{19}F-NMR of 3jc in $CDCl_3$ at 376 MHz, 298K

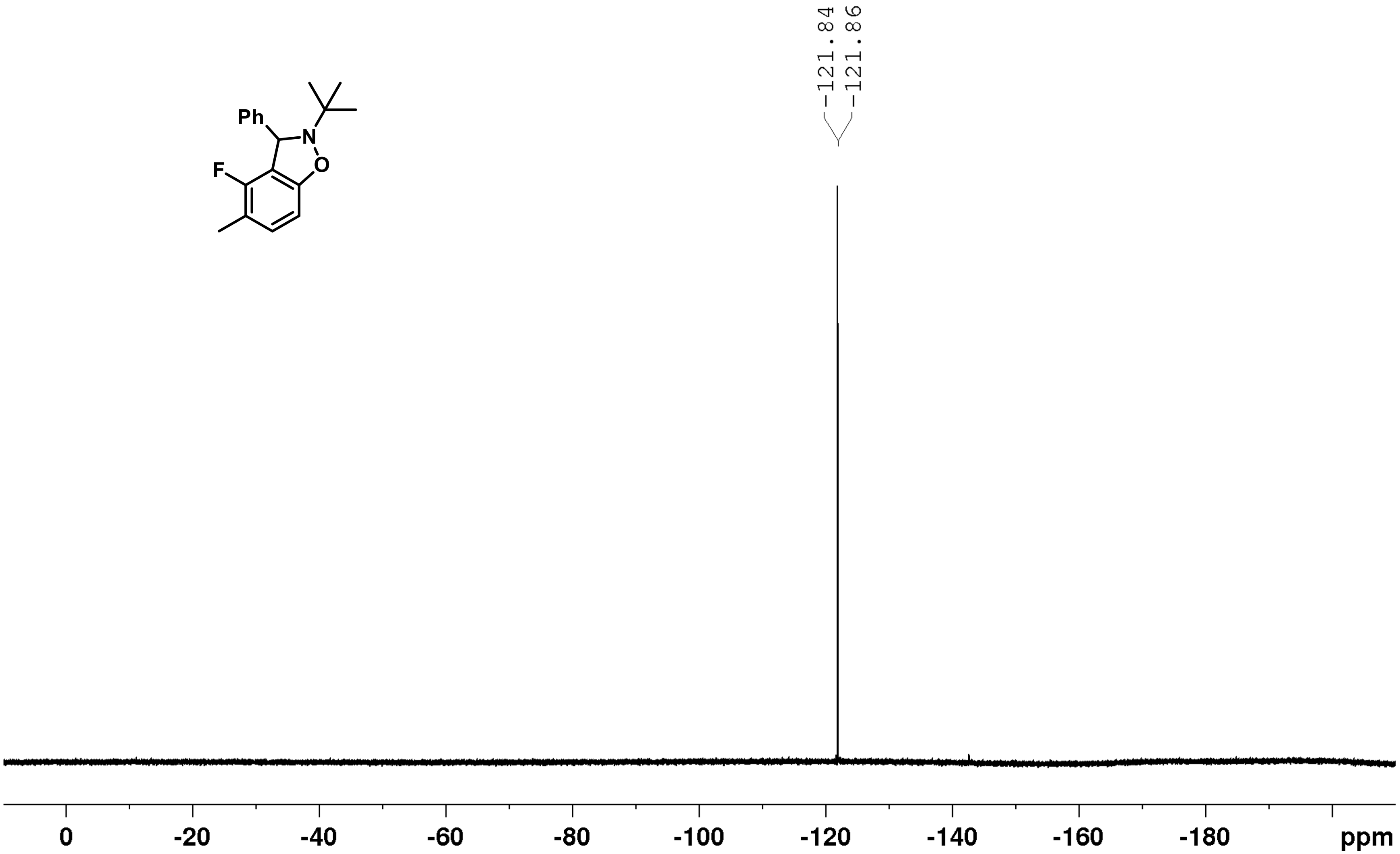

^{1}H-NMR of Compound 3kc in $CDCl_3$ at 400 MHz, 298K

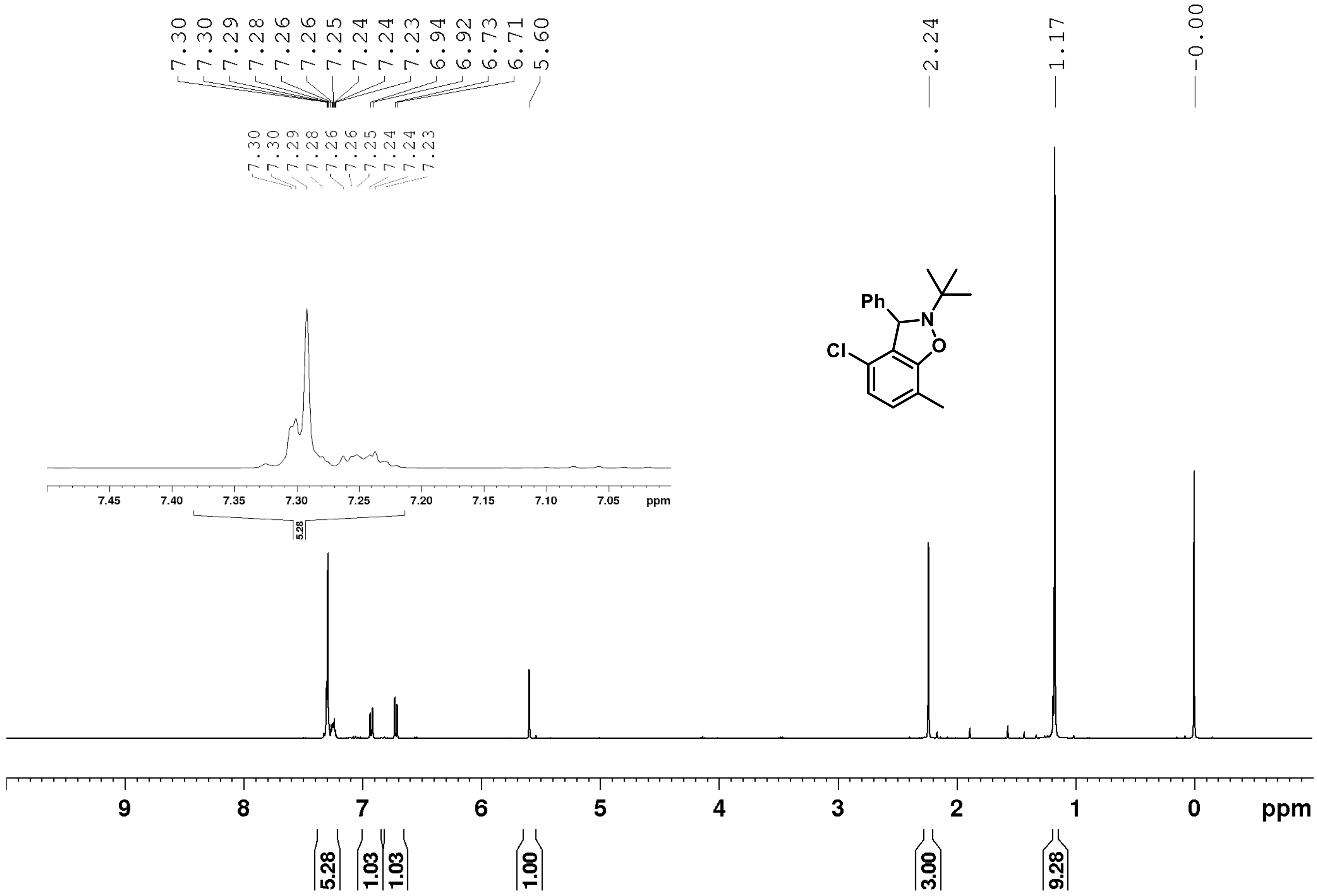

$^{13}C\{^1H\}$ NMR of Compound 3kc in $CDCl_3$ at 100 MHz, 298K

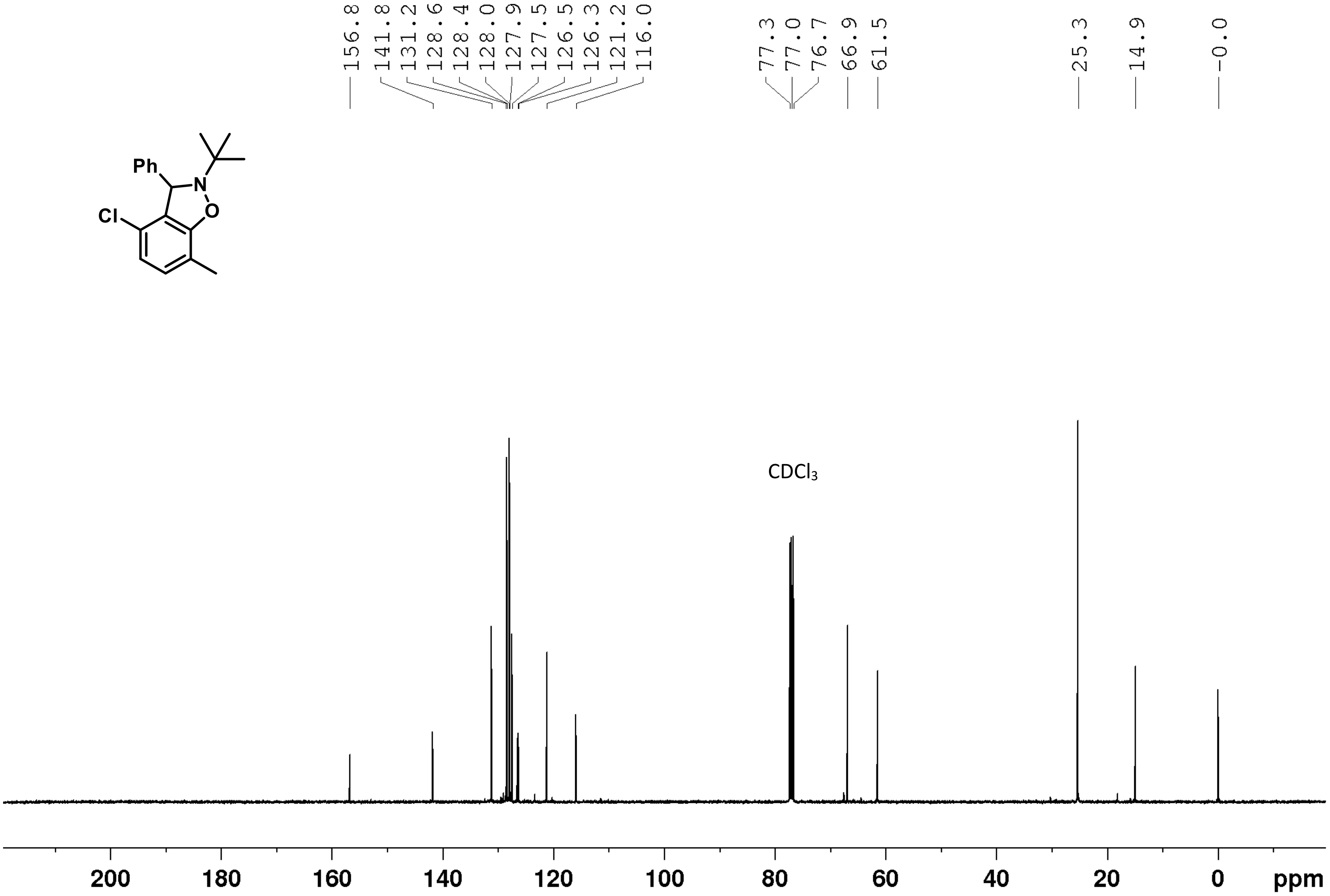

^{1}H-NMR of Compound 3lc in $CDCl_3$ at 400 MHz, 298K

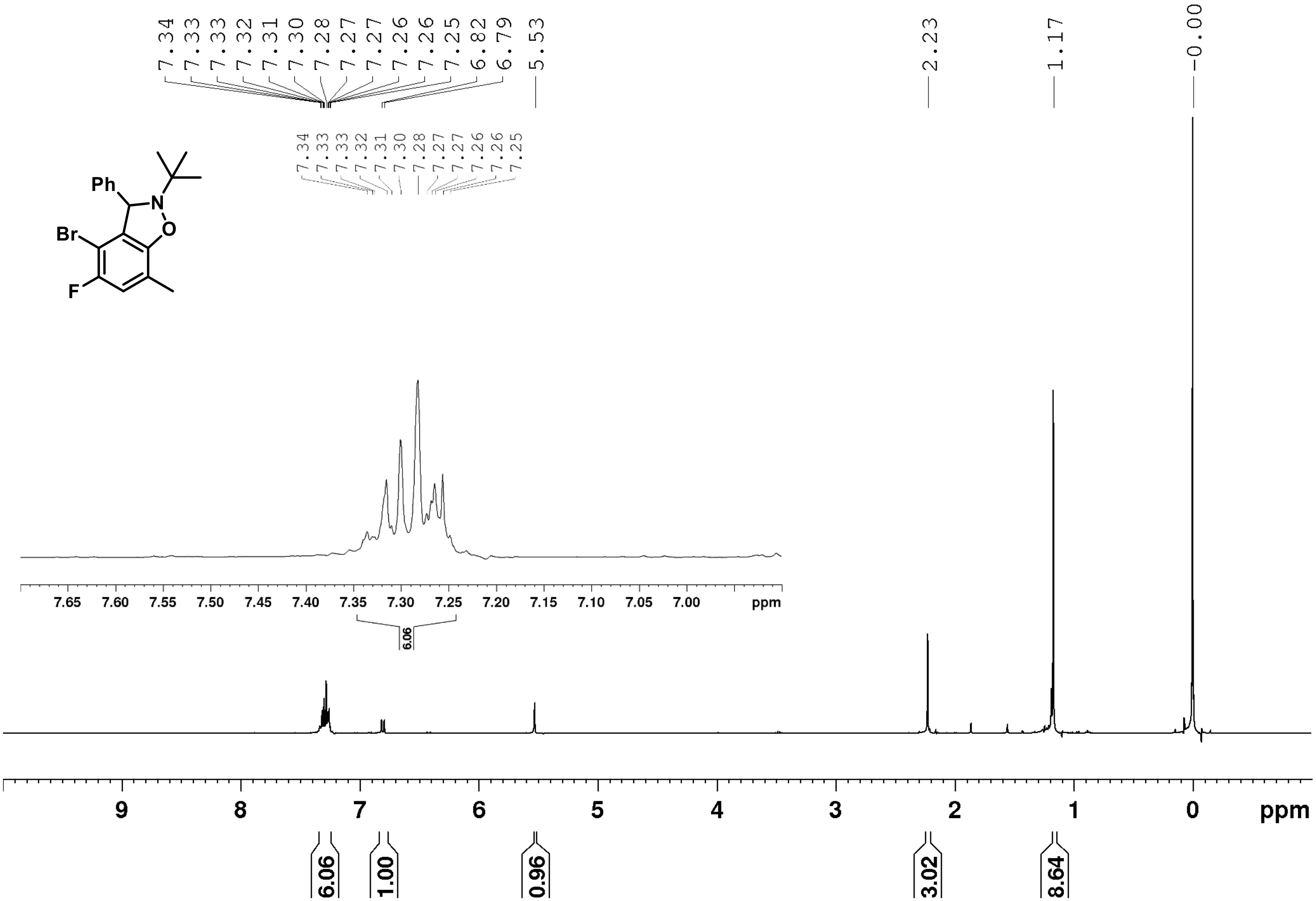

$^{13}C\{^{1}H\}$ NMR of Compound 3lc in $CDCl_3$ at 100 MHz, 298K

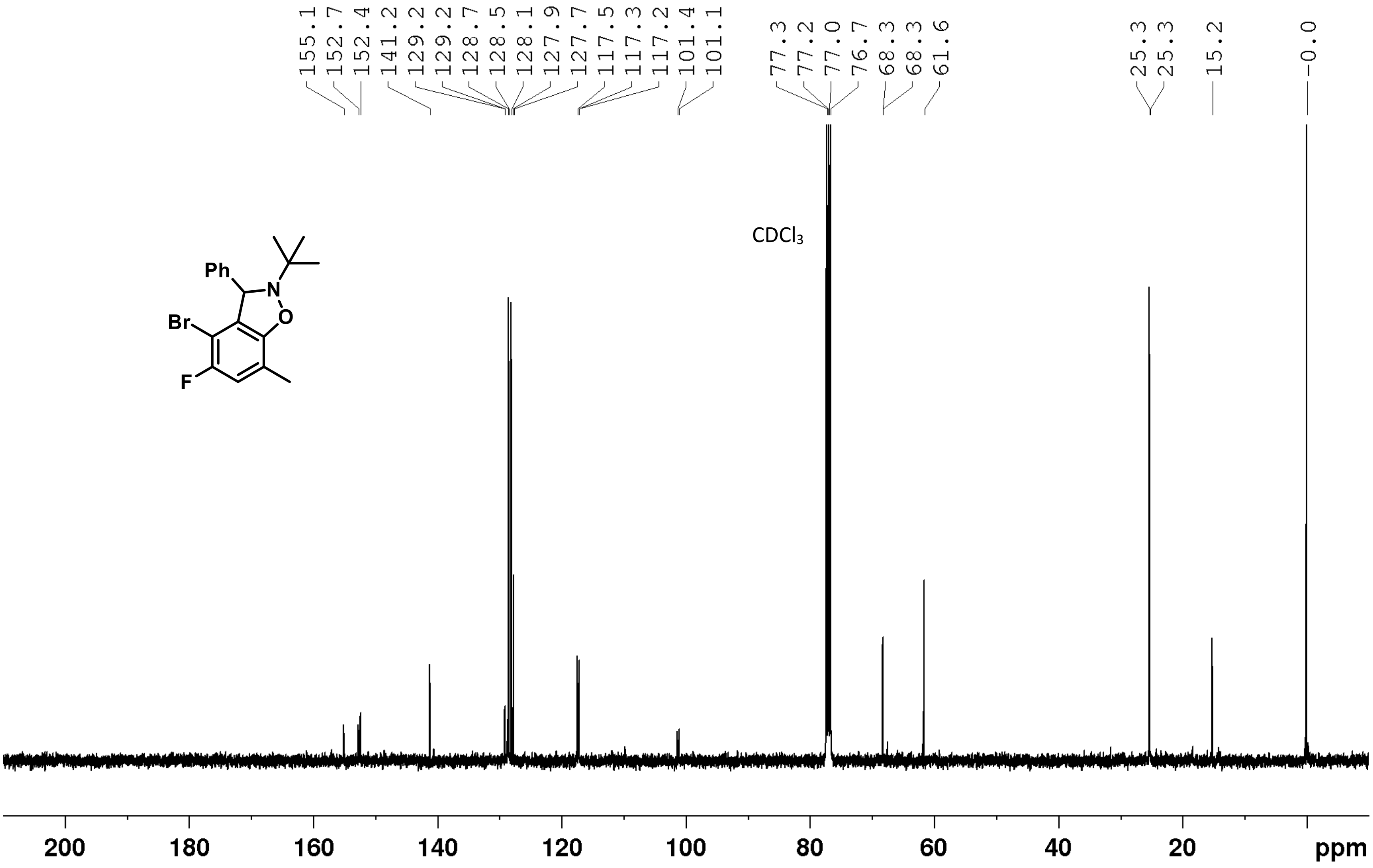

$^{19}F\{^{1}H\}$ NMR of compound 3lc in $CDCl_3$ at 376 MHz, 298K

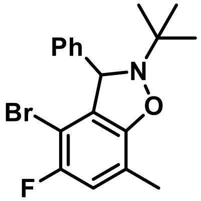

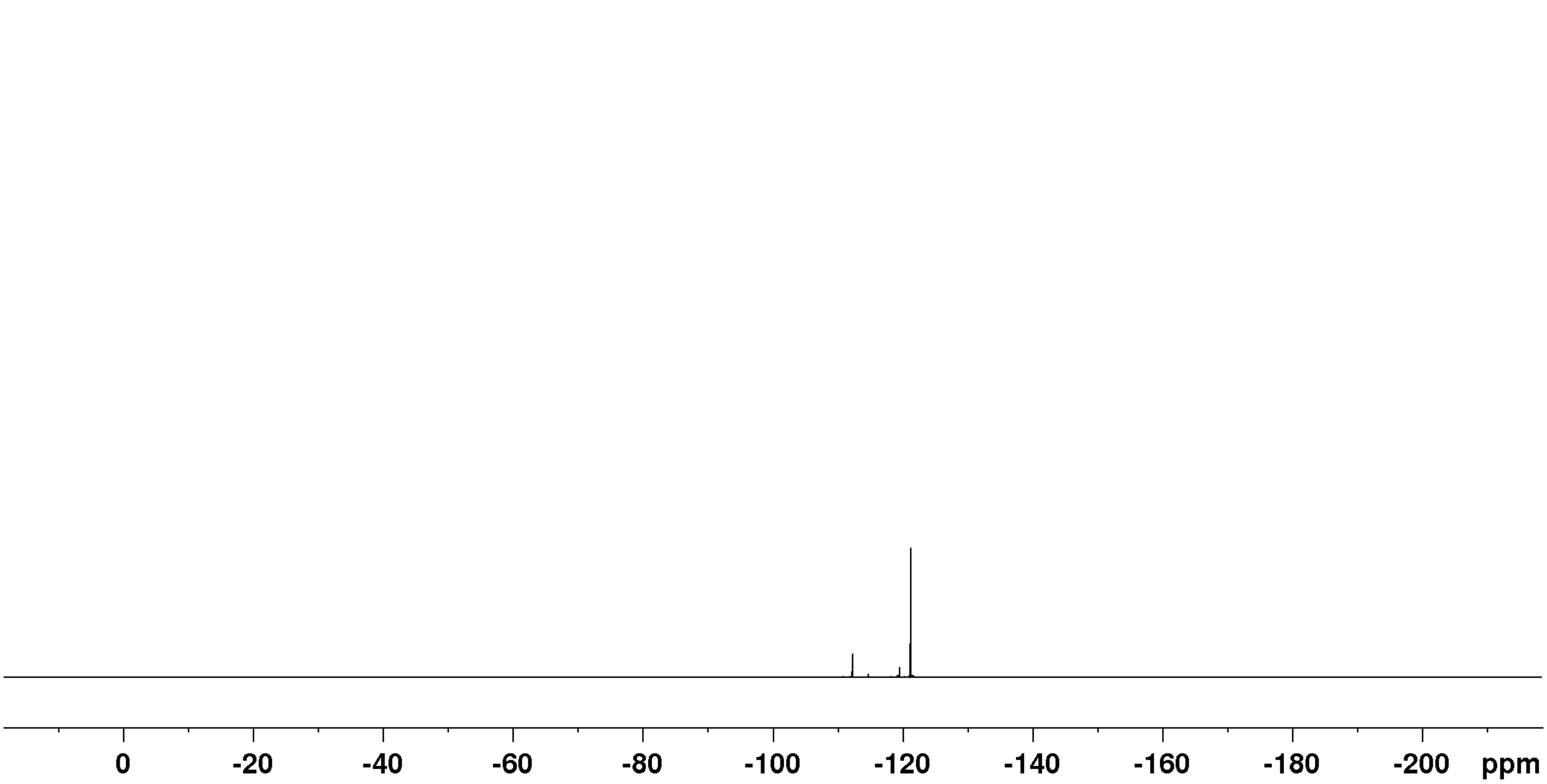

^{1}H-NMR of Compound 3bf in $CDCl_3$ at 400 MHz, 298K

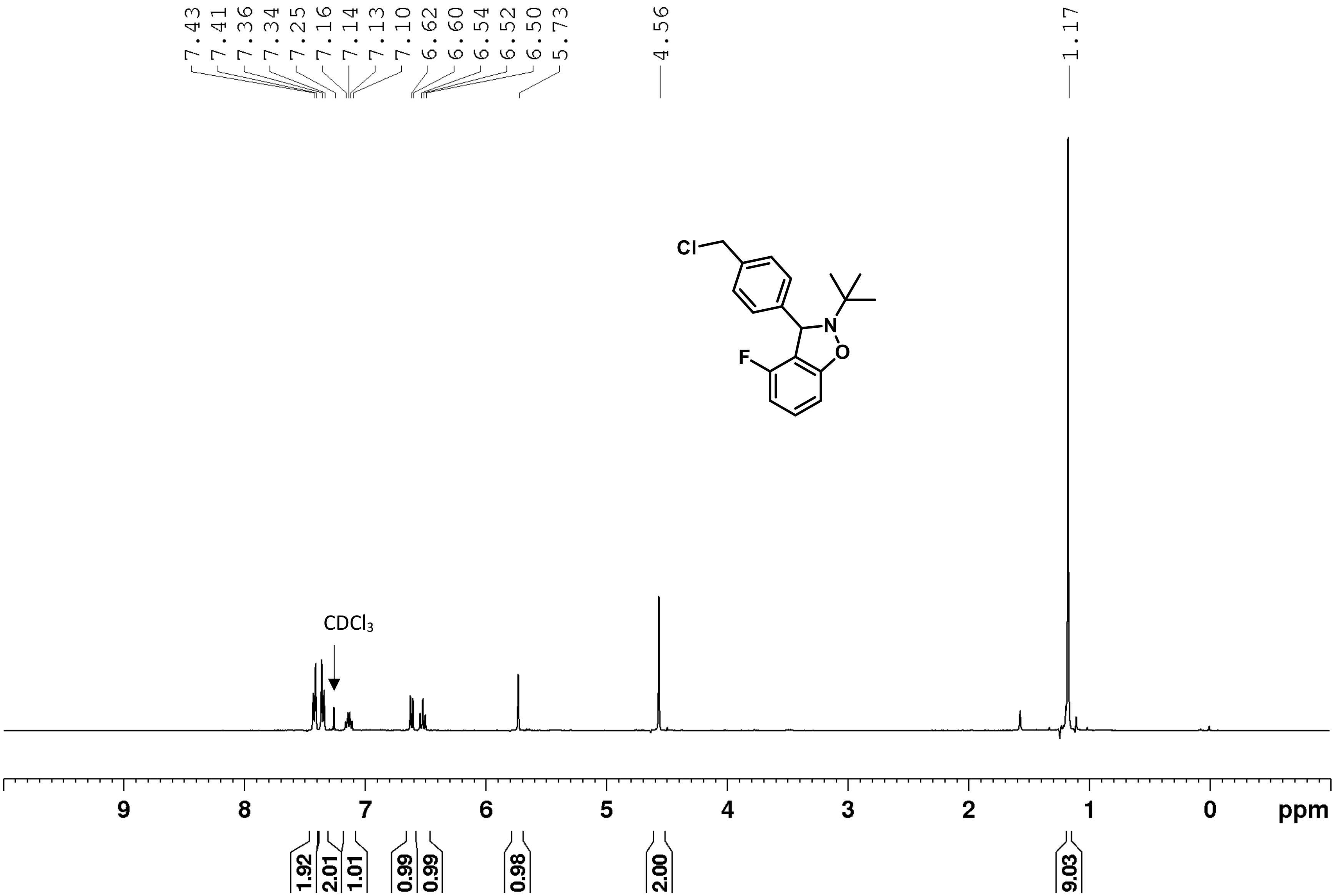

$^{13}C\{^{1}H\}$ NMR of Compound 3bf in $CDCl_3$ at 100 MHz, 298K

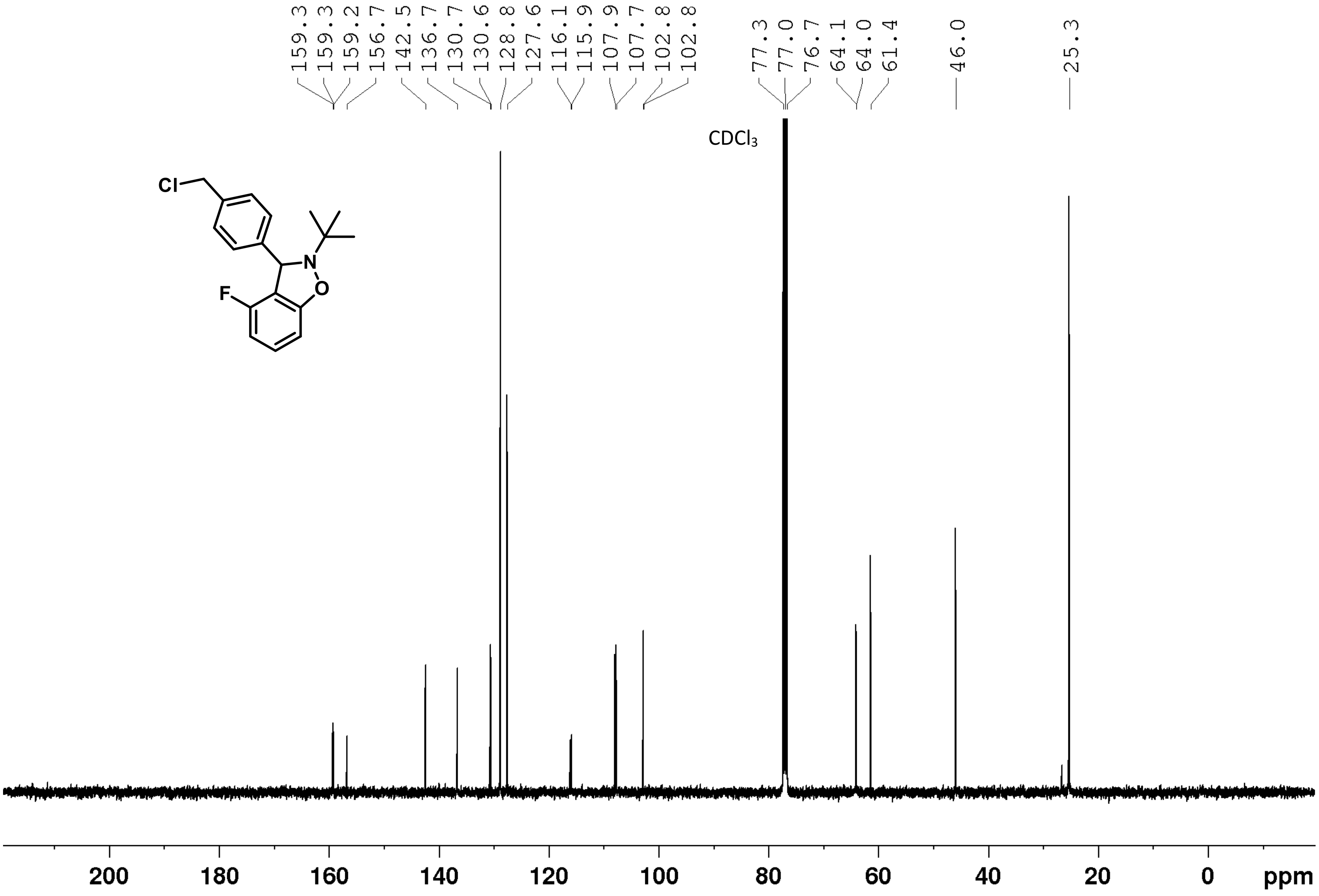

$^{19}F\{^{1}H\}$ NMR of Compound 3bf in $CDCl_3$ at 376 MHz, 298K

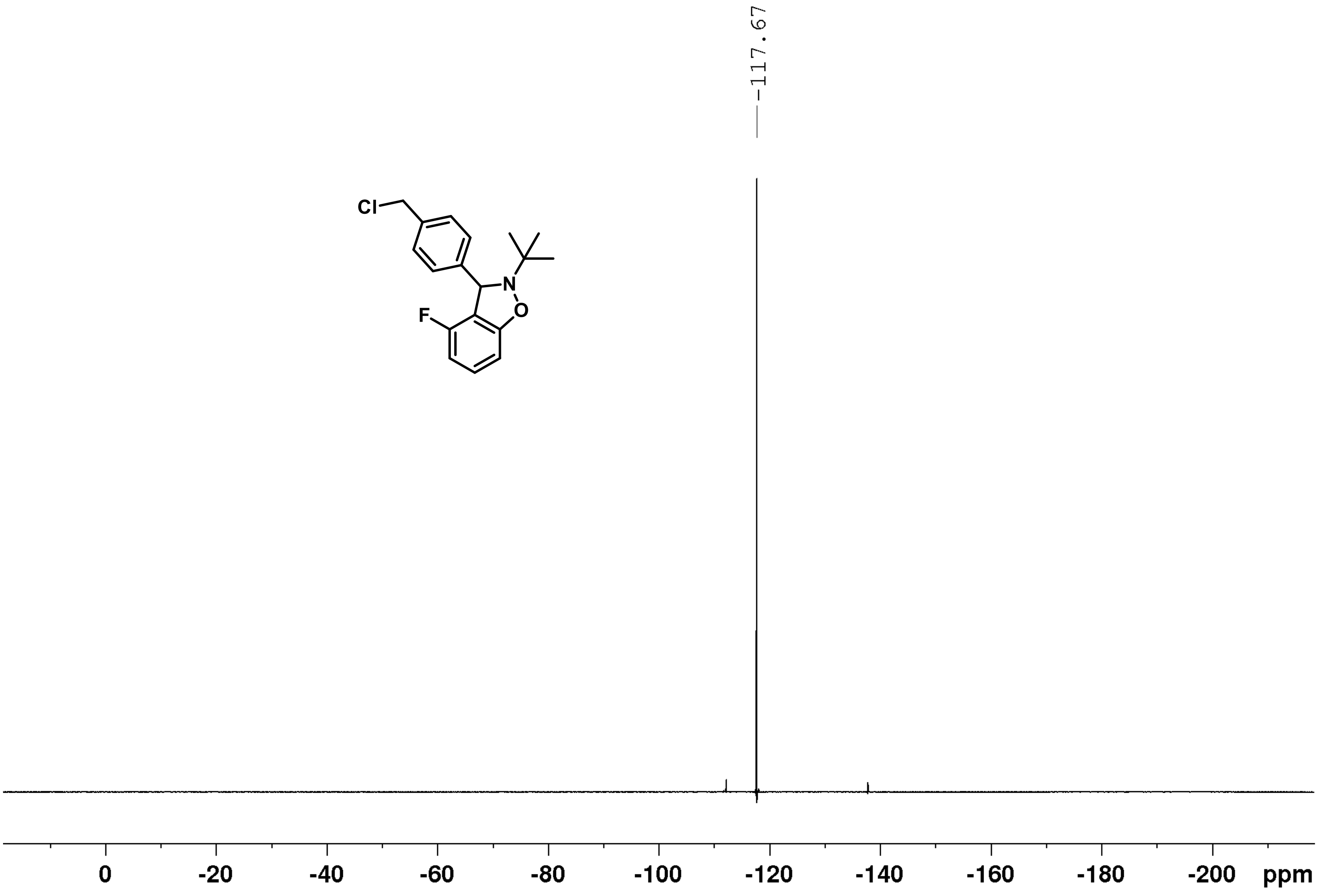

^{1}H-NMR of Compound 3bg in $CDCl_3$ at 400 MHz, 298K

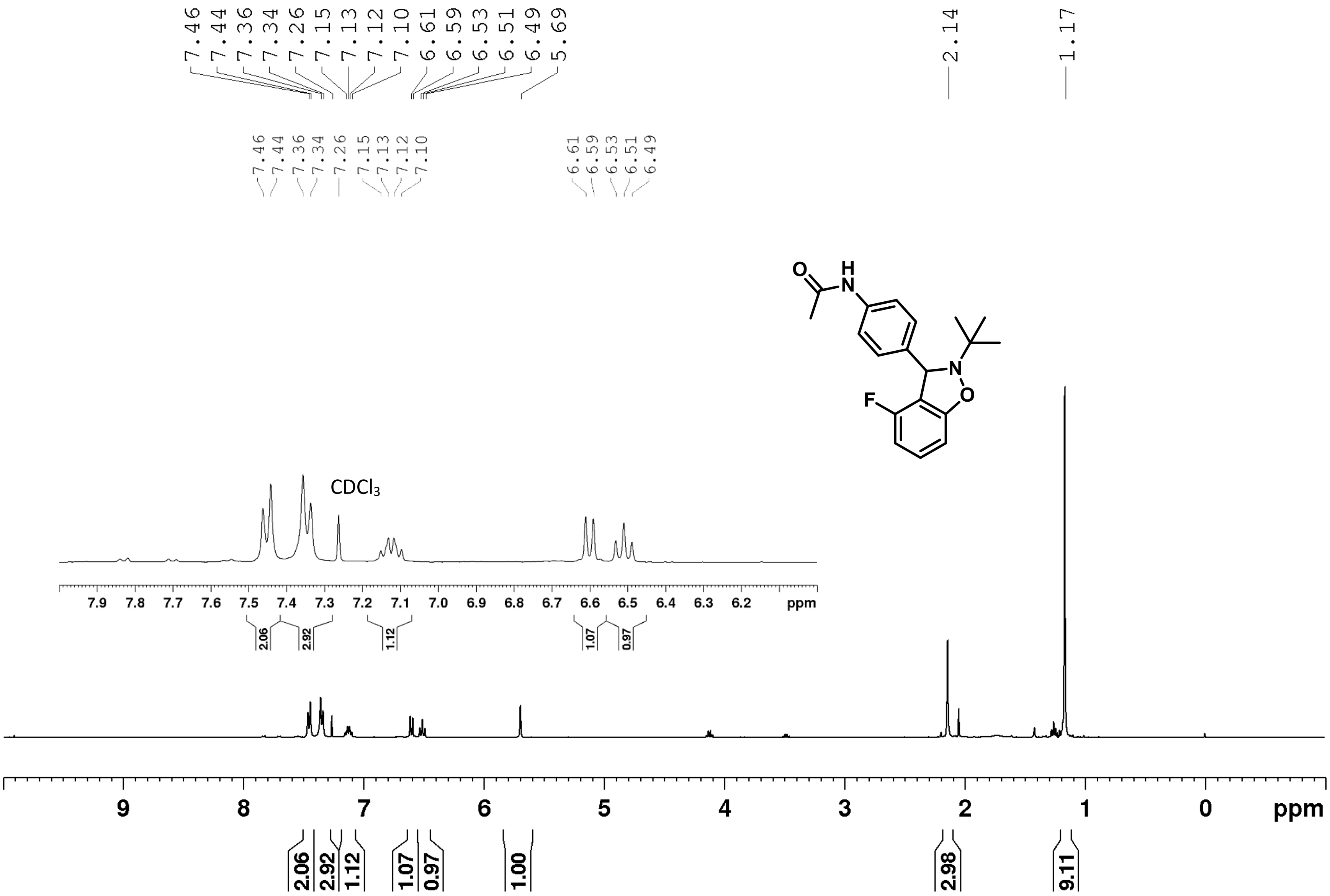

$^{13}C\{^{1}H\}$ NMR of Compound 3bg in $CDCl_3$ at 100 MHz, 298K

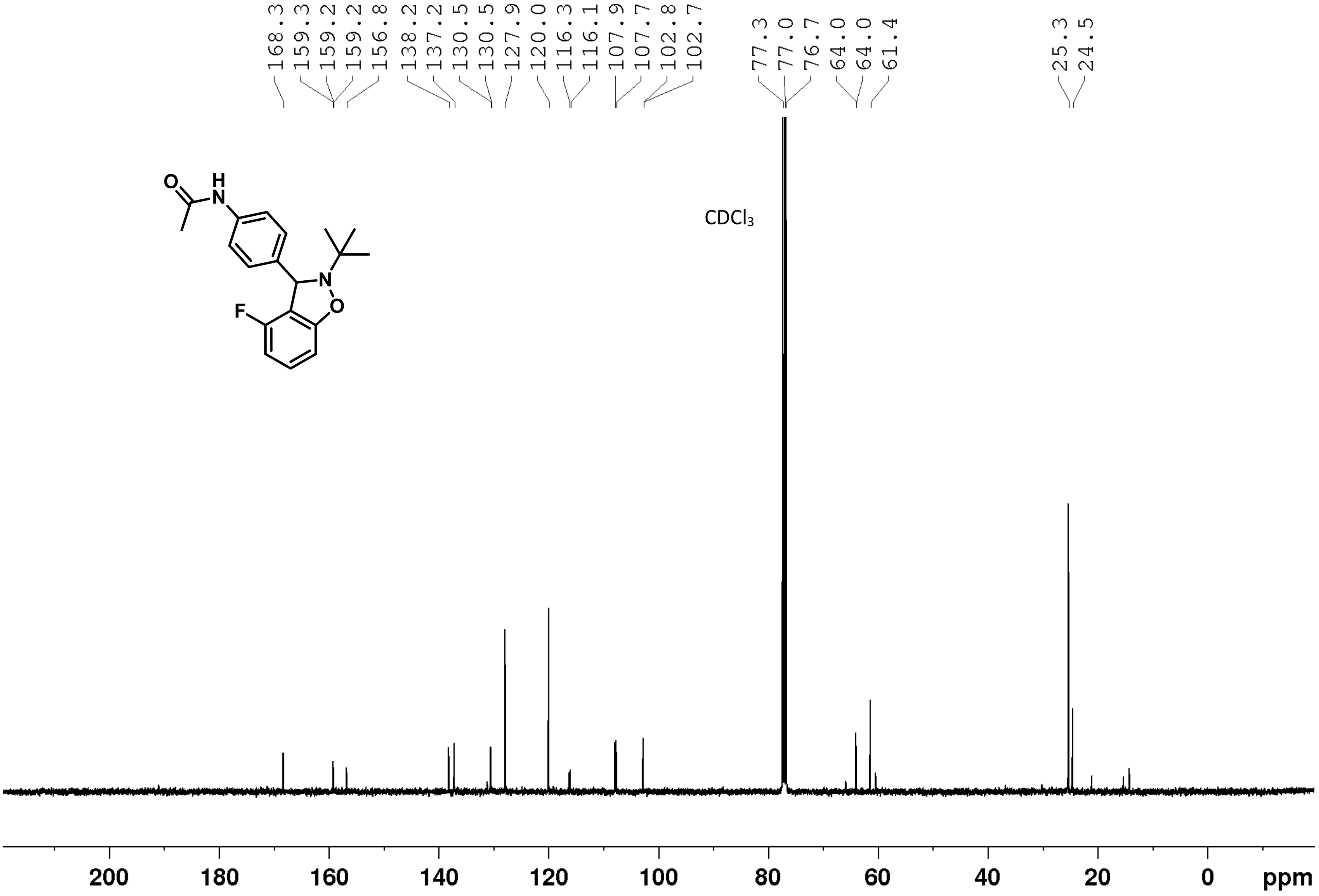

$^{19}F\{^1H\}$ NMR of Compound 3bg in $CDCl_3$ at 376 MHz, 298K

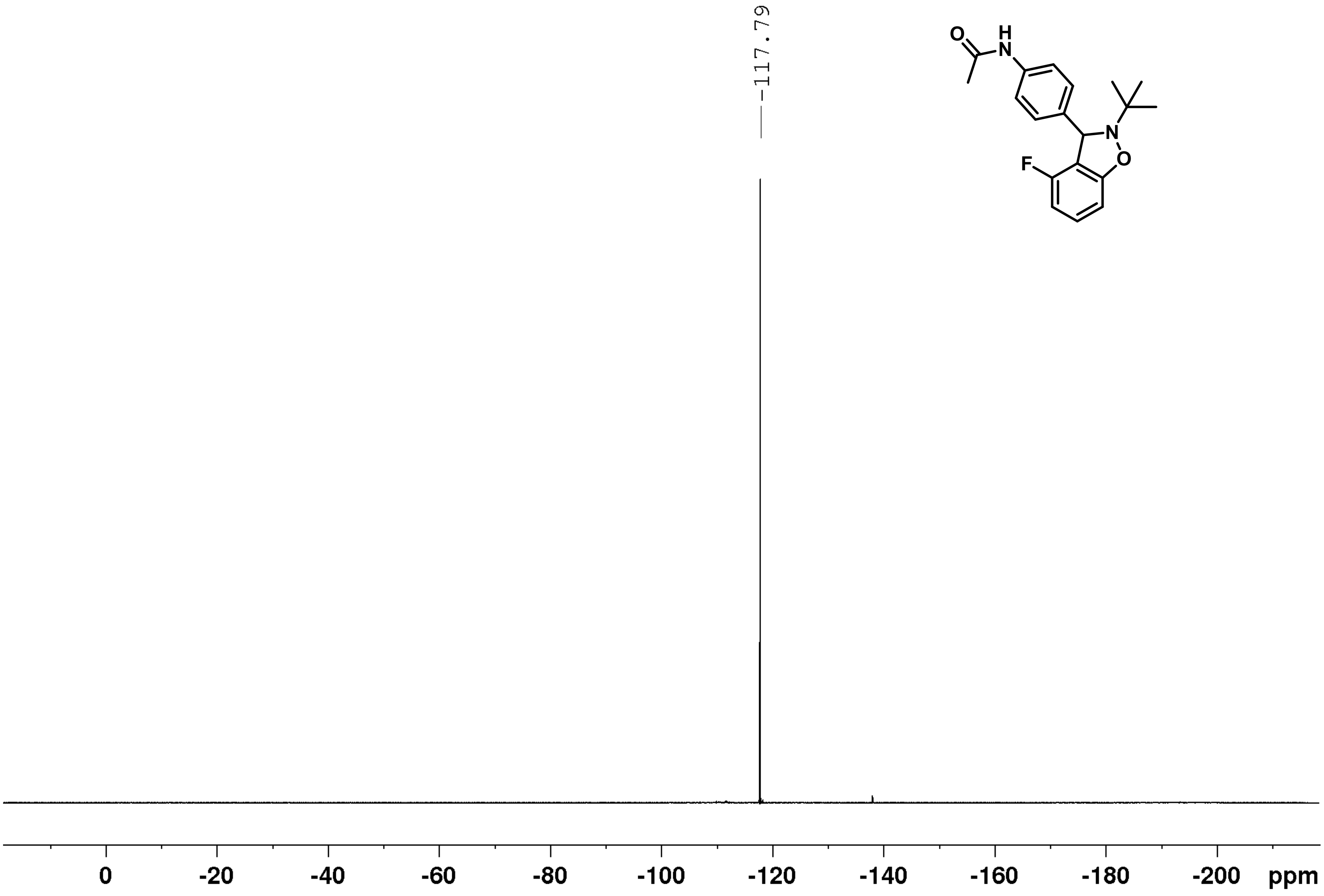

^{1}H-NMR of Compound 3bh in $CDCl_3$ at 400 MHz, 298K

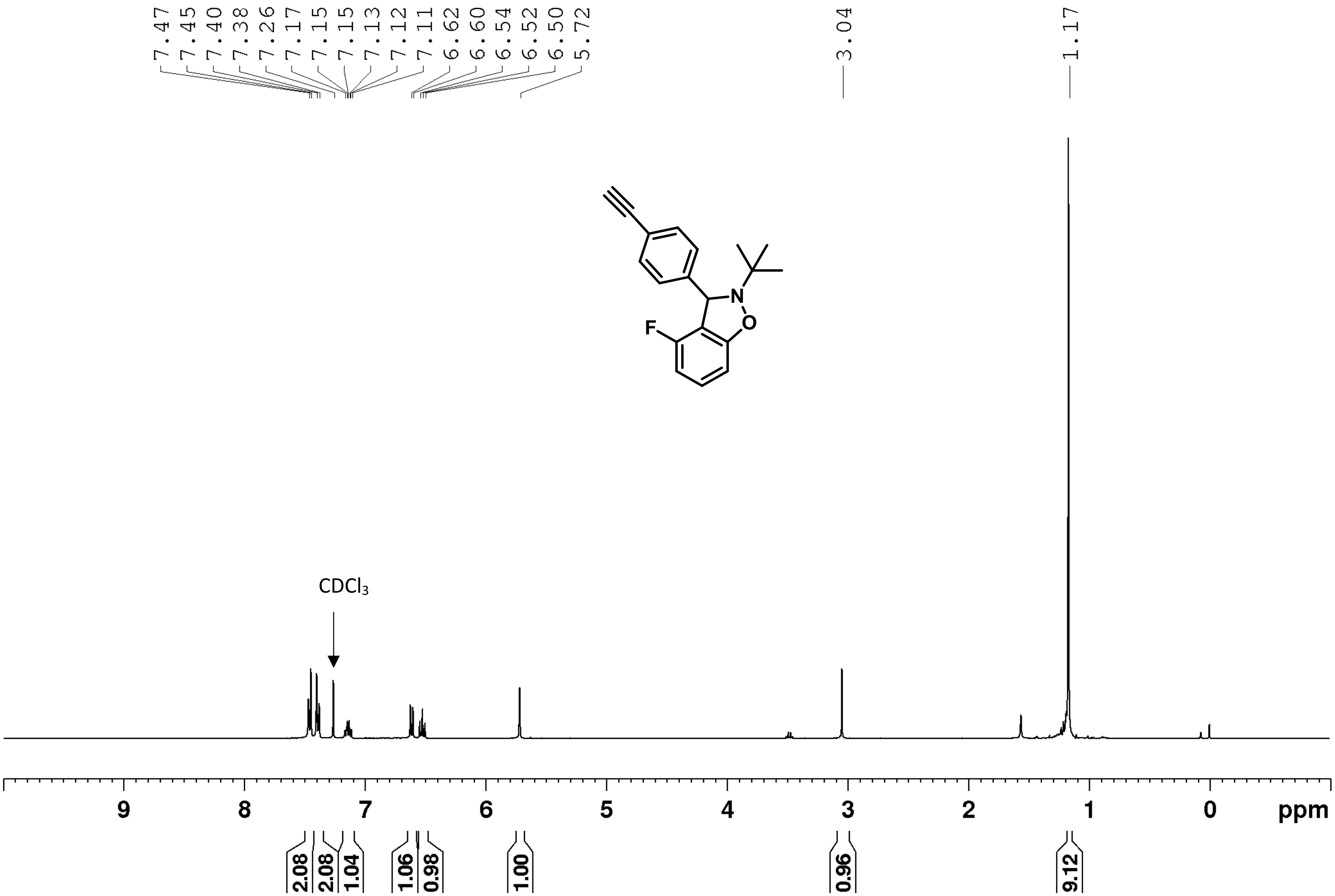

$^{13}C\{^1H\}$ NMR of Compound 3bh in $CDCl_3$ at 100 MHz, 298K

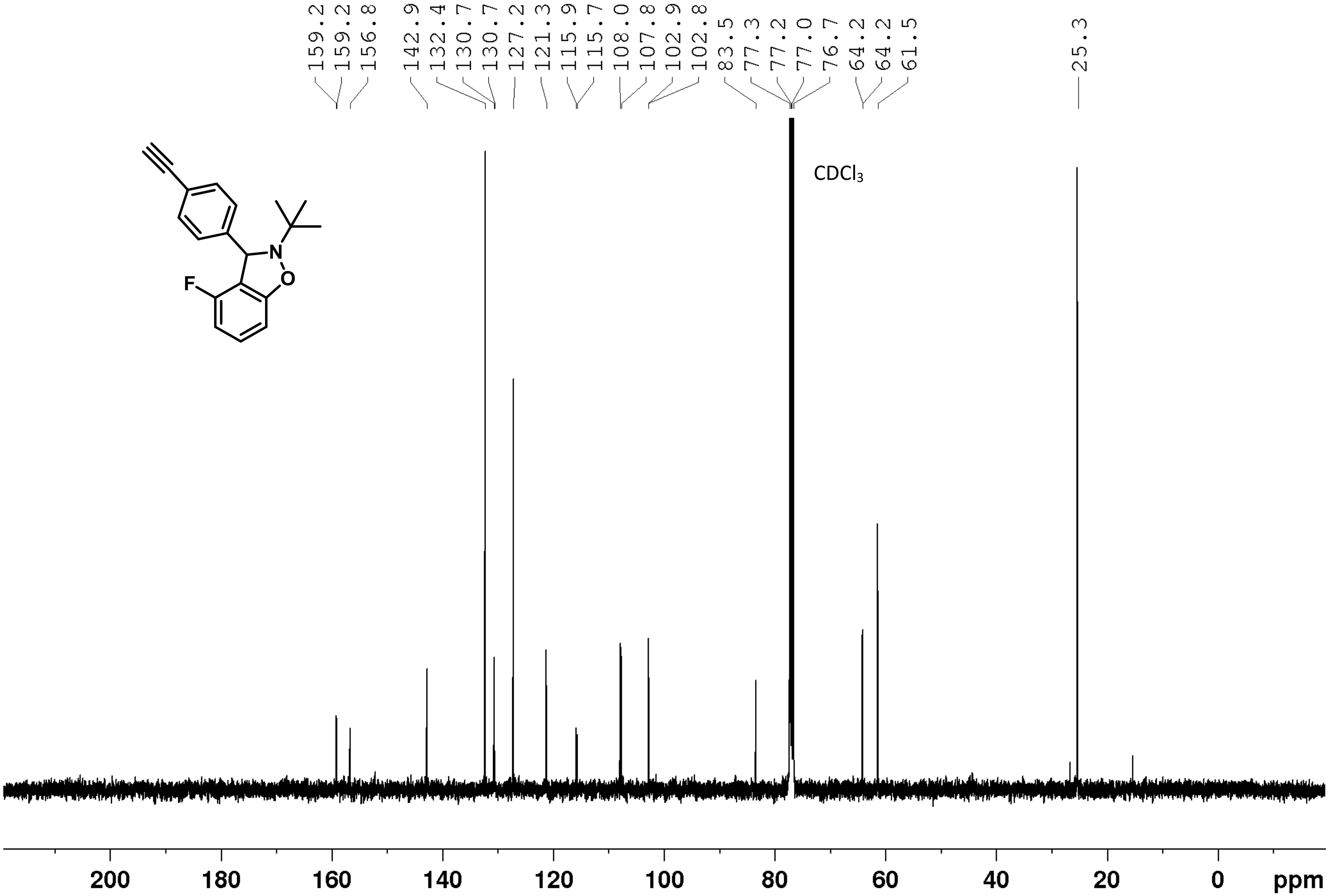

$^{19}F\{^{1}H\}$ NMR of Compound 3bh in $CDCl_3$ at 376 MHz, 298K

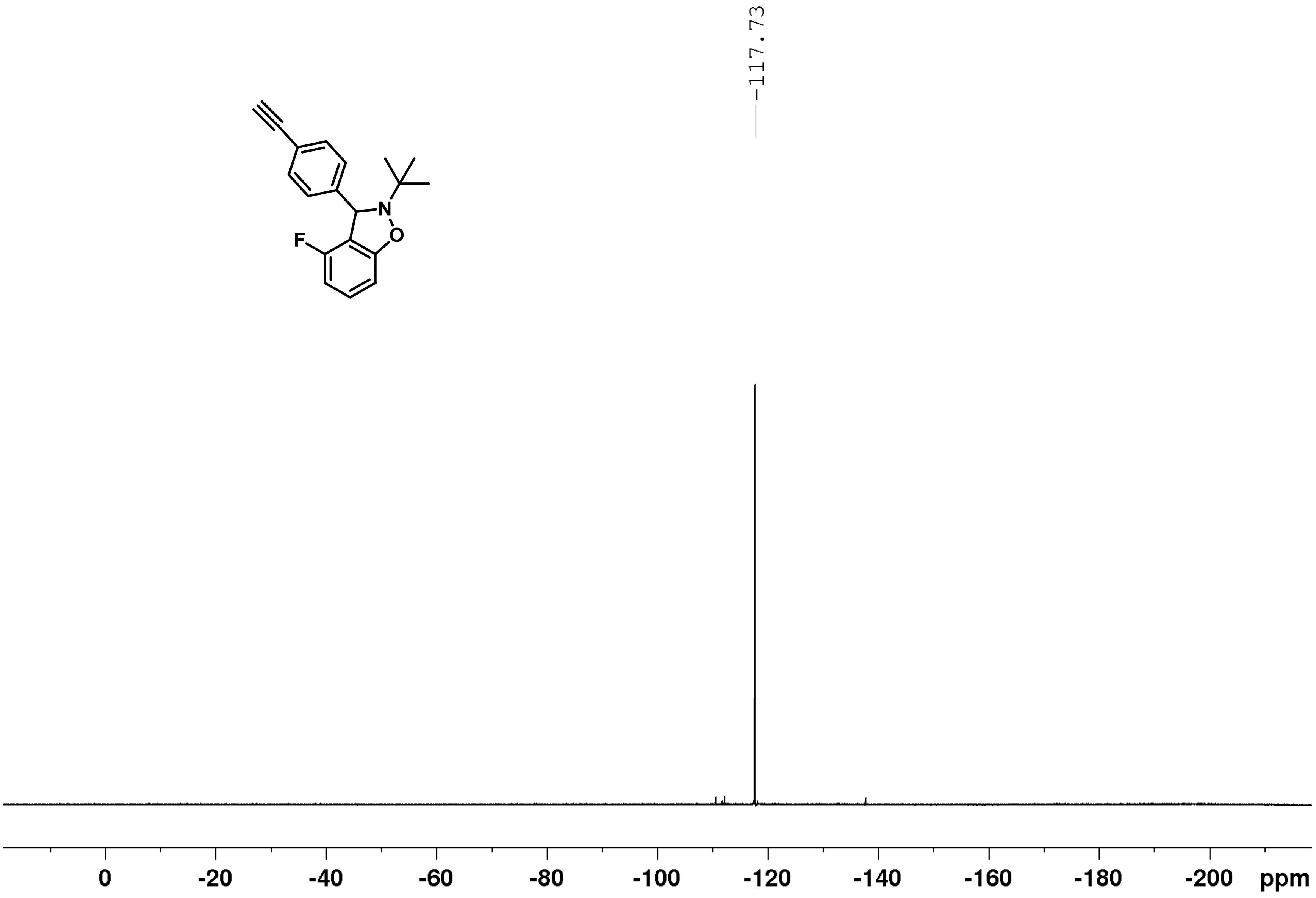

^{1}H-NMR of Compound 3bi in $CDCl_3$ at 400 MHz, 298K

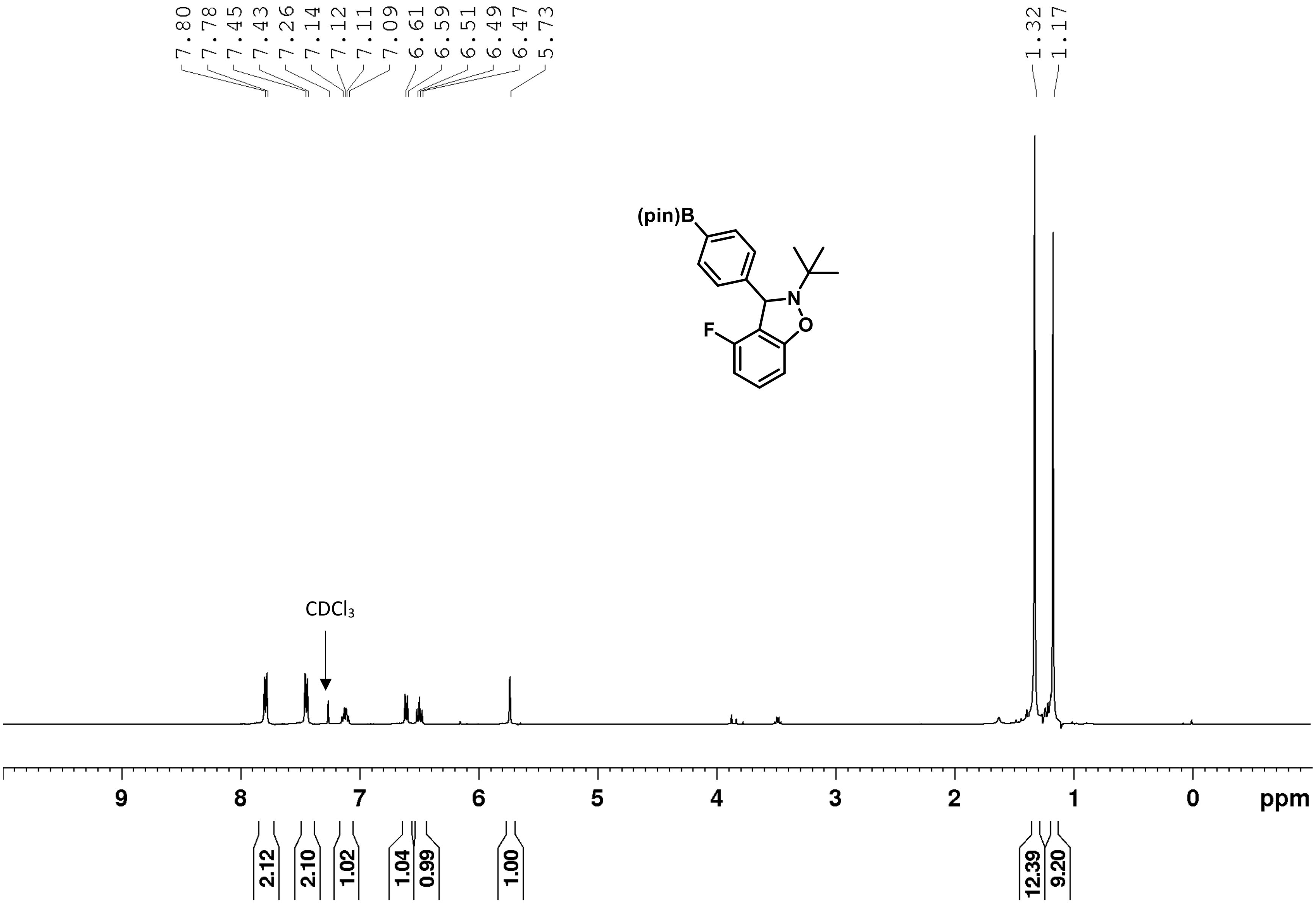

$^{13}C\{^{1}H\}$ NMR of Compound 3bi in $CDCl_3$ at 100 MHz, 298K

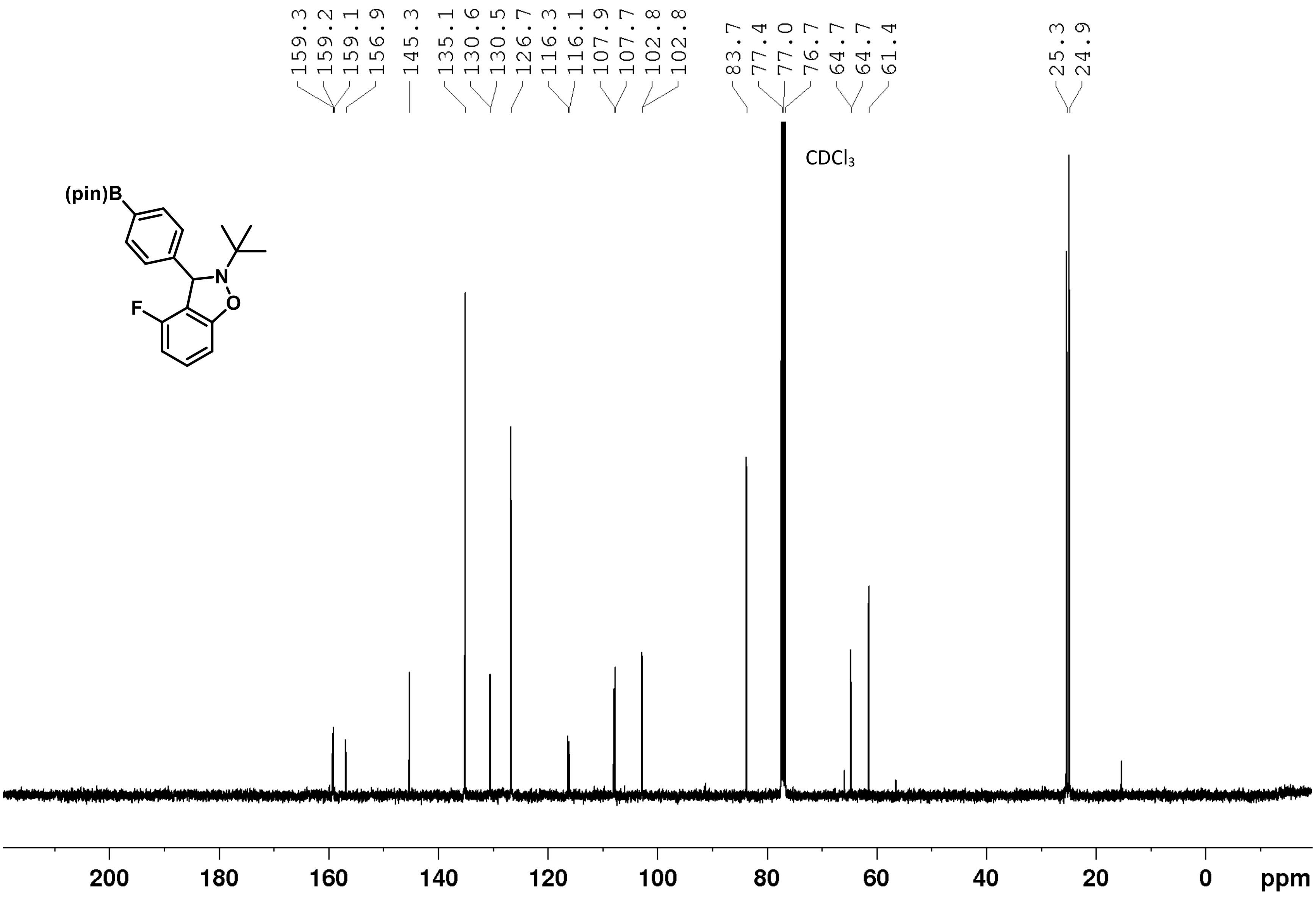

$^{19}F\{^1H\}$ NMR of Compound 3bi in $CDCl_3$ at 376 MHz, 298K

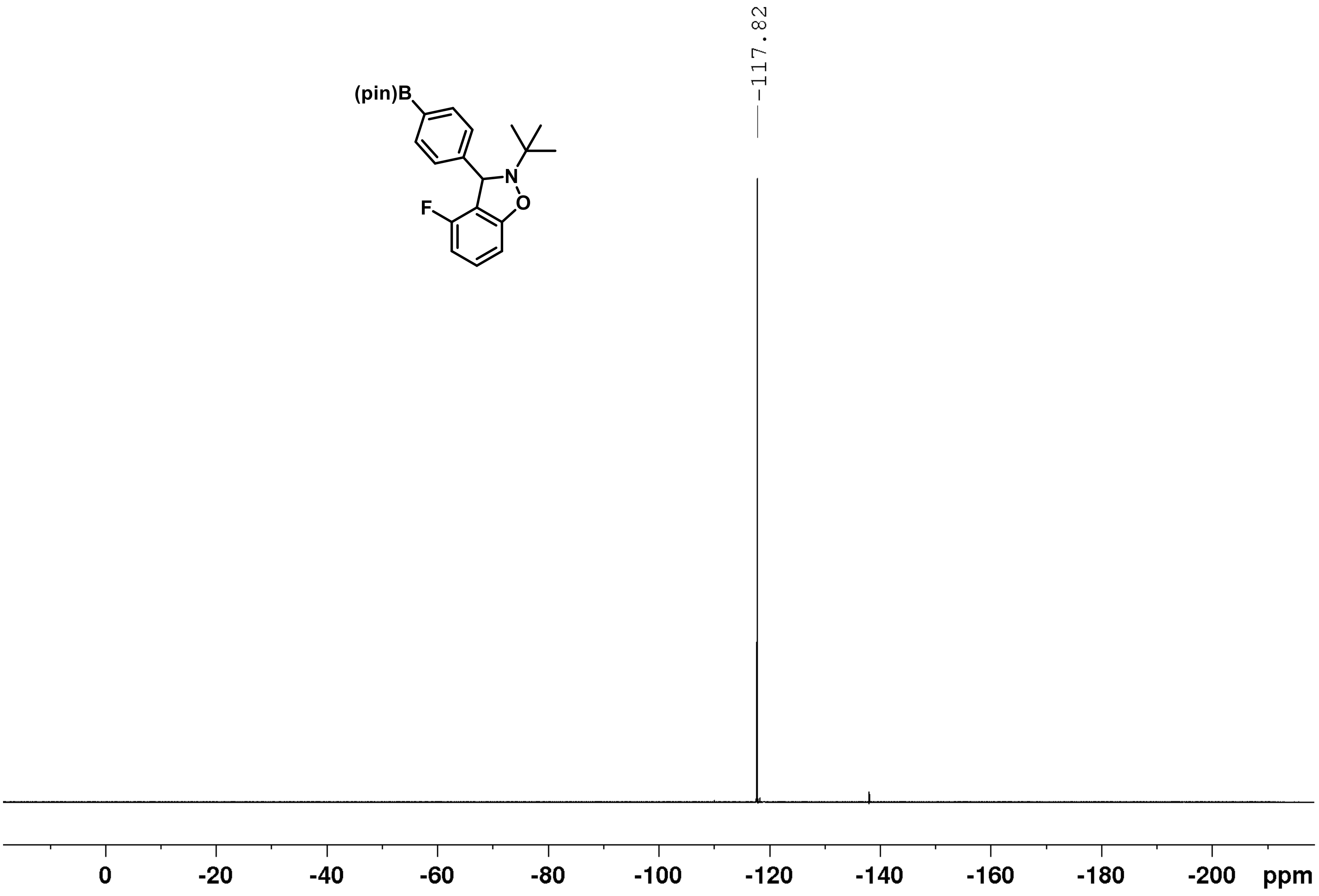

^{1}H-NMR of Compound 3bj in $CDCl_3$ at 400 MHz, 298K

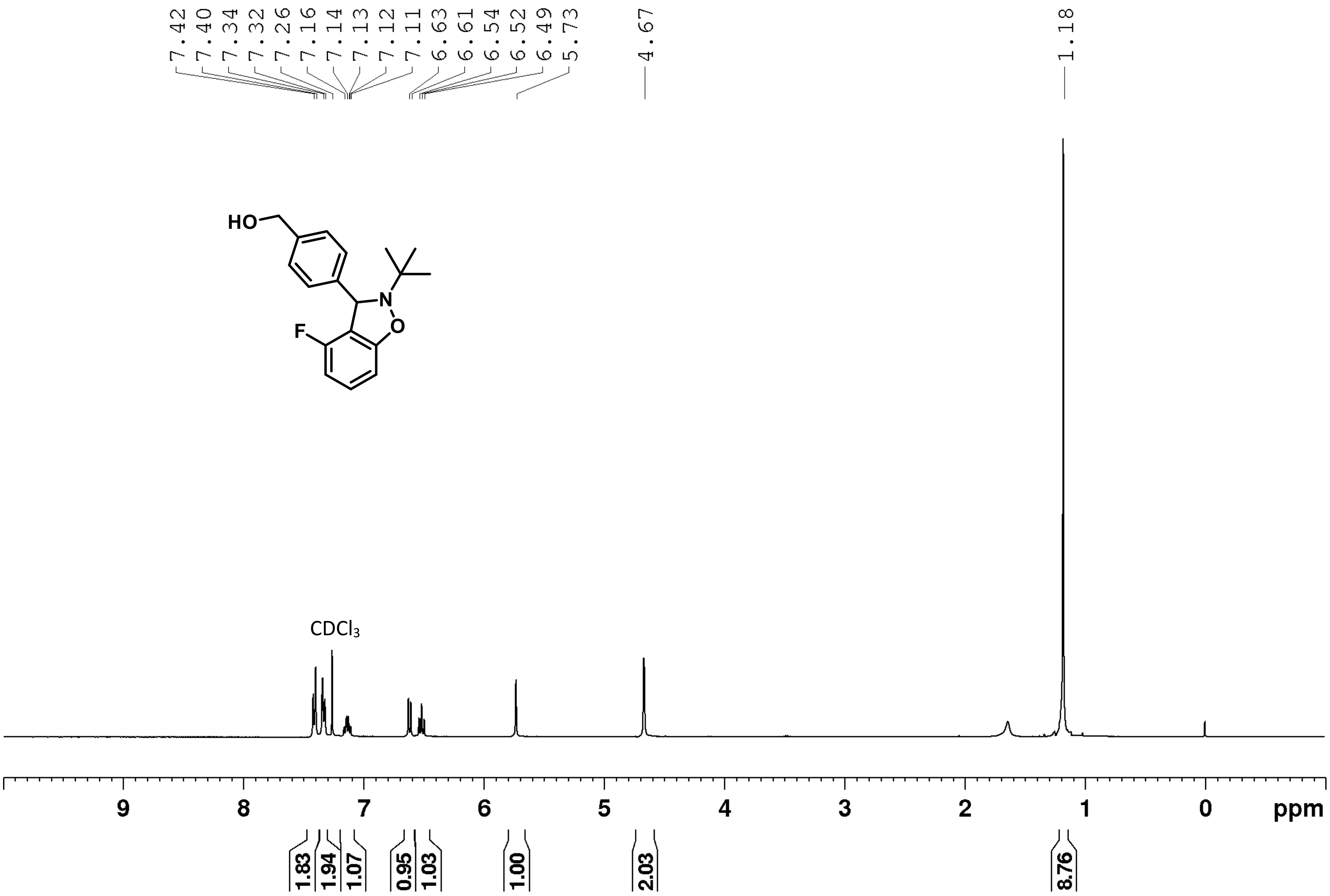

$^{13}C\{^{1}H\}$ NMR of Compound 3bj in $CDCl_3$ at 100 MHz, 298K

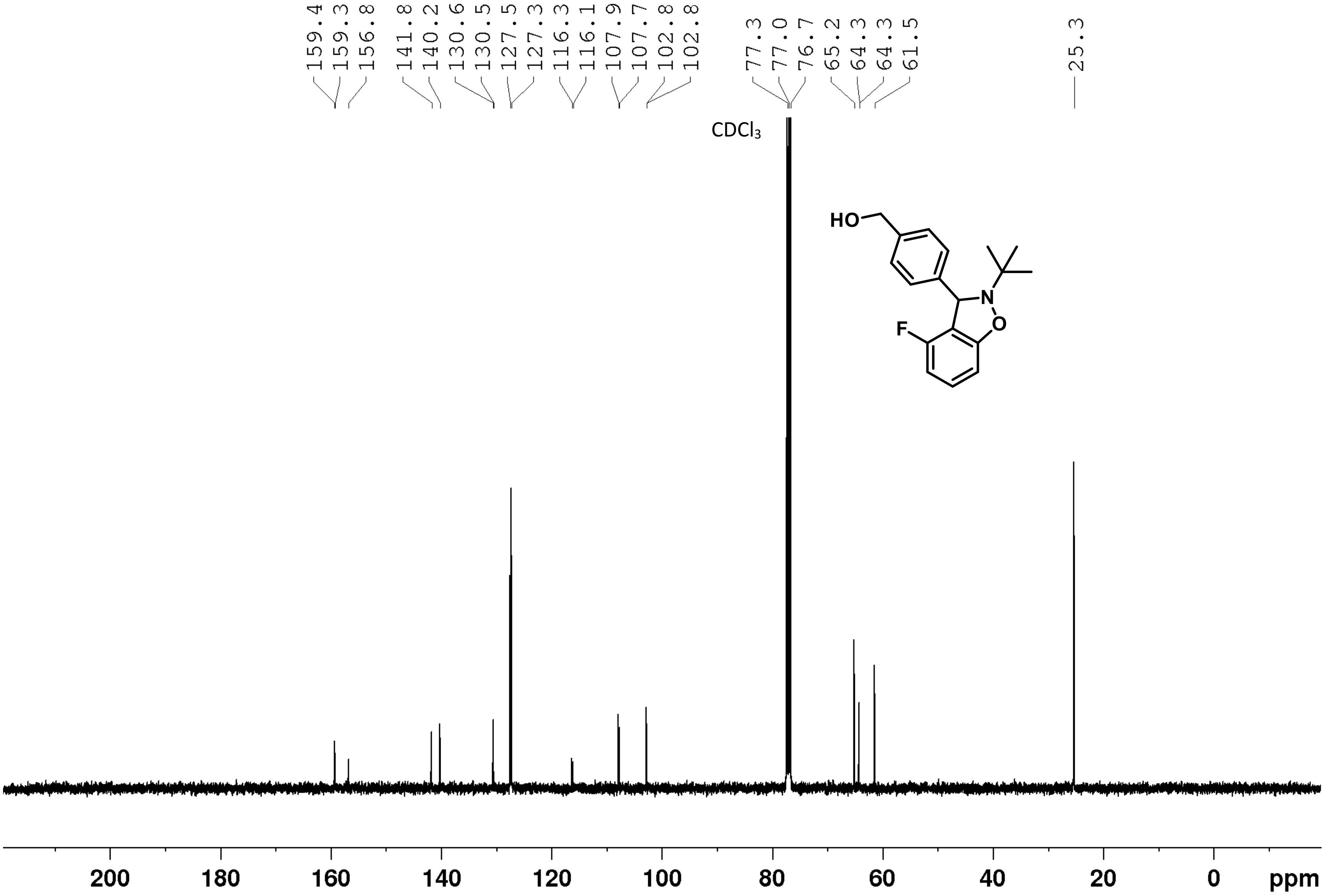

$^{19}F\{^1H\}$ NMR of Compound 3bj in $CDCl_3$ at 376 MHz, 298K

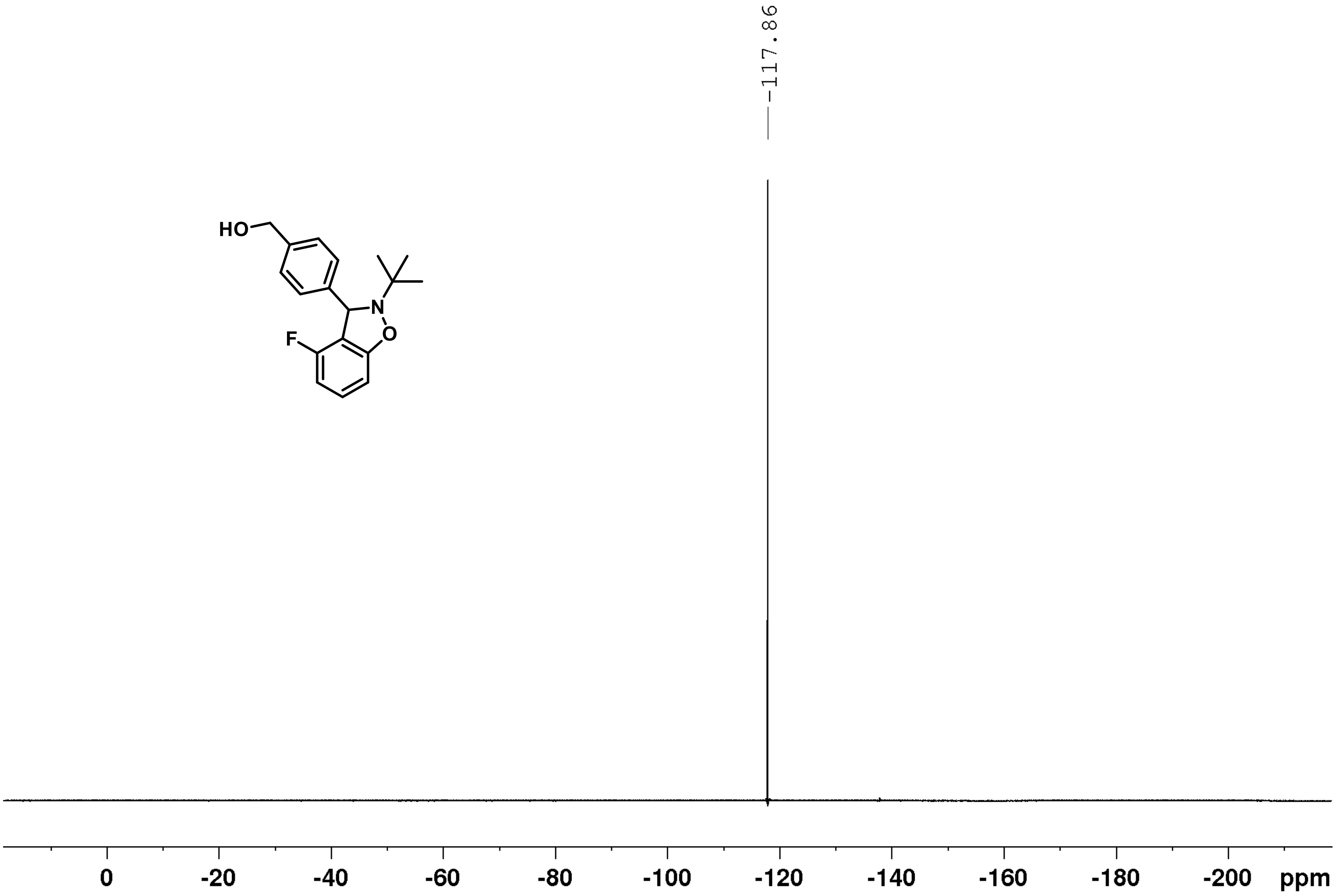

^{1}H-NMR of Compound 3mc in $CDCl_3$ at 400 MHz, 298K

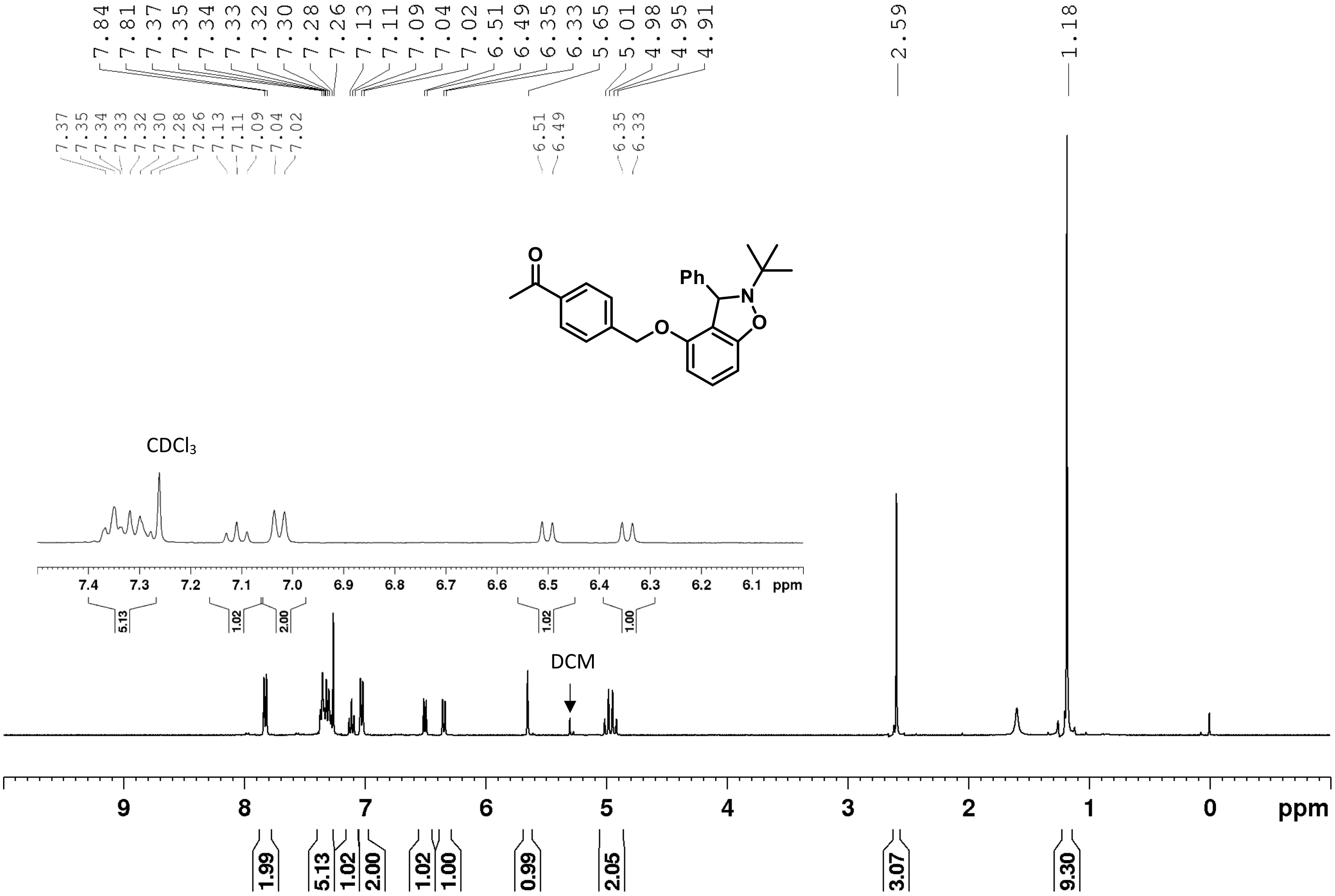

$^{13}C\{^{1}H\}$ NMR of Compound 3mc in $CDCl_3$ at 100 MHz, 298K

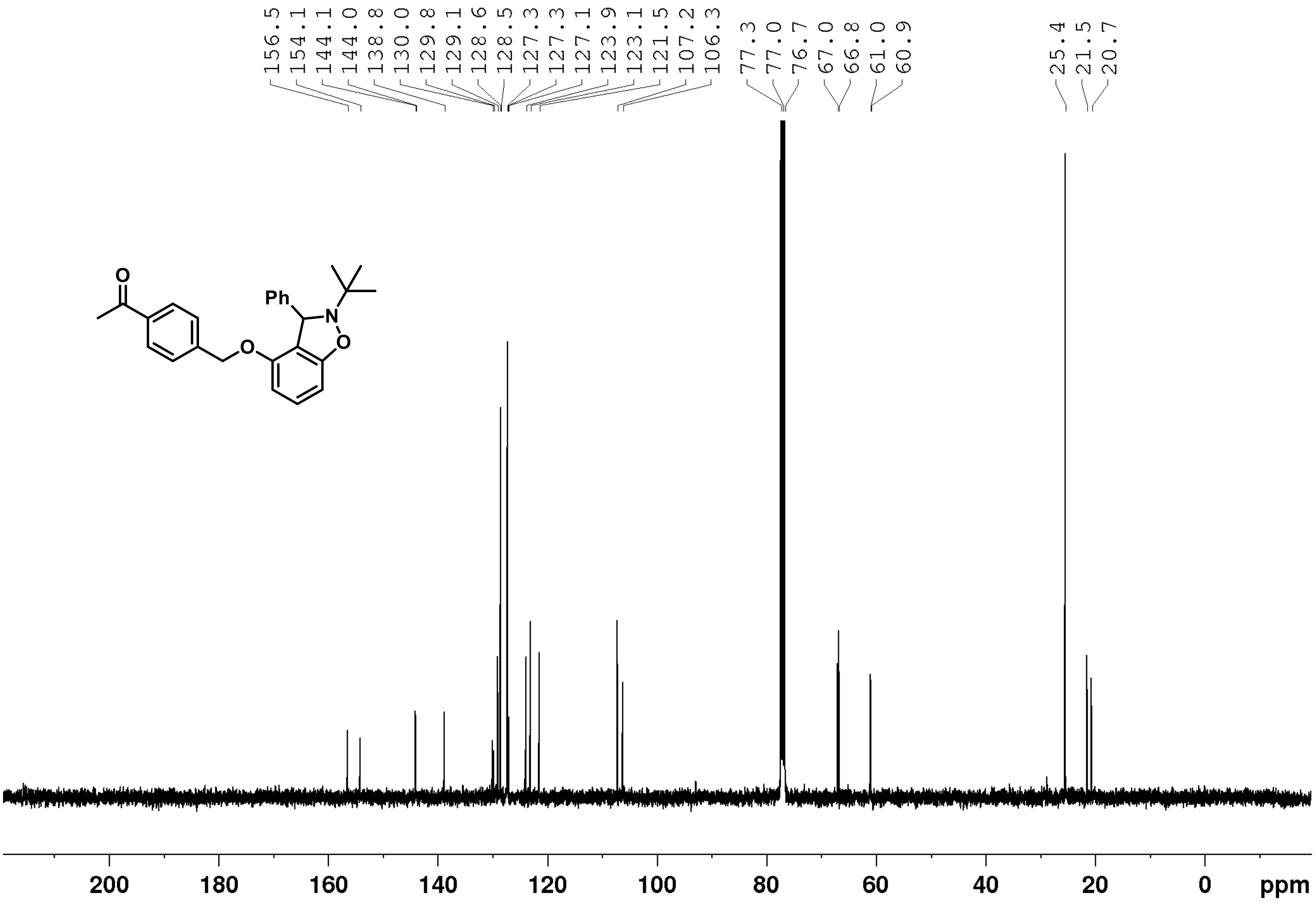

^{1}H-NMR of Compound 3nc in $CDCl_3$ at 400 MHz, 298K

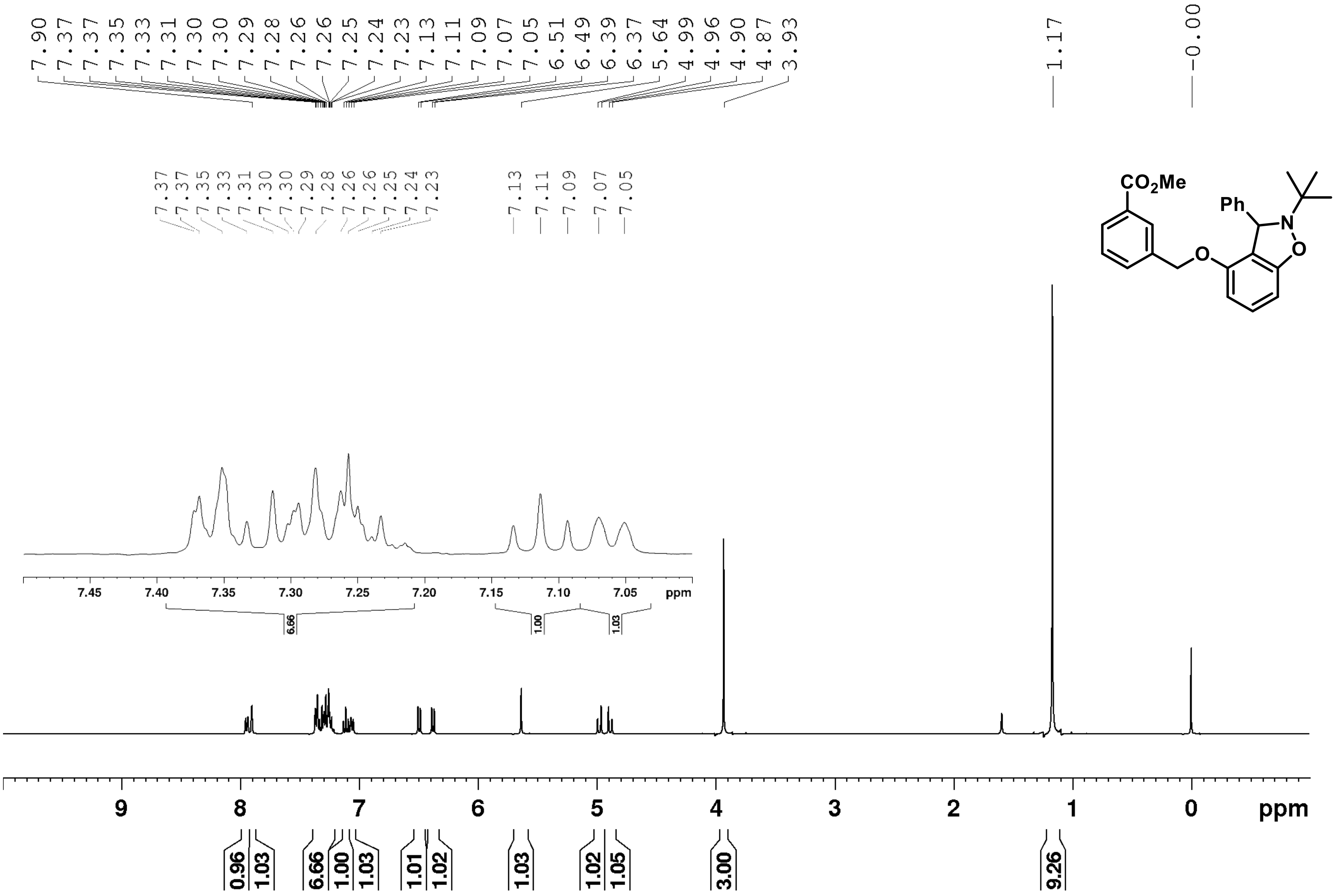

$^{13}C\{^1H\}$ NMR of Compound 3nc in $CDCl_3$ at 100 MHz, 298K

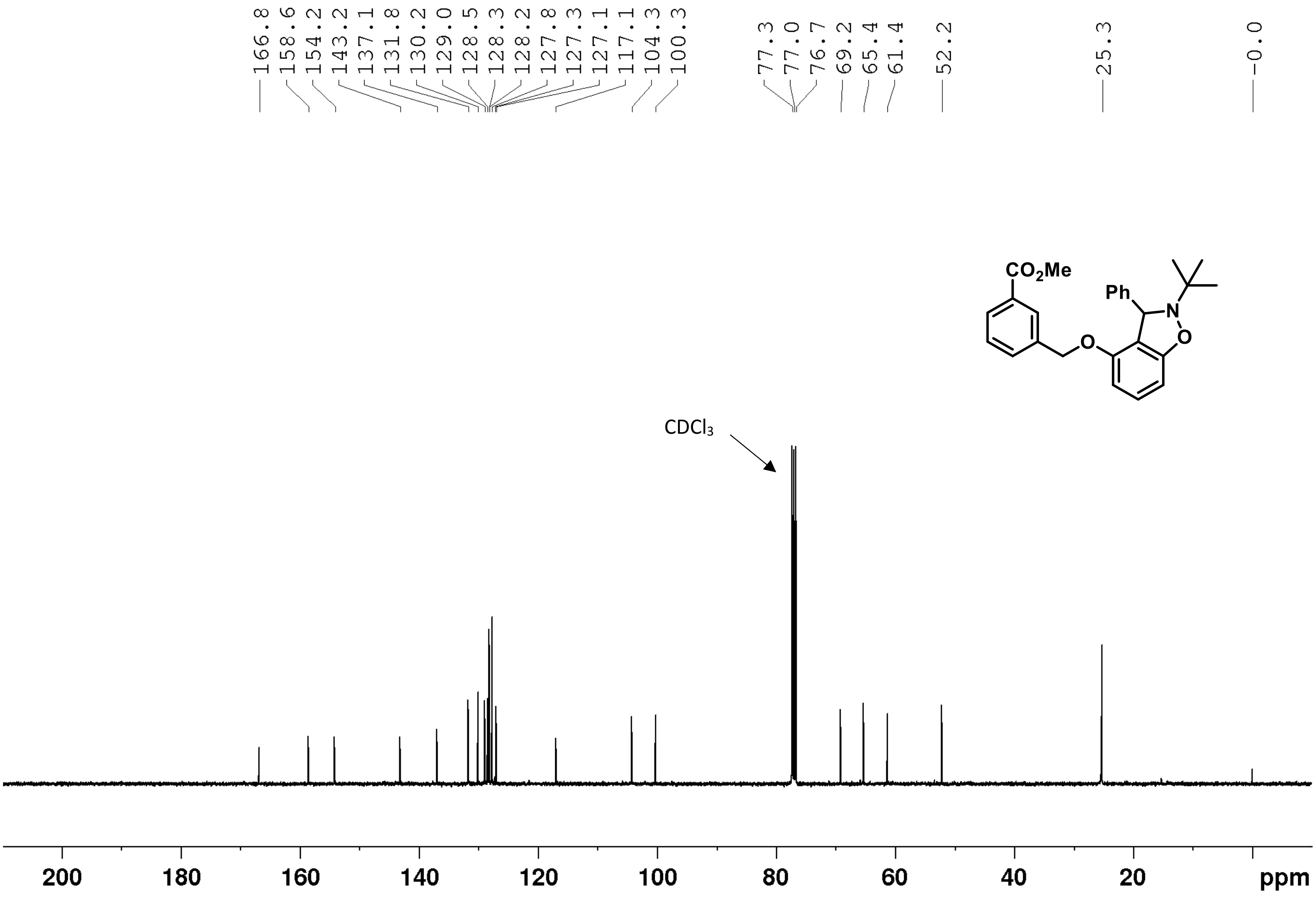

^{1}H-NMR of Compound 3mf in $CDCl_3$ at 400 MHz, 298K

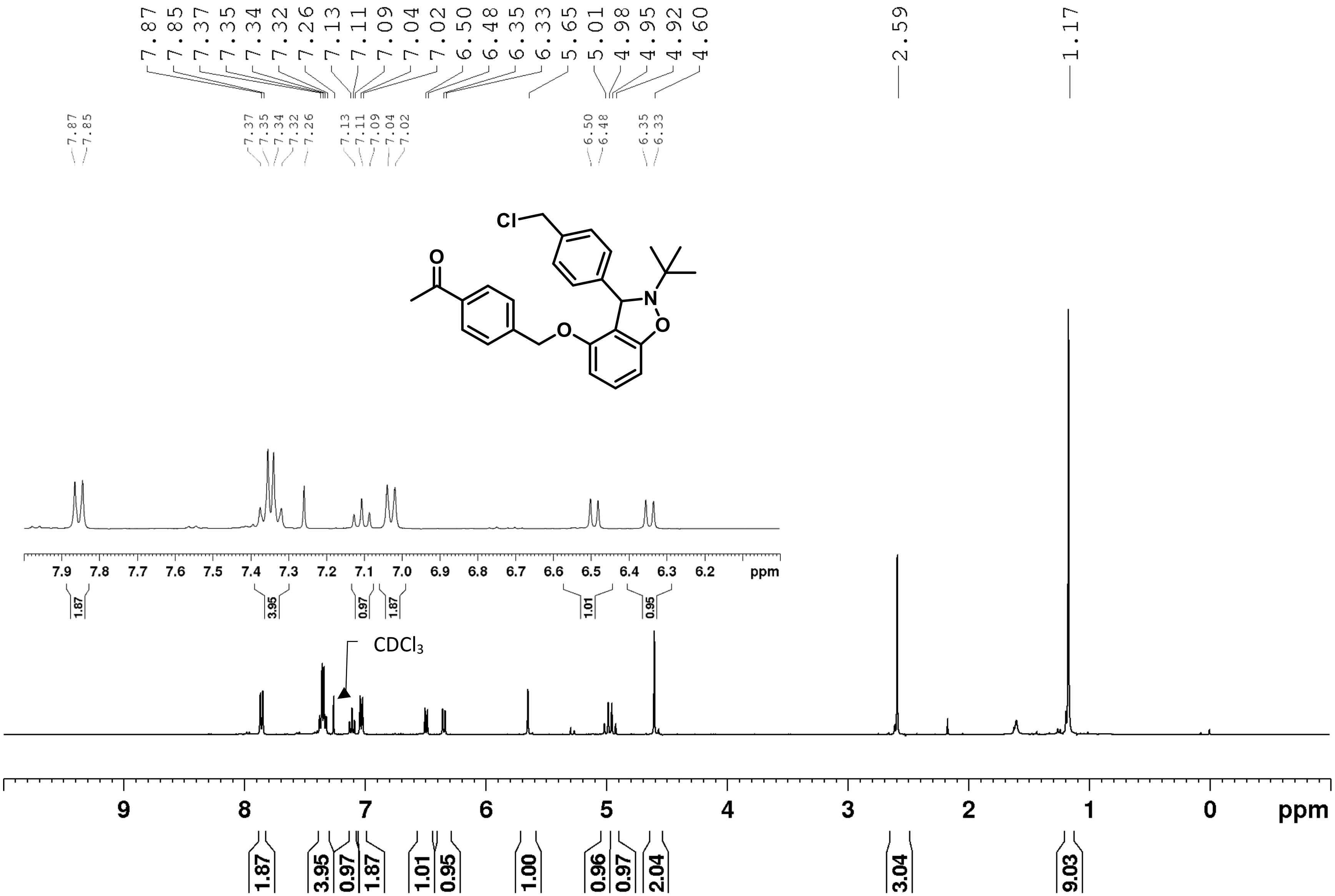

$^{13}C\{^1H\}$ NMR of Compound 3mf in $CDCl_3$ at 100 MHz, 298K

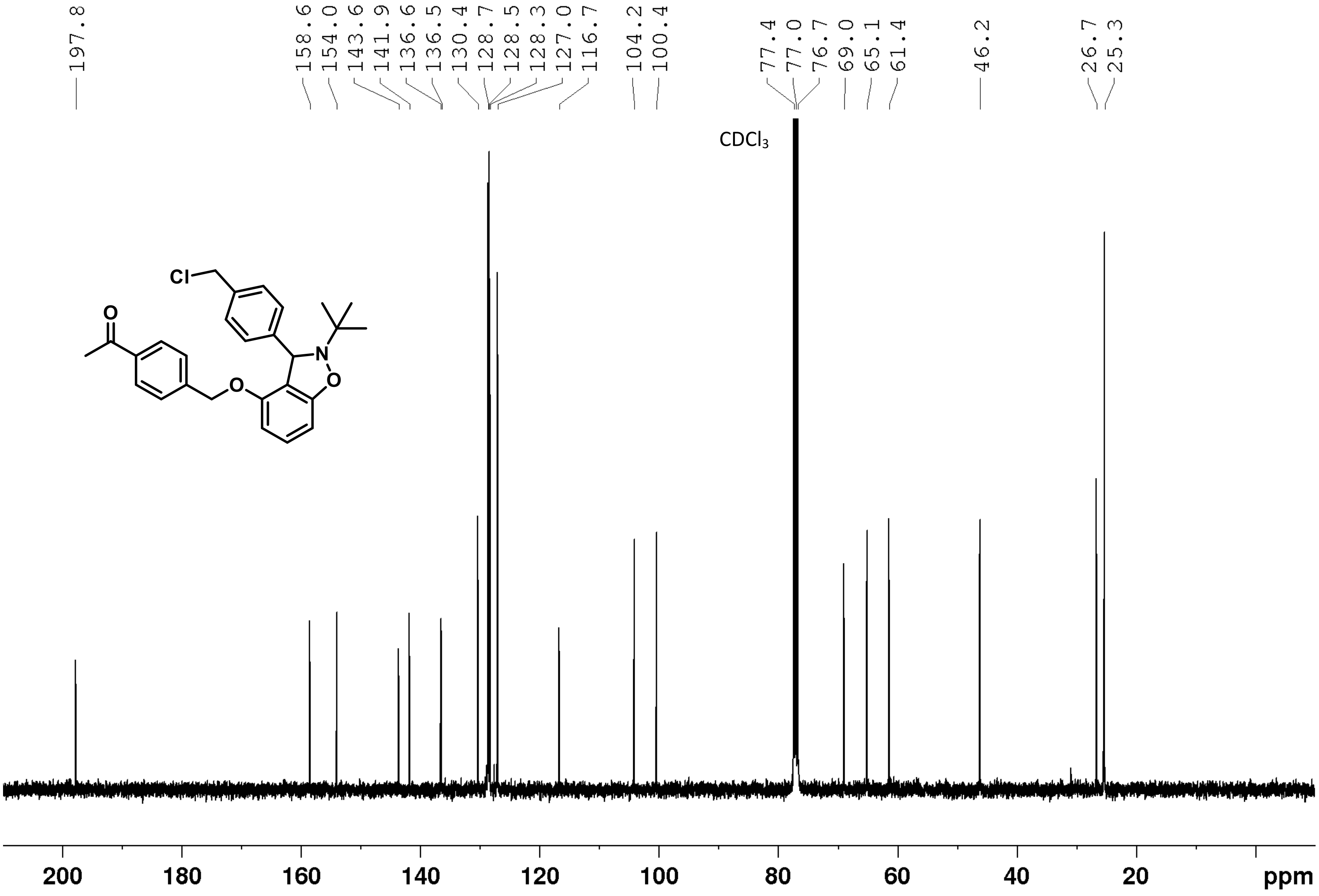

^{1}H-NMR of Compound 3bl in $CDCl_3$ at 400 MHz, 298K

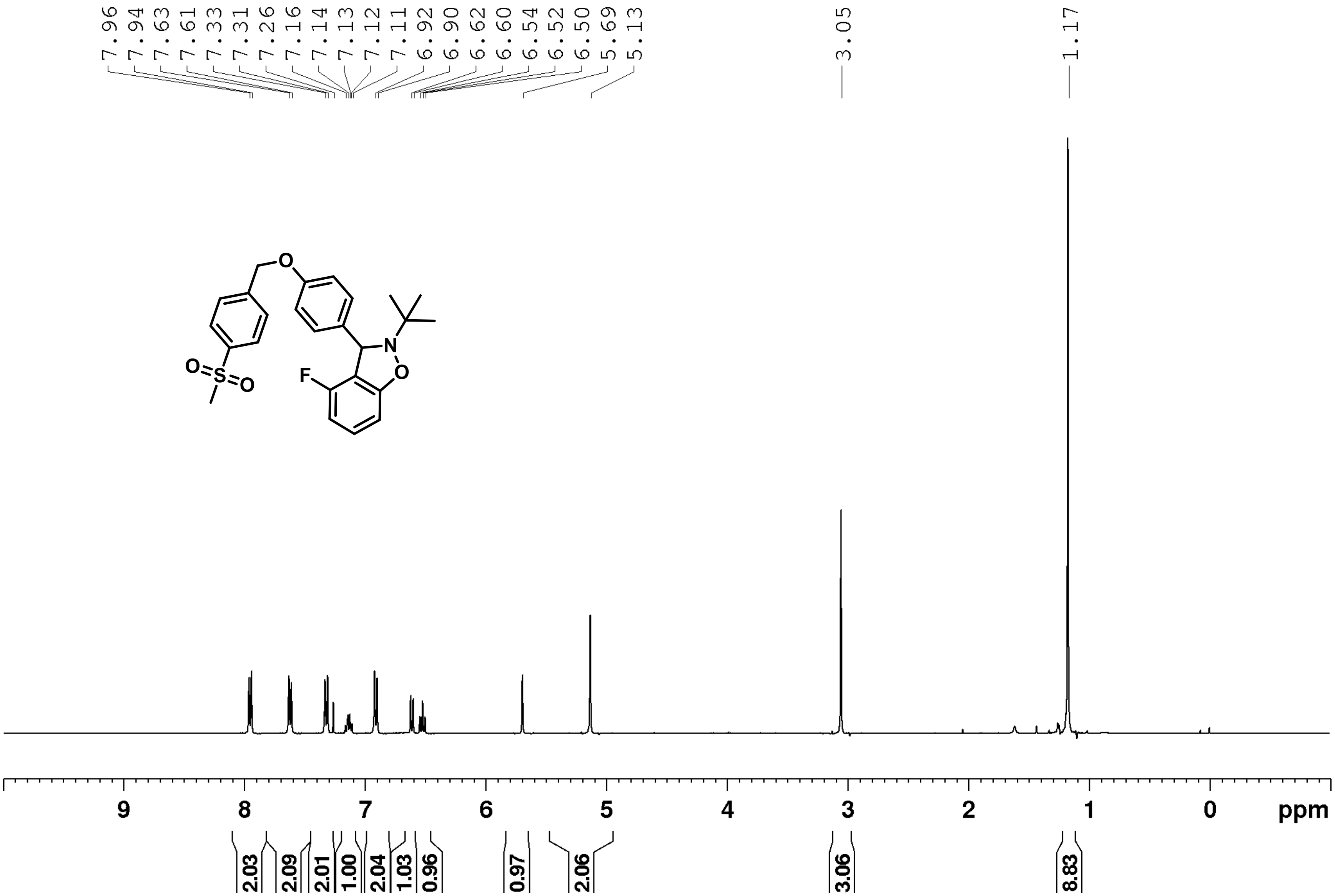

$^{13}C\{^1H\}$ NMR of Compound 3bl in $CDCl_3$ at 100 MHz, 298K

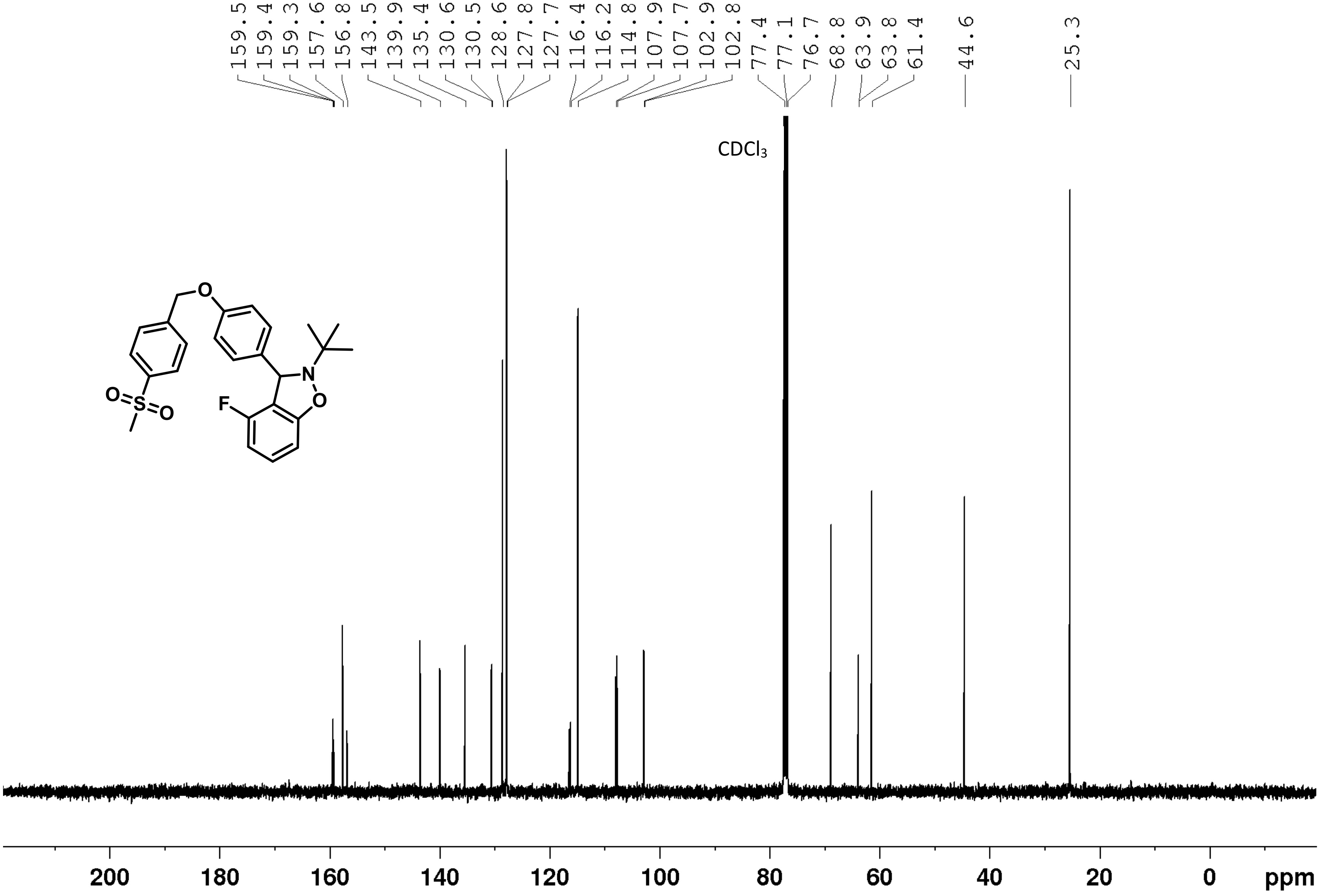

$^{19}F\{^1H\}$ NMR of Compound 3bl in $CDCl_3$ at 376 MHz, 298K

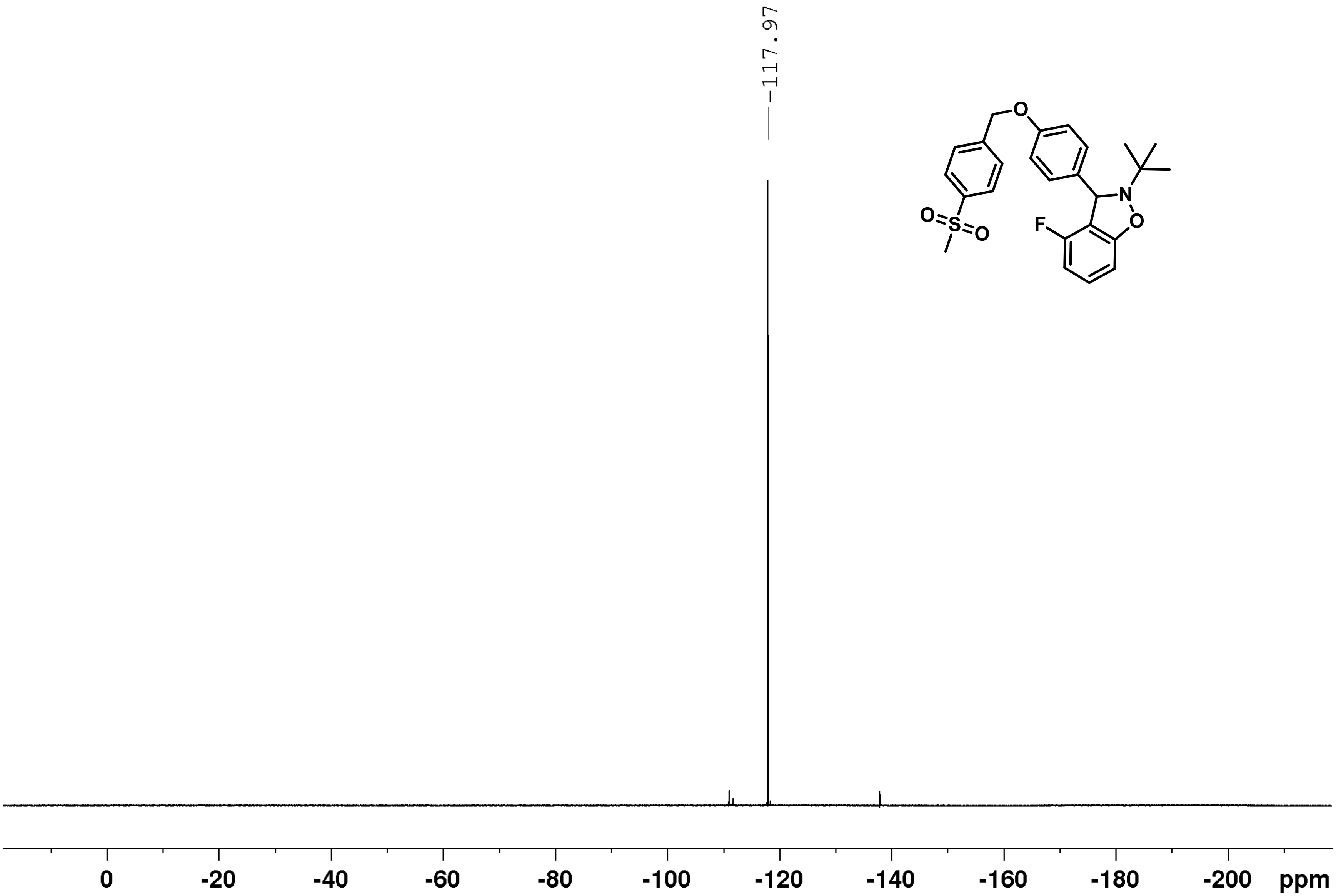

^{1}H NMR of Compound 3bk in $CDCl_3$ at 400 MHz, 298K

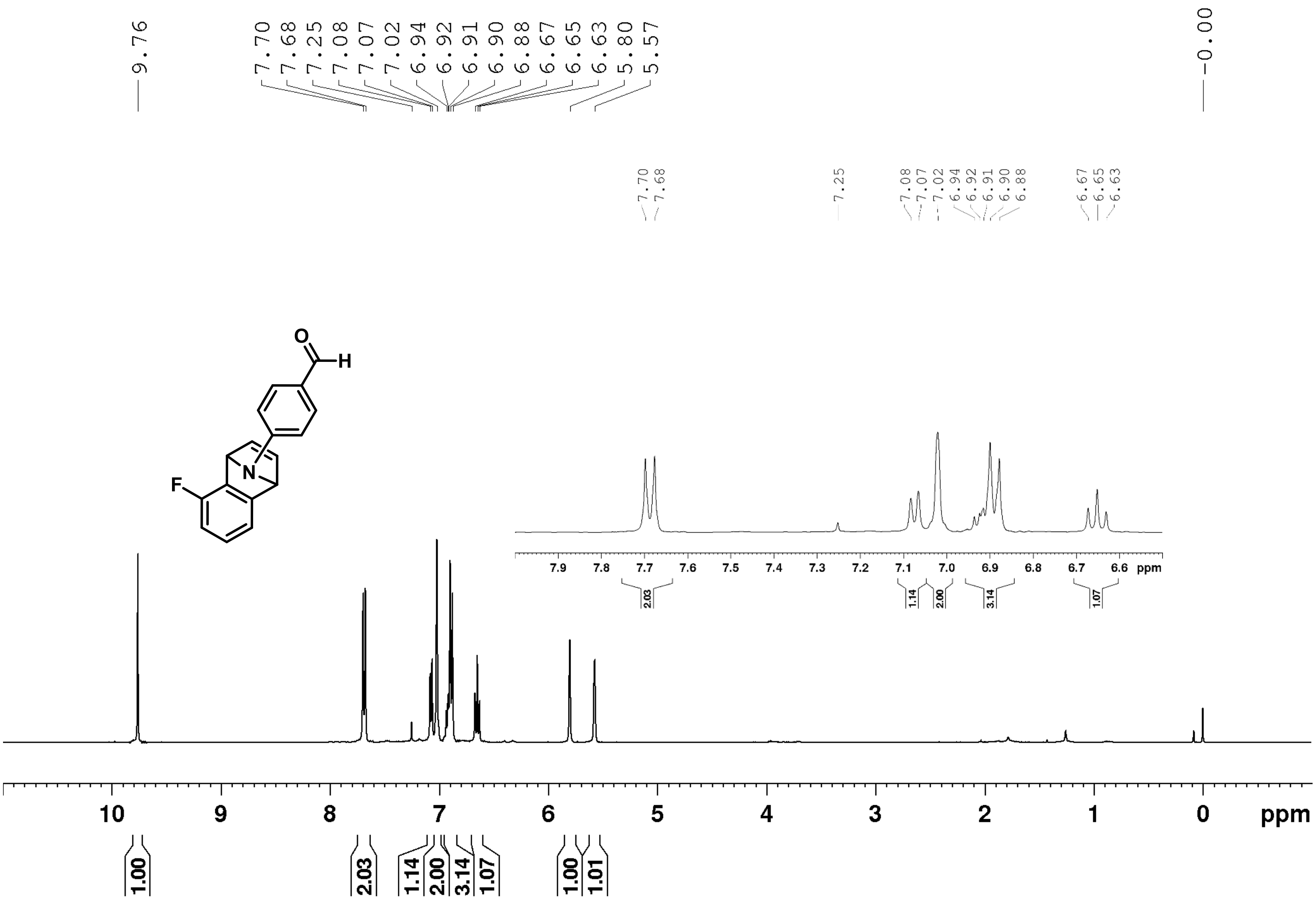

$^{13}C\{^1H\}$ NMR of Compound 3bk in $CDCl_3$ at 100 MHz, 298K

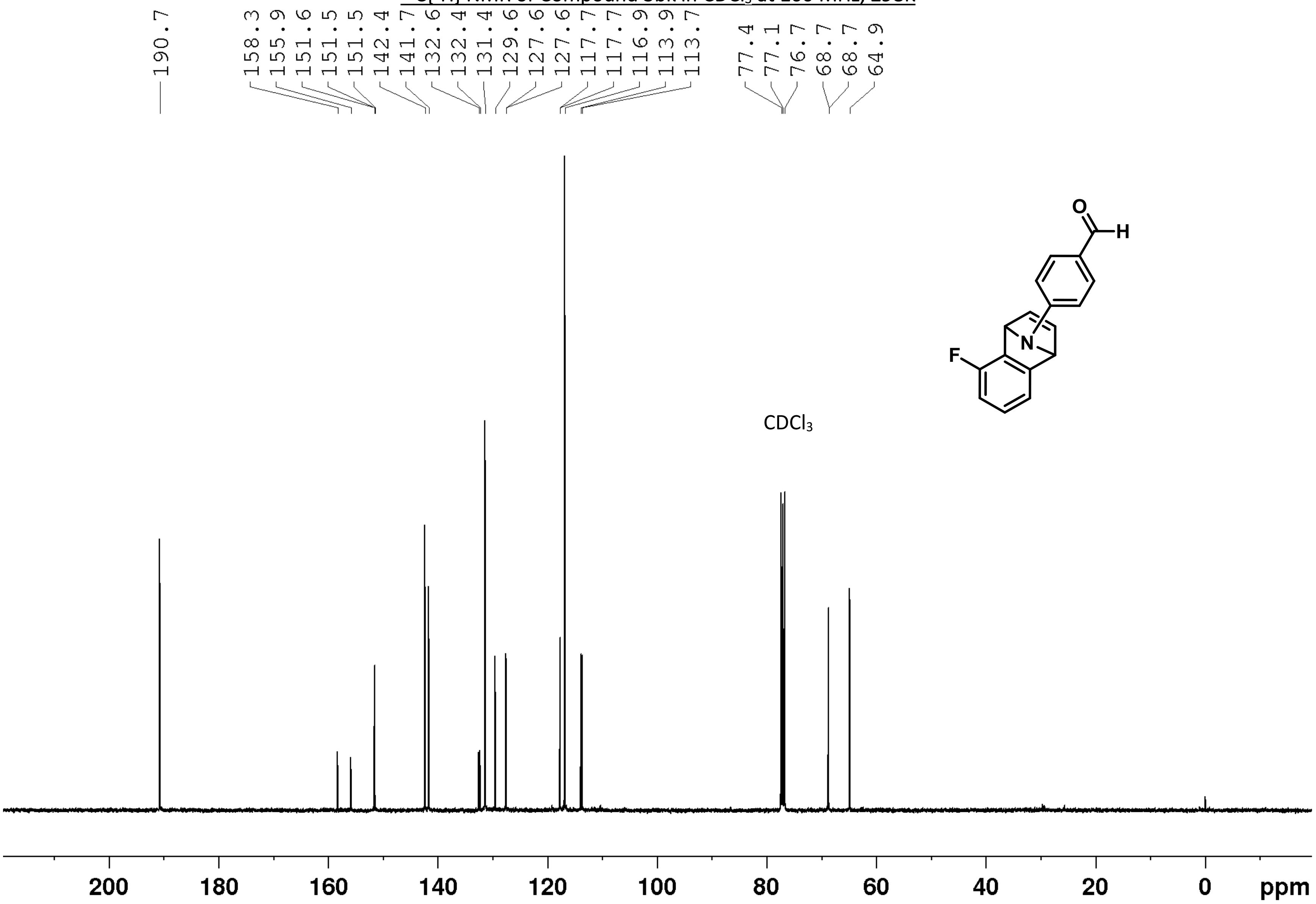

$^{19}F\{^1H\}$ NMR of Compound 3bk in $CDCl_3$ at 376 MHz, 298K

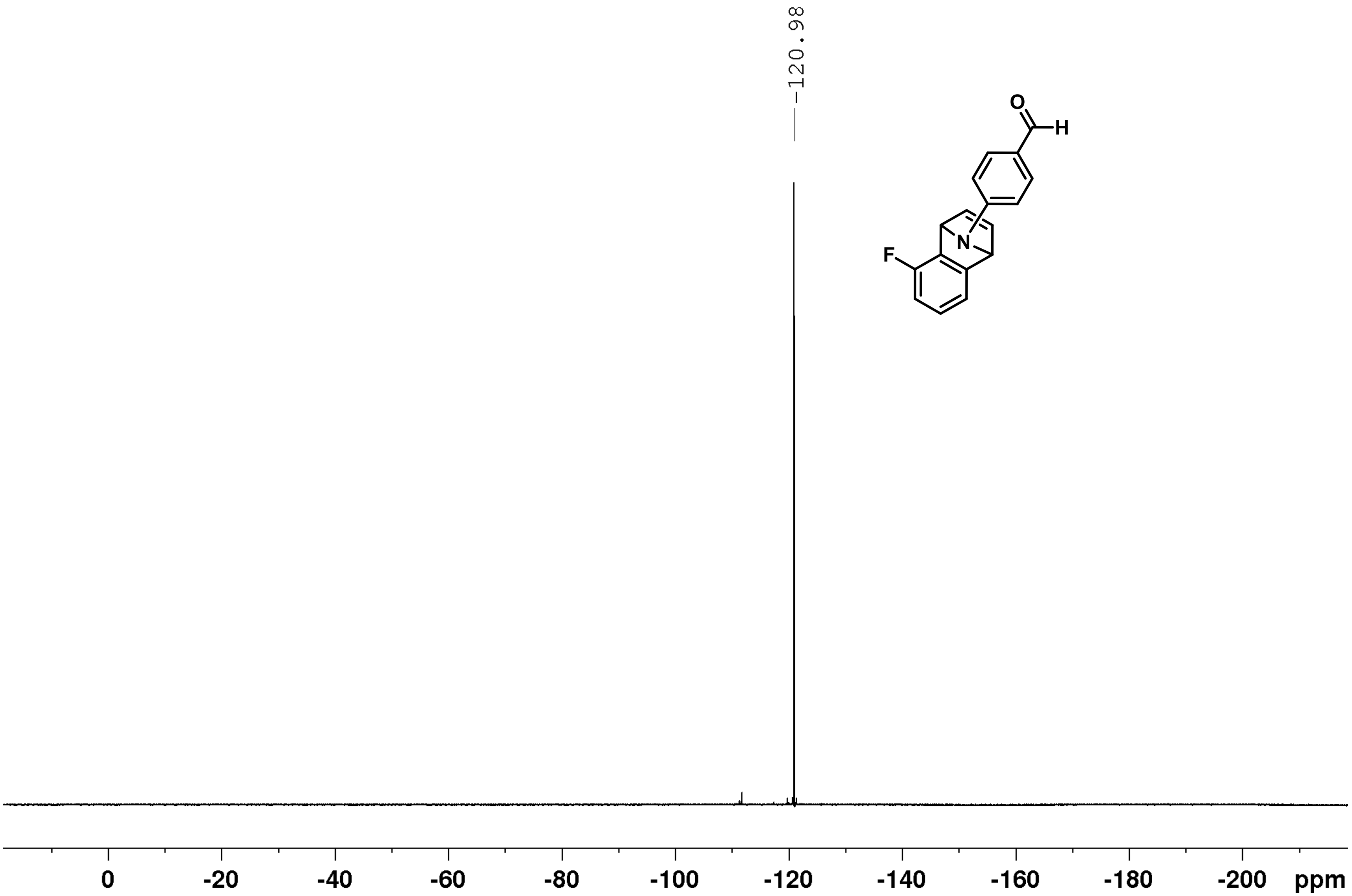

References

1. Vogel, A. I.; Furniss, B. S.; Hannaford, A. J.; Rogers, V.; Smith, P.; W. G.; Tatchell, A. R. Vogel's Textbook of Practical Organic Chemistry (4th Ed) **1978**, 308.
2. https://www.chem.rochester.edu/notvoodoo/pages/how_to/titrate_alkyllithiums.php
3. A. Nilova, B. Metze, and D. R. Stuart, *Org. Lett.* **2021** *23*, 4813
4. Y-D. Kwon, J. Son, and J-H. Chun, *J. Org. Chem.* **2019** *84*, 3678
5. S. K. Sundalam, A. Nilova, T. L. Seidl, D. R. Stuart, *Angew. Chem. Int. Ed.* **2016**, *55*, 8431
6. J. M. Medina, J. L. Mackey, N. K. Garg, and K. N. Houk *J. Am. Chem. Soc.* **2014** *136*, 15798
7. Xu, Z.L.; Li. H. X.; Ren, Z. G.; Du, W. Y.; Xu, W.C.; Lang, J.P.; *Tetrahedron* **2011**, 67, 5282
8. S. Morales, F. G. Guijarro, I. Alonso, J. L. G. Ruano, and M. B. Cid, *ACS Catalysis* **2016** *6*, 84
9. Li, X., Chen, H., Liu, X., Wang, L., Han, J., *ChemistrySelect* **2023**, 8, e202301890
10. A. Watanabe, K. Miyamoto, T. Okada, T. Asawa, and M. Uchiyama, *J. Org. Chem.* **2018** *83*, 14262
11. P. Kazmierczak, L. Skulski, *Synthesis* **1998**, 12, 1721
12. A.J.J. Lennox, and G.C. Lloyd-Jones, *Angew. Chem. Int. Ed.* **2012,** 51, 9385
13. S. Karandikar, D. R. Stuart*, Chem. Commun.* **2022**, 58, 1211
14. R. A. Roberts, B. E. Metze, A. Nilova, and D. R. Stuart, *J. Am. Chem. Soc.* **2023,** 145, 3306
15. V. Blechta, S. Šabata, J. Sýkora, J. Hetflejš, L. Soukupová and J. Schraml, Magn*. Reson. Chem.* **2012**, 50, 128
16. G.S.M. Sundaram, I.R. Morgan and E.M. Tippmann, *Amino Acids* **2012,** 39, 1381
17. B. Metze, A. Battacharjee, T.M McCormick, D.R. Stuart, *Synthesis* **2022**; 54, 4989
18. E. Yoshioka, K. Kakigi, S. Miyoshi, Y. Kawasaki, and H. M., *J. Org. Chem.* **2020** *85*, 13544
19. a) M. Lanzi, Q. Dherbassy, J. Wencel-Delord, *Angew. Chem. Int. Ed.* **2021**, *60*, 14852; *Angew. Chem.* **2021**, *133*, 14978;
    b) M. Lanzi, R. Abed Ali Abdine, M. De Abreu, J. Wencel-Delord, *Org. Lett.* **2021**, *23*, 9047;
    c) Y. Wang, Y.-N. Tian, S. Ren, R. Zhu, B. Huang, Y. Wen, S. Li, *Org. Chem. Front.* **2023**, *10*, 793;
    d) S. S. Karandikar, A. Bhattacharjee, B. E. Metze, N. Javaly, E. J. Valente, T. M. McCormick, D. R. Stuart, *Chem. Sci*. **2022**, *13*, 6532;
    e) M. Lanzi, T. Rogge, T. S. Truong, K. N. Houk, J. Wencel-Delord, *J. Am. Chem. Soc.* **2023**, *145*, 345;
    f) M. Lin, H. Jiang, J. Tang, Z. Ye, F. Zhang, Y. Wu, *Org. Lett.* **2023**, *25*, 2777;
    g) O. Smith, M. J. Hindson, A. Sreenithya, V. Tataru, R. S. Paton, J. W. Burton, M. D. Smith, *ChemRxiv preprint*, **2023**, DOI: 10.26434/chemrxiv-2023-5wwqd;
    h) H. Yuan, W. Yin, J. Hu, Y. Li, *Nature Commun.* **2023**, *14*, 1841.
20. Y. Gao, Y. Mao, B. Zhang, Y. Zhan, and Y. Huo, *Org. Biomol. Chem***. 2018,** 16, 3881
21. Solano, M.V., Della Pia, E.A., Jevric, M., Schubert, C., Wang, X., van der Pol, C., Kadziola, A., Nørgaard, K., Guldi, D.M., Nielsen, M.B. and Jeppesen, J.O. Chem. Eur. J. **2014**, 20, 9918
